# Discovery of Potent Benzothiazole Inhibitors of Oxidoreductase NQO2, a Target for Inflammation and Cancer

**DOI:** 10.3390/ijms252212025

**Published:** 2024-11-08

**Authors:** Asma A. Belgath, Aya M. Emam, Joshua Taujanskas, Richard A. Bryce, Sally Freeman, Ian J. Stratford

**Affiliations:** 1 Division of Pharmacy & Optometry, School of Health Sciences, University of Manchester, Manchester M13 9PT, UKaya.emam@postgrad.manchester.ac.uk (A.M.E.); joshua.taujanskas@manchester.ac.uk (J.T.); ian.stratford@manchester.ac.uk (I.J.S.); 2 Pharmacy School, University of Al-Zawia, Al-Zawia 16418, Libya; 3 Department of Medicinal Chemistry, Faculty of Pharmacy, Zagazig University, Zagazig 44519, Egypt

**Keywords:** NQO2, benzothiazole, Jacobsen cyclisation, cancer, inflammation, oxidative damage

## Abstract

Inhibitors of NQO2 (NRH: quinone oxidoreductase) have potential application in several areas of medicine and pharmacology, including cancer, neurodegeneration (PD and AD), stroke, and diabetes. Here, resveratrol, a known inhibitor of NQO2, was used as the lead by replacing the double bond in resveratrol with a benzothiazole scaffold. Fifty-five benzothiazoles were designed as NQO2 inhibitors and synthesized, comprising five benzothiazole series with 3,5-dimethoxy, 2,4-dimethoxy, 2,5-dimethoxy, 3,4-dimethoxy, and 3,4,5-trimethoxy substituents, the key synthetic step being a Jacobson cyclisation with the appropriate thiobenzamide. All compounds were evaluated in an NQO2 enzyme inhibition assay, with four compounds having IC_50_ values of <100 nM. The most active (IC_50_ 25 nM) was 6-hydroxy-2-(3’,5’-dihydroxyphenyl)benzo[d]thiazole (**15**), a good mimetic of resveratrol. Three of the 3’,4’,5’-trimethoxybenzothiazole analogues, with 6-methoxy (**40**, IC_50_ 51 nM), 6-amino (**48**, IC_50_ 79 nM), and 6-acetamide (**49**, IC_50_ 31 nM) substituents, were also potent inhibitors of NQO2. Computational modelling indicated the most active compounds exhibited good shape complementarity and polar interactions with the NQO2 active site. Through the inhibition of NQO2, benzothiazole-based compounds may have the potential to enhance the efficiency of cancer therapies or minimise oxidative damage in neuroinflammation.

## 1. Introduction

N-Ribosylnicotinamide: quinone oxidoreductase 2 (NQO2) catalyses the reduction of quinones using reduced N-ribosylnicotinamide (NRH) or its derivatives as co-substrates to generate hydroquinones and reactive oxygen species (ROS) [1,2,3]. The excessive ROS production has deleterious effects on various cells and body organs. ROS can react with proteins, lipids, and DNA base pairs, leading to protein denaturation, lipid peroxidation, loss in enzymatic function, and formation of DNA adducts. Consequently, ROS can lead to tissue destruction and oxidative stress, which are correlated with several health disorders, including diabetes, stroke, heart attacks, atherosclerosis, and neurodegenerative diseases [1]. 

Notably, it has been found that inhibition of NQO2 protects cells from quinone toxicity, such as menadione [4]. In addition, NQO2 knockdown resulted in an increase in antioxidant and detoxifying enzyme levels and a decrease in the cell proliferation rate [2]. Moreover, NQO2 has been implicated in the aetiology of many diseases, including malaria and cancer, as chloroquine and imatinib are both NQO2 inhibitors [5]. Furthermore, it has been found that deletion of the NQO2 gene abolished TNF-mediated cell survival signalling of NF-kB and upregulated the apoptotic effect mediated by TNF-α [6,7].

There are a wide variety of NQO2 inhibitors, including the natural product quercetin [8,9], clinically used drugs, such as antimalarial aminoquinolines, imatinib, and nilotinib, used for the treatment of leukaemia [10,11], the thrombin inhibitor dabigatran [12], and melatonin, the neuro-hormone antioxidant [13]. Although these compounds are very potent NQO2 inhibitors within their clinically used concentrations, they lack selectivity towards NQO2.

Resveratrol **1**, a trans-3,4’,5-trihydroxystilbene, has been studied for its chemo-protective and cardio-protective roles [14]. Resveratrol has a high affinity towards NQO2, with a dissociation constant of 35 nM. Interestingly, the K562 chronic myelogenous leukaemia cell line treated with resveratrol exhibited similar properties to NQO2-deficient cell lines, resulting in a decrease in cell proliferation, resistance to menadione toxicity, and an increase in the expression of detoxification and antioxidant enzymes [2]. Therefore, developing resveratrol analogues may provide an effective therapeutic strategy to target ROS via the inhibition of NQO2. However, despite the high absorption of resveratrol, rapid and extensive hepatic 3-O-glucuronidation and 3-O-sulfation of hydroxyl groups and reduction of the stilbene bond by intestinal microflora result in low bioavailability of resveratrol [15,16], making it an unsuitable drug candidate. 

Inspired by resveratrol, with the three hydroxyl groups crucial in its binding to NQO2 active site residues [2], the stilbene bond was replaced with a benzothiazole ring with appropriate positioning of functional groups to anchor these compounds to the active site of NQO2. The benzothiazole heterocycle is present in naturally occurring compounds and clinically used drugs, including Riluzole for the palliative treatment of amyotrophic lateral sclerosis (ALS) [17]. In addition, ethoxzolamide is used for glaucoma and duodenal ulcer treatment [18], and Zopolrestat for the treatment of diabetic complications [19]. Furthermore, ^18^F-labelled 2-phenylbenzothiazoles have been used as PET imaging agents for the diagnosis of Alzheimer’s disease [20]. 

**Figure 1:**
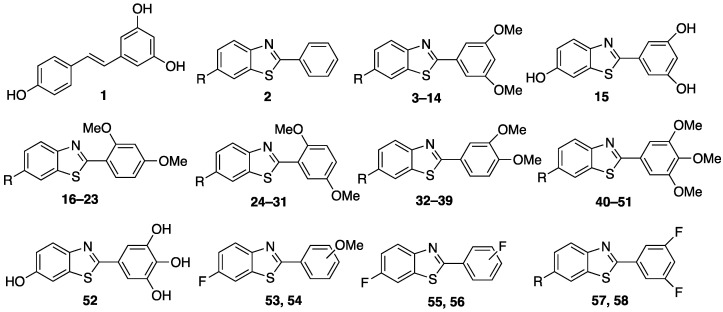
Structures of resveratrol (**1**) and benzothiazole analogues **2–58** (R = OCH_3_, Br, CF_3_, Cl, CN, F, I, NO_2_, NH_2_, NH(O)R). Benzothiazole compounds of similar sizes and dimensions to resveratrol were explored, bearing functional groups in a variety of orientations.

In this work, a range of substituents were included on the benzothiazole ring **2** (R = OCH_3_, Br, CF_3_, Cl, CN, F, I, NO_2_, NH_2_, N(O)CR) (Figure 1, Table 1). Five benzothiazole series were generated from **2** by changing the right-hand phenyl ring to 3,5-dimethoxyphenyl (**3–14**), 2,4-dimethoxyphenyl (**16–23**), 2,5-dimethoxyphenyl (**24–31**), 3,4-dimethoxyphenyl (**32–39**), and 3,4,5-trimethoxyphenyl (**40–51**) benzothiazoles (Figure 1). Compounds with free OH groups (**15, 52**), as well as fluoro groups on the phenyl ring (**55–58**), were also prepared as benzothiazole mimetics of resveratrol.

## 2. Results and Discussion

### 2.1. Synthesis of Benzothiazoles ***3***–***58***

**Scheme 1:**
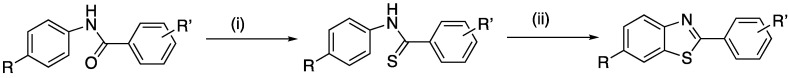
Synthesis of benzothiazoles from a substituted benzamide. Reagents and conditions: (**i**) Lawesson’s reagent, dry toluene, microwave, 150 °C, 5 min; (**ii**) K_3_Fe(CN)_6_, EtOH/CH_3_CN, 10%aq NaOH, 90 °C-r.t.

The benzamide intermediate (prepared from the appropriate benzoic acid/acid chloride with an aniline) [21,22] was converted to the thiobenzamide intermediate using Lawesson’s reagent (Scheme 1). The synthesis of the benzothiazole products was achieved through Jacobsen cyclization of the thiobenzamide intermediate using K_3_Fe(CN)_6_ in an alkaline solution (Scheme 1) [21]. Hydroxybenzothiazoles **15** and **52**, with two and three free phenol groups, respectively, were prepared through deprotection of compounds **3** and **40** using boron tribromide as the demethylating agent [21]. For further details of the synthetic routes/conditions and the spectroscopic characterisation of the fifty-five benzothiazoles, see Section 3.1 and the Supplementary Data.

### 2.2. Inhibition of NQO2 Enzyme by Benzothiazoles

The IC_50_ values for the inhibition of NQO2 enzymatic activity by benzothiazoles are summarised in Table 1. In this assay, dichlorophenolindophenol (DCPIP) was used as a substrate for NQO2. NRH (SI or EP0152R) was used as the co-substrate for the enzyme. Resveratrol was used as a positive control, giving an IC_50_ of 997 nM with NQO2. 

Benzothiazoles showed variation in the inhibition of NQO2 enzymatic activity. Notably, methoxy- (**3**) and trifluoromethyl- (**5**) benzothiazoles were the most active compounds among the 3,5-dimethoxybenzothiazole series, with IC_50_ values of 108 nM and 123 nM. The replacement of dimethoxy groups in **3** with difluoro groups in **57** resulted in a significant decrease in the inhibition of NQO2 activity, with an increase in the IC_50_ value from 108 nM to 12 µM. Moreover, substitution of all methoxy groups in both rings of **3** with fluorine groups in benzothiazole **58** resulted in a loss of inhibition of NQO2 activity. Interestingly, the replacement of all methoxy groups in **3** with hydroxyl groups in **15**, a good resveratrol mimetic, resulted in the enhancement of inhibition, with an IC_50_ of 25 nM. The change to an amino group in **11** or an acetamide group in **12** gave IC_50_ values of 863 nM and 605 nM respectively, whereas replacement with a benzamide group (**13**, **14**) resulted in further loss of activity. 

Iodo (**22**), fluoro (**21**), trifluoromethyl (**18**), and methoxy (**16**) benzothiazoles were the most active compounds among the 2,4-dimethoxybenzothiazole series, with IC_50_ values from 1 to 6 µM, while benzothiazoles **19** and **17** were weak enzyme inhibitors, with IC_50_ values of 33.5 µM and 56.9 µM, respectively. Methoxybenzothiazole **24** was the most active compound in the 2,5-dimethoxybenzothiazoles series, with an IC_50_ value of 846 nM for the inhibition of NQO2. The chloro- **27**, trifluoro- **26**, and fluoro- **29** benzothiazoles had slightly lower IC_50_ values of 1.18 µM, 2.77 µM, and 2.06 µM, respectively.

Methoxybenzothiazole **32** was the most active compound of the 3,4-dimethoxybenzothiazole series, which inhibited NQO2 with an IC_50_ of 1.27 µM. In this series, fluorobenzothiazole **37** (IC_50_ 5.93 µM) was slightly more active than bromobenzothiazole **33** (IC_50_ 9.09 µM), with the iodo **38** and chloro **35** analogues being less active, with IC_50_ values of 22.9 µM and 91.7 µM. Furthermore, nitrile and nitro compounds of the 3,5-dimethoxy-, 2,4-dimethoxy-, 2,5-dimethoxy-, and 3,4-dimethoxy- benzothiazole series were very weak enzyme inhibitors. 

Remarkably, many of 3,4,5-trimethoxybenzothiazoles series showed nano-molar IC_50_ values for the inhibition of NQO2 (Table 1). The most active inhibitors were methoxy-**40** (IC_50_ 51 nM), acetamide-**49** (IC_50_ 31 nM), and amino-**48** (IC_50_ 79 nM) benzothiazoles. Trifluoromethyl **42** and nitrile-**44** benzothiazoles were the weakest NQO2 inhibitors among this series, with IC_50_ values of 18.4 µM and 13.3 µM. 

The mono-substituted benzothiazoles showed a wide range of inhibition of NQO2. 3-Methoxybenzothiazole **53** was the most potent NQO2 inhibitor (IC_50_ 908 nM), whereas 3-fluorobenzothiazole **55** was much less active (IC_50_ 52.55 µM). The 4-substituted benzothiazoles **54** and **56** were not active, with IC_50_ values greater than 100 µM.

### 2.3. Molecular Modelling

**Figure 2:**
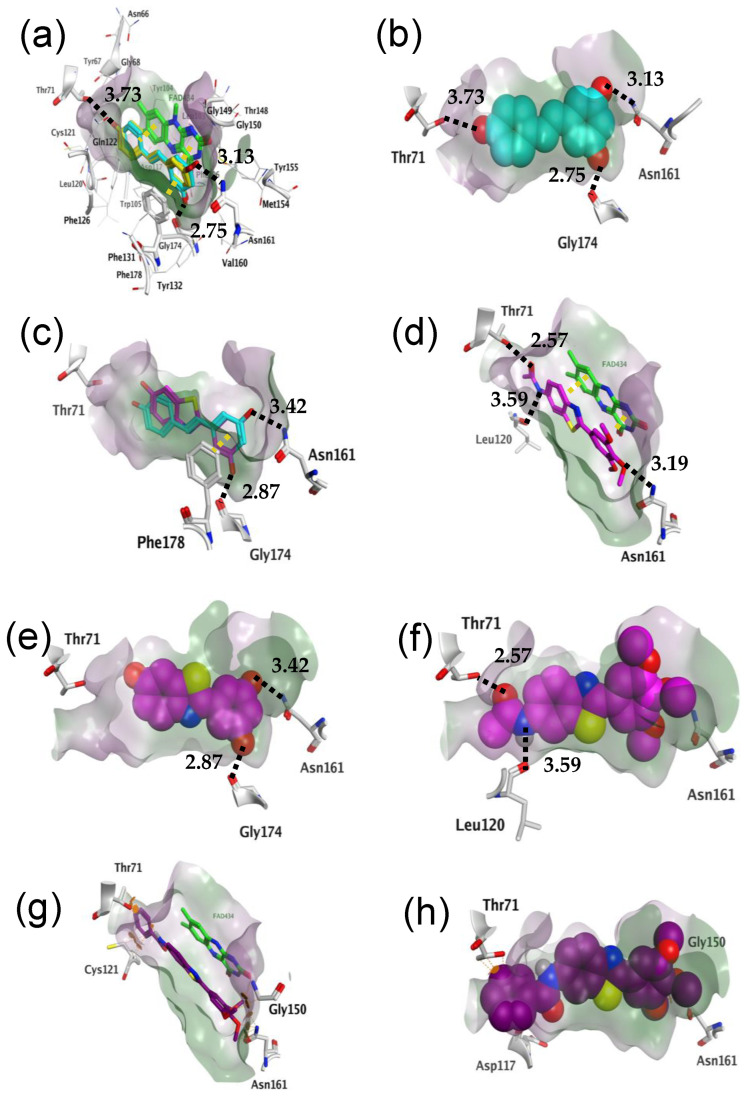
(**a**) Top ranked pose of docked resveratrol (yellow) superposed on crystallographic pose (cyan) with RMSD value of 0.4 Å. Resveratrol forms π–π stacking (yellow dashed line) with the isoalloxazine ring of FAD (green), π–π stacking with Phe178, and three hydrogen bonds (black dashed line) with Val160, Gly174, and Thr71. (**b**) Space-filling representations of crystallographic pose of resveratrol in the binding site of NQO2. (**c**) Overlay of docked pose of **15** (magenta) with resveratrol (cyan). (**d**) Docked pose of **49** (magenta stick representation), and interactions with the key amino acid residues. Docked poses of compounds (**e**) **15** and (**f**) **49** (magenta space-filling representation). Docked pose of compound **50** in (**g**) stick and (**h**) space-filling representation (magenta), making steric clashes with NQO2 residues (orange regions). Distances in Å.

We then considered the interactions of these benzothiazole inhibitors with the NQO2 protein via computational docking. In the X-ray structure of the NQO2 complex with resveratrol (PDB code 1SG0) [2], the binding site is a largely hydrophobic pocket, defined by amino acid residues Phe126, Phe131, Phe178, Tyr132, Val160, and Trp105 on one side and the FAD cofactor opposite (Figure 2a). Flanking the pocket are polar amino acid residues Thr71 and Gln122 on one side and Asn161 and the backbone of Gly174 on the other. The resveratrol ligand forms π–π stacking with FAD and Phe178, as well as hydrogen bonds with the backbone of Gly174, and the side chains of Asn161 and Thr 71, with distances of 2.75 Å, 3.13 Å, and 3.73 Å between heavy atoms, respectively (Figure 2a,b).

Prior to predicting the binding modes of benzothiazole compounds, the docking protocol using FRED [23] was validated through re-docking of resveratrol to the NQO2 protein. The crystallographic mode of resveratrol is closely reproduced through docking, with a root mean square deviation (RMSD) value for the top ranked pose of 0.4 Å (Figure 2a). Indeed, the docking calculation strongly predicts that this orientation is favoured, such that only one pose out of the top ten was docked in the reverse orientation (pose 8), with the 3,5-dihydroxyphenyl group flipped to the other side of the pocket.

Subsequently, the nine most potent benzothiazole inhibitors from the series **3–58** above were assessed through docking. These were compounds **15, 49, 40, 48, 46, 3, 5, 41,** and **52,** as listed in order of decreasing potency, with a corresponding experimentally determined IC_50_ range of 25 nM to 0.2 µM (Table 1). The resulting predicted ligand poses indicated a consistent docked orientation in NQO2, where the substituted phenyl ring is located on the Asn161/Gly174 side of the pocket and the benzothiazole ring to the Thr71/Gln122 side, for example **15** (Figure 2c). All compounds form π–π stacking interactions with the isoalloxazine ring of FAD, including, for example compound **49** (Figure 2d). The ligands are accommodated well by the NQO2 pocket, such that the Chemgauss4 docking scores for the benzothiazole compounds exceed that of resveratrol (−15.5), ranging from −19.4 for compound **49** to −16.8 for compound **3**.

Interestingly, the most potent compound assayed in this study, **15**, occupies the protein pocket in a very similar way to resveratrol: the ligand forms analogous π–π stacking with both Phe178 and FAD (Figure 2c,e) and forges hydrogen bonds with Asn161 and Gly174 via its phenyl OH groups, with distances of 3.42 Å and 2.87 Å, respectively; however, the distance of the benzothiazole ring’s OH oxygen to the threonine side chain O of Thr71 is slightly longer than for resveratrol, with differences of 0.80 Å and 0.19 Å for the first and second poses, respectively (Figure 2c).

The remaining compounds with a potency exceeding 100 nM feature a 3,4,5-trimethoxyphenyl group, namely **40, 48,** and **49** (Table 1). The 3,4,5-methoxy motif is predicted from docking to optimally occupy the pocket on the Asn161/Gly174 side, such as, for example, for **49** (Figure 2d,f). Compound **49** has an acetamide R group on the benzothiazole ring. This acetamide group is computed to be favourably oriented, forming hydrogen bonds with Thr71 and Leu120 with respective O…O and O…N distances of 2.57 Å and 3.59 Å (Figure 2d,f). The NQO2 pocket on the Asn161/Gly174 side is well-occupied by the trimethoxy motif for the other benzothiazole ligands of this study (Figure S138) but rather less well by compounds with fewer OMe groups. For example, removal of the 4-OMe or the 5-OMe from 3,4,5-trimethoxy compound **40** to give ligands **3** and **32,** respectively, led to a lower computed occupation of this pocket (Figure S139) and a 2-fold and 20-fold reduction in potency experimentally (Table 1). Similarly, compounds **55–58** with the smaller fluoro substituents failed to occupy the pocket in docking (e.g., **57** in Figure S139) and displayed micromolar or higher IC_50_s (Table 1).

We do also note a limit to the dimensions of these trimethoxy compounds: the end-to-end length of compound **49** is 14.7 Å (as measured between carbon atoms of the 4-OMe and acetamide CH_3_ groups). However, if the acetamide group is substituted by a phenyl group, as found in compound **50**, the ligand length increases to 17.1 Å. Inspection of the docked pose of **50** indicates a steric clash with the two sides of the NQO2 active site, with residues Thr71, Cys121, Asn161, and Gly150 (orange region, Figure 2g,h), and accompanying reduction in docking score from -19.4 for compound **49** to -11.7 for compound **50**. Correspondingly, the IC_50_ of **50** is found to be 3.17 µM, which is two orders of magnitude less potent than for **49**.

In summary, as predicted through molecular docking, to enhance the biological activities, the small molecule NQO2 inhibitor should be planar and lead to favourable π–π stacking with the isoalloxazine ring of FAD, fill the pocket very well with an optimal length and shape, and create stronger hydrogen bonds with the key amino acid residues, as found for resveratrol.

Finally, in order to obtain insight into the suitability of the set of the nine most potent benzothiazole inhibitors as therapeutics and, specifically, their ability to target CNS-treated diseases, we computed transport and toxicity properties based on the chemical structure (Table S1). We find that none of the compounds (**15**, **49**, **40**, **48**, **46**, **3**, **5**, **41,** or **52)** are predicted to be hERG channel blockers, as predicted using the PRED-hERG server [24]. Regarding cytotoxicity, only compound **49** has some indication of this propensity (probability score *p* of 0.51), as calculated using the ProTox-3.0 software [25,26]. All compounds have a prediction of potential hepatoxicity, in the range of *p* from 0.61 to 0.69, and they are flagged as possible inhibitors of CYP1A2 (Table S1) [25,26]. Regarding stability, we observe that the sulfur atom in the thiazole ring may be prone to oxidation; however, we note that benzothiazole compounds Riluzole and Zopolrestat are biologically active. In addition, the use of benzothiazoles as PET imaging agents for the diagnosis of Alzheimer’s disease indicates an ability for benzothiazoles to be blood–brain barrier (BBB) penetrant. Here, of the nine most highly active benzothiazoles, compounds **3**, **41,** and **43** are predicted as BBB permeants via the SWISS-ADME server (Table S1) [27]. This result further indicates potential for this benzothiazole series to be engineered for CNS penetration.

## 3. Materials and Methods

See SD Section 1 for Chemicals and Materials

### 3.1. Synthesis of Benzothiazole Analogues [21]

Appropriate phenylbenzothioamides (see SI for synthesis) (1.0 mmol) were wetted with ethanol, acetonitrile, or both (volume and solvent confirmed for each compound). A solution of 10% aq NaOH (6.0 mL) was added to the solvent mixture, which was then added dropwise to potassium ferricyanide (4.0 mmol) in water (5.0 mL) at 90 °C. The reaction mixture was heated for a further 30 min and then allowed to cool down and stirred overnight at room temperature. The reaction’s completion was identified using TLC (30% ethyl acetate in hexane). The reaction mixture was filtered, and the solid residue was dissolved in DCM, dried with dry MgSO_4_, and concentrated under vacuum. The crude residue was purified using column chromatography. The details of the wetting solvents and purification are specified for each compound.

**2-(3,5-Dimethoxyphenyl)-6-methoxybenzo[d]thiazole (3)** [21]. 3,5-Dimethoxy-*N*-(4-methoxyphenyl)benzothioamide was wetted with 1.5 mL of ethanol. Compound (**3**) was purified using silica gel column chromatography eluting with 10% ethyl acetate in hexane to give a white solid (0.063 g, 80%), m.p. = 121–123 °C. IR (cm^−1^): 2924 (ArCH), 2847 (CH_3_), 1581, 838; ^1^H-NMR (300 MHz, CDCl_3_): δ 7.95 (1H, br d, J = 9.0 Hz, H4), 7.35 (1H, d, J = 2.4 Hz, H7), 7.20 (2H, d, J = 2.1 Hz, H2′, 6′), 7.10 (1H, dd, J_1_ = 8.7 Hz, J_2_ = 2.4 Hz, H5), 6.57 (1H, t, J = 2.1 Hz, H4′), 3.89 (9H, s, 3 × OCH_3_); ^13^C-NMR (125 MHz, CDCl_3_) (assignments made with the aid of DEPT-135): δ 165.4 (C2), 161.1 (C3′, 5′), 157.9 (C6), 148.6 (C9), 136.5 (C8), 135.6 (C1′), 123.8 (C4), 115.7 (C5), 105.2 (C2′, 6′), 104.1 (C7), 103.0 (C4′), 55.8 (OCH_3_), 55.6 (2 × OCH_3_).
**6-Bromo-2-(3,5-dimethoxyphenyl)benzo[d]thiazole (4).** *N*-(4-Bromophenyl)-3,5-dimethoxybenzothioamide was wetted with 1.5 mL of acetonitrile. Compound (**4**) was purified using silica gel column chromatography eluting with 5% ethyl acetate in hexane to give a white solid (0.04 g, 46%), m.p. = 164–165 °C. IR (cm^−1^): 2937 (ArCH), 2842 (CH_3_), 1598, 1504, 675; ^1^H-NMR (300 MHz, CDCl_3_): δ 8.02 (1H, d, ^4^J_HH_ = 1.8 Hz, H7), 7.91 (1H, d, J = 8.7 Hz, H4), 7.59 (1H, dd, J_1_ = 8.7 Hz, J_2_ = 1.8 Hz, H5), 7.22 (2H, d, J = 2.1 Hz, H2′, 6′), 6.60 (1H, t, J = 2.1 Hz, H4′), 3.89 (6H, s, 2 × OCH_3_); ^13^C-NMR (125 MHz, CDCl_3_) (assignments made with the aid of DEPT-135): δ 168.4 (C2), 161.2 (C3′, 5′), 152.8 (C9), 136.7 (C8), 134.9 (C1′), 129.8 (C5), 124.3 (C7), 124.1 (C4), 118.8 (C6), 105.4 (C2′, 6′), 103.6 (C4′), 55.6 (2 × OCH_3_). ESMS *m/z* [M + H] ^+^ 350, ^79^Br; [M + H]^+^ 352, ^81^Br. HRMS calcd for C_15_H_13_BrNO_2_S: 349.9845, 351.9824. Found 349.9833, 351.9812.
**2-(3,5-Dimethoxyphenyl)-6-(trifluoromethyl)benzo[d]thiazole (5).** 3,5-Dimethoxy-*N*-(4-(trifluoromethyl)phenyl)benzothioamide was wetted with 1.5 mL of ethanol. Compound (**5**) was purified using silica gel column chromatography eluting with 5% ethyl acetate in hexane to give a white solid (0.109 g, 55%), m.p. = 103–105 °C. IR (cm^−1^): 2937 (ArCH), 2841 (CH_3_), 1590, 850; ^1^H-NMR (300 MHz, CDCl_3_): δ 8.19 (1H, br s, H7), 8.14 (1H, br d, J = 8.4 Hz, H5), 7.73 (1H, d, J = 7.8 Hz, H4), 7.25 (2H, br s, H2′, 6′), 6.62 (1H, br s, H4′), 3.90 (6H, s, 2 × OCH_3_); ^13^C-NMR (125 MHz, CDCl_3_) (assignments made with the aid of DEPT-135): δ 171.0 (C2), 161.2 (C3′, 5′), 155.9 (C9), 135.1 (C8), 134.7 (C1′), 127.3 (q, J = 32.5 Hz, C6), 124.2 (J = 270.6 Hz, CF_3_), 123.5 (C4), 123.3 (J = 3.3 Hz, C5), 119.3 (q, J = 4.1 Hz, C7), 105.6 (C2′, 6′), 103.9 (C4′), 55.6 (2 × OCH_3_); ^19^F-NMR (^1^H-decoupled, 376 MHz, CDCl_3_): δ −64.7 (s, CF_3_). ESMS *m/z* [M + H]^+^ 340. HRMS calcd for C_16_H_13_F_3_NO_2_S: 340.0614. Found: 340.0603. HPLC > 95% pure.
**6-Chloro-2-(3,5-dimethoxyphenyl)benzo[d]thiazole (6).** *N*-(4-Chlorophenyl)-3,5-dimethoxybenzothioamide was wetted with 1.5 mL of ethanol. Compound (**6**) was purified using silica gel column chromatography eluting with 5% ethyl acetate in hexane to give a white solid (0.122 g, 59%), m.p. = 150–151 °C. IR (cm^−1^): 2964 (ArCH), 2843 (CH_3_), 1590, 833; ^1^H-NMR (300 MHz, CDCl_3_): δ 7.96 (1H, d, J = 8.7 Hz, H4), 7.85 (1H, d, J = 1.2 Hz, H7), 7.44 (1H, dd, J_1_ = 8.4 Hz, d, J_2_ = 1.4 Hz, H5), 7.21 (2H, d, J = 1.2 Hz, H2′, 6′), 6.59 (1H, t, J = 1.8 Hz, H4′), 3.88 (6H, s, 2 × OCH_3_); ^13^C-NMR (125 MHz, CDCl_3_) (assignments made with the aid of DEPT-135): δ 168.4 (C2), 161.2 (C3′,5′), 152.5 (C9), 136.2 (C8), 135.0 (C1′), 131.1 (C6), 127.1 (C5), 123.9 (C4), 121.2 (C7), 105.4 (C2′, 6′), 103.6 (C4′), 55.6 (2 × OCH_3_). ESMS *m/z* [M + H]^+^ 306, ^35^Cl; 308, ^37^Cl. HRMS calcd for C_15_H_13_ClNO_2_S: 306.0350, 308.0321. Found: 306.0346, 308.0314.
**2-(3,5-Dimethoxyphenyl)benzo[d]thiazole-6-carbonitrile (7).** *N*-(4-Cyanophenyl)-3,5-dimethoxybenzothioamide) was wetted with 1.0 mL of acetonitrile. Compound (**7**) was purified using silica gel column chromatography eluting with 8% ethyl acetate in hexane and obtained as a white solid (0.018 g, 18%), m.p. = 222–224 °C. IR (cm^−1^): 3086 (ArCH), 2843 (CH_3_), 2223 (CN), 1600, 847; ^1^H-NMR (300 MHz, CDCl_3_): δ 8.23 (1H, d, J = 1.2 Hz, H7), 8.12 (1H, d, J = 8.4 Hz, H4), 7.74 (1H, dd, J = 8.4 Hz, d, J = 1.5 Hz, H5), 7.25 (2H, d, J = 2.1 Hz, H2′, 6′), 6.64 (1H, t, J = 2.3 Hz, H4′), 3.90 (6H, s, 2 × OCH_3_); ^13^C-NMR (125 MHz, CDCl_3_) (assignments made with the aid of DEPT-135): δ 172.2 (C2), 161.3 (C3′, 5′), 156.3 (C9), 135.5 (C8), 134.4 (C1′), 129.5 (C5), 126.4 (C7), 123.9 (C4), 118.7 (CN), 108.6 (C6), 105.8 (C2′, 6′), 104.3 (C4′), 55.7 (2 × OCH_3_). ESMS *m/z* [M + H]^+^ 297. HRMS calcd for C_16_H_13_N_2_O_2_S: 297.0692. Found: 297.0684.
**2-(3,5-Dimethoxyphenyl)-6-fluorobenzo[d]thiazole (8).** *N*-(4-Fluorophenyl)-3,5-dimethoxybenzothioamide) was wetted with 2.0 mL of ethanol. Compound (**8**) was purified using silica gel column chromatography eluting with 10% ethyl acetate in hexane and obtained as a white solid (0.085 g, 83%), m.p. = 138–140 °C. IR (cm^−1^): 2957 (ArCH), 2843 (CH_3_), 1594, 943; ^1^H-NMR (300 MHz, CDCl_3_): δ 8.01 (1H, dd, J_1_ = 9.0 Hz, J_2_ = 4.8 Hz, H4), 7.58 (1H, dd, J_1_ = 8.1 Hz, J_2_ = 2.7 Hz, H7), 7.25–7.19 (1H, m, H5), 7.21 (2H, d, J = 2.1 Hz, H2′, 6′), 6.59 (1H, t, J = 2.1 Hz, H4′), 3.89 (6H, s, 2 × OCH_3_); ^13^C-NMR (125 MHz, CDCl_3_) (assignments made with the aid of DEPT-135): δ 167.7 (d, J = 3.5 Hz, C9), 161.2 (C3′, 5′), 160.5 (d, J = 244.3 Hz, C6), 150.6 (d, J = 1.5 Hz, C2), 136.0 (d, J = 10.8 Hz, C8), 135.1 (C1′), 124.2 (d, J = 9.5 Hz, C4), 115.0 (d, J = 24.4 Hz, C5), 107.8 (d, J = 26.4 Hz, C7), 105.3 (C2′, 6′), 103.4 (C4′), 55.6 (2 × OCH_3_); ^19^F-NMR (^1^H-decoupled, 376 MHz, CDCl_3_): − 118.9 (s, F at C6); ^19^F-NMR (^1^H-coupled, 376 MHz, CDCl_3_): δ −118.9 (td, J_1_ = 8.3 Hz, J_2_ = 4.9 Hz, F at C6). ESMS *m/z* [M + H]^+^ 290. HRMS calcd for C_15_H_13_FNO_2_S: 290.0646. Found: 290.0641.
**2-(3,5-Dimethoxyphenyl)-6-iodobenzo[d]thiazole (9).** *N*-(4-Iodophenyl)-3,5-dimethoxybenzothioamide was wetted with 1.0 mL of ethanol. Compound (**9**) was purified using silica gel column chromatography eluting with 5% ethyl acetate in hexane to give an off-white solid (0.035 g, 59%), m.p. = 150–152 °C. IR (cm^−1^): 2920 (ArCH), 2850 (CH_3_), 1597, 1351, 929; ^1^H-NMR (300 MHz, CDCl_3_): δ 8.23 (1H, br s, H7), 7.78 (2H, br s, H4, 5), 7.22 (2H, br s, H2′, 6′), 6.60 (1H, br s, H4′), 3.89 (6H, s, 2 × OCH_3_); ^13^C-NMR (125 MHz, CDCl_3_) (assignments made with the aid of DEPT-135): δ 168.4 (C2), 161.2 (C3′, 5′), 153.3 (C9), 137.1 (C8), 135.4 (C5), 134.8 (C1′), 130.1 (C7), 124.7 (C4), 105.5 (C2′, 6′), 103.7 (C4′), 89.5 (C6), 55.6 (2 × OCH_3_). ESMS *m/z* [M + H]^+^ 397.7. HRMS calcd for C_15_H_13_INO_2_S: 397.9706. Found: 397.9704.
**2-(2,4-Dimethoxyphenyl)-6-methoxybenzo[d]thiazole (16).** *N*-(4-Fluorophenyl)-2,4-dimethoxybenzothioamide was wetted with 2.0 mL of acetonitrile. Compound (**16**) was purified using silica gel column chromatography eluting with 10% ethyl acetate in hexane to give a white solid (0.084 g, 74%), m.p. = 150–151 °C. IR (cm^−1^): 2966 (ArCH), 2825 (CH_3_), 1602, 813; ^1^H-NMR (300 MHz, CDCl_3_): δ 8.40 (1H, d, J = 8.7 Hz, H6′), 7.92 (1H, br d, J = 9.0 Hz, H4), 7.35 (1H, d, J = 2.4 Hz, H7), 7.07 (1H, dd, J_1_ = 8.7 Hz, J_2_ = 2.4 Hz, H5), 6.66 (1H, dd, J_1_ = 8.7 Hz, J_2_ = 2.3 Hz, H5′), 6.56 (1H, d, J = 1.8 Hz, H3′), 4.01 (3H, s, OCH_3_), 3.88 (6H, s, 2 × OCH_3_); ^13^C-NMR (125 MHz, CDCl_3_) (assignments made with the aid of DEPT-135): δ 162.6 (C2), 161.0 (C4′), 158.3 (C2′), 157.1 (C6), 146.7 (C9), 136.9 (C8), 130.4 (C6′), 122.8 (C4), 115.8 (C1′), 115.1 (C5), 105.9 (C5′), 103.6 (C7), 98.5 (C3′), 55.8 (OCH_3_), 55.7 (OCH_3_), 55.5 (OCH_3_).
**6-Bromo-2-(2,4-dimethoxyphenyl)benzo[d]thiazole (17).** *N*-(4-Bromophenyl)-2,4-dimethoxybenzothioamide was wetted with 1.0 mL of acetonitrile and 1.5ml of ethanol. Compound (**17**) was purified using silica gel column chromatography eluting with 7% ethyl acetate in hexane to give a white solid (0.078 g, 62%), m.p. = 148–149 °C. IR (cm^−1^): 2936 (ArCH), 2831 (CH_3_), 1606, 1437, 835; ^1^H-NMR (300 MHz, CDCl_3_): δ 8.44 (1H, br d, J = 8.7 Hz, H6′), 8.00 (1H, d, J = 1.8 Hz, H7), 7.87 (1H, br d, J = 8.4 Hz, H4), 7.54 (1H, dd, J_1_ = 8.4 Hz, J_2_ = 1.8 Hz, H5), 6.67 (1H, dd, J_1_ = 8.7 Hz, J_2_ = 2.1 Hz, H5′), 6.56 (1H, d, J = 2.1 Hz, H3′), 4.03 (3H, s, OCH_3_), 3.89 (3H, s, OCH_3_); ^13^C-NMR (125 MHz, CDCl_3_) (assignments made with the aid of DEPT-135): δ 163.8 (C2), 163.1 (C4′), 158.6 (C2′), 151.1 (C9), 137.4 (C8), 130.7 (C6′), 129.2 (C5), 123.6 (C7), 123.4 (C4), 117.4 (C6), 115.2 (C1′), 106.0 (C5′), 98.4 (C3′), 55.7 (OCH_3_), 55.6 (OCH_3_). ESMS *m/z* [M + H] ^+^ 350, ^79^Br; [M + H]^+^,352, ^81^Br. HRMS calcd for C_15_H_13_BrNO_2_S: 349.9845, 351.9824. Found: 349.9833, 351.9811.
**2-(2,4-Dimethoxyphenyl)-6-(trifluoromethyl)benzo[d]thiazole (18).** 2,4-Dimethoxy-N-(4-(trifluoromethyl)phenyl)benzothioamide was wetted with 1.5 mL of ethanol and 1.5 mL of acetonitrile. Compound (18) was purified using silica gel column chromatography eluting with 7% ethyl acetate in hexane to give a white solid (0.115 g, 59%), m.p. = 136–138 °C. IR (cm^−1^): 2937 (ArCH), 2844 (CH3), 1603, 1578, 924, 834; 1H-NMR (300 MHz, CDCl3): δ 8.48 (1H, d, 3JHH = 8.7 Hz, H6′), 8.16 (1H, br s, H7), 8.08 (1H, d, J = 8.7 Hz, H4), 7.68 (1H, br d, J = 8.4 Hz, H5), 6.67 (1H, dd, J1 = 8.7 Hz, J2 = 1.5 Hz, H5′), 6.56 (1H, br s, H3′), 4.03 (3H, s, OCH3), 3.88 (s, 3H, OCH3); 13C-NMR (125 MHz, CDCl3) (assignments made with the aid of DEPT-135): δ 166.2 (C2), 163.5 (C4′), 158.9 (C2′), 154.1 (C9), 135.6 (C8), 130.9 (C6′), 126.0 (q, J = 32.2 Hz, C6), 124.5 (q, J = 270.3 Hz, CF3), 122.7 (q, J = 3.3 Hz, C5), 122.4 (C4), 118.8 (q, J = 4.1 Hz, C7), 115.0 (C1′), 106.1 (C5′), 98.3 (C3′), 55.64 (OCH3), 55.55 (OCH3); 19F-NMR (1H-decoupled, 376 MHz, CDCl3): δ −64.3 (s, CF3). ESMS *m/z* [M + H] + 340. HRMS calcd for C16H13F3NO2S: 340.0614. Found: 340.0610.
**6-Chloro-2-(2,4-dimethoxyphenyl)benzo[d]thiazole (19).** *N*-(4-Chlorophenyl)-2,4-dimethoxybenzothioamide) was wetted with 1.5 mL of ethanol. Compound (**19**) was purified using silica gel column chromatography eluting with 5% ethyl acetate in hexane to give a white solid (0.075 g, 66%), m.p. = 170–171 °C. IR (cm^−1^): 2939 (ArCH), 2831 (CH_3_), 1607, 1577, 1440, 1290, 832; ^1^H-NMR (300 MHz, CDCl_3_): δ 8.43 (1H, d, J = 8.7 Hz, H6′), 7.92 (1H, br d, J = 8.7 Hz, H4), 7.85 (1H, d, J = 1.8 Hz, H7), 7.41 (1H, dd, J_1_ = 8.7 Hz, J_2_ = 1.8 Hz, H5), 6.67 (1H, dd, J_1_ = 8.7 Hz, J_2_ = 2.1 Hz, H5′), 6.57 (1H, d, J = 2.1 Hz, H3′), 4.03 (3H, s, OCH_3_), 3.89 (3H, s, OCH_3_); ^13^C-NMR (125 MHz, CDCl_3_) (assignments made with the aid of DEPT-135): δ 163.8 (C2), 163.1 (C4′), 158.6 (C2′), 150.8 (C9), 136.9 (C8), 130.7 (C6′), 129.8 (C6), 126.5 (C5), 123.0 (C4), 120.6 (C7), 115.3 (C1′), 106.1 (C5′), 98.4 (C3′), 55.7 (OCH_3_), 55.6 (OCH_3_). ESMS *m/z* [M + H]^+^ 306, ^35^Cl; 308, [M + H]^+^, ^37^Cl. HRMS calcd for C_15_H_13_ClNO_2_S: 306.0357, 308.0327. Found: 306.0339, 308.0307.
**2-(2,4-Dimethoxyphenyl)benzo[d]thiazole-6-carbonitrile (20).** *N*-(4-Cyanophenyl)-2,4-dimethoxybenzothioamide was wetted with 2.0 mL of acetonitrile. Compound (**20**) was purified using silica gel column chromatography eluting with DCM to give a white solid (0.030 g, 25%), m.p. = 235–237 °C. IR (cm^−1^): 2984 (ArCH), 2832 (CH_3_), 2226 (CN), 1609, 1577, 1437, 816; ^1^H-NMR (300 MHz, CDCl_3_): δ 8.50 (1H, br d, J = 8.7 Hz, H6′), 8.21 (1H, d, J = 1.2 Hz, H7), 8.05 (1H, d, J = 8.4 Hz, H4), 7.70 (1H, dd, J_1_= 8.6 Hz, J_2_ = 1.7 Hz, H5), 6.70 (1H, dd, J_1_ = 8.9 Hz, J_2_ = 2.3 Hz, H5′), 6.59 (1H, d, J = 2.4 Hz, H3′), 4.07 (3H, s, OCH_3_), 3.91 (3H, s, OCH_3_); ^13^C-NMR (125 MHz, CDCl_3_) (assignments made with the aid of DEPT-135): δ 167.4 (C2), 163.8 (C4′), 159.1 (C2′), 154.6 (C9), 136.0 (C8), 131.1 (C6′), 129.0 (C5), 126.0 (C4), 122.8 (C7), 119.3 (CN), 114.8 (C1′), 107.0 (C6), 106.3 (C5′), 98.3 (C3′), 55.74 (OCH_3_), 55.65 (OCH_3_). ESMS *m/z* [M + H] ^+^ 297. HRMS calcd for C_16_H_13_N_2_O_2_S: 297.0700. Found: 297.0682.
**2-(2,4-Dimethoxyphenyl)-6-fluorobenzo[d]thiazole (21).** *N*-(4-Fluorophenyl)-2,4-dimethoxybenzothioamide was wetted with 1.5 mL of ethanol and 1.5 mL of acetonitrile. Compound (**21**) was purified using silica gel column chromatography eluting with 5% ethyl acetate in hexane to give a white solid (0.072 g, 66%), m.p. = 157–158 °C. IR (cm^−1^): 2920 (ArCH), 2853 (CH_3_), 1604, 1449, 803; ^1^H-NMR (300 MHz, CDCl_3_): δ 8.42 (1H, d, J = 8.7 Hz, H6′), 7.97 (1H, dd, J_1_ = 8.7 Hz, J_2_ = 4.5 Hz, H4), 7.56 (1H, dd, J_1_ = 8.1 Hz, J_2_ = 1.8 Hz, H5′), 7.19 (1H, td, J_1_ = 9.1 Hz, J_2_ = 2.1 Hz, H5), 6.67 (1H, dd, J_1_ = 9.0 Hz, J_2_ = 1.8 Hz, H7), 6.57 (1H, brs, H3′), 4.03 (3H, s, OCH_3_), 3.89 (3H, s, OCH_3_); ^13^C-NMR (125 MHz, CDCl_3_) (assignments made with the aid of DEPT-135): δ 163.1 (d, J = 3.3 Hz, C9), 162.9 (C2′), 159.9 (d, J = 242.5 Hz, C6), 158.5 (C4′), 148.8 (C2), 136.6 (d, J = 10.9 Hz, C8), 130.5 (C6′), 123.1 (d, J = 9.5 Hz, C4), 115.4 (C1′), 114.3 (d, J = 24.5 Hz, C5), 107.1 (d, J = 26.3 Hz, C7), 106.0 (C5′), 98.4 (C3′), 55.7 (OCH_3_), 55.6 (OCH_3_); ^19^F-NMR (^1^H-decoupled, 376 MHz, CDCl_3_): δ −120.8 (s, F); ^19^F-NMR (^1^H-coupled, 376 MHz, CDCl_3_): δ −120.8 −120.9 (m, F). ESMS *m/z* [M + H] ^+^ 290. HRMS calcd for C_15_H_13_FNO_2_S: 290.0646. Found: 290.0632.
**2-(2,4-Dimethoxyphenyl)-6-nitrobenzo[d]thiazole (22).** 2,4-Dimethoxy-*N*-(4-nitrophenyl)benzothioamide was wetted with 1.5 mL of acetonitrile. Compound (**22**) was purified using silica gel column chromatography eluting with 10% ethyl acetate in hexane, increasing to 20% of ethyl acetate in hexane, to give a yellow solid (0.045 g, 45%), m.p. = 205–207 °C. IR (cm^−1^): 2925 (ArCH), 2840 (CH_3_), 1613, 1434, 817; ^1^H-NMR (300 MHz, CDCl_3_): δ 8.81 (2H, d, J = 2.1 Hz, H7), 8.51 (1H, d, J = 8.7 Hz, H4), 8.33 (1H, dd, J_1_ = 9.0 Hz, J_2_ = 2.1 Hz, H5), 8.01 (1H, d, J = 9.0 Hz, H6′), 6.70 (1H, dd, J_1_ = 8.9 Hz, J_2_ = 2.3 Hz, H5′), 6.59 (1H, d, J = 2.1 Hz, H3′), 4.01 (3H, s, OCH_3_), 3.92 (3H, s, OCH_3_); ^13^C-NMR (125 MHz, CDCl_3_) (assignments made with the aid of DEPT-135): δ 169.0 (C2), 164.0 (C4′), 159.2 (C9), 156.1 (C2′), 144.0 (C6), 135.8 (C8), 131.1 (C6′), 122.1 (C4), 121.5 (C5), 117.9 (C7), 114.9 (C1′), 106.3 (C5′), 98.3 (C3′), 55.8 (OCH_3_), 55.7 (OCH_3_). ESMS *m/z* [M + H]^+^ 317. HRMS calcd for C_15_H_13_N_2_O_4_S: 317.0591. Found: 317.0584.
**2-(2,4-Dimethoxyphenyl)-6-iodobenzo[d]thiazole (23).** *N*-(4-Iodophenyl)-2,4-dimethoxybenzothioamide was wetted with 1.5 mL of ethanol. Compound (**23**) was purified using silica gel column chromatography eluting with 5% ethyl acetate in hexane to give an off-white solid (0.068 g, 67%), m.p. = 130–131 °C. IR (cm^−1^): 2924 (ArCH), 2847 (CH_3_), 1601, 813, 846; ^1^H-NMR (300 MHz, CDCl_3_): δ 8.36 (2H, d, J = 8.7 Hz, H6′), 8.11 (1H, br s, H7), 7.67 (1H, br d, J = 8.7 Hz, H4), 7.63 (1H, dd, J_1_ = 8.7 Hz, ^4^J_2_ = 1.2 Hz, H5), 6.58 (1H, dd, J_1_ = 8.9 Hz, J_2_ = 2.3 Hz, H5′), 6.48 (1H, d, J = 2.1 Hz, H3′), 3.94 (3H, s, OCH_3_), 3.80 (3H, s, OCH_3_); ^13^C-NMR (125 MHz, CDCl_3_) (assignments made with the aid of DEPT-135): δ 163.7 (C2), 163.2 (C4′), 158.7 (C2′), 151.6 (C9), 137.9 (C8), 134.7 (C7), 130.8 (C5), 129.5 (C6′), 123.8 (C4), 115.1 (C1′), 106.1 (C5′), 98.4 (C3′), 88.1 (C6), 56.7 (OCH_3_), 55.6 (OCH_3_). ESMS *m/z* [M + H] ^+^ 398. HRMS calcd for C_15_H_13_INO_2_S: 397.9706. Found: 397.9694.
**2-(2,5-Dimethoxyphenyl)-6-methoxybenzo[d]thiazole (24).** 2,5-Dimethoxy-*N*-(4-methoxyphenyl)benzothioamide was wetted with 2.5 mL of acetonitrile. Compound (**24**) was purified using silica gel column chromatography eluting with 8% ethyl acetate in hexane to give an off-white solid (0.065 g, 74%), m.p. = 80–83 °C. IR (cm^−1^): 2931 (ArCH), 2836 (CH_3_), 1603, 1505, 995, 830; ^1^H-NMR (300 MHz, CDCl_3_): δ 8.03 (1H, br s, H6′), 7.97 (1H, d, J = 9.0 Hz, H4), 7.37 (1H, d, J = 2.1 Hz, H7), 7.10 (1H, dd, J_1_ = 9.0 Hz, J_2_ = 2.4 Hz, H5), 7.0 (2H, br s, H3′, 4′), 4.0 (3H, s, OCH_3_), 3.90 (3H, s, OCH_3_), 3.89 (3H, s, OCH_3_); ^13^C-NMR (125 MHz, CDCl_3_) (assignments made with the aid of DEPT-135): δ 160.5 (C2), 157.4 (C6), 154.1 (C5′), 151.6 (C2′), 146.9 (C9), 137.6 (C8), 123.4 (C4), 123.0 (C1′), 118.3 (C5), 115.5 (C4′), 113.6 (C3′), 112.4 (C6′), 103.4 (C7), 56.5 (OCH_3_), 56.0 (OCH_3_), 55.8 (OCH_3_). ESMS *m/z* [M + H] ^+^ 302. HRMS calcd for C_16_H_16_INO_3_S: 302.0845. Found: 302.0833.
**6-Bromo-2-(2,5-dimethoxyphenyl)benzo[d]thiazole (25).** *N*-(4-Bromophenyl)-2,5-dimethoxybenzothioamide was wetted with 1.5 mL of ethanol. Compound (**25**) was purified using silica gel column chromatography eluting with 8% ethyl acetate in hexane to give a white solid (0.075 g, 66%), m.p. = 135–137 °C. IR (cm^−1^): 2932 (ArCH), 2830 (CH_3_), 1593, 1499, 803, 730; ^1^H-NMR (300 MHz, CDCl_3_): δ 8.05 (1H, d, J = 2.7 Hz, H6′), 8.03 (1H, d, J = 1.8 Hz, H7), 7.92 (1H, d, J = 8.7 Hz, H4), 7.57 (1H, dd, J_1_ = 8.7 Hz, J_2_ = 1.8 Hz, H5), 7.05 (1H, dd, J_1_ = 9.0 Hz, J_2_ = 2.7 Hz, H4′), 7.00 (1H, d, J = 9.0 Hz, H3′), 4.01 (3H, s, OCH_3_), 3.90 (3H, s, OCH_3_); ^13^C-NMR (125 MHz, CDCl_3_) (assignments made with the aid of DEPT-135): δ 163.4 (C2), 153.9 (C9), 151.9 (C2′), 150.9 (C5′), 138.0 (C8), 129.3 (C5), 123.9 (C7), 123.7 (C4), 122.3 (C1′), 119.2 (C6), 118.0 (C4′), 113.4 (C3′), 112.6 (C6′), 56.4 (OCH_3_), 56.0 (OCH_3_). ESMS *m/z* [M + H]^+^ 349.9, ^79^Br; [M + H]^+^ 351.9, ^81^Br. HRMS calcd for C_15_H_13_BrNO_2_S: 349.9845, 351.9824. Found 349.9841, 351.9819.
**2-(2,5-Dimethoxyphenyl)-6-(trifluoromethyl)benzo[d]thiazole (26).** 2,5-Dimethoxy-*N*-(4-(trifluoromethyl)phenyl)benzothioamide was wetted with 1.5 mL ethanol. Compound (**26**) was purified using silica gel column chromatography eluting with 5% of ethyl acetate in hexane to give a white solid (0.073 g, 64%), m.p. = 140–142 °C. IR (cm^−1^): 2937 (ArCH), 2839 (CH_3_), 1583, 1501, 874; ^1^H-NMR (300 MHz, CDCl_3_): δ 8.21 (1H, br s, H7), 8.15 (1H, d, J = 8.4 Hz, H4), 8.10 (1H, d, J = 2.7 Hz, H6′), 7.71 (1H, d, J = 8.4 Hz, H5), 7.08 (1H, dd, J_1_ = 9.2 Hz, J_2_ = 2.9 Hz, H4′), 7.02 (1H, d, J = 9.0 Hz, H3′), 4.04 (3H, s, OCH_3_), 3.92 (3H, s, OCH_3_); ^13^C-NMR (125 MHz, CDCl_3_) (assignments made with the aid of DEPT-135): δ 165.9 (C2), 153.95 (C9), 153.93 (C5′), 152.1 (C2′), 136.2 (C8), 126.5 (q, ^2^J_CF_ = 32.1 Hz, C6), 124.4 (q, J = 270.4 Hz, CF_3_), 123.0 (C1′), 122.8 (q, J = 3.4 Hz, C5), 122.1 (C4), 119.6 (C4′), 118.9 (q, J = 4.2 Hz, C7), 113.4 (C3′), 112.6 (C6′), 56.4 (OCH_3_), 56.0 (OCH_3_); ^9^F-NMR (^1^H-decoupled, 376 MHz, CDCl_3_): δ −64.4 (s, CF_3_). ESMS *m/z* [M + H]^+^ 340. HRMS calcd for C_16_H_13_F_3_NO_2_S: 340.0621. Found: 340.0600.
**6-Chloro-2-(2,5-dimethoxyphenyl)benzo[d]thiazole (27).** *N*-(4-Chlorophenyl)-2,5-dimethoxybenzothioamide was wetted with 1.5 mL of ethanol. Compound (**27**) was purified using silica gel column chromatography eluting with 5% ethyl acetate in hexane to give a white solid (0.080 g, 65%), m.p. = 220–222 °C. IR (cm^−1^): 2935 (ArCH), 2831 (CH_3_), 1584, 1503, 790; ^1^H-NMR (300 MHz, CDCl_3_): δ 8.06 (1H, br s, H7), 7.98 (1H, br d, J = 8.7 Hz, H4), 7.88 (1H, br s, H6′), 7.43 (1H, br d, J = 8.1 Hz, H5), 7.09–6.96 (2H, m, H3′, 4′), 4.02 (s, 3H, OCH_3_), 3.91 (s, 3H, OCH_3_); ^13^C-NMR (125 MHz, CDCl_3_) (assignments made with the aid of DEPT-135): δ 163.4 (C2), 154.0 (C5′), 151.9 (C9), 150.6 (C2′), 137.5 (C8), 130.4 (C6), 126.7 (C5), 123.5 (C4), 122.4 (C1′), 120.8 (C7), 119.1 (C4′), 113.5 (C3′), 112.6 (C6′), 56.4 (OCH_3_), 56.0 (OCH_3_). ESMS *m/z* [M + H]^+^ 306, ^35^Cl; 308 (M + H]^+^,308, ^37^Cl. HRMS calcd for C_15_H_13_ClNO_2_S: 306.0350, 308.0321. Found: 306.0346, 308.0315.
**2-(2,5-Dimethoxyphenyl)benzo[d]thiazole-6-carbonitrile (28).** *N*-(4-Cyanophenyl)-2,5-dimethoxybenzothioamide was wetted with 3.0 mL of acetonitrile. Compound (**28**) was purified using silica gel column chromatography eluting with 8% ethyl acetate in hexane to give a white solid (0.035 g, 32%), m.p. = 182–184 °C. IR (cm^−1^): 2940 (ArCH), 2836 (CH_3_), 2219 (CN), 1584, 1501, 708; ^1^H-NMR (300 MHz, CDCl_3_): δ 8.24 (1H, br s, H7), 8.11 (1H, d, J = 8.7 Hz, H4), 8.08 (1H, br s, H6′), 7.71 (1H, br d, J = 8.1 Hz, H5), 7.09 (1H, dd, J_1_ = 8.9 Hz, J_2_ = 2.6 Hz, H4′), 7.03 (1H, d, J = 8.7 Hz, H3′), 4.02 (3H, s, OCH_3_), 3.91 (s, 3H, OCH_3_); ^13^C-NMR (125 MHz, CDCl_3_) (assignments made with the aid of DEPT-135): δ 167.1 (C2), 154.4 (C9), 154.0 (C5′), 152.3 (C2′), 136.7 (C8), 129.0 (C5), 126.2 (C7), 123.4 (C4), 121.8 (C1′), 120.1 (C4′), 119.1 (CN), 113.4 (C3′), 112.7 (C6′), 107.7 (C6), 56.4 (OCH_3_), 56.0 (OCH_3_). ESMS *m/z* [M + H]^+^ 297. HRMS calcd for C_16_H_13_N_2_O_2_S: 297.0692. Found: 297.0685.
**2-(2,5-Dimethoxyphenyl)-6-fluorobenzo[d]thiazole (29).** *N*-(4-Fluorophenyl)-2,5-dimethoxybenzothioamide was wetted with 3.0 mL of acetonitrile. Compound (**29**) was purified using silica gel column chromatography eluting with 8% ethyl acetate in hexane to give a white solid (0.073 g, 68%), m.p. = 105–107 °C. IR (cm^−1^): 2922 (ArCH), 2840 (CH_3_), 1601, 1565, 1505, 804, 730; ^1^H-NMR (300 MHz, CDCl_3_): δ 8.05 (1H, d, J = 2.7 Hz, H6′), 8.03 (1H, dd, J_1_ = 9.0 Hz, J_2_ = 4.8 Hz, H4), 7.59 (1H, dd, J_1_ = 8.1 Hz, J_2_ = 2.4 Hz, H7), 7.22 (1H, td, J_1_ = 8.9 Hz, J_2_ = 2.6 Hz, H5), 7.05 (1H, dd, J_1_ = 9.2 Hz, J_2_ = 2.7 Hz, H4′), 7.01 (1H, d, J = 9.3 Hz, H3′), 4.02 (3H, s, OCH_3_), 3.91 (3H, s, OCH_3_); ^13^C-NMR (125 MHz, CDCl_3_) (assignments made with the aid of DEPT-135): δ 162.7 (d, J = 3.4 Hz, C9), 160.2 (d, J = 243.8 Hz, C6), 154.0 (C5′), 151.8 (C2′), 148.7 (C2), 137.3 (d, J = 10.8 Hz, C8), 123.7 (d, J = 9.6 Hz, C4), 122.6 (C1′), 118.9 (C4′), 114.6 (d, J = 24.5 Hz, C5), 113.5 (C3′), 112.5 (C6′), 107.2 (d, J = 26.1 Hz, C7), 56.4 (OCH_3_), 56.0 (OCH_3_); ^19^F-NMR (^1^H-decoupled, 376 MHz, CDCl_3_): δ −120.0 (s, F). ESMS *m/z* [M + H]^+^ 290. HRMS calcd for C_15_H_13_FNO_2_S: 290.0646. Found: 290.0633.
**2-(2,5-Dimethoxyphenyl)-6-nitrobenzo[d]thiazole (30).** 2,5-Dimethoxy-*N*-(4-nitrophenyl)benzothioamide was wetted with 3.0 mL of acetonitrile. Compound (**30**) was purified using silica gel column chromatography eluting with 8% ethyl acetate in hexane to give a yellow solid (0.015 g, 13%), m.p. = 192–193 °C. IR (cm^−1^): 2918 (ArCH), 2833 (CH_3_), 1589, 1565, 1045, 793; ^1^H-NMR (300 MHz, CDCl_3_): δ 8.86 (1H, d, J = 2.1 Hz, H7), 8.36 (1H, dd, J_1_ = 9.0 Hz, J_2_ = 2.1 Hz, H5), 8.13 (1H, d, J = 6.3 Hz, H4), 8.12 (1H, br d, J = 2.7 Hz, H6′), 7.12 (1H, dd, J_1_ = 9.3 Hz, J_2_ = 3.0 Hz, H4′), 7.05 (1H, d, J = 9.0 Hz, H3′), 4.07 (3H, s, OCH_3_), 3.93 (3H, s, OCH_3_); ^13^C-NMR (125 MHz, CDCl_3_) (assignments made with the aid of DEPT-135): δ 168.6 (C2), 155.8 (C9), 153.9 (C5′), 152.4 (C2′), 144.4 (C6), 136.5 (C8), 122.8 (C4), 121.8 (C1′), 121.5 (C5), 120.4 (7), 118.0 (C4′), 113.4 (C3′), 112.6 (C6′), 56.4 (OCH_3_), 56.0 (OCH_3_).
**2-(2,5-Dimethoxyphenyl)-6-iodobenzo[d]thiazole (31).** *N*-(4-Iodophenyl)-2,5-dimethoxybenzothioamide was wetted with 2.0 mL of ethanol. Compound (**31**) was purified using silica gel column chromatography eluting with 5% ethyl acetate in hexane to give a white solid (0.060 g, 56%), m.p. = 130–132 °C. IR (cm^−1^): 2928 (ArCH), 2831 (CH_3_), 1586, 835; ^1^H-NMR (300 MHz, CDCl_3_): δ 8.24 (1H, br d, J = 1.2 Hz, H7), 8.06 (1H, br d, J = 2.4 Hz, H6′), 7.81 (1H, d, J = 8.7 Hz, H4), 7.75 (1H, dd, J_1_ = 8.7 Hz, J_2_ = 1.5 Hz, H5), 7.05 (1H, dd, J_1_ = 9.0 Hz, J_2_ = 2.7 Hz, H4′), 7.0 (1H, d, J = 9.0 Hz, H3′), 4.02 (3H, s, OCH_3_), 3.90 (3H, s, OCH_3_); ^13^C-NMR (125 MHz, CDCl_3_) (assignments made with the aid of DEPT-135): δ 163.3 (C2), 154.0 (C9), 152.0 (C5′), 151.4 (C2′), 138.5 (C8), 134.9 (C7), 129.7 (C5), 124.3 (C4), 122.3 (C1′), 119.2 (C4′), 113.5 (C3′), 112.6 (C6′), 88.8 (C6), 56.4 (OCH_3_), 56.0 (OCH_3_). ESMS *m/z* [M + H]^+^ 398. HRMS calcd for C_15_H_13_INO_2_S: 397.9713. Found: 397.9693.
**2-(3,4-Dimethoxyphenyl)-6-methoxybenzo[d]thiazole (32).** 3,4-Dimethoxy-*N*-(4-methoxyphenyl)benzothioamide was wetted with 3.0 mL of acetonitrile. Compound (**32**) was purified using silica gel column chromatography eluting, initially with 10% ethyl acetate in hexane and increasing to 25% of ethyl acetate in hexane, to give a white solid (0.075 g, 74%), m.p. = 136–138 °C. IR (cm^−1^): 2961 (ArCH), 2844 (CH_3_), 1601, 1458, 803; ^1^H-NMR (300 MHz, CDCl_3_): δ 7.91 (1H, d, J = 9.0 Hz, H4), 7.66 (1H, d, J = 1.8 Hz, H7), 7.53 (1H, dd, J_1_ = 8.3 Hz, J_2_ = 2.0 Hz, H5), 7.33 (1H, br d, J = 2.4 Hz, H2′), 7.07 (1H, dd, J_1_ = 8.9 Hz, J_2_ = 2.4 Hz, H6′), 6.92 (1H, d, J = 8.4 Hz, H5′), 4.01 (3H, s, OCH_3_), 3.94 (3H, s, OCH_3_), 3.88 (s, 3H, OCH_3_); ^13^C-NMR (125 MHz, CDCl_3_) (assignments made with the aid of DEPT-135): δ 165.4 (C2), 157.5 (C6), 151.2 (C3′), 149.3 (C4′), 148.7 (C9), 136.2 (C8), 126.9 (C1′), 123.3 (C4), 120.7 (C6′), 115.3 (C5), 111.0 (C2′), 109.5 (C5′), 104.2 (C7), 56.1 (OCH_3_), 56.0 (OCH_3_), 55.8 (OCH_3_).
**6-Bromo-2-(3,4-dimethoxyphenyl)benzo[d]thiazole (33).** *N*-(4-Bromophenyl)-3,4-dimethoxybenzothioamide was wetted with 2.0 mL of acetonitrile and 0.5 ml of ethanol. Compound (**33**) was purified using silica gel column chromatography eluting with 8% ethyl acetate in hexane to give a white solid (0.074 g, 68%), m.p. = 165–168 °C. IR (cm^−1^): 2934 (ArCH), 2836 (CH_3_), 1588, 1524, 1402, 1250, 794; ^1^H-NMR (300 MHz, CDCl_3_): δ 7.97 (1H, br s, H7), 7.85 (1H, br d, J = 8.4 Hz, H4), 7.66 (1H, br s, H2′), 7.55 (2H, br d, J = 8.1 Hz, H5, 6′), 6.92 (1H, br d, J = 8.1 Hz, H5′), 4.01 (3H, s, OCH_3_), 3.95 (3H, s, OCH_3_); ^13^C-NMR (125 MHz, CDCl_3_) (assignments made with the aid of DEPT-135): δ 168.4 (C2), 153.0 (C9), 151.8 (C3′), 149.4 (C4′), 136.5 (C8), 129.7 (C5), 126.2 (C1′), 124.0 (C7), 123.8 (C4), 121.2 (C6′), 118.3 (C6), 111.0 (C2′), 109.7 (C5′), 56.1 (OCH_3_), 56.0 (OCH_3_).
**2-(3,4-Dimethoxyphenyl)-6-(trifluoromethyl)benzo[d]thiazole (34).** 3,4-Dimethoxy-*N*-(4-(trifluoromethyl)phenyl)benzothioamide was wetted with 2.0 mL of ethanol. Compound (**34**) was purified using silica gel column chromatography eluting initially with 5% ethyl acetate in hexane, increasing to 10% ethyl acetate in hexane, to give a white solid (0.050 g, 46%), m.p. = 164–166 °C. IR (cm^−1^): 2931 (ArCH), 2837 (CH_3_), 1592, 1522, 825, 792; ^1^H-NMR (300 MHz, CDCl_3_): δ 8.16 (1H, br s, H7), 8.10 (1H, br d, J = 8.4 Hz, H4), 7.71 (1H, d, J = 1.2 Hz, H2′), 7.70 (1H, dd, J_1_ = 6.6 Hz, J_2_ = 1.2 Hz, H6′), 7.62 (1H, dd, J = 8.4 Hz, J = 1.8 Hz, H5), 6.96 (1H, br d, J = 8.4 Hz, H5′), 4.03 (3H, s, OCH_3_), 3.97 (3H, s, OCH_3_); ^13^C-NMR (125 MHz, CDCl_3_) (assignments made with the aid of DEPT-135): δ 170.9 (C2), 156.1 (C9), 152.2 (C3′), 149.5 (C4′), 135.1 (C8), 126.9 (q, J = 32.2 Hz, C6), 126.0 (C1′), 124.2 (q, J = 270.8 Hz, CF_3_), 123.2 (q, J = 3.3 Hz, C5), 123.0 (C4), 121.6 (C6′), 119.1 (q, J = 4.1 Hz, C7), 111.1 (C2′), 109.9 (C5′), 56.15 (OCH_3_), 56.08 (OCH_3_); ^19^F-NMR (^1^H-decoupled, 376 MHz, CDCl_3_): δ −64.6 (s, CF_3_). ESMS *m/z* [M + H]^+^ 340. HRMS calcd for C_16_H_13_F_3_NO_2_S: 340.0614. Found: 340.0600.
**6-Chloro-2-(3,4-dimethoxyphenyl)benzo[d]thiazole (35).** *N*-(4-Chlorophenyl)-3,4-dimethoxybenzothioamide was wetted with 2.0 mL of acetonitrile and 0.5 ml of ethanol. Compound (**35**) was purified using silica gel column chromatography eluting, initially with 5% ethyl acetate in hexane and increasing to 10% ethyl acetate in hexane, to give a white solid (0.075 g, 69%), m.p. = 160–162 °C. IR (cm^−1^): 2935 (ArCH), 2837 (CH_3_), 1601, 1526, 793; ^1^H-NMR (300 MHz, CDCl_3_): δ 7.92 (1H, br d, J = 8.7 Hz, H4), 7.83 (1H, d, J = 1.8 Hz, H7), 7.67 (1H, d, J = 1.8 Hz, H2′), 7.57 (1H, dd, J_1_ = 8.4 Hz, J_2_ = 1.8 Hz, H5), 7.42 (1H, dd, J_1_ = 8.7 Hz, J_2_ = 2.1 Hz, H6′), 6.93 (1H, br d, J = 8.4 Hz, H5′), 4.01 (3H, s, OCH_3_), 3.95 (3H, s, OCH_3_); ^13^C-NMR (125 MHz, CDCl_3_) (assignments made with the aid of DEPT-135): δ 168.4 (C2), 152.7 (C9), 151.8 (C3′), 149.4 (C4′), 136.1 (C8), 130.7 (C6), 127.0 (C5), 126.2 (C1′), 123.5 (C4), 121.2 (C7), 121.1 (C6′), 111.0 (C2′), 109.7 (C5′), 56.1 (OCH_3_), 56.0 (OCH_3_).
**2-(3,4-Dimethoxyphenyl)benzo[d]thiazole-6-carbonitrile (36).** *N*-(4-Cyanophenyl)-3,4-dimethoxybenzothioamide was wetted with 2.0 mL of acetonitrile. Compound (**36**) was purified using silica gel column chromatography eluting, initially with 10% ethyl acetate in hexane and increasing to 20% of ethyl acetate in hexane, to give a white solid (0.018 g, 18%), m.p. = 186–189 °C. IR (cm^−1^): 2942 (ArCH), 2839 (CH_3_), 2222 (CN), 1584, 825; ^1^H-NMR (400 MHz, CDCl_3_): δ 8.20 (1H, d, J = 1.6 Hz, H7), 8.08 (1H, d, J = 8.4 Hz, H4), 7.73 (1H, dd, J_1_ = 8.4 Hz, J_2_ = 1.6 Hz, H5), 7.72 (1H, d, J = 2.0 Hz, H2′), 7.64 (1H, dd, J_1_ = 8.4 Hz, J_2_ = 2.0 Hz H6′), 6.97 (1H, d, ^2^J = 8.4 Hz, H5′), 4.03 (3H, s, OCH_3_), 3.98 (3H, s, OCH_3_); ^13^C-NMR (125 MHz, CDCl_3_) (assignments made with the aid of DEPT-135): δ 172.1 (C2), 156.6 (C9), 152.5 (C3′), 149.5 (C4′), 135.4 (C8), 129.5 (C5), 126.2 (C7), 125.7 (C1′), 123.4 (C6′), 121.8 (C4), 118.8 (CN), 111.1 (C2′), 109.9 (C5′), 108.1 (C6), 56.2 (OCH_3_), 56.1 (OCH_3_). ESMS *m/z* [M + H] ^+^ 297. HRMS calcd for C_16_H_13_N_2_O_2_S: 297.0692. Found: 297.0678.
**2-(3,4-Dimethoxyphenyl)-6-fluorobenzo[d]thiazole (37)** [28]. *N*-(4-Fluorophenyl)-3,4-dimethoxybenzothioamide was wetted with 2.0 mL of ethanol. Compound (**37**) was purified using silica gel column chromatography eluting with 8% ethyl acetate in hexane to give a white solid (0.070 g, 67%), m.p. = 152–154 °C, lit (153–155 °C) [29]. IR (cm^−1^): 2917 (ArCH), 2867 (CH_3_), 1602, 1553, 864; ^1^H-NMR (300 MHz, CDCl_3_): δ 7.96 (1H, dd, J_1_ = 8.9 Hz, J_2_ = 5.0 Hz, H4), 7.66 (1H, d, J = 1.8 Hz, H2′), 7.57–7.50 (2H, m, H6′, 7), 7.19 (1H, td, J_1_ = 8.9 Hz, J_2_ = 2.6 Hz, H5), 6.93 (1H, br d, J = 8.4 Hz, H5′), 4.01 (3H, s, OCH_3_), 3.95 (3H, s, OCH_3_); ^13^C-NMR (125 MHz, CDCl_3_) (assignments made with the aid of DEPT-135): δ 167.6 (d, J = 3.3 Hz, C9), 160.3 (d, J = 243.8 Hz, C6), 151.6 (C2), 150.8 (C3′), 149.4 (C4′), 135.8 (d, J = 10.9 Hz, C8), 126.4 (C1′), 123.6 (d, J = 9.1 Hz, C4), 121.0 (C6′), 114.8 (d, J = 24.3 Hz, C5), 111.0 (C2′), 109.6 (C5′), 107.7 (d, ^2^J_CF_ = 26.9 Hz, C7), 56.1 (OCH_3_), 56.0 (OCH_3_); ^19^F-NMR (^1^H-decoupled, 376 MHz, CDCl_3_): δ −119.6 (s,); ^19^F-NMR (^1^H-coupled, 376 MHz, CDCl_3_): δ −119.55 −119.68 (m, F).
**2-(3,4-Dimethoxyphenyl)-6-iodobenzo[d]thiazole (38).** *N*-(4-Iodophenyl)-3,4-dimethoxybenzothioamide was wetted with 2.0 mL of ethanol. Compound (**38**) was purified using silica gel column chromatography eluting with 8% ethyl acetate in hexane to give a white solid (0.040 g, 45%). IR (cm^−1^): 2937 (ArCH), 2834 (CH_3_), 1601, 1523, 805; ^1^H-NMR (300 MHz, CDCl_3_): δ 8.19 (1H, br s, H7), 7.74 (2H, br s, H4, 6′), 7.68 (1H, d, J = 1.8 Hz, H2′), 7.57 (1H, dd, J_1_ = 9.0 Hz, J_2_ = 1.8 Hz, H5), 6.94 (1H, d, J = 8.4 Hz, H5′), 4.00 (3H, s, OCH_3_), 3.96 (3H, s, OCH_3_); ^13^C-NMR (125 MHz, CDCl_3_) (assignments made with the aid of DEPT-135): δ 168.3 (C2), 153.5 (C9), 151.8 (C3′), 149.4 (C4′), 137.0 (C8), 135.3 (C5), 129.9 (C7), 126.1 (C1′), 124.2 (C4), 121.2 (C6′), 111.0 (C2′), 109.7 (C5′), 88.9 (C6), 56.12 (OCH_3_), 56.06 (OCH_3_). ESMS *m/z* [M + H]^+^ 398. HRMS calcd for C_15_H_13_INO_2_S: 397.9706. Found: 397.9690.
**6-Methoxy-2-(3,4,5-trimethoxyphenyl)benzo[d]thiazole (40).** 3,4,5-Trimethoxy-*N*-(4-methoxyphenyl)benzothioamide was wetted with 0.5 mL of ethanol. Compound (**40**) was purified using silica gel column chromatography eluting, initially with 10% ethyl acetate in hexane and increasing to 15% ethyl acetate in hexane, to give an off-white solid (0.090 g, 90%), m.p. = 113–114 °C. IR (cm^−1^): 2929 (ArCH), 2835 (CH_3_), 1582, 827; ^1^H-NMR (300 MHz, CDCl_3_): δ 7.94 (1H, d, J = 9.0 Hz, H4), 7.35 (1H, d, J = 2.4 Hz, H7), 7.28 (2H, br s, H2′, 6′), 7.10 (1H, dd, J = 9.0 Hz, J = 2.4 Hz, H5), 3.99 (6H, s, 2 × OCH_3_), 3.92 (3H, s, OCH_3_), 3.90 (3H, s, OCH_3_); ^13^C-NMR (125 MHz, CDCl_3_) (assignments made with the aid of DEPT-135): δ 165.3 (C2), 157.7 (C6), 153.6 (C3′, 5′), 148.6 (C9), 140.3 (C4′), 136.4 (C8), 129.2 (C1′), 123.6 (C4), 115.6 (C5), 104.4 (C2′, 6′), 104.2 (C7), 61.0 (OCH_3_), 56.3 (2 × OCH_3_), 55.8 (OCH_3_). ESMS *m/z* [M + H]^+^ 332. HRMS calcd for C_17_H_18_NO_4_S: 332.0951. Found: 332.0945. HPLC > 95% pure.
**6-Bromo-2-(3,4,5-trimethoxyphenyl)benzo[d]thiazole (41).** *N*-(4-Bromophenyl)-3,4,5-trimethoxybenzothioamide was wetted with 2.0 mL of ethanol. Compound (**41**) was purified using silica gel column chromatography eluting with 8% ethyl acetate in hexane to give a white solid (0.073 g, 69%), m.p. = 171–172 °C. IR (cm^−1^): 2938 (ArCH), 2834 (CH_3_), 1597, 1485, 995; ^1^H-NMR (300 MHz, CDCl_3_): δ 8.02 (1H, d, J = 1.8 Hz, H7), 7.89 (1H, d, J = 8.7 Hz, H4), 7.59 (1H, dd, J_1_ = 8.7 Hz, J_2_ = 1.8 Hz, H5), 7.30 (2H, br s, H2′, 6′), 3.98 (6H, s, 2 × OCH_3_), 3.93 (3H, s, OCH_3_); ^13^C-NMR (125 MHz, CDCl_3_) (assignments made with the aid of DEPT-135): δ 168.3 (C2), 153.6 (C3′, 5′), 153.0 (C9), 141.0 (C4′), 136.7 (C8), 129.8 (C5), 128.6 (C1′), 124.13 (C7), 124.07 (C4), 118.6 (C6), 104.8 (C2′, 6′), 61.0 (OCH_3_), 56.4 (2 × OCH_3_). ESMS *m/z* [M + H]^+^ 379.9, ^79^Br; [M + H] 382, ^81^Br. HRMS calcd for C_16_H_15_BrNO_3_S: 379.9951, 381.9930. Found 379.9948, 381.9927.
**6-(Trifluoromethyl)-2-(3,4,5-trimethoxyphenyl)benzo[d]thiazole (42).** 3,4,5-Trimethoxy-*N*-(4-(trifluoromethyl)phenyl)benzothioamide was wetted with 2.0 mL of ethanol. Compound (**42**) was purified using silica gel column chromatography eluting with 5% ethyl acetate in hexane to give a white solid (0.055 g, 54%), m.p. = 171–172 °C. IR (cm^−1^): 2929 (ArCH), 2838 (CH_3_), 1589, 1487, 824; ^1^H-NMR (300 MHz, CDCl_3_): δ 8.18 (1H, br s, H7), 8.12 (1H, d, J = 8.7 Hz, H4), 7.72 (1H, br d, J = 8.1 Hz, H5), 7.34 (2H, br s, H2′, 6′), 4.00 (6H, s, 2 × OCH_3_), 3.94 (3H, s, OCH_3_); ^13^C-NMR (125 MHz, CDCl_3_) (assignments made with the aid of DEPT-135): δ 170.8 (C2), 156.0 (C9), 153.7 (C3′, 5′), 141.3 (C4′), 135.1 (C8), 128.4 (C1′), 127.2 (q, J = 32.5 Hz, C6), 124.2 (q, J = 270.7 Hz, CF_3_), 123.4 (q, J = 4.0 Hz, C5), 123.3 (C4), 119.2 (q, J = 3.3 Hz, C7), 105.0 (C2′, 6′), 61.1 (OCH_3_), 56.4 (2 × OCH_3_); ^9^F-NMR (^1^H-decoupled, 376 MHz, CDCl_3_): δ −64.4 (s, CF_3_). ESMS *m/z* [M + H]^+^ 370. HRMS calcd for C_17_H_15_F_3_NO_3_S: 370.0719. Found: 370.0704.
**6-Chloro-2-(3,4,5-trimethoxyphenyl)benzo[d]thiazole (43).** *N*-(4-Chlorophenyl)-3,4,5-trimethoxybenzothioamide was wetted with 2.0 mL of ethanol. Compound (**43**) was purified using silica gel column chromatography eluting with 8% ethyl acetate in hexane to give an off-white solid (0.070 g, 64%), m.p. = 173–174 °C. IR (cm^−1^): 2940 (ArCH), 2835 (CH_3_), 1586, 1488, 817; ^1^H-NMR (300 MHz, CDCl_3_): δ 7.94 (1H, d, J = 8.7 Hz, H4), 7.85 (1H, d, J = 1.8 Hz, H7), 7.44 (1H, dd, J_1_ = 8.7 Hz, J_2_ = 2.1 Hz, H5), 7.29 (2H, br s, H2′, 6′), 3.98 (6H, s, 2 × OCH_3_), 3.92 (3H, s, OCH_3_); ^13^C-NMR (125 MHz, CDCl_3_) (assignments made with the aid of DEPT-135): δ 168.2 (C2), 153.6 (C3′, 5′), 152.6 (C9), 140.9 (C4′), 136.2 (C8), 131.0 (C6), 128.6 (C1′), 127.1 (C5), 123.8 (C4), 121.1 (C7), 104.8 (C2′, 6′), 61.0 (OCH_3_), 56.4 (2 × OCH_3_).
**2-(3,4,5-Trimethoxyphenyl)benzo[d]thiazole-6-carbonitrile (44).** *N*-(4-Cyanophenyl)-3,4,5-trimethoxybenzothioamide was wetted with 0.5 mL of ethanol. Compound (**44**) was purified using silica gel column chromatography eluting with 15% ethyl acetate in hexane to give a white solid (0.015 g, 15%), m.p. = 228–230 °C. IR (cm^−1^): 2923 (ArCH), 2853 (CH_3_), 2222 (CN), 1586, 1485, 819; ^1^H-NMR (300 MHz, CDCl_3_): δ 8.22 (1H, br s, H7), 8.10 (1H, d, J = 8.7 Hz, H4), 7.74 (1H, dd, J_1_ = 8.4 Hz, J_2_ = 1.2 Hz, H5), 7.34 (2H, br s, H2′, 6′), 3.99 (6H, s, 2 × OCH_3_), 3.94 (3H, s, OCH_3_); ^13^C-NMR (125 MHz, CDCl_3_) (assignments made with the aid of DEPT-135): δ 172.0 (C2), 156.4 (C9), 153.7 (C3′, 5′), 141.5 (C4′), 135.5 (C8), 129.6 (C5), 128.0 (C1′), 126.3 (C7), 123.6 (C4), 118.8 (CN), 108.3 (C6), 105.0 (C2′, 6′), 61.1 (OCH_3_), 56.4 (2 × OCH_3_). ESMS *m/z* [M + H]^+^ 327. HRMS calcd for C_17_H_15_F_3_N_2_O_3_S: 327.0798. Found: 327.0784.
**6-Fluoro-2-(3,4,5-trimethoxyphenyl)benzo[d]thiazole (45).** *N*-(4-Fluorophenyl)-3,4,5-trimethoxybenzothioamide was wetted with 2.0 mL of ethanol. Compound (**45**) was purified using silica gel column chromatography eluting with 8% ethyl acetate in hexane to give a white solid (0.065 g, 60%), m.p. = 156–158 °C. IR (cm^−1^): 2938 (ArCH), 2839 (CH_3_), 1587, 1360, 1218, 810; ^1^H-NMR (300 MHz, CDCl_3_): δ 8.00 (1H, dd, J_1_ = 8.7 Hz, J_2_ = 4.8 Hz, H4), 7.57 (1H, dd, J_1_ = 8.0 Hz, J_2_ = 2.3 Hz, H7), 7.29 (2H, br s, H2′, 6′), 7.23 (1H, td, J_1_ = 8.7 Hz, J_2_ = 2.4 Hz, H5), 3.99 (6H, s, 2 × OCH_3_), 3.93 (3H, s, OCH_3_); ^13^C-NMR (125 MHz, CDCl_3_) (assignments made with the aid of DEPT-135): δ 167.5 (d, J = 3.0 Hz, C9), 160.4 (d, J = 245.0 Hz, C6), 153.7 (C3′, 5′), 150.7 (d, J = 2.5 Hz, C2), 140.8 (C4′), 136.0 (d, J = 11.3 Hz, C8), 128.8, C1′), 124.0 (d, J = 9.4 Hz, C4), 114.9 (d, J = 24.5 Hz, C5), 107.8 (d, J = 26.9 Hz, C7), 104.7 (C2′, 6′), 61.0 (OCH_3_), 56.4 (2 × OCH_3_); ^19^F-NMR (^1^H-decoupled, 376 MHz, CDCl_3_): δ −119.1 (s, F); ^19^F-NMR (^1^H-coupled, 376 MHz, CDCl_3_): δ −119.12 –119.05 (m, F).
**6-Iodo-2-(3,4,5-trimethoxyphenyl)benzo[d]thiazole (46).** *N*-(4-Iodophenyl)-3,4,5-trimethoxybenzo-thioamide was wetted with 0.5 mL of ethanol. Compound (**46**) was purified using silica gel column chromatography eluting, initially with 2% ethyl acetate in hexane and increasing to 10% of ethyl acetate in hexane, to give a white solid (0.03 g, 25%), m.p. = 153–155 °C. IR (cm^−1^): 2933 (ArCH), 2832 (CH_3_), 1587, 1484, 995, 816; ^1^H-NMR (300 MHz, CDCl_3_): δ 8.21 (1H, brs, H7), 7.77 (2H, br s, H4, 5), 7.30 (2H, br s, H2′, 6′), 3.98 (6H, s, 2 × OCH_3_), 3.93 (3H, s, OCH_3_); ^13^C-NMR (125 MHz, CDCl_3_) (assignments made with the aid of DEPT-135): δ 168.2 (C2), 153.6 (C3′, 5′), 153.4 (C9), 140.9 (C4′), 137.1 (C8), 135.5 (C5), 130.0 (C7), 128.5 (C1′), 124.5 (C4), 104.7 (C2′, 6′), 89.3 (C6), 61.0 (OCH_3_), 56.4 (2 × OCH_3_). ESMS *m/z* [M + H]^+^ 428. HRMS calcd for C_16_H_15_INO_3_S: 427.9812. Found: 427.9795. HPLC > 95% pure.
**6-Fluoro-2-(3-methoxyphenyl)benzo[d]thiazole (53).** *N*-(4-Fluorophenyl)-3-methoxybenzothioamide was wetted with 0.5 mL of ethanol. Compound (**53**) was purified using silica gel column chromatography eluting with 5% ethyl acetate in hexane to give a white solid (0.180 g, 91%), m.p. = 103–105 °C. IR (cm^−1^): 2920 (ArCH), 2840 (CH_3_), 1603, 916; ^1^H-NMR (300 MHz, CDCl_3_): δ 8.02 (1H, dd, J_1_ = 9.0 Hz, J_2_ = 4.8 Hz, H4), 7.65–7.55 (3H, m, H7, 2′, 6′), 7.40 (1H, t, J = 8.0 Hz, H5′), 7.23 (1H, td, J_1_= 8.9 Hz, J_2_ = 2.8 Hz, H5), 7.05 (1H, dd, J_1_ = 8.4 Hz, J_2_ = 1.8 Hz, H4′), 3.92 (3H, s, OCH_3_); ^13^C-NMR (125 MHz, CDCl_3_) (assignments made with the aid of DEPT-135): δ 167.6 (d, J = 3.0 Hz, C9), 160.5 (d, J = 244.3 Hz, C6), 160.0 (C3′), 150.6 (C2), 136.0 (d, J = 11.0 Hz, C8), 134.6 (C1′), 130.1 (C5′), 124.1 (d, J = 9.3 Hz, C4), 120.1 (C6′), 117.3 (C4′), 114.9 (d, J = 24.4 Hz, C5), 111.9 (2′), 107.8 (d, J = 26.4 Hz, C7), 55.5 (OCH_3_); ^19^F-NMR (^1^H-decoupled, 376 MHz, CDCl_3_): δ −118.9 (s, F).
**6-Fluoro-2-(4-methoxyphenyl)benzo[d]thiazole (54).** *N*-(4-Fluorophenyl)-4-methoxybenzothioamide was wetted with 1.5 mL of acetonitrile. Compound (**54**) was purified using silica gel column chromatography eluting with 5% ethyl acetate in hexane to give an off-white solid (0.275 g, 93%), m.p. = 127–130 °C. IR (cm^−1^): 3009 (ArCH), 2839 (CH_3_), 1603, 816; ^1^H-NMR (300 MHz, CDCl_3_): δ 7.98 (2H, br d, J_1_ = 8.7 Hz, H2′, 6′), 7.94 (1H, dd, J_1_ = 9.0 Hz, J_2_ = 4.8 Hz, H4), 7.54 (1H, dd, J_1_ = 8.1 Hz, J_2_ = 2.7 Hz, H7), 7.19 (1H, td, ^3^J_HH_ = ^3^J_HF_ = 8.9 Hz, J_2_ = 2.6 Hz, H5), 6.99 (2H, d, J = 9.0 Hz, H3′, 5′), 3.87 (3H, s, OCH_3_); ^13^C-NMR (125 MHz, CDCl_3_) (assignments made with the aid of DEPT-135): δ 167.6 (d,J = 2.9 Hz, C2), 161.9 (C4′), 160.2 (d,J = 243.3 Hz, C6), 150.8 (C9), 135.8 (d, J = 11.5 Hz, C8), 129.0 (C2′, 6′), 126.1 (C1′), 123.6 (d, J = 9.1 Hz, C4), 114.7 (d, J = 24.4 Hz, C5), 114.4 (3′, 5′), 107.7 (d, J = 26.9 Hz, C7), 55.4 (OCH_3_); ^19^F-NMR (^1^H-decoupled, 376 MHz, CDCl_3_): −119.6 (s, F).
**6-Fluoro-2-(3-fluorophenyl)benzo[d]thiazole (55).** Fluoro-*N*-(4-fluorophenyl)benzothioamide was wetted with 0.5 mL of ethanol. Compound (**55**) was purified using silica gel column chromatography eluting with 5% of ethyl acetate in hexane to give a yellow solid (0.375 g, 85%), m.p. = 111–112 °C. IR (cm^−1^): 1603, 1589, 995, 701; ^1^H-NMR (300 MHz, CDCl_3_): δ 7.93 (1H, dd, J_1_ = 9.0 Hz, J = 4.8 Hz, H4), 7.74–7.67 (2H, m, H2′, 6′), 7.50 (1H, dd, J_1_ = 8.1 Hz, J = 2.7 Hz, H7), 7.37 (1H, td, J_1_ = 7.9 Hz, J_2_ = 5.7 Hz, H5′), 7.15 (1H, td, J_1_ = 8.9 Hz, J_2_ = 2.9 Hz, H5), 7.14–7.06 (1H, m, H4′); ^13^C-NMR (125 MHz, CDCl_3_) (assignments made with the aid of DEPT-135): δ 166.1 (dd ~ t, J = 3.3 Hz, C2), 163.0 (d, J = 245.9 Hz, C3′), 160.6 (d, J = 245.0 Hz, C6), 150.6 (d, J = 1.4 Hz, C9), 136.1 (d, J = 10.9 Hz, C8), 135.4 (d, J = 7.9 Hz, C1′), 130.7 (d, J = 7.9 Hz, C5′), 124.4 (d, J = 9.6 Hz, C4), 123.2 (d, J = 2.9 Hz, C6′), 117.9 (d, J = 21.1 Hz, C2′), 115.2 (d, J = 24.9 Hz, C4′), 114.2 (d, J = 23.4 Hz, C5), 107.9 (d, J = 26.4 Hz, C7); ^19^F-NMR (^1^H-decoupled, 376 MHz, CDCl_3_): δ -108.7 (s, F), − 115.7 (s, F).
**6-Fluoro-2-(4-fluorophenyl)benzo[d]thiazole (56).** 4-Fluoro-*N*-(4-fluorophenyl)benzothioamide was wetted with 0.5 mL of ethanol. Compound (**56**) was purified using silica gel column chromatography eluting with 5% ethyl acetate in hexane to give a pale-yellow solid (0.180 g, 91%), m.p. = 138–140 °C. IR (cm^−1^): 2924 (ArCH), 2924 (CH_3_), 1598, 974, 833; ^1^H-NMR (300 MHz, CDCl_3_): δ 8.06 (2H, dd, J_1_ = 8.7 Hz, J_2_ = 5.1 Hz, H2′, 6′), 8.0 (1H, dd, J_1_ = 8.9 Hz, J_2_ = 5.0 Hz, H4), 7.58 (1H, dd, J_1_ = 8.1 Hz, J_2_ = 2.4 Hz, H7), 7.254–7.215 (1H, m, H5), 7.14 (2H, t, J = 8.7 Hz, H3′, 5′); ^13^C-NMR (125 MHz, CDCl_3_) (assignments made with the aid of DEPT-135): δ 166.5 (d, J = 3.3 Hz, C9), 164.5 (d, J = 250.8 Hz, C4′), 160.5 (d, J = 244.6 Hz, C6), 150.7 (C2), 136.0 (d, J = 11.3 Hz, C8), 129.7 (d, J = 3.1 Hz, C1′), 129.4 (d, J = 8.6 Hz, C2′, 6′), 124.1 (d, J = 9.5 Hz, C4), 116.2 (d, J = 21.9 Hz, C3′, 5′), 115.1 (d, J = 24.4 Hz, C5), 107.9 (d, J = 27.0 Hz, C7); ^19^F-NMR (^1^H-decoupled, 376 MHz, CDCl_3_): δ −111.8 (s, F), − 118.8 (s, F).
**2-(3,5-Difluorophenyl)-6-methoxybenzo[d]thiazole (57).** 3,5-Difluoro-*N*-(4-methoxyphenyl)benzothioamide was wetted with 1.0 mL of ethanol. Compound (**57**) was purified using silica gel column chromatography eluting with 5% ethyl acetate in hexane to give a white solid (0.170 g, 80%), m.p. =149–151 °C. IR (cm^−1^): 2923 (ArCH), 2844 (CH_3_), 1592, 1509, 984, 846; ^1^H-NMR (300 MHz, CDCl_3_): δ 7.96 (1H, br d, J = 9.0 Hz, H4), 7.59–7.55 (2H, m, H2′, 6′), 7.36 (1H, d, J = 2.4 Hz, H7), 7.13 (1H, dd, J_1_ = 9.0 Hz, J_2_ = 2.7 Hz, H5), 6.90 (1H, tt, J_1_ = 8.7 Hz, J_2_ = 2.3 Hz, H4′), 3.90 (3H, s, OCH_3_); ^13^C-NMR (125 MHz, CDCl_3_) (assignments made with the aid of DEPT-135): δ 163.3 (dd, J_1_ = 247.9 Hz, J_2_ = 12.6 Hz, C3′, 5′), 162.4 (t, J = 3.6 Hz, C2), 158.3 (C6), 148.4 (C9), 136.7 (t, J = 10.0 Hz, C1′), 136.6 (C8), 124.2 (C4), 116.2 (C5), 110.1 (dd, J_1_ = 20.5 Hz, J_2_ = 6.8 Hz, C2′, 6′), 105.5 (t, J = 25.4 Hz, C4′), 104.0 (C7), 55.8 (OCH_3_); ^19^F-NMR (^1^H-decoupled, 376 MHz, CDCl_3_): δ −111.8 (s, 2 × F). ESMS *m/z* [M + H] ^+^ 278. HRMS calcd for C_14_H_10_F_2_NOS: 278.0446. Found: 278.0436.
**2-(3,5-Difluorophenyl)-6-fluorobenzo[d]thiazole (58)**. 3,5-Difluoro-*N*-(4-fluorophenyl)benzothioamide was wetted with 3.0 mL of ethanol. Compound (**58**) was purified using silica gel column chromatography eluting with 3% ethyl acetate in hexane to give a white solid (0.095 g, 66%), m.p. = 140–142 °C. IR (cm^−1^): 2962 (ArCH), 2852 (CH_3_), 1592, 846; ^1^H-NMR (300 MHz, CDCl_3_): δ 8.04 (1H, dd, J_1_ = 9.0 Hz, J_2_ = 4.8 Hz, H4), 7.63–7.58 (3H, m, H7, 2′, 6′), 7.26 (1H, td, J_1_ = 8.9 Hz, J_2_ = 2.6 Hz, H5), 6.94 (1H, tt, J_1_ = 8.6 Hz, J_2_ = 2.3 Hz, H4′); ^13^C-NMR (125 MHz, CDCl_3_) (assignments made with the aid of DEPT-135): δ 164.7 (dt ~ q, J = 3.5 Hz, C2), 163.3 (dd, J_1_ = 248.6 Hz, J_2_ = 12.4 Hz, C3′, 5′), 160.8 (d, J = 248.5 Hz, C6), 150.5 (d, J = 1.3 Hz, C9), 136.2 (t, J = 10.3 Hz, C1′), 136.1 (d, J = 11.5 Hz, C8), 124.7 (d, J = 9.4 Hz, C4), 115.5 (d, J = 24.5 Hz, C5), 110.3 (dd, ^2^J_CF_ = 20.6 Hz, ^4^J_CF_ = 6.9 Hz, C2′, 6′), 107.9 (d, J = 27.0 Hz, C7), 106.1 (t, J = 25.3 Hz, C4′); ^19^F-NMR (^1^H-decoupled, 376 MHz, CDCl_3_): δ -111.4 (s, 2F), -117.7 (s, F); ^19^F-NMR (^1^H-coupled, 376 MHz, CDCl_3_): δ −111.4 (t, J = 7.3 Hz, 2F), − 117.7 (td, J_1_ = 8.3 Hz, J_2_ = 4.9 Hz, F). ESMS *m/z* [M + H]^+^ 266. HRMS calcd for C_13_H_7_F_3_NS: 266.0246. Found: 266.0236.

#### 3.1.1. General Procedure for Synthesis of Phenylbenzo[d]thiazoles **10**, **39** and **47**

Phenylbenzothioamides (*N*-(2-Chloro-4-nitrophenyl)-3,5-dimethoxybenzothioamide, *N*-(2-Chloro-4-nitrophenyl)-3,4-dimethoxybenzothioamide, and *N*-(2-Chloro-4-nitrophenyl)-3,4,5-trimethoxybenzothioamide) (1.0 mmol) and 2.0 mmol of sodium hydride in dry DMSO (3.0 mL) were heated in a microwave at 150 °C for 10 min. The reaction mixture was cooled down and quenched with water. The precipitated solid was filtered and washed with water. The solid was dissolved in DCM. The DCM layer was dried with dry MgSO_4_ and purified using column chromatography; the details of purification are specified under each compound.

**2-(3,5-Dimethoxyphenyl)-6-nitrobenzo[d]thiazole (10).** Purified using silica gel column chromatography eluting, initially with 10% ethyl acetate in hexane and increasing to 15% ethyl acetate in hexane, to give a pale brown solid (0.110 g, 82%), m.p. = 199–200 °C. IR (cm^−1^): 2923 (ArCH), 2851 (CH_3_), 1590, 1505, 753; ^1^H-NMR (300 MHz, CDCl_3_): δ 8.83 (1H, d, J = 2.1 Hz, H7), 8.37 (1H, dd, J_1_ = 8.9 Hz, J_2_ = 2.3 Hz, H5), 8.13 (1H, d, J = 9.0 Hz, H4), 7.26 (2H, d, J = 1.8 Hz, H2′, 6′), 6.65 (1H, t, J = 2.1 Hz, H4′), 3.91 (6H, s, 2 × OCH_3_); ^13^C-NMR (125 MHz, CDCl_3_) (assignments made with the aid of DEPT-135): δ 173.7 (C2), 161.3 (C3′, 5′), 157.7 (C9), 144.9 (C6), 135.3 (C8), 134.4 (C1′), 123.3 (C4), 121.9 (C5), 118.2 (C7), 105.8 (C2′, 6′), 104.4 (C4′), 55.7 (2 × OCH_3_). ESMS (ES) *m/z* [M + H] ^+^ 317. ESMS *m/z* [M + H]^+^ 317. HRMS calcd for C_15_H_13_N_2_O_4_S: 317.0591. Found: 317.0580.
**2-(3,4-Dimethoxyphenyl)-6-nitrobenzo[d]thiazole (39).** Obtained as a yellow solid (0.07 g, 54%), m.p. = 221–222 °C. IR (cm^−1^): 2922 (ArCH), 2848 (CH_3_), 1596, 1510, 750; ^1^H-NMR (300 MHz, CDCl_3_): δ 8.81 (1H, d, J = 2.1 Hz, H7), 8.36 (1H, dd, ^3^J = 9.0 Hz, ^4^J = 2.4 Hz, H5), 8.08 (1H, d,J = 9.0 Hz, H4), 7.73 (1H, d, J = 1.8 Hz, H2′), 7.66 (1H, dd, J_1_ = 8.4 Hz, J_2_ = 2.1 Hz, H6′), 6.98 (1H, d, J = 8.1 Hz, H5′), 4.04 (s, 3H, OCH_3_), 3.99 (s, 3H, OCH_3_); ^13^C-NMR (125 MHz, CDCl_3_) (assignments made with the aid of DEPT-135): δ 173.6 (C2), 158.0 (C9), 152.6 (C3′), 149.5 (C4′), 144.6 (C6), 135.2 (C8), 125.7 (C1′), 122.8 (C4), 122.0 (C5), 121.9 (C6′), 118.1 (C7), 111.1 (C2′), 109.9 (C5′), 56.2 (OCH_3_), 56.1 (OCH_3_).
**6-Nitro-2-(3,4,5-trimethoxyphenyl)benzo[d]thiazole (47).** Purified using silica gel column chromatography eluting with 15% ethyl acetate in hexane to give a yellow solid (0.103 g, 67%), m.p. = 200–201 °C. IR (cm^−1^): 3938 (ArCH), 2836 (CH_3_), 1588, 1516, 750; ^1^H-NMR (300 MHz, CDCl_3_): δ 8.82 (1H, d, J = 2.1 Hz, H7), 8.37 (1H, dd, J_1_ = 9.0 Hz, J_2_ = 2.1 Hz, H5), 8.11 (1H, d, J = 8.7 Hz, H4), 7.36 (2H, br s, H2′, 6′), 4.00 (6H, s, 2 × OCH_3_), 3.95 (3H, s, OCH_3_); ^13^C-NMR (125 MHz, CDCl_3_) (assignments made with the aid of DEPT-135): δ 173.5 (C2), 157.8 (C9), 153.7 (C3′, 5′), 144.8 (C6), 141.8 (C4′), 135.3 (C8), 128.0 (C1′), 123.1 (C4), 122.0 (C5), 118.1 (C7), 105.2 (C2′, 6′), 61.1 (OCH_3_), 56.4 (2 × OCH_3_ ).

#### 3.1.2. General Procedures for Synthesis of Aminobenzothiazoles (**11**) and (**48**) [28]

Stannous chloride (5.0 mmol) was added to (**10**) or (**47**) (1.0 mmol) in methanol (6.0 mL), THF (2.0 mL), and concentrated HCl (0.1 mL) [1]. The reaction mixture was heated at 65 °C for 4 h (compound **10**) or for 10 h (compound **47**). Consumption of the starting material was monitored using TLC (30% ethyl acetate in hexane). The reaction mixture was concentrated, DCM was added, and the mixture was stirred for 15 min. The base was added (NaOH (2.0 M) to pH 12.0. The reaction mixture was extracted with DCM and the organic layer was dried with dry MgSO_4_ and evaporated to give a desired compound.

**2-(3,5-Dimethoxyphenyl)benzo[d]thiazol-6-amine (11).** Obtained as a yellow solid (0.108 g, 80%), m.p. = 155–153 °C. IR (cm^−1^): 3448 (NH), 3319 (NH), 2923 (ArCH), 2853 (CH_3_), 1591, 811; ^1^H-NMR (400 MHz, DMSO-d_6_): δ 7.71 (1H, d, J = 8.8 Hz, H4), 7.09 (1H, d, J = 2.4 Hz, H7), 7.07 (2H, d, J = 2.4 Hz, H2′, 6′), 6.80 (1H, dd, J_1_ = 8.6 Hz, J_2_ = 2.2 Hz, H5), 6.63 (1H, t, J_1_ = 2.4 Hz, H4′), 5.53 (2H, br s, NH_2_), 3.83 (6H, s, 2 × OCH_3_); ^13^C-NMR (125 MHz, DMSO-d_6_) (assignments made with the aid of DEPT-135): δ 160.9 (C3′, 5′), 160.1 (C2), 147.6 (C6), 145.0 (C9), 136.4 (C8), 135.3 (C1′), 123.3 (C4), 115.2 (5), 104.2 (C2′, 6′), 103.6 (C7), 102.2 (C4′), 55.4 (2 × OCH_3_). ESMS (ES) *m/z* [M + H]^+^ 287. HRMS calcd for C_15_H_15_N_2_O_2_S: 287.0849. Found: 287.0852.
**2-(3,4,5-Trimethoxyphenyl)benzo[d]thiazol-6-amine (48).** Obtained as a yellow solid (0.110 g, 80%), m.p = 212–215 °C. IR (cm^−1^): 3409 (NH), 3329 (NH), 2939 (ArCH), 2831 (CH_3_), 1584, 820; ^1^H-NMR (400 MHz, DMSO-d_6_): δ 7.69 (1H, d, J = 8.8 Hz, H4), 7.21 (2H, br s, H2′, 6′), 7.09 (1H, d, J = 2.4 Hz, H7), 6.79 (1H, dd, J_1_ = 8.8 Hz, J_2_ = 2.0 Hz, H5), 5.94 (2H, br s, NH_2_), 3.89 (6H, s, 2 × OCH_3_), 3.73 (3H, s, OCH_3_); ^13^C-NMR (125 MHz, DMSO-d_6_) (assignments made with the aid of DEPT-135): δ 160.3 (C2), 153.3 (C3′, 5′), 147.4 (C6), 145.1 (C9), 139.2 (C4′), 136.4 (C8), 129.0 (C1′), 123.1 (C4), 115.1 (C5), 103.7 (C7), 103.6 (C2′, 6′), 60.2 (OCH_3_), 56.0 (2 × OCH_3_). ESMS *m/z* [M + H]^+^ 317. HRMS calcd for C_16_H_17_N_2_O_3_S: 317.0954. Found: 317.0941. HPLC > 95% pure.

#### 3.1.3. General Procedures for Synthesis of Benzothiazole Amides **12**–**14** and **49**–**51** [22]

Acetic acid, benzoic acid or 3,5-dimethoxybenzoic acid (1.3 mmol), 1.0 mmol of amine **11** or **48,** and PCl_3_ (1.0 mmol) in dry acetonitrile (3.0 mL) were heated in a microwave at 150 °C for 5 min.

Workup for compounds (**12**, **50**, **51**): Acetonitrile was removed through evaporation. The remaining solid residue was washed with water and NaOH (2.0 M) and extracted with DCM. The organic layer was dried with dry MgSO_4_ and evaporated.

Workup for compounds (**13**, **14**, **49**): Acetonitrile was removed through evaporation. The remaining solid residue was washed with water and NaOH (2.0 M) and extracted with DCM. The organic layer was then evaporated and dried.

***N*-(2-(3,5-Dimethoxyphenyl)benzo[d]thiazol-6-yl)acetamide (12).** Yellow solid (0.08 g, 87%), m.p. = 179–181 °C. IR (cm^−1^): 3318 (NH), 2939 (ArCH), 2838 (CH_3_), 1670 (C=O), 1594, 832; ^1^H-NMR (400 MHz, CDCl_3_): δ 8.45 (1H, d, J = 1.6 Hz, H7), 7.95 (1H, d, J = 8.8 Hz, H4), 7.61 (1H, br s, NH), 7.29 (1H, dd, J_1_ = 8.8 Hz, J_2_ = 1.6 Hz, H5), 7.20 (2H, d, J = 2.4 Hz, H2′, 6′), 6.57 (1H, t, J = 1.6 Hz, H4′), 3.87 (6H, s, 2 × OCH_3_), 2.22 (3H, s, CH_3_); ^13^C-NMR (125 MHz, CDCl_3_) (assignments made with the aid of DEPT-135): δ 168.4 (C=O), 167.4 (C2), 161.1 (C3′, 5′), 150.6 (C9), 136.1 (C6), 135.34 (C8), 135.30 (C1′), 123.2 (C4), 118.9 (C5), 112.4 (C7), 105.3 (C2′, 6′), 103.4 (C4′), 55.6 (2 × OCH_3_), 24.7 (CH_3_). ESMS *m/z* [M + H]^+^ 329. HRMS calcd for C_17_H_17_N_2_O_3_S: 329.0954. Found: 329.0940.
***N*-(2-(3,5-Dimethoxyphenyl)benzo[d]thiazol-6-yl)benzamide (13).** Yellow solid (0.085 g, 78%), m.p. = 138–140 °C. IR (cm^−1^): 3312 (NH), 2925 (ArCH), 2847 (CH_3_), 1642 (C=O), 1591, 706; ^1^H-NMR (400 MHz, DMSO-d_6_): δ 10.58 (1H, br s, NH), 8.68 (1H, d, J = 2.0 Hz, H7), 8.05 (1H, d, J = 9.2 Hz, H4), 8.00 (2H, br d, J = 7.6 Hz, H2″, 6″), 7.85 (1H, dd, J_1_ = 8.8 Hz, J_2_ = 1.6 Hz, H5), 7.62 (1H, t, J = 7.4 Hz, H4″), 7.56 (2H, t, J = 7.2 Hz, H3″, 5″), 7.18 (2H, d, J = 2.0 Hz, H2′, 6′), 6.71 (1H, t, J = 2.2 Hz, H4′), 3.86 (6H, s, 2 × OCH_3_); ^13^C-NMR (125 MHz, DMSO-d_6_) (assignments made with the aid of DEPT-135): δ 165.8 (C2, C=O), 161.0 (C3′, 5′), 149.7 (C9), 137.1 (C8), 135.0 (C1′), 134.8 (C1″), 131.7 (C6), 129.2 (C4″), 128.4 (C3″, 5″), 127.7 (C2″, 6″), 122.9 (C4), 120.3 (C5), 112.6 (C7), 104.8 (C2′, 6′), 103.1 (C4′), 55.6 (2 × OCH_3_). ESMS *m/z* [M + H] ^+^ 391. HRMS calcd for C_22_H_19_N_2_O_3_S: 391.1111. Found: 391.1094.
***N*-(2-(3,5-Dimethoxyphenyl)benzo[d]thiazol-6-yl)-3,5-dimethoxybenzamide (14).** Pale-yellow solid (0.085 g, 68%), m.p. = 159–162 °C. IR (cm^−1^): 3318 (NH), 2939 (ArCH), 2838 (CH_3_), 1670 (C=O), 1594, 832; ^1^H-NMR (300 MHz, CDCl_3_): δ 8.60 (1H, d, J = 1.8 Hz, H7), 8.02 (1H, d, J = 9.0 Hz, H4), 7.96 (1H, br s, NH), 7.43 (1H, dd, J_1_ = 9.0 Hz, J_2_ = 2.1 Hz, H5), 7.23 (2H, d, J = 2.1 Hz, H2′, 6′), 7.00 (2H, d, J = 2.1 Hz, H2″, 6″), 6.64 (1H, t, J = 2.1 Hz, H4′), 6.59 (1H, t, J = 2.1 Hz, H4″), 3.89 (6H, s, 2 × OCH_3_), 3.86 (6H, s, 2 × OCH_3_); ^13^C-NMR (125 MHz, CDCl_3_) (assignments made with the aid of DEPT-135): δ 167.6 (C2), 165.6 (C=O), 161.13 (C3″, 5″), 161.12 (C3′, 5′), 150.9 (C9), 137.0 (C8), 136.2 (C6), 135.3 (C1, 1″), 123.3 (C4), 119.3 (C5), 112.6 (C7), 105.3 (C2″, 6″), 105.0 (C2′, 6′), 103.9 (C4″), 103.5 (C4′), 55.7 (OCH_3_), 55.6 (2 × OCH_3_). ESMS *m/z* [M + H]^+^ 451. HRMS calcd for C_24_H_23_N_2_O_5_: 451.1322. Found: 451.1322.
***N*-(2-(3,4,5-Trimethoxyphenyl)benzo[d]thiazol-6-yl)acetamide (49).** Yellow solid (0.085 g, 94%), m.p. = 208–209 °C. IR (cm^−1^): 3186 (NH), 2940 (ArCH), 2838 (CH_3_), 1679 (C=O), 1584, 808; ^1^H-NMR (400 MHz, DMSO-d_6_): δ 10.30 (1H, br s, NH), 8.50 (1H, d, J = 1.6 Hz, H7), 7.97 (1H, d, J = 8.8 Hz, H4), 7.57 (1H, dd, J_1_ = 9.0 Hz, J_2_ = 1.8 Hz, H5), 7.29 (2H, br s, H2′, 6′), 3.91 (6H, s, 2 × OCH_3_), 3.74 (3H, s, OCH_3_), 2.10 (s, 3H, CH_3_); ^13^C-NMR (125 MHz, DMSO-d_6_) (assignments made with the aid of DEPT-135): δ 168.5 (C=O), 165.4 (C2), 153.4 (C3′, 5′), 149.3 (C9), 140.0 (C4′), 137.0 (C6), 135.2 (C8), 128.4 (C1′), 122.7 (C4), 118.9 (C5), 111.1 (C7), 104.2 (C2′, 6′), 60.2 (OCH_3_), 56.1 (2 × OCH_3_), 24.0 (CH_3_). ESMS *m/z* [M + H]^+^ 359. HRMS calcd for C_18_H_19_N_2_O_4_S: 359.1060. Found: 359.1044. HPLC > 95% pure.
***N*-(2-(3,4,5-Trimethoxyphenyl)benzo[d]thiazol-6-yl)benzamide (50).** Yellow solid (0.1 g, 94%), m.p. = 205- 207 °C. IR (cm^−1^): 3309 (NH), 2935 (ArCH), 2836 (CH_3_), 1646 (C=O), 1582, 822; ^1^H-NMR (400 MHz, DMSO-d_6_): δ 10.56 (1H, br s, NH), 8.67 (1H, d, J = 2.0 Hz, H7), 8.04 (1H, d, J = 8.8 Hz, H4), 8.00 (2H, br d, J = 7.2 Hz, H2″, 6″), 7.84 (1H, dd, J_1_ = 8.8 Hz, J_2_ = 2.0 Hz, H5), 7.62 (1H, t, J = 7.2 Hz, H4″), 7.56 (2H, t, J = 7.2 Hz, H3″, 5″), 7.33 (2H, br s, H2′, 6′), 3.92 (6H, s, 2 × OCH_3_), 3.75 (s, 3H, OCH_3_); ^13^C-NMR (125 MHz, DMSO-d_6_) (assignments made with the aid of DEPT-135): δ 165.9 (C2), 165.7 (C=O), 153.4 (C3′, 5′), 149.8 (C9), 140.0 (C4′), 136.8 (C8), 135.1 (C6), 134.8 (C1″), 131.7 (C4″), 128.4 (C3″, 5″), 127.7 (C2″, 6″), 122.6 (C4), 120.2 (C5), 112.6 (C7), 104.2 (C2′, 6′), 60.2 (OCH_3_), 56.1 (2 × OCH_3_). ESMS (ES) *m/z* [M + H]^+^ 421. HRMS calcd for C_23_H_21_N_2_O_4_S: 421.1217. Found: 421.1197.
**3,5-Dimethoxy-*N*-(2-(3, 4, 5-trimethoxyphenyl)benzo[d]thiazol-6-yl)benzamide (51).** Yellow solid (0.110 g, 91%), m.p. = 185–187 °C. IR (cm^−1^): 3265 (NH), 2931 (ArCH), 2837 (CH_3_), 1646 (C=O), 1583, 997; ^1^H-NMR (400 MHz, CDCl_3_): δ 8.56 (1H, d, J = 2.0 Hz, H7), 8.24 (1H, br s, NH), 7.95 (1H, d, J = 8.8 Hz, H4), 7.43 (1H, dd, J_1_ = 8.8 Hz, J_2_ = 2.0 Hz, H5), 7.27 (2H, br s, H2′, 6′), 6.99 (2H, d, J = 2.4 Hz, H2″, 6″), 6.59 (1H, t, ^4^J = 2.2 Hz, H4″), 3.96 (6H, s, 2 × OCH_3_), 3.91 (3H, s, OCH_3_), 3.81 (6H, s, 2 × OCH_3_); ^13^C-NMR (125 MHz, CDCl_3_) (assignments made with the aid of DEPT-135): δ 167.4 (C2), 165.7 (C=O), 161.0 (C3″, 5″), 153.5 (C3′, 5′), 150.9 (C9), 140.5 (C4′), 136.9 (C8), 136.0 (C6), 135.2 (C1′), 128.9 (C1″), 123.0 (C4), 119.4 (C5), 112.7 (C7), 105.5 (C2″, 6″), 104.5 (C2′, 6′), 103.8 (C4″), 61.0 (OCH_3_), 56.3 (2 × OCH_3_), 55.6 (2 × OCH_3_). ESMS (ES) *m/z* [M + H]^+^ 481. HRMS calcd for C_25_H_25_N_2_O_6_S: 481.1428. Found: 481.1408.

#### 3.1.4. General Procedures for Synthesis of Hydroxybenzothiazoles (**15**) and (**52**) [21]

Boron tribromide (12.0 mmol) for (**15**) and 20.0 mmol for (**52)** was added dropwise to the (**3**) solution or the (**40)** solution (1.0 mmol) in dry DCM (10.0 mL) at -78 °C. After one hour, the reaction mixture was stirred overnight at room temperature. Consumption of the starting material was identified with TLC (30% ethyl acetate in hexane). Details regarding the workup and purification are specified for each compound.

**6-Hydroxy-2-(3,5-dihydroxyphenyl)benzo[d]thiazole (15)** [21]. The reaction mixture was quenched with water and extracted with ethyl acetate, the organic layer was dried with dry MgSO_4_, and compound (**15**) was purified using silica gel column chromatography eluting with 4% MeOH in DCM to give a white solid (0.075 g, 44%), m.p > 300 °C. IR (cm^−1^): 3465–3023 (3 × OH), 2960 (ArCH), 2895 (CH_3_), 1596, 832; ^1^H-NMR (300 MHz, acetone-d_6_): δ 8.74 (1H, br s, OH), 8.53 (2H, br s, 2 × OH), 7.76 (1H, d, J = 8.7 Hz, H4), 7.37 (1H, d, J = 2.4 Hz, H7), 7.01 (2H, d, J = 2.1 Hz, H2′, 6′), 6.99 (1H, dd, J_1_ = 9.0 Hz, J_2_ = 2.1 Hz, H5), 6.44 (1H, t, J = 2.3 Hz, H4′); ^13^C-NMR (125 MHz, acetone-d_6_) (assignments made with the aid of DEPT-135): δ 165.1 (C2), 160.0 (C3′, 5′), 156.7 (C6), 148.9 (C9), 137.3 (C8), 136.6 (C1′), 124.6 (C4), 116.9 (C5), 107.5 (C7), 106.4 (C2′, 6′), 105.8 (C4′). HPLC > 95% pure.
**6-Hydroxy-2-(3,4,5-trihydroxyphenyl)benzo[d]thiazole (52).** The reaction mixture was quenched with water and filtered. The filtrate was extracted with DCM and the organic layer was dried with dry MgSO_4_ and evaporated to give a yellow solid (**52**) (0.050 g, 20%), m.p. = 240–242 °C. IR (cm^−1^): 3625–3000 (4 x OH), 2923 (ArCH), 1590, 1204; ^1^H-NMR (400 MHz, methanol-d4): δ 7.73 (1H, d, J = 8.8 Hz, H4), 7.28 (1H, d, J = 2.0 Hz, H7), 7.04 (2H, br s, H2′, 6′), 6.98 (1H, dd, J_1_ = 8.8 Hz, ^4^J_2_ = 2.4 Hz, H5); ^13^C-NMR (125 MHz, methanol-d4) (assignments made with the aid of DEPT-135): δ 168.3 (C2), 157.1 (C6), 147.5 (C3′, 5′), 147.4 (C9), 138.2 (C8), 136.7 (C1′), 124.9 (C4′), 124.8 (C4) 123.2 (C5), 117.3 (C7), 107.8 (C2′, 6′). ESMS (ES) *m/z* [M + H]^+^ 276. HRMS calcd for C_13_H_10_NO_4_S: 276.0325. Found: 276.0313. HPLC > 95% pure.

**See Supplementary Data Sections S1–S9.**

### 3.2. Inhibition of Recombinant Human NQO2 Enzyme Assay

The NQO2 enzyme assay was carried out using a MULTISKAN GO spectrophotometer [30] by measuring the absorbance of DCPIP at 600 nm. NRH [31] or EPR were used as co-substrates. The experiment was performed using a 96-well plate with a 200 mL final volume using phosphate buffer (50 mM, pH 7.4). The final concentrations were DCPIP 40 µM and NRH or EPR 200 µM. The final enzyme concentration was 100 ng. The assay was performed at 37 °C and initiated through the addition of NQO2. The initial rates of the enzyme reactions were calculated from the curve by taking the initial slopes of the reactions using the formula (ΔAb/ΔTime). The assay of inhibitors was carried out using the same assay conditions. Resveratrol was used as a positive control, and six different concentrations of inhibitors were used. Each experiment was performed in triplicate. Rates were converted to the percentage of enzyme activity, and IC_50_ values were calculated using GraphPad Prism 7.03 software using the non-linear regression function. IC_50_ values were determined as a 50% decrease in NQO2 activity.

### 3.3. Computational Methods

**Ligand and protein preparation.** The SMILES of the small molecules were created using ChemDraw. Subsequently, Omega was used to generate the 3D structures of conformers in OEBinary format [32]. OEDOCKING 4.3.1.0 MakeReceptor, also an OpenEye tool, was used to prepare the NQO2 protein for molecular docking [33]. A box enclosing the binding site for the target (NQO2 protein) and the bound ligand (resveratrol) was generated, encompassing the entire active region within the box. Shape control was used to specify the shape of the active site, where the shape potential was kept to the default setting (Balanced) and the inner counter was disabled.

**Validation of the docking method.** The docking process was firstly validated through re-docking of resveratrol to the binding site of NQO2 (PDB ID: 1SG0). To compare the conformations of the docked poses and the crystal ligand, the RMSD (root mean square deviation) was calculated using the open-source DockRMSD tool [34].

Docking was performed using FRED 3.4.0.2, which uses an exhaustive systematic search algorithm [23]. ChemGauss4 was used to score the docked poses. For visualisation, Vida 4.4.0 and MOE 2022.02 were used to analyse the docked poses [35,36].

See also Supplementary Data Section S2.

## 4. Conclusions

In this study, we report the design, synthesis, docking, and biological testing of benzothiazoles against the NQO2 enzyme. Among the changes made to the resveratrol structure to make a more stable drug moiety by replacing the metabolically susceptible stilbene bond with a thiazole ring to form benzothiazole, the sizes of the benzothiazoles were comparable to resveratrol, and this makes them fit into the NQO2 binding cleft in a manner similar to resveratrol and resulted in higher calculated binding affinities. Benzothiazoles were synthesised via Jacobsen cyclisation, starting with benzoic acid derivatives and aniline derivatives. Benzothiazoles show variation in the experimental binding affinities and in their interaction, with the most potent inhibitor of NQO2 found here being the hydroxy 3,5-dimethoxybenzothiazole **15** with an IC_50_ value of 25 nM. Several 3,4,5-trimethoxybenzothiazoles inhibited NQO2 at nano-molar concentrations, with the most active being an acetylamide analogue with an IC_50_ value of 31 nM. These potent benzothiazole inhibitors of NQO2 could be of benefit for a range of therapeutic areas, including Alzheimer’s disease [37] and Parkinson’s disease [38].

## Figures and Tables

**Table 1:** Structures of benzothiazoles and IC_50_ values for their inhibition of NQO2.

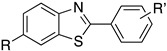			
**Compound**	**R**	**R′**	**IC_50_ (µM) ± SD**
**3**	MeO	3,5-(MeO)_2_	0.108 ± 0.012
**4**	Br	3,5-(MeO)_2_	>100
**5**	CF_3_	3,5-(MeO)_2_	0.123 ± 0.029
**6**	Cl	3,5-(MeO)_2_	82.93 ± 0.04
**7**	CN	3,5-(MeO)_2_	>100
**8**	F	3,5-(MeO)_2_	>100
**9**	I	3,5-(MeO)_2_	2.46 ± 0.01
**10**	NO_2_	3,5-(MeO)_2_	93.44 ± 0.02
**11**	NH_2_	3,5-(MeO)_2_	0.863 ± 0.07
**12**	NHCOMe	3,5-(MeO)_2_	0.605 ± 0.06
**13**	NHCOC_6_H_5_	3,5-(MeO)_2_	8.44 ± 0.03
**14**	NHCO [3,5-(MeO)_2_C_6_H_3_]	3,5-(MeO)_2_	1.36 ± 0.06
**15**	OH	3,5-(OH)_2_	0.025 ± 0.003
**16**	MeO	2,4-(MeO)_2_	5.86 ± 0.07
**17**	Br	2,4-(MeO)_2_	56.90 ± 0.02
**18**	CF_3_	2,4-(MeO)_2_	4.52 ± 0.04
**19**	Cl	2,4-(MeO)_2_	33.55 ± 0.02
**20**	CN	2,4-(MeO)_2_	>100
**21**	F	2,4-(MeO)_2_	3.51 ± 0.08
**22**	I	2,4-(MeO)_2_	1.19 ± 0.05
**23**	NO_2_	2,4-(MeO)_2_	>100
**24**	MeO	2,5-(MeO)_2_	0.846 ± 0.02
**25**	Br	2,5-(MeO)_2_	6.71 ± 0.02
**26**	CF_3_	2,5-(MeO)_2_	2.77 ± 0.01
**27**	Cl	2,5-(MeO)_2_	1.18 ± 0.002
**28**	CN	2,5-(MeO)_2_	>100
**29**	F	2,5-(MeO)_2_	2.06 ± 0.01
**30**	I	2,5-(MeO)_2_	8.54 ± 0.05
**31**	NO_2_	2,5-(MeO)_2_	>100
**32**	MeO	3,4-(MeO)_2_	1.27 ± 0.03
**33**	Br	3,4-(MeO)_2_	9.09 ± 0.04
**34**	CF_3_	3,4-(MeO)_2_	>100
**35**	Cl	3,4-(MeO)_2_	91.67 ± 0.08
**36**	CN	3,4-(MeO)_2_	>100
**37**	F	3,4-(MeO)_2_	5.93 ± 0.05
**38**	I	3,4-(MeO)_2_	22.94 ± 0.02
**39**	NO_2_	3,4-(MeO)_2_	63.28 ± 0.02
**40**	MeO	3,4,5-(MeO)_3_	0.051 ± 0.020
**41**	Br	3,4,5-(MeO)_3_	0.193 ± 0.003
**42**	CF_3_	3,4,5-(MeO)_3_	18.36 ± 0.030
**43**	Cl	3,4,5-(MeO)_3_	0.773 ± 0.016
**44**	CN	3,4,5-(MeO)_3_	13.28 ± 0.02
**45**	F	3,4,5-(MeO)_3_	0.717 ± 0.015
**46**	I	3,4,5-(MeO)_3_	0.106 ± 0.004
**47**	NO_2_	3,4,5-(MeO)_3_	0.754 ± 0.008
**48**	NH_2_	3,4,5-(MeO)_3_	0.079 ± 0.017
**49**	-NHCOMe	3,4,5-(MeO)_3_	0.031 ± 0.008
**50**	-NHCOPh	3,4,5-(MeO)_3_	3.17 ± 0.031
**51**	-NHCO[3,5-(MeO)_2_C_6_H_3_]	3,4,5-(MeO)_3_	3.11 ± 0.030
**52**	OH	3,4,5-(OH)_3_	0.20 ± 0.01
**53**	F	3-MeO	0.908 ± 0.02
**54**	F	4-MeO	>100
**55**	F	3-F	52.55 ± 0.06
**56**	F	4-F	>100
**57**	MeO	3,5-DiF	11.78 ± 0.02
**58**	F	3,5-DiF	>100

## Data Availability

Synthesis and docking data are provided in the article and/or the accompanying Supplementary Materials.

## Supplementary Materials

The supporting information can be downloaded at https://www.mdpi.com/article/10.3390/ijms252212025/s1. References [39,40] are cited in the Supplementary Materials.
