# Supplementary material for: Discovery of Potent Benzothiazole Inhibitors of Oxidoreductase NQO2, a Target for Inflammation and Cancer
Source: Int J Mol Sci. 2024 Nov 8;25(22):12025. doi: 10.3390/ijms252212025 (PMC11593696; doi:10.3390/ijms252212025)
Supplement: Supplementary file 1 [file ijms-25-12025-s001.zip › ijms-3254902-supplementary.pdf]

SUPPLEMENTARY DATA: INTERNATIONAL JOURNAL OF MOLECULAR SCIENCES

Discovery of Potent Benzothiazole Inhibitors of Oxidoreductase NQO2, a Target for Inflammation and Cancer

Asma A. Belgath, Aya M. Emam, Joshua Taujanskas, Richard A. Bryce, Sally Freeman, Ian J. Stratford

TABLE OF CONTENTS

1. Chemicals and materials……………………………………………………………**8**

2. General procedure for the synthesis of amides A [1] **8**

2.1. 3,5-Dimethoxy-*N*-(4-methoxyphenyl)benzamide [1] 9

2.2. *N*-(4-Fluorophenyl)-3,5-dimethoxybenzamide 9

2.3. *N*-(4-Iodophenyl)-3,5-dimethoxybenzamide 9

2.4. 3,5-Difluoro-*N*-(4-methoxyphenyl)benzamide 10

3. General procedure for synthesis of amides B **10**

3.1. 3,5-Dimethoxy-*N*-(4-(trifluoromethyl)phenyl)benzamide 10

3.2. 3,5-Dimethoxy-*N*-(4-nitrophenyl)benzamide 11

3.3. *N*-(4-Cyanophenyl)-3,5-dimethoxybenzamide 11

3.4. *N*-(4-Chlorophenyl)-3,5-dimethoxybenzamide 11

3.5. *N*-(4-Bromophenyl)-3,5-dimethoxybenzamide 12

3.6. 3,5-Difluoro-*N*-(4-fluorophenyl)benzamide 12

3.7. 2,4-Dimethoxy-*N*-(4-methoxyphenyl)benzamide 12

3.8. *N*-(4-Fluorophenyl)-2,4-dimethoxybenzamide 13

3.9. *N*-(4-Iodophenyl)-2,4-dimethoxybenzamide 13

3.10. *N*-(4-Bromophenyl)-2,4-dimethoxybenzamide 13

3.11. 2,4-Dimethoxy-*N*-(4-(trifluoromethyl)phenyl)benzamide 14

3.12. *N*-(4-Chlorophenyl)-2,4-dimethoxybenzamide 14

3.13. *N*-(4-Cyanophenyl)-2,4-dimethoxybenzamide 14

3.14. 2,4-Dimethoxy-*N*-(4-nitrophenyl)benzamide 15

3.15. 2,5-Dimethoxy-*N*-(4-methoxyphenyl)benzamide 15

3.16. *N*-(4-Fluorophenyl)-2,5-dimethoxybenzamide 15

3.17. *N*-(4-Iodophenyl)-2,5-dimethoxybenzamide....16
3.18. *N*-(4-Bromophenyl)-2,5-dimethoxybenzamide....16
3.19. 2,5-Dimethoxy-N-(4-(trifluoromethyl)phenyl)benzamide....16
3.20. *N*-(4-Chlorophenyl)-2,5-dimethoxybenzamide....17
3.21. *N*-(4-Cyanophenyl)-2,5-dimethoxybenzamide....17
3.22. 2,5-Dimethoxy-*N*-(4-nitrophenyl)benzamide....17
3.23. 3,4-Dimethoxy-*N*-(4-methoxyphenyl)benzamide....18
3.24. *N*-(4-Fluorophenyl)-3,4-dimethoxybenzamide....18
3.25. *N*-(4-Iodophenyl)-3,4-dimethoxybenzamide....18
3.26. *N*-(4-Bromophenyl)-3,4-dimethoxybenzamide....19
3.27. 3,4-Dimethoxy-*N*-(4-(trifluoromethyl)phenyl)benzamide....19
3.28. *N*-(4-Chlorophenyl)-3,4-dimethoxybenzamide....19
3.29. *N*-(4-Cyanophenyl)-3,4-dimethoxybenzamide....20
3.30. 3,4-Dimethoxy-*N*-(4-nitrophenyl)benzamide....20
3.31. 3,4,5-Trimethoxy-*N*-(4-methoxyphenyl)benzamide....20
3.32. *N*-(4-Fluorophenyl)-3,4,5-trimethoxybenzamide....20
3.33. *N*-(4-Iodophenyl)-3,4,5-trimethoxybenzamide....21
3.34. *N*-(4-Bromophenyl)-3,4,5-trimethoxybenzamide....21
3.35. 3,4,5-Trimethoxy-*N*-(4-(trifluoromethyl)phenyl)benzamide....21
3.36. *N*-(4-Chlorophenyl)-3,4,5-trimethoxybenzamide....22
3.37. *N*-(4-Cyanophenyl)-3,4,5-trimethoxybenzamide....22
3.38. 3,4,5-Trimethoxy-*N*-(4-nitrophenyl)benzamide....22
4. General procedure for the synthesis of amides C....**23**
4.1. *N*-(2-Chloro-4-nitrophenyl)-3,5-dimethoxybenzamide....23
4.2. *N*-(2-Chloro-4-nitrophenyl)-3,4-dimethoxybenzamide....23
4.3. *N*-(2-chloro-4-nitrophenyl)-3,4,5-trimethoxybenzamide....23
4.4. *N*-(4-Fluorophenyl)-3-methoxybenzamide....24
4.5. *N*-(4-Fluorophenyl)-4-methoxybenzamide....24
4.6. 3-Fluoro-*N*-(4-fluorophenyl)benzamide....24
4.7. 4-Fluoro-*N*-(4-fluorophenyl)benzamide....24
5. General procedure for synthesis of phenylbenzothioamide....**25**
5.1. 3,5-Dimethoxy-*N*-(4-methoxyphenyl)benzothioamide [1]....25
5.2. *N*-(4-Fluorophenyl)-3,5-dimethoxybenzothioamide....25
5.3. *N*-(4-Iodophenyl)-3,5-dimethoxybenzothioamide....26

5.4. *N*-(4-Bromophenyl)-3,5-dimethoxybenzothioamide....26
5.5. 3,5-Dimethoxy-*N*-(4-(trifluoromethyl)phenyl)benzothioamide....26
5.6. *N*-(4-Chlorophenyl)-3,5-dimethoxybenzothioamide....26
5.7. *N*-(4-Cyanophenyl)-3,5-dimethoxybenzothioamide....27
5.8. 3,5-Dimethoxy-*N*-(4-nitrophenyl)benzothioamide....27
5.9. 2,4-Dimethoxy-*N*-(4-methoxyphenyl)benzothioamide....27
5.10. *N*-(4-Fluorophenyl)-2,4-dimethoxybenzothioamide....27
5.11. *N*-(4-Iodophenyl)-2,4-dimethoxybenzothioamide....28
5.12. *N*-(4-Bromophenyl)-2,4-dimethoxybenzothioamide....28
5.13. 2,4-Dimethoxy-*N*-(4-(trifluoromethyl)phenyl)benzothioamide....28
5.14. *N*-(4-Chlorophenyl)-2,4-dimethoxybenzothioamide....29
5.15. *N*-(4-Cyanophenyl)-2,4-dimethoxybenzothioamide....29
5.16. 2,4-Dimethoxy-*N*-(4-nitrophenyl)benzothioamide....29
5.17. 2,5-Dimethoxy-*N*-(4-methoxyphenyl)benzothioamide....30
5.18. *N*-(4-Fluorophenyl)-2,5-dimethoxybenzothioamide....30
5.19. *N*-(4-Iodophenyl)-2,5-dimethoxybenzothioamide....30
5.20. *N*-(4-Bromophenyl)-2,5-dimethoxybenzothioamide....31
5.21. 2,5-Dimethoxy-*N*-(4-(trifluoromethyl)phenyl)benzothioamide....31
5.22. *N*-(4-Chlorophenyl)-2,5-dimethoxybenzothioamide....31
5.23. *N*-(4-Cyanophenyl)-2,5-dimethoxybenzothioamide....32
5.24. 2,5-Dimethoxy-*N*-(4-nitrophenyl)benzothioamide....32
5.25. 3,4-Dimethoxy-*N*-(4-methoxyphenyl)benzothioamide....32
5.26. *N*-(4-Fluorophenyl)-3,4-dimethoxybenzothioamide....32
5.27. *N*-(4-Iodophenyl)-3, 4-dimethoxybenzothioamide....33
5.28. *N*-(4-Bromophenyl)-3,4-dimethoxybenzothioamide....33
5.29. 3,4-Dimethoxy-*N*-(4-(trifluoromethyl)phenyl)benzothioamide....33
5.30. *N*-(4-Chlorophenyl)-3,4-dimethoxybenzothioamide....34
5.31. *N*-(4-Cyanophenyl)-3,4-dimethoxybenzothioamide....34
5.32. 3,4-Dimethoxy-*N*-(4-nitrophenyl)benzothioamide....34
5.33. 3,4,5-Trimethoxy-*N*-(4-methoxyphenyl)benzothioamide....34
5.34. *N*-(4-Fluorophenyl)-3,4,5-trimethoxybenzothioamide....35
5.35. *N*-(4-Iodophenyl)-3,4,5-trimethoxybenzothioamide....35
5.36. *N*-(4-Bromophenyl)-3,4,5-trimethoxybenzothioamide....35
5.37. 3,4,5-Trimethoxy-*N*-(4-(trifluoromethyl)phenyl)benzothioamide....36

5.38. *N*-(4-Chlorophenyl)-3,4,5-trimethoxybenzothioamide 36
5.39. *N*-(4-Cyanophenyl)-3,4,5-trimethoxybenzothioamide 36
5.40. 3,4,5-Trimethoxy-*N*-(4-nitrophenyl)benzothioamide 36
5.41. 3,5-Difluoro-*N*-(4-methoxyphenyl)benzothioamide 37
5.42. 3,5-Difluoro-*N*-(4-fluorophenyl)benzothioamide 37
5.43. *N*-(4-Fluorophenyl)-3-methoxybenzothioamide 37
5.44. *N*-(4-Fluorophenyl)-4-methoxybenzothioamide 38
5.45. 3-Fluoro-*N*-(4-fluorophenyl)benzothioamide 38
5.46. 4-Fluoro-*N*-(4-fluorophenyl)benzothioamide 39
5.47. *N*-(2-Chloro-4-nitrophenyl)-3,5-dimethoxybenzothioamide 39
5.48. *N*-(2-Chloro-4-nitrophenyl)-3,4-dimethoxybenzothioamide 39
5.49. *N*-(2-Chloro-4-nitrophenyl)-3,4,5-trimethoxybenzothioamide 39
6. Synthesis of *N*-Ribosyldihydronicotinamide (NRH) 40
7.LCMS Analysis 40
8. References 42
9. NMR Spectra and LCMS data of phenylbenzothiazoles 3-58 43
10.Docking of Benzothiazoles in NQO2 active site **112**

List of Tables

Table S1. Predicted transport and toxicity properties of most potent NQO2 inhibitors. Organ toxicity and toxicity end point, BBB and metabolism predicted by ProTox-3.0 server [5,6]. HERG blocking predicted by PRED-hERG web server [7]. BBB and Metabolism predicted by SwissADME [8] as well. Prediction probabilities indicated for selected compounds/properties in parentheses. 41

List of Figures

Figure S1. ^{1}H-NMR (300 MHz, CDCl spectrum of 3) 43
Figure S2. ^{13}C-NMR (125 MHz, $CDCl_3$) spectrum of 3 44
Figure S3. ^{1}H-NMR (300 MHz, $CDCl_3$) spectrum of 4 44
Figure S4. ^{13}C-NMR (125 MHz, $CDCl_3$) spectrum of 4 44
Figure S5. ^{1}H-NMR (300 MHz, $CDCl_3$) spectrum of 5 45
Figure S6. ^{13}C-NMR (125 MHz, $CDCl_3$) spectrum of 5 45
Figure S7. ^{19}F-NMR (376 MHz, $CDCl_3$) spectrum of 5 46
Figure S8. LCMS spectrum of 5 46
Figure S9. ^{1}H-NMR (300 MHz, $CDCl_3$) spectrum of 6 47
Figure S10. ^{13}C-NMR (125 MHz, $CDCl_3$) spectrum of 6 47
Figure S11. ^{1}H-NMR (300 MHz, $CDCl_3$) spectrum of 7 48
Figure S12. ^{13}C-NMR (125 MHz, $CDCl_3$) spectrum of 7 48
Figure S13. ^{1}H-NMR (300 MHz, $CDCl_3$) spectrum of 8 49

Figure S14. ^{13}C-NMR (125 MHz, $CDCl_3$) spectrum of 8 49
Figure S15. ^{19}F-NMR (376 MHz, $CDCl_3$) spectrum of 8 50
Figure S16. ^{1}H-NMR (300 MHz, $CDCl_3$) spectrum of 9 50
Figure S17. ^{13}C-NMR (125 MHz, $CDCl_3$) spectrum of 9 51
Figure S18. ^{1}H-NMR (300 MHz, $CDCl_3$) spectrum of 10 51
Figure S19. ^{13}C-NMR (125 MHz, $CDCl_3$) spectrum of 10 52
Figure S20. ^{1}H-NMR (400 MHz, DMSO-d_6) spectrum of 11 52
Figure S21. ^{13}C-NMR (125 MHz, DMSO-d_6) spectrum of 11 53
Figure S22. ^{1}H-NMR (400 MHz, $CDCl_3$) spectrum of 12 53
Figure S23. ^{13}C-NMR (125 MHz, $CDCl_3$) spectrum of 12 54
Figure S24. ^{1}H-NMR (400 MHz, DMSO-d_6) spectrum of 13 54
Figure S25. ^{13}C-NMR (125 MHz, DMSO-d_6) spectrum of 13 55
Figure S26. ^{1}H-NMR (300 MHz, $CDCl_3$) spectrum of 14 55
Figure S27. ^{13}C-NMR (125 MHz, $CDCl_3$) spectrum of 14 56
Figure S28. ^{1}H-NMR (300 MHz, acetone-d_6) spectrum of 15 56
Figure S29. ^{13}C-NMR (125 MHz, acetone-d_6) spectrum of 15 57
Figure S30.LCMS spectrum of 15 57
Figure S31. ^{1}H-NMR (300 MHz, $CDCl_3$) spectrum of 16 58
Figure S32. ^{13}C-NMR (125 MHz, $CDCl_3$) spectrum of 16 58
Figure S33. ^{1}H-NMR (300 MHz, $CDCl_3$) spectrum of 17 59
Figure S34.^{13}C-NMR (125 MHz, $CDCl_3$) spectrum of 17 59
Figure S35. ^{1}H-NMR (300 MHz, $CDCl_3$) spectrum of 18 60
Figure S36. ^{13}C-NMR (125 MHz, $CDCl_3$) spectrum of 18 60
Figure S37. ^{19}F-NMR (376 MHz, $CDCl_3$) spectrum of 18 61
Figure S38. ^{1}H-NMR (300 MHz, $CDCl_3$) spectrum of 19 61
Figure S39. ^{13}C-NMR (125 MHz, $CDCl_3$) spectrum of 19 62
Figure S40. ^{1}H-NMR (300 MHz, $CDCl_3$) spectrum of 20 62
Figure S41. ^{13}C-NMR (125 MHz, $CDCl_3$) spectrum of 20 63
Figure S42. ^{1}H-NMR (300 MHz, $CDCl_3$) spectrum of 21 63
Figure S43. ^{13}C-NMR (125 MHz, $CDCl_3$) spectrum of 21 64
Figure S44.^{19}F-NMR (376 MHz, $CDCl_3$) spectrum of 21 64
Figure S45. ^{1}H-NMR (300 MHz, $CDCl_3$) spectrum of 22 65
Figure S46. ^{13}C-NMR (125 MHz, $CDCl_3$) spectrum of 22 65
Figure S47. ^{1}H-NMR (300 MHz, $CDCl_3$) spectrum of 23 66
Figure S48. ^{13}C-NMR (125 MHz, $CDCl_3$) spectrum of 23 66
Figure S49. ^{1}H-NMR (300 MHz, $CDCl_3$) spectrum of 24 67
Figure S50. ^{13}C-NMR (125 MHz, $CDCl_3$) spectrum of 24 67
Figure S51. ^{1}H-NMR (300 MHz, $CDCl_3$) spectrum of 25 68
Figure S52. ^{13}C-NMR (125 MHz, $CDCl_3$) spectrum of 25 68
Figure S53. ^{1}H-NMR (300 MHz, $CDCl_3$) spectrum of 26 69
Figure S54. ^{13}C-NMR (125 MHz, $CDCl_3$) spectrum of 26 69
Figure S55. ^{19}F-NMR (376 MHz, $CDCl_3$) spectrum of 26 70
Figure S56. ^{1}H-NMR (300 MHz, $CDCl_3$) spectrum of 27 70
Figure S57. ^{13}C-NMR (125 MHz, $CDCl_3$) spectrum of 27 71
Figure S58. ^{1}H-NMR (300 MHz, $CDCl_3$) spectrum of 28 71
Figure S59. ^{13}C-NMR (125 MHz, $CDCl_3$) spectrum of 28 72

Figure S60. ^{1}H-NMR (300 MHz, $CDCl_3$) spectrum of 29 72
Figure S61. ^{13}C-NMR (125 MHz, $CDCl_3$) spectrum of 29 73
Figure S62. ^{19}F-NMR (376 MHz, $CDCl_3$) spectrum of 29 73
Figure S63. ^{1}H-NMR (300 MHz, $CDCl_3$) spectrum of 30 74
Figure S64. ^{13}C-NMR (125 MHz, $CDCl_3$) spectrum of 30 74
Figure S65. ^{1}H-NMR (300 MHz, $CDCl_3$) spectrum of 31 75
Figure S66. ^{13}C-NMR (125 MHz, $CDCl_3$) spectrum of 31 75
Figure S67. ^{1}H-NMR (300 MHz, $CDCl_3$) spectrum of 32 76
Figure S68. ^{13}C-NMR (125 MHz, $CDCl_3$) spectrum of 32 76
Figure S69. ^{1}H-NMR (300 MHz, $CDCl_3$) spectrum of 33 77
Figure S70. ^{13}C-NMR (125 MHz, $CDCl_3$) spectrum of 33 77
Figure S71. ^{1}H-NMR (300 MHz, $CDCl_3$) spectrum of 34 78
Figure S72. ^{13}C-NMR (125 MHz, $CDCl_3$) spectrum of 34 78
Figure S73. ^{19}F-NMR (376 MHz, $CDCl_3$) spectrum of 34 79
Figure S74. ^{1}H-NMR (300 MHz, $CDCl_3$) spectrum of 35 79
Figure S75. ^{13}C-NMR (125 MHz, $CDCl_3$) spectrum of 35 80
Figure S76. ^{1}H-NMR (400 MHz, $CDCl_3$) spectrum of 36 80
Figure S77. ^{13}C-NMR (125 MHz, $CDCl_3$) spectrum of 36 81
Figure S78. ^{1}H-NMR (300 MHz, $CDCl_3$) spectrum of 37 81
Figure S79. ^{13}C-NMR (125 MHz, $CDCl_3$) spectrum of 37 82
Figure S80. ^{19}F-NMR (376 MHz, $CDCl_3$) spectrum of 37 82
Figure S81. ^{1}H-NMR (300 MHz, $CDCl_3$) spectrum of 38 83
Figure S82. ^{13}C-NMR (125 MHz, $CDCl_3$) spectrum of 38 83
Figure S83. ^{1}H-NMR (300 MHz, $CDCl_3$) spectrum of 39 84
Figure S84. ^{13}C-NMR (125 MHz, $CDCl_3$) spectrum of 39 84
Figure S85. ^{1}H-NMR (300 MHz, $CDCl_3$) spectrum of 40 85
Figure S86. ^{13}C-NMR (125 MHz, $CDCl_3$) spectrum of 40 85
Figure S87. LCMS spectrum of 40 86
Figure S88. ^{1}H-NMR (300 MHz, $CDCl_3$) spectrum of 41 86
Figure S89. ^{13}C-NMR (125 MHz, $CDCl_3$) spectrum of 41 87
Figure S90. ^{1}H-NMR (300 MHz, $CDCl_3$) spectrum of 42 87
Figure S91. ^{13}C-NMR (125 MHz, $CDCl_3$) spectrum of 42 88
Figure S92. ^{19}F-NMR (376 MHz, $CDCl_3$) spectrum of 42 88
Figure S93. ^{1}H-NMR (300 MHz, $CDCl_3$) spectrum of 43 89
Figure S94. ^{13}C-NMR (125 MHz, $CDCl_3$) spectrum of 43 89
Figure S95. ^{1}H-NMR (300 MHz, $CDCl_3$) spectrum of 44 90
Figure S96. ^{13}C-NMR (125 MHz, $CDCl_3$) spectrum of 44 90
Figure S97. ^{1}H-NMR (300 MHz, $CDCl_3$) spectrum of 45 91
FigureS 98. ^{13}C-NMR (125 MHz, $CDCl_3$) spectrum of 45 91
Figure S99. ^{19}F-NMR (376 MHz, $CDCl_3$) spectrum of 45 92
Figure S100. ^{1}H-NMR (300 MHz, $CDCl_3$) spectrum of 46 92
Figure S101. ^{13}C-NMR (125 MHz, $CDCl_3$) spectrum of 46 93
Figure S102. LCMS spectrum of 46 93
Figure S103. ^{1}H-NMR (300 MHz, $CDCl_3$) spectrum of 47 94
Figure S104. ^{13}C-NMR (125 MHz, $CDCl_3$) spectrum of 47 94
Figure S105. ^{1}H-NMR (400 MHz, DMSO-d_6) spectrum of 48 95

Figure S106. ^{13}C-NMR (125 MHz, DMSO-d_6) spectrum of 48 95
Figure S107. LCMS spectrum of 48 96
Figure S108. ^{1}H-NMR (400 MHz, DMSO-d_6) spectrum of 49 96
Figure S109. ^{13}C-NMR (125 MHz, DMSO-d_6) spectrum of 49 97
Figure S110. LCMS Spectrum of 49 97
Figure S111. ^{1}H-NMR (400 MHz, DMSO-d_6) spectrum of 50 98
Figure S112. ^{13}C-NMR (125 MHz, DMSO-d_6) spectrum of 50 98
Figure S113. ^{1}H-NMR (400 MHz, $CDCl_3$) spectrum of 51 99
Figure S114. ^{13}C-NMR (125 MHz, $CDCl_3$) spectrum of 51 99
Figure S115. ^{1}H-NMR (400 MHz, methanol-d4) spectrum of 52 100
Figure S116. ^{13}C-NMR (125 MHz, methanol-d4) spectrum of 52 100
Figure S117. LCMS spectrum of 52 101
Figure S118. ^{1}H-NMR (300 MHz, $CDCl_3$) spectrum of 53 101
Figure S119. ^{13}C-NMR (125 MHz, $CDCl_3$) spectrum of 53 102
Figure S120. ^{19}F-NMR (376 MHz, $CDCl_3$) spectrum of 53 102
Figure S121. ^{1}H-NMR (300 MHz, $CDCl_3$) spectrum of 54 103
Figure S122. ^{13}C-NMR (125 MHz, $CDCl_3$) spectrum of 54 103
Figure S123. ^{19}F-NMR (376 MHz, $CDCl_3$) spectrum of 54 104
Figure S124. ^{1}H-NMR (300 MHz, $CDCl_3$) spectrum of 55 104
Figure S125. ^{13}C-NMR (125 MHz, $CDCl_3$) spectrum of 55 105
Figure S126. ^{19}F-NMR (376 MHz, $CDCl_3$) spectrum of 55 105
Figure S127. ^{1}H-NMR (300 MHz, $CDCl_3$) spectrum of 56 106
Figure S128. ^{13}C-NMR (125 MHz, $CDCl_3$) spectrum of 56 106
Figure S129. ^{19}F-NMR (376 MHz, $CDCl_3$) spectrum of 56 107
Figure S130. ^{1}H-NMR (300 MHz, $CDCl_3$) spectrum of 57 107
Figure S131. ^{13}C-NMR (125 MHz, $CDCl_3$) spectrum of 57 108
Figure S132. ^{19}F-NMR (376 MHz, $CDCl_3$) spectrum of 57 108
Figure S133. ^{1}H-NMR (300 MHz, $CDCl_3$) spectrum of 58 109
Figure S134. ^{13}C-NMR (125 MHz, $CDCl_3$) spectrum of 58 109
Figure S135. ^{19}F-NMR (376 MHz, $CDCl_3$) spectrum of 58 110
Figure S136.^{1}H-NMR (400 MHz, D_2O) spectrum of *N*-Ribosyldihydronicotinamide (NRH) 110
Figure S137. ^{13}C-NMR (100 MHz, D_2O) spectrum of *N*-Ribosyldihydronicotinamide (NRH) 111
Figure S138. Docked poses of the 9 most potent benzothiazole compounds 15, 49, 40, 48, 46, 3, 5, 41, and 52 (listed in order of decreasing potency) in the NQO2 binding site (PDB ID: 1SG0). Stick representation shows π-π stacking of the docked compounds (magenta) with the isoalloxazine ring of FAD (green). Space-filling representation shows the orientation of the docked small molecules in NQO2 binding pocket, where the substituted phenyl ring is located on the right. 112
Figure S139. Space- filling representation of 6-methoxy benzothiazoles (compounds 40, 3, 24, 32, 16, and 57 listed in order of decreasing activity) docked into the NQO2 binding site (PDB ID: 1SG0). Compounds 40 (3,4,5-trimethoxy phenyl) and 3 (3,5-dimethoxy phenyl) are coloured in magenta, compounds 32 (3,4-dimethoxy phenyl), 16 (2,4-dimethoxy phenyl), and 24 (2,5-dimethoxy phenyl) are coloured in dark magenta, compound 57 (difluoro phenyl) is coloured in dark cyan. 113

1. Chemicals and materials

Chemicals and materials were purchased from Sigma-Aldrich (Gillingham, UK) and Alfa Aesar (Oxford, UK). Solvents were purchased from Fisher Scientific (Loughborough, UK). TLC was performed on silica gel 60 on aluminum sheets with F_{254} and spots were visualized using Mineralight lamp (254/365) UVGL-58. Column chromatography was performed on silica gel silica particle size 40-63 microns, and activated basic alumina (Brookman grade I, pore size 58Å). Microwave reactions were carried out using Biotage® Initiator microwave synthesizer. Melting points were measured using a Stuart® Scientific melting point apparatus SAMP10. Evaporation of solvents was carried out by using a rotary evaporator (Buchi rotavapor® R-200) equipped with a Buchi heating bath B-490. ^{1}H-NMR, ^{13}C-NMR, and ^{19}F-NMR were performed on a Bruker Avance 300 (Division of Pharmacy and Optometry), 400 Bruker and 500 Bruker (School of Chemistry). Chemical shifts are quoted in parts per million (ppm) to the nearest 0.01 ppm and referenced to the residual solvent peak, 7.26 ppm ($CHCl_3$), 2.50 ppm (DMSO), 2.09 ppm for acetone-d_6, and 3.34 ppm (methanol-d4) for ^{1}H-NMR, 77.0 ppm ($CDCl_3$), 39.0 ppm DMSO-d_6, 29.8, and 206.3 ppm for acetone-d_6, 49.3 ppm (methanol-d_4) for ^{13}C-NMR, hexafluorobenzene was used as internal reference at 164 ppm for ^{19}F-NMR. Peak splittings was reported as s (singlet), d (doublet), t (triplet), q (quartet), m (multiplet), br s (broad singlet), br d (broad doublet), dd (doublet of doublet), dt (doublet of triplet), dq (doublet of quartet), td (triplet of doublet), and tt (triplet of triplet). Mass spectroscopy and accurate mass measurement were carried out by the Mass Spectrometry Laboratory, School of Chemistry, University of Manchester. Molecular ions, fragments from molecular ions and other major peaks are reported as mass/charge (*m*/*z*) ratios and are within ± 5 ppm mass units for electrospray (ES) and high-resolution mass spectrometry (HRMS). These were obtained using a Micromass Platform II (ES) and Waters QTOF (HRMS). IR spectroscopy was performed on the solid and liquid states using a J.A.S.C.O Fourier transform infrared spectrophotometer.

2. General procedure for the synthesis of amides A [1]

Thionyl chloride (6.0 mmol) was added dropwise to 3,5-dimethoxybenzoic or 3,5-difluorobenzoic acid (1.5 mmol) at 0 ^{o}C. Reaction mixture was stirred overnight at 45 ^{o}C. Thionyl chloride was evaporated. 3,5-Dimethoxybenzoyl chloride or 3,5-difluorobenzoyl chloride in dry DCM (5.0 ml) was added dropwise to p-anisidine, 4-fluoroaniline, or 4-iodoaniline (1.0mmol) and Et_3N (6.0mmol) in dry DCM (15.0 ml) at 0 ^{o}C. Reaction mixture

was stirred at 0 °C for 15 minutes and then stirred at room temperature overnight. Completion of the reaction was monitored by TLC (30% ethyl acetate in hexane). The workup and purification are detailed under each amide compound.

2.1. 3,5-Dimethoxy-*N*-(4-methoxyphenyl)benzamide [1]
Reaction mixture was concentrated under vacuum and purified using silica gel column chromatography eluting with 10% ethyl acetate in hexane to give a white solid (2.8 g, 62%), m.p. = 108-109 °C. IR (cm^{-1}): 3293 (NH), 2935 (ArCH), 2837 (CH_3), 1638 (C=O), 1591, 1500, 1331, 795; ^{1}H-NMR (300 MHz, $CDCl_3$): δ 7.73 (1H, br s, NH), 7.53 (2H, br d, J = 8.7 Hz, H2`, 6`), 6.97 (2H, d, J = 1.8 Hz, H2, 6), 6.90 (2H, br d, J = 9.0 Hz, H3`, 5`), 6.60 (1H, t, J = 2.1 Hz, H4), 3.84 (6H, s, 2 x OCH_3), 3.81 (3H, s, OCH_3); ^{13}C-NMR (125 MHz, $CDCl_3$) (assignments made with the aid of DEPT-135): δ 165.5 (C=O), 161.0 (C3, 5), 156.6 (C4`), 137.3 (C1), 131.0 (C1`), 122.0 (C3`, 5`), 114.2 (C2`, 6`), 104.9 (C2, 6), 103.7 (C4), 55.6 (2 x OCH_3), 55.5 (OCH_3).

2.2. *N*-(4-Fluorophenyl)-3,5-dimethoxybenzamide
Reaction mixture was quenched with water, extracted with ethyl acetate, washed with 1.0M NaOH, and dried with dry $MgSO_4$. The solution was concentrated under vacuum and purified with column chromatography using 8% ethyl acetate in hexane to give a white solid (0.477 g, 77%), m.p. = 149-150 °C. IR (cm^{-1}): 3299 (NH), 2939 (ArCH), 2836 (CH_3), 1642 (C=O), 1602, 1521, 828; ^{1}H-NMR (300 MHz, $CDCl_3$): δ 7.87 (br s, 1H, NH), 7.59 (2H, dd, J_1 = 9.0 Hz, J_2 = 4.8 Hz, H2`, 6`), 7.05 (2H, t, ^{3}J = 8.9 Hz, H3`, 5`), 6.95 (2H, d, J = 2.1 Hz, H2, 6), 6.60 (1H, t, J = 2.3 Hz, H4), 3.83 (6H, s, 2 x OCH_3); ^{13}C-NMR (125 MHz, $CDCl_3$) (assignments made with the aid of DEPT-135): δ 165.7 (C=O), 161.0 (C3, 5), 159.5 (d, J = 242.5 Hz, C4`), 136.9 (C1), 133.8 (d, J = 2.8 Hz, C1`), 122.1 (d, J = 7.9 Hz, C2`, 6`), 115.6 (d, J = 22.4 Hz, C3`, 5`), 104.9 (C2, 6), 103.8 (C4), 55.5 (2 x OCH_3); ^{19}F-NMR (^{1}H-decoupled, 376 MHz, $CDCl_3$): δ −120.7 (s, F).

2.3. *N*-(4-Iodophenyl)-3,5-dimethoxybenzamide
Reaction mixture was concentrated under vacuum and purified using silica gel column chromatography eluting with 10% of ethyl acetate in hexane to give a white solid (0.210 g, 40%), m.p. = 162-163 °C. IR (cm^{-1}): 3298 (NH), 2961 (ArCH), 2834 (CH_3), 1647 (C=O), 1587, 1516, 1155, 772; ^{1}H-NMR (300 MHz, $CDCl_3$): δ 7.73 (1H, br s, NH), 7.67 (2H, br d, J = 8.7 Hz, H2`, 6`), 7.42 (2H, br d, J = 9.0 Hz, H3`, 5`), 6.95 (2H, d, J = 2.1 Hz, H2, 6), 6.62 (1H, t, J = 2.0 Hz, H4), 3.85 (6H, s, 2 x OCH_3); ^{13}C-NMR (125 MHz, $CDCl_3$ (assignments made with

the aid of DEPT-135): δ 165.5 (C=O), 161.1 (C3, 5), 138.0 (C3`, 5`), 137.6 (C1`), 136.8 (C1), 121.9 (C2`, 6`), 104.9 (C2, 6), 103.9 (C4), 87.8 (C4`), 55.6 (2 x OCH_3).

2.4. 3,5-Difluoro-*N*-(4-methoxyphenyl)benzamide

Reaction mixture was concentrated under vacuum and purified using silica gel column chromatography eluting initially with 10% of ethyl acetate in hexane, increasing to 70% of ethyl acetate in hexane to give a white solid (0.450 g, 89%), m.p. = 198-199 °C. IR (cm^{-1}) 3314 (NH), 3087 (ArCH), 2841 (CH), 1652 (C=O), 1589, 1521, 1234, 971; ^{1}H-NMR (300 MHz, DMSO-d_6): δ 10.24 (1H, br s, NH), 7.66 (4H, br d, J = 9.0 Hz, H2, 6, 2`, 6`), 7.51 (1H, t, J = 9.2 Hz, H4), 6.94 (2H, br d , J = 9.0 Hz, H3`, 5`), 3.75 (3H, s, OCH_3); ^{13}C-NMR (125 MHz, DMSO-d_6) (assignments made with the aid of DEPT-135): δ 162.3 (t, J = 2.8 Hz, C=O), 162.2 (dd, $^1J_1$ = 245.7 Hz, J_2 = 12.7Hz, C3, 5), 155.8 (C4`), 138.4 (t, J = 8.3 Hz, C1), 131.6 (C1`), 122.1 (C2`, 6`), 113.8 (C3`, 5`), 111.0 (dd, J_1 = 20.3 Hz, J_2 = 6.5 Hz, C2, 6), 106.8 (t, J = 25.7 Hz, C4), 55.2 (OCH_3); ^{19}F-NMR (^{1}H-decoupled, 376 MHz, DMSO-d_6): δ −111.2 (s, 2 x F); ^{19}F-NMR (^{1}H-coupled, 376 MHz, DMSO-d_6): δ −111.2 (t, J = 7.9 Hz, 2F).

3. General procedure for synthesis of amides B

appropriate benzoic acids (3,5-Difluorobenzoic acid, 3,5-dimethoxybenzoic acid, 2,4-dimethoxybenzoic acid, 2,5-dimethoxybenzoic acid, 3,4-dimethoxybenzoic acid, 3,4, 5-trimethoxybenzoic, acid 3-methoxybenzoic acid,) (1.1 mmol), appropriate *p*-anilines (4-bromoaniline, 4-(trifluoromethyl) aniline, 4-fluoroaniline, *p*-anisidine, 4-chloroaniline, 4-aminobenzonitrile, 4-iodoaniline, 4-nitroaniline) (1.0 mmol) and PCl_3 (1.0 mmol) in dry acetonitrile (15.0 ml) were heated in a microwave for 5 minutes at 150 °C[2]. Amide formation was identified using TLC (30%, 50% ethyl acetate in hexane). Reaction mixtures were quenched with water, acetonitrile was evaporated. Reaction residues were either filtred, extracted with ethyl acetate or purified using an activated basic alumina column. Details of workup and column purification are specified for each compound.

3.1. 3,5-Dimethoxy-*N*-(4-(trifluoromethyl)phenyl)benzamide

Reaction mixture was extracted with ethyl acetate, and the extract was washed with 2.0 M NaOH. The organic layer was dried with dry $MgSO_4$ and evaporated to give a white solid (0.335 g, 98%), m.p. = 169-170 °C. IR (cm^{-1}): 3290 (NH), 2937 (ArCH), 2840 (CH_3), 1653 (C=O), 1591, 1515, 1328, 780, 678; ^{1}H-NMR (300 MHz, $CDCl_3$): δ 7.93 (1H, br s, NH), 7.77 (2H, br d, J = 8.4 Hz, H3`, 5`), 7.62 (2H, br d, J = 8.7 Hz, H2`, 6`), 6.97 (2H, d, J = 2.1 Hz, H2, 6), 6.63

(1H, t, J = 2.1 Hz, H4), 3.85 (6H, s, 6H, 2 x OCH_3); ^{13}C-NMR (125 MHz, $CDCl_3$) (assignments made with the aid of DEPT-135: δ 165.7 (C=O), 161.1 (C3, 5), 140.9 (C1`), 136.6 (C1), 126.4 (q, J = 3.4 Hz, C3`, 5`), 126.3 (q, J = 32.5 Hz, C4`), 124.0 (q, J = 269.5 Hz, CF_3), 119.7 (C2`, 6`), 105.0 (C2, 6), 104.0 (C4), 55.7 (2 x OCH_3); ^{19}F-NMR (^{1}H-decoupled, 376 MHz, $CDCl_3$): δ − 65.3 (s, CF_3).

3.2. 3,5-Dimethoxy-*N*-(4-nitrophenyl)benzamide

Reaction mixture was purified using basic activated alumina column chromatography eluting with DCM to give a yellow solid (0.400 g, 92%), m.p. = 175-177 °C. IR (cm^{-1}): 3293 (NH), 3003 (ArCH), 1655 (C=O), 1587, 1204, 850; ^{1}H-NMR (300 MHz, DMSO-d_6): δ 10.72 (1H, br s, NH), 8.26 (2H, br d, J = 8.7 Hz, H3`, 5`), 8.05 (2H, br d, J = 8.4 Hz, H2`, 6`), 7.11 (2H, br s, H2, 6), 6.76 (1H, br s, H4), 3.8 3 (6H, s, 2 x OCH_3); ^{13}C-NMR (125 MHz, DMSO-d_6) (assignments made with the aid of DEPT-135): δ 165.8 (C=O), 160.4 (C3, 5), 145.3 (C4`), 142.5 (C1`), 136.2 (C1), 124.7 (C3`, 5`), 119.9 (C2`, 6`), 105.9 (C2, 6), 103.8 (C4), 55.5 (2 x OCH_3).

3.3. *N*-(4-Cyanophenyl)-3,5-dimethoxybenzamide

Reaction mixture was extracted with ethyl acetate, and the extract was washed with 2.0 M NaOH. The organic layer was dried with dry $MgSO_4$ and evaporated to give a white solid (0.388 g, 90%), m.p. = 171-172 °C. IR (cm^{-1}): 3307 (NH), 2943 (ArCH), 2837 (CH_3), 2225 (CN), 1649 (C=O), 1585, 1203, 834, 671; ^{1}H-NMR (300 MHz, $CDCl_3$): δ 8.02 (1H, br s, NH), 7.78 (2H, br d, J = 8.7 Hz, H2`, 6`), 7.65 (2H, br d, J = 8.7 Hz, H3`, 5`), 6.96 (2H, d, J = 2.1 Hz, H2, 6), 6.64 (1H, t, J = 2.1 Hz, H4), 3.84 (6H, s, 2 x OCH_3); ^{13}C-NMR (125 MHz, $CDCl_3$) (assignments made with the aid of DEPT-135): δ 165.7 (C=O), 161.2 (C3, 5), 141.9 (C1`), 136.3 (C1), 133.3 (C3`, 5`), 119.9 (2`, 6`), 118.8 (CN), 107.4 (C4`), 105.1 (C2, 6), 104.2 (C4), 55.7 (2 x OCH_3).

3.4. *N*-(4-Chlorophenyl)-3,5-dimethoxybenzamide

Reaction mixture was extracted with ethyl acetate, and the extract was washed with 2.0 M NaOH. The organic layer was dried with dry $MgSO_4$ and evaporated to give a white solid (**196**) (0.376 g, 82%), m.p = 156-157 °C lit (156-157 °C).[3] IR (cm^{-1}): 3306 (NH), 2938 (ArCH), 2839 (CH_3), 1645 (C=O), 1590, 1324, 684; ^{1}H-NMR (300 MHz, $CDCl_3$): δ 7.86 (1H, br s, NH), 7.58 (2H, br d, J = 8.7 Hz, H2`, 6`), 7.32 (2H, br d, J = 8.7 Hz, H3`, 5`), 6.95 (2H, d, J = 2.1 Hz, H2, 6), 6.61 (1H, t, J = 2.1 Hz, H4), 3.83 (6H, s, 2 x OCH_3); ^{13}C-NMR (75 MHz, $CDCl_3$) (assignments made with the aid of DEPT-135): δ 165.5 (C=O), 161.1 (C3, 5), 136.9

(C1`), 136.4 (C4`), 129.6 (C1), 129.1 (3`, 5`), 121.4 (C2`, 6`), 105.0 (C2, 6), 103.9 (C4), 55.6 (2 x OCH_3).

3.5. *N*-(4-Bromophenyl)-3,5-dimethoxybenzamide

Reaction mixture was extracted with ethyl acetate, and the extract was washed with 2.0 M NaOH. The organic layer was dried with dry $MgSO_4$ and evaporated to give an off white solid (0.366 g, 94%), m.p. = 139-140 ^oC, lit (131-133° C) [3] IR (cm^{-1}): 3308 (NH), 2935 (ArCH), 2836 (CH_3), 1647 (C=O), 1589, 1150, 678; ^{1}H-NMR (300 MHz, $CDCl_3$): δ 7.87 (1H, br s, NH), 7.54 (2H, br d, J = 9.0 Hz, H2`, 6`), 7.46 (2H, br d, J = 8.7 Hz, H3`, 5`), 6.95 (2H, d, J = 2.1 Hz, H2, 6), 6.61 (1H, t, J = 2.3 Hz, H4), 3.83 (6H, s, 2 x OCH_3); ^{13}C-NMR (125 MHz, $CDCl_3$) (assignments made with the aid of DEPT-135): δ 165.5 (C=O), 161.1 (C3, 5), 136.9 (C1`), 136.8 (C1), 132.0 (C3`, 5`), 121.7 (2`, 6`), 117.2 (C4`), 105.0 (C2, 6), 103.9 (C4), 55.6 (2 x OCH_3).

3.6. 3,5-Difluoro-*N*-(4-fluorophenyl)benzamide

Reaction mixture was extracted with ethyl acetate, and the extract was washed with 2.0 M NaOH. The organic layer was dried with dry $MgSO_4$ and evaporated to give a white solid (0.190 g, 84%), m.p. = 160-161 ^oC. IR (cm^{-1}): 3301 (NH), 3096 (ArCH), 1645 (C=O), 1610, 1507, 1263, 1222; ^{1}H-NMR (300 MHz, DMSO-d_6): δ 10.47 (1H, br s, NH), 7.79 (2H, dd, J_1 = 9.0 Hz, J_2 = 5.1Hz, H2`, 6`), 7.69 (2H, br d, J = 6.6 Hz, H2, 6), 7.53 (1H, tt, J_1 = 9.3 Hz, J_2 = 2.2 Hz, H4), 7.21 (2H, t, J = 8.9 Hz, H3`, 5`); ^{13}C-NMR (75 MHz, MDSO-d_6) (assignments made with the aid of DEPT-135): δ 162.7 (t, J = 2.8 Hz, C=O), 162.3 (dd, J_1 = 245.7 Hz, J_2 = 12.6 Hz, C3, 5), 158.5 (d, J = 239.4 Hz, C4`), 138.1 (t, J = 8.5 Hz, C1), 134.9 (d, J = 2.7 Hz, C1`), 122.4 (d, J = 7.9 Hz, C2`, 6`), 115.2 (d, J = 22.2 Hz, C3`, 5`), 111.1 (dd, J_1 = 17.6 Hz, J_2 = 8.6 Hz, C2, 6), 107.0 (t, J = 25.7 Hz, C4); ^{19}F NMR (^{1}H-decoupled, 376 MHz, DMSO-d_6): δ −111.1 (s, 2F), −120.5 (s,F).

3.7. 2,4-Dimethoxy-*N*-(4-methoxyphenyl)benzamide

Reaction mixture was purified using basic activated alumina column chromatography eluting with DCM to give an off white solid (0.407 g, 87%), m.p. = 105-106 ^oC. IR (cm^{-1}): 3359 (NH), 3006 (ArCH), 2836 (CH_3), 1654 (C=O), 1600, 1500, 1026, 829; ^{1}H-NMR (300 MHz, $CDCl_3$): δ 9.57 (1H, br s, NH), 8.25 (1H, d, J = 9.0 Hz, H6), 7.56 (2H, br d, J = 9.0 Hz, H2`, 6`), 6.89 (2H, br d, J = 9.0 Hz, H3`, 5`), 6.64 (1H, dd, J_1 = 8.7 Hz, J_2 = 2.1 Hz, H5), 6.52 (1H, d, J = 2.1 Hz, H3), 4.00 (3H, s, OCH_3), 3.86 (3H, s, OCH_3), 3.80 (3H, s, OCH_3); ^{13}C-NMR (125 MHz, $CDCl_3$) (assignments made with the aid of DEPT-135): δ 163.6 (C4), 162.9 (C=O), 158.5 (C4`),

156.1 (C2), 134.1 (C6), 131.8 (C1`), 122.0 (C2`, 6`), 114.8 (C1), 114.1 (C3`, 5`), 105.6 (C5), 98.7 (C3), 56.1 (OCH_3), 55.5 (OCH_3), 55.4 (OCH_3).

3.8. *N*-(4-Fluorophenyl)-2,4-dimethoxybenzamide

Reaction mixture was purified using basic activated alumina column chromatography eluting with DCM to give a white solid (0.407 g, 87%), m.p. = 110-111 °C. IR (cm^{-1}): 3336 (NH), 2948 (ArCH), 2839 (CH_3), 1646 (C=O), 1608, 1501, 924; ^{1}H-NMR (300 MHz, $CDCl_3$): δ 9.67 (1H, br s, NH), 8.25 (1H, d, J = 8.7 Hz, H6), 7.63 (2H, dd, J_1 = 9.0 Hz, J_2 = 4.8 Hz, H2`, 6`), 7.04 (2H, t, J = 8.7 Hz, H3`, 5`), 6.65 (1H, dd, J_1 = 8.9 Hz, J_2 = 2.3Hz, H5), 6.52 (1H, d, J = 2.1 Hz, H3), 4.02 (3H, s, OCH_3), 3.87 (s, 3H, OCH_3); ^{13}C-NMR (125 MHz, $CDCl_3$) (assignments made with the aid of DEPT-135): δ 163.7 (C4), 163.0 (C=O), 159.1 (d, J = 241.1 Hz, C4`), 158.5 (C2), 134.6 (d, J = 2.8 Hz, C1`), 134.1 (C6), 122.0 (d, J = 7.8 Hz, C2`, 6`), 115.4 (d, J = 21.9 Hz, C3`, 5`), 114.3 (C1), 105.7 (C5), 98.6 (C3), 56.1 (OCH_3), 55.5 (OCH_3); ^{19}F-NMR (^{1}H-decoupled, 376 MHz, $CDCl_3$): δ −122.0 (s, F); ^{19}F-NMR (^{1}H-coupled, 376 MHz, $CDCl_3$) δ −122.0 −122.1(m, F).

3.9. *N*-(4-Iodophenyl)-2,4-dimethoxybenzamide

Reaction mixture was purified using basic activated alumina column chromatography eluting with DCM to give a white solid (0.310 g, 63%), m.p. = 110-111 °C. IR (cm^{-1}): 3331 (NH), 3010 (ArCH), 2837 (CH_3), 1662 (C=O), 1582, 1527, 839; ^{1}H-NMR (300 MHz, $CDCl_3$): δ 9.71 (1H, br s, NH), 8.24 (1H, d, J = 9.0 Hz, H6), 7.64 (2H, br d, J = 8.7 Hz, H2`, 6`), 7.45 (2H, br d, J = 8.7 Hz, H3`, 5`), 6.66 (1H, dd, J_1 = 8.9 Hz, J_2 = 2.3 Hz, H5), 6.53 (1H, d, J = 2.1 Hz, H3), 4.03 (3H, s, OCH_3), 3.88 (3H, s, OCH_3); ^{13}C-NMR (125 MHz, $CDCl_3$) (assignments made with the aid of DEPT-135): δ 163.9 (C4), 163.1 (C=O), 158.5 (C1`), 138.4 (C2), 137.7 (C3`, 5`), 134.1 (C6), 122.1 (C2`, 6`), 114.3 (C1), 105.7 (C5), 98.7 (C3), 86.7 (C4`), 56.2 (OCH_3), 55.5 (OCH_3).

3.10. *N*-(4-Bromophenyl)-2,4-dimethoxybenzamide

Reaction mixture was extracted with ethyl acetate, washed with 2.0 M NaOH. The organic layer was dried with dry $MgSO_4$ and evaporated to give a white solid (0.310 g, 68%), m.p. = 135-136 °C. IR (cm^{-1}): 3348 (NH), 2938 (ArCH), 2838 (CH_3), 1659 (C=O), 1591, 1515, 1435, 837; ^{1}H-NMR (300 MHz, $CDCl_3$): δ 9.71 (1H, br s, NH), 8.23 (1H, d, J = 9.0 Hz, H6), 7.56 (br d, J = 8.7 Hz, 2H, H2`, 6`), 7.44 (br d, J = 8.7 Hz, 2H, H3`, 5`), 6.65 (1H, dd, J_1 = 8.9 Hz, J_2 = 2.3 Hz, H5), 6.52 (1H, d, J = 2.1 Hz, H3), 4.02 (3H, s, OCH_3), 3.86 (3H, s, OCH_3); ^{13}C-NMR (75 MHz, $CDCl_3$) (assignments made with the aid of DEPT-135): δ 163.9 (C4), 163.1 (C=O),

158.5 (C1`), 137.7 (C2), 134.2 (C6), 131.8 (C3`, 5`), 121.8 (C2`, 6`), 116.2 (C4`), 114.4 (C1), 105.8 (C5), 98.8 (C3), 56.2 (OCH_3), 55.6 (OCH_3).

3.11. 2,4-Dimethoxy-*N*-(4-(trifluoromethyl)phenyl)benzamide

Reaction mixture was extracted with ethyl acetate, and the extract washed with 2.0 M NaOH. The organic layer was dried with dry $MgSO_4$ and evaporated to give a white solid (0.320 g, 79%), m.p. = 150-151 ^{o}C. IR (cm^{-1}): 3351 (NH), 2948 (ArCH), 1661 (C=O), 1598, 824; ^{1}H-NMR (300 MHz, $CDCl_3$): δ 9.88 (1H, br s, NH), 8.25 (1H, d, J = 9.0 Hz, H6), 7.78 (2H, br d, J = 8.4 Hz, H3`, 5`), 7.59 (2H, br d, J = 8.7 Hz, H2`, 6`), 6.67 (1H, dd, J_1 = 8.7 Hz, J_2 = 2.1 Hz, H5), 6.54 (1H, d, J = 2.1 Hz, H3), 4.05 (3H, s, OCH_3), 3.88 (3H, s, OCH_3); ^{13}C-NMR (125 MHz, $CDCl_3$) (assignments made with the aid of DEPT-135: δ 164.1 (C=O), 163.3 (C4), 158.6 (C2), 141.7 (C1`), 134.3 (C6), 126.1 (q, J = 3.8 Hz, C3`, 5`), 125.4 (q, J = 32.4 Hz, C4`), 124.2 (q, J = 269.7 Hz, CF_3), 119.8 (C2`, 6`), 114.1 (C1), 105.9 (C5), 98.7 (C3), 56.2 (OCH_3), 55.6 (OCH_3); ^{19}F-NMR (^{1}H-decoupled, 376 MHz, $CDCl_3$): δ − 65.1 (s, CF_3).

3.12. *N*-(4-Chlorophenyl)-2,4-dimethoxybenzamide

Reaction mixture was extracted with ethyl acetate, and the extract was washed with 2.0 M NaOH. The organic layer was dried with dry $MgSO_4$ and evaporated to give an off white solid (0.370 g, 81%), m.p. = 117-118 ^{o}C. IR (cm^{-1}): 3348 (NH), 2940 (ArCH), 2839 (CH_3), 1660 (C=O), 1599, 1542, 1209, 828; ^{1}H-NMR (300 MHz, $CDCl_3$): δ 9.72 (1H, br s, NH), 8.24 (1H, d, J = 9.0 Hz, H6), 7.62 (2H, br d, J = 8.7 Hz, H2`, 6`), 7.30 (2H, br d, J = 8.7 Hz, H3`, 5`), 6.66 (1H, dd, J_1 = 8.9 Hz, J_2 = 2.3 Hz, H5), 6.53 (1H, d, J = 2.1 Hz, H3), 4.03 (3H, s, OCH_3), 3.88 (3H, s, OCH_3); ^{13}C-NMR (125 MHz, $CDCl_3$) (assignments made with the aid of DEPT-135): δ 163.9 (C4), 163.1 (C=O), 158.5 (C2), 137.2 (C1`), 134.2 (C6), 128.9 (C3`, 5`), 128.7 (C4`), 121.5 (C2`, 6`), 114.4 (C1), 105.8 (C5), 98.8 (C3), 56.2 (OCH_3), 55.6 (OCH_3).

3.13. *N*-(4-Cyanophenyl)-2,4-dimethoxybenzamide

Reaction mixture was purified using basic activated alumina column chromatography eluting initially with DCM, increasing to 2% MeOH in DCM to give a white solid (0.460 g, 94%), m.p. = 171-172 ^{o}C. IR (cm^{-1}): 3333 (NH), 2222 (CN), 1669 (C=O), 1592, 1537, 807; ^{1}H-NMR (300 MHz, DMSO-d_6): δ 10.29 (1H, br s, NH), 7.93 (2H, br d, J = 8.7 Hz, H2`, 6`), 7.79 (2H, br d, J = 8.7 Hz, H3`, 5`), 7.71 (1H, d, J = 8.4 Hz, H6), 6.69 (1H, dd, J_1 = 6.9 Hz, J_2 = 1.8 Hz, H5), 6.65 (1H, d, J = 1.8 Hz, H3), 3.93 (3H, s, OCH_3), 3.84 (3H, s, OCH_3); ^{13}C-NMR (75 MHz, DMSO-d_6) (assignments made with the aid of DEPT-135): δ 164.3 (C4), 163.3 (C=O), 158.5

(C1`), 143.2 (C2), 133.2 (C3`, 5`), 132.0 (C6), 119.8 (C2`, 6`), 119.1 (CN), 115.9 (C1), 105.6 (C5), 105.0 (C4`), 98.6 (C3), 56.2 (OCH_3), 55.6 (OCH_3).

3.14. 2,4-Dimethoxy-*N*-(4-nitrophenyl)benzamide

The precipitated solid was filtered, washed with water, and dried to give a yellow solid (0.395 g, 90%). m.p. = 197 ^oC. IR (cm^{-1}): 3315 (NH), 2952 (ArCH), 2843 (CH_3), 1680 (C=O), 1608, 1551, 751; ^1H-NMR (300 MHz, DMSO-d_6): δ 10.46 (1H, br s, NH), 8.24 (2H, br d, J = 9.0 Hz, H3`, 5`), 7.99 (2H, br d, J = 9.3 Hz, H2`, 6`), 7.71 (1H, d, J = 8.4 Hz, H6), 6.70 (1H, dd, J_1 = 7.2 Hz, J_2 = 1.8 Hz, H5), 6.66 (1H, d, J = 1.8 Hz, H3), 3.94 (3H, s, OCH_3), 3.85 (3H, s, OCH_3); ^{13}C-NMR (125 MHz, DMSO-d_6) (assignments made with the aid of DEPT-135): δ 164.6 (C4), 163.4 (C=O), 158.5 (C1`), 145.3 (C4`), 142.2 (C2), 132.0 (C6), 124.9 (C3`, 5`), 119.4 (C2`, 6`), 115.8 (C1), 105.9 (C5), 98.6 (C3), 56.2 (OCH_3), 55.7 (OCH_3).

3.15. 2,5-Dimethoxy-*N*-(4-methoxyphenyl)benzamide

Reaction mixture was eluted through activated basic alumina column using DCM to give an off white solid (0.410 g, 92%), m.p. = 91-93 ^oC. IR (cm^{-1}): 3304 (NH), 2959 (ArCH), 2834 (CH_3), 1641 (C=O), 1604, 1511, 805; ^1H-NMR (300 MHz, $CDCl_3$): δ 9.83 (1H, br s, NH), 7.83 (1H, d, J = 3.0 Hz, H6), 7.59 (2H, br d, J = 9.0 Hz, H2`, 6`), 7.03 (1H, dd, J_1 = 9.0 Hz, J_2 = 3.0Hz, H4), 6.95 (1H, d, J = 9.0 Hz, H3), 6.90 (2H, br d, J = 9.0 Hz, H3`, 5`), 3.99 (3H, s, OCH_3), 3.83 (3H, s, OCH_3), 3.80 (3H, s, OCH_3); ^{13}C-NMR (125 MHz, $CDCl_3$) (assignments made with the aid of DEPT-135): δ 162.7 (C=O), 156.2 (C4`), 154.1 (C5), 151.4 (C2), 131.5 (C1`), 122.3 (C1), 121.9 (C2`, 6`), 119.6 (C4), 115.5 (C3), 114.1 (C3`, 5`), 113.2 (C6), 56.8 (OCH_3), 55.8 (OCH_3), 55.4 (OCH_3).

3.16. *N*-(4-Fluorophenyl)-2,5-dimethoxybenzamide

Reaction mixture was eluted through activated basic alumina column using 2% MeOH in DCM to give a white solid (0.360 g, 73%), m.p. = 99-100 ^oC. IR (cm^{-1}): 3339 (NH), 2848 (CH), 1672 (C=O), 1550, 1042, 922; ^1H-NMR (300 MHz, DMSO-d_6): δ 10.20 (1H, br s, NH), 7.77 (2H, dd, J = 8.7 Hz, J_2 = 5.1Hz, H2`, 6`), 7.21 (1H, d, J = 1.8 Hz, H6), 7.18 (2H, t, J = 9.0 Hz, H3`, 5`), 7.12 (1H, d, J = 9.0 Hz, H3), 7.07 (1H, dd, J_1 = 9.0 Hz, J_2 = 2.9Hz, H4), 3.85 (3H, s, OCH_3), 3.75 (3H, s, OCH_3); ^{13}C-NMR (75 MHz, DMSO-d_6) (assignments made with the aid of DEPT-135): δ 163.9 (C=O), 158.2 (d, J = 238.6 Hz, C4`), 153.0 (C5), 150.6 (C2), 135.3 (d, J = 2.5 Hz, C1`), 125.1 (C1), 121.5 (d, J = 7.7Hz, C2`, 6`), 117.3 (C4), 115.2 (d, J = 22.0 Hz, C3`, 5`), 114.6 (C3), 113.5 (C6), 56.4 (OCH_3), 55.5 (OCH_3); ^{19}F-NMR (^1H-decoupled, 376

MHz, DMSO-d_6): δ −121.3 (s, F); ^{19}F-NMR (^{1}H-coupled, 376 MHz, DMSO-d_6): δ − 121.2 − 121.6 (m, F).

3.17. *N*-(4-Iodophenyl)-2,5-dimethoxybenzamide

The reaction mixture was purified using basic activated alumina column chromatography eluting with 1% MeOH in DCM to give a white solid (0.320 g, 92%), m.p. = 108-109 °C. IR (cm^{-1}): 3336 (NH), 3958 (ArCH), 2841 (CH_3), 1663 (C=O), 1533, 800; ^{1}H-NMR (300 MHz, $CDCl_3$): δ 9.96 (1H, br s, NH), 7.80 (1H, d, J = 3.0 Hz, H6), 7.64 (2H, br d, J = 8.7 Hz, H2`, 6`), 7.46 (2H, br d, J = 8.7 Hz, H3`, 5`), 7.05 (1H, dd, J_1 = 9.0 Hz, J_2 = 3.3 Hz, H4), 6.96 (1H, d, J = 9.0 Hz, H3), 4.0 (3H, s, OCH_3), 3.83 (3H, s, OCH_3); ^{13}C-NMR (75 MHz, $CDCl_3$) (assignments made with the aid of DEPT-135): δ 163.0 (C=O), 154.2 (C5), 151.4 (C2), 138.2 (C1`), 137.8 (C3`, 5`), 122.1 (C2`, 6`), 122.0 (C1), 120.1 (C4), 115.6 (C3), 113.3 (C6), 87.1 (C4`), 56.8 (OCH_3), 55.8 (OCH_3).

3.18. *N*-(4-Bromophenyl)-2,5-dimethoxybenzamide

Reaction mixture was purified using basic activated alumina column chromatography eluting with 1% MeOH in DCM to give a white solid (0.360 g, 85%), m.p. = 115-117 °C. IR (cm^{-1}): 3323 (NH), 2952 (ArCH), 2834 (CH_3), 1659 (C=O), 1603, 1544, 805; ^{1}H-NMR (300 MHz, DMSO-d_6): δ 10.27 (1H, br s, NH), 7.72 (2H, br d, J = 8.7 Hz, H2`, 6`), 7.52 (2H, br d, J = 8.7 Hz, H3`, 5`), 7.19 (1H, d, J = 2.4 Hz, H6), 7.12 (1H, d, J = 9.0 Hz, H3), 7.08 (1H, dd, J_1 = 9.2 Hz, J_2 = 2.3 Hz, H4), 3.84 (3H, s, OCH_3), 3.75 (3H, s, OCH_3); ^{13}C-NMR (125 MHz, DMSO-d_6) (assignments made with the aid of DEPT-135): δ 164.2 (C=O), 153.0 (C5), 150.5 (C2), 138.3 (C1`), 131.5 (C3`, 5`), 125.1 (C4`), 121.6 (C2`, 6`), 117.4 (C4), 115.2 (C1), 114.5 (C3), 113.5 (C6), 56.4 (OCH_3), 55.6 (OCH_3).

3.19. 2,5-Dimethoxy-N-(4-(trifluoromethyl)phenyl)benzamide

Reaction mixture was extracted with ethyl acetate, and the extract was washed with 2.0 M NaOH. The organic layer was dried with dry $MgSO_4$ and evaporated to give a white solid (0.404 g, 100%), m.p. = 99-101 °C. IR (cm^{-1}): 3315 (NH), 2952 (ArCH), 2833 (CH), 1683 (C=O), 1604, 1545, 1320, 838; ^{1}H-NMR (300 MHz, $CDCl_3$): δ 10.16 (1H, br s, NH), 7.83 (1H, d, J = 3.0 Hz, H6), 7.81 (2H, brd, J = 8.1 Hz, H3`, 5`), 7.61 (2H, br d, J = 8.7 Hz, H2`, 6`), 7.08 (1H, dd, J_1 = 8.9 Hz, J_2 = 3.2 Hz, H4), 6.99 (1H, br d, J = 9.0 Hz, H3), 4.04 (3H, s, OCH_3), 3.85 (3H, s, OCH_3); ^{13}C-NMR (75 MHz, $CDCl_3$) (assignments made with the aid of DEPT-135): δ 163.2 (C=O), 154.2 (C5), 151.5 (C2), 141.4 (C1`), 126.2 (q, J = 3.8 Hz, C3`, 5`), 125.8 (q, J = 32.4 Hz, C4`), 124.2 (q, J = 269.7 Hz, CF_3), 121.8 (C1), 120.3 (C4), 119.9 (C2`, 6`), 115.7 (C3),

113.3 (C6), 56.9 (OCH_3), 55.8 (OCH_3); ^{19}F-NMR (^{1}H-decoupled, 376 MHz, $CDCl_3$): δ − 65.2 (s, CF_3).

3.20. *N*-(4-Chlorophenyl)-2,5-dimethoxybenzamide
Reaction mixture was purified using basic activated alumina column chromatography eluting with 1% MeOH in DCM to give a white solid (0.455g, 100%), m.p. = 113-114 °C. IR (cm^{-1}): 3329 (NH), 1670 (C=O), 1543, 1401, 1280, 806; ^{1}H-NMR (300 MHz, DMSO-d_6): δ 10.27 (1H, br s, NH), 7.78 (2H, br d, J = 8.7 Hz, H2`, 6`), 7.39 (2H, br d, J = 9.0 Hz, H3`, 5`), 7.20 (1H, br d, J = 2.4 Hz, H6), 7.12 (1H, br d, J = 9.0 Hz, H3), 7.08 (1H, dd, J_1 = 9.2 Hz, J_2 = 2.3 Hz, H4), 3.84 (s, 3H, OCH_3), 3.75 (s, 3H, OCH_3); ^{13}C-NMR (75 MHz, DMSO-d_6) (assignments made with the aid of DEPT-135): δ 164.1 (C=O), 153.0 (C5), 150.5 (C2), 137.9 (C1`), 128.5 (C3`,5`), 127.1 (C4`), 125.1 (C1), 121.2 (C2`, 6`), 117.4 (C4), 114.5 (C3), 113.5 (C6), 56.4 (OCH_3), 55.6 (OCH_3).

3.21. *N*-(4-Cyanophenyl)-2,5-dimethoxybenzamide
Reaction mixture was purified using basic activated alumina column chromatography eluting with 2% MeOH in DCM to give a white solid (0.471 g, 99%), m.p. = 163-164 °C. IR (cm^{-1}): 3320 (NH), 2949 (ArCH), 2834 (CH_3), 2221 (CN), 1670 (C=O), 1594, 1494, 801; ^{1}H-NMR (300 MHz, DMSO-d_6): δ 10.56 (1H, br s, NH), 7.93 (2H, br d, J = 8.7 Hz, H2`, 6`), 7.81 (2H, br d, J = 8.7 Hz, H3`, 5`), 7.19 (1H, d, J = 2.7 Hz, H6), 7.13 (1H, d, J = 9.0 Hz, H3), 7.09 (1H, dd, J_1 = 9.3 Hz, J_2 = 2.7 Hz, H4), 3.84 (3H, s, OCH_3), 3.75 (3H, s, OCH_3); ^{13}C-NMR (125 MHz, DMSO-d_6) (assignments made with the aid of DEPT-135): δ 164.8 (C=O), 153.0 (C5), 150.6 (C2), 143.1 (C1`), 133.2 (C3`, 5`), 124.9 (C1), 119.7 (C2`, 6`), 119.1 (CN), 117.7 (C4), 114.5 (C3), 113.5 (C6), 105.3 (C4`), 56.4 (OCH_3), 55.6 (OCH_3).

3.22. 2,5-Dimethoxy-*N*-(4-nitrophenyl)benzamide
Reaction mixture was purified using basic activated alumina column chromatography eluting with 2% MeOH in DCM to give a yellow solid (0.430 g, 98%), m.p. = 170-171 °C. IR (cm^{-1}): 3305 (NH), 2941 (ArCH), 2836 (CH_3), 1676 (C=O), 1550, 1406, 849; ^{1}H-NMR (300 MHz, $CDCl_3$): δ 10.33 (1H, br s, NH), 8.23 (2H, br d, $^3J_{HH}$ = 9.3 Hz, H3`, 5`), 7.84 (2H, br d, J = 9.3 Hz, H2`, 6`), 7.80 (1H, d, J = 3.0 Hz, H6), 7.10 (1H, dd, J_1 = 9.0 Hz, J_2 = 3.0Hz, H4), 7.00 (2H, d, J = 9.0 Hz, H3), 4.06 (3H, s, OCH_3), 3.85 (3H, s, OCH_3); ^{13}C-NMR (125 MHz, $CDCl_3$) (assignments made with the aid of DEPT-135): δ 163.4 (C=O), 154.3 (C5), 151.5 (C2), 144.2 (C1`), 143.4 (C4`), 125.1 (C3`, 5`), 121.3 (C1), 120.7 (C2`, 6`), 119.6 (C3), 115.7 (C4), 113.3 (C6), 56.9 (OCH_3), 55.8 (OCH_3).

3.23. 3,4-Dimethoxy-*N*-(4-methoxyphenyl)benzamide

Reaction mixture was purified using basic activated alumina column chromatography eluting with 2% MeOH in DCM to give an off white solid (0.398 g, 85%), m.p. = 175-177 ^{o}C. IR (cm^{-1}): 3296 (NH), 2954 (ArCH), 2840 (CH_3), 1639 (C=O), 1595, 1018, 825; ^{1}H-NMR (300 MHz, $CDCl_3$): δ 7.94 (1H, br s, NH), 7.52 (2H, br d, J = 8.7 Hz, H2`, 6`), 7.47 (1H, br d, J = 1.5 Hz, H2), 7.38 (1H, dd, J_1 = 8.1 Hz, J_2 = 1.5 Hz, H6), 6.87 (2H, br d, J = 9.0 Hz, H3`, 5`), 6.84 (1H, br d, J = 8.4 Hz, H5), 3.91 (3H, s, OCH_3), 3.89 (3H, s, OCH_3), 3.79 (s, 3H, OCH_3); ^{13}C-NMR (125 MHz, $CDCl_3$) (assignments made with the aid of DEPT-135): δ 165.2 (C=O), 156.5 (C4`), 151.9 (C4), 149.1 (C3), 131.2 (C1`), 127.6 (C1), 122.1 (C2`, 6`), 119.4 (C6), 114.2 (C3`, 5`), 110.7 (C2), 110.3 (C5), 56.0 (2 x OCH_3), 55.5 (OCH_3).

3.24. *N*-(4-Fluorophenyl)-3,4-dimethoxybenzamide

Reaction mixture was purified using basic activated alumina column chromatography eluting with 1% MeOH in DCM to give a white solid (0.482 g, 97%), m.p. = 190-192 ^{o}C. IR (cm^{-1}): 3289 (NH), 3020 (ArCH), 2846 (CH_3), 1638 (C=O), 1500, 1017, 816, 755; ^{1}H-NMR (300 MHz, DMSO-d_6): δ 10.14 (1H, br s, NH), 7.78 (2H, dd, J_1 = 9.0 Hz, J_2 = 5.1Hz, H2`, 6`), 7.62 (1H, dd, $^{2}J_1$ = 8.4 Hz, J_2 = 1.8Hz, H6), 7.53 (1H, d, J = 1.8 Hz, H2), 7.19 (2H, t, J 8.9 Hz, H3`, 5`), 7.08 (1H, br d, J = 8.4 Hz, H5), 3.842 (3H, s, OCH_3), 3.835 (3H, s, OCH_3); ^{13}C-NMR (75 MHz, DMSO-d_6) (assignments made with the aid of DEPT-135): δ 164.8 (C=O), 158.2 (d, J = 238.5 Hz, C4`), 151.6 (C4), 148.3 (C3), 135.6 (d, J = 2.6 Hz, C1`), 126.8 (C1), 122.2 (d, J = 7.8 Hz, C2`, 6`), 121.0 (C6), 115.1 (d, J = 22.0 Hz, C3`, 5`), 111.0 (C2), 110.9 (C5), 55.64 (OCH_3), 55.61 (OCH_3); ^{19}F-NMR (^{1}H-decoupled, 376 MHz, DMSO-d_6): δ − 119.2 (s, F); ^{19}F-NMR (^{1}H-coupled, 376 MHz, DMSO-d_6): δ − 119.4 − 119.5 (m, F).

3.25. *N*-(4-Iodophenyl)-3,4-dimethoxybenzamide

Reaction mixture was purified using basic activated alumina column chromatography eluting with 1% MeOH in DCM to give a white solid (0.320 g, 92%), m.p. = 203-206 ^{o}C. IR (cm^{-1}); 3296 (NH), 2933 (ArCH), 2838 (CH), 1649 (C=O), 1508, 1019, 814; ^{1}H-NMR (300 MHz, DMSO-d_6): δ 10.15 (1H, br s, NH), 7.69 (2H, br d, J = 8.7 Hz, H2`, 6`), 7.61 (3H, br d, J = 9.0 Hz, H3`, 5`, 6), 7.51 (1H, d, J = 1.5 Hz, H2), 7.08 (1H, d, J = 8.4 Hz, H5), 3.84 (6H, s, 2 x OCH_3); ^{13}C-NMR (125 MHz, DMSO-d_6) (assignments made with the aid of DEPT-135): δ 165.0 (C=O), 151.7 (C4), 148.3 (C3), 139.2 (C1`), 137.2 (C3`,5`), 126.7 (C1), 122.5 (C2`, 6`), 121.1 (C6), 111.0 (C2), 110.9 (C5), 87.1 (C4`), 55.7 (OCH_3), 55.6 (OCH_3).

3.26. ***N*-(4-Bromophenyl)-3,4-dimethoxybenzamide**

Reaction mixture was purified using basic activated alumina column chromatography eluting with 2% MeOH in DCM to give a white solid (0.350 g, 82%), m.p. = 211-212 ^{o}C. IR (cm^{-1}): 3303 (NH), 3936 (ArCH), 2842 (CH_3), 1649 (C=O), 1583, 1408, 1270, 1018, 772; ^{1}H-NMR (300 MHz, DMSO-d_6): δ 10.20 (1H, br s, NH), 7.75 (2H, br d, J = 8.7 Hz, H 2`, 6`), 7.62 (1H, dd, J_1 = 8.4 Hz, J_2 = 1.8 Hz, H6), 7.53 (2H, br d, J = 7.2 Hz, H3`, 5`), 7.52 (1H, d, J = 1.8 Hz, H2), 7.09 (1H, br d, J = 8.4 Hz, H5), 3.84 (6H, s, 2 x OCH_3); ^{13}C-NMR (125 MHz, DMSO-d_6) (assignments made with the aid of DEPT-135): δ 165.0 (C=O), 151.7 (C3), 148.3 (C4), 138.6 (C1`), 131.2 (C3`, 5`), 126.7 (C1), 122.3 (C2`, 6`), 121.1 (C6), 115.1 (C4`), 111.1 (C2), 110.7 (C5), 55.6 (2 x OCH_3).

3.27. 3,4-Dimethoxy-*N*-(4-(trifluoromethyl)phenyl)benzamide

Reaction mixture was purified using basic activated alumina column chromatography eluting with 2% MeOH in DCM to give a yellow solid (0.402 g, 100%), m.p. = 208-209 ^{o}C. IR (cm^{-1}): 3302 (NH), 3021 (ArCH), 2846 (CH_3), 1647 (C=O), 1504, 1111, 827; ^{1}H-NMR (300 MHz, DMSO-d_6): δ 10.40 (1H, br s, NH), 8.0 (2H, br d, J = 8.4 Hz, H3`, 5`), 7.72 (2H, br d, J = 8.7 Hz, H2`, 6`), 7.65 (1H, dd, J_1 = 8.7 Hz, J_2 = 1.7 Hz, H6), 7.54 (1H, d, J = 1.2 Hz, H2), 7.11 (1H, d, J = 8.4 Hz, H5), 3.85 (6H, s, 2 x OCH_3); ^{13}C-NMR(125 MHz, DMSO-d_6) (assignments made with the aid of DEPT-135): δ 165.4 (C=O), 152.0 (C4), 148.4 (C3), 143.0 (C1`), 126.7 (C1), 125.9 (q, J = 3.5 Hz, C3`, 5`), 124.4 (q, J = 269.9 Hz, $CF_3$), 123.4 (q, J = 31.8 Hz, C4`), 121.3 (C6), 120.1 (C2`, 6`), 111.1 (C2), 110.9 (C5), 55.7 (OCH_3), 55.6 (OCH_3); ^{19}F-NMR (^{1}H-decoupled, 376 MHz, DMSO-d_6): δ − 65.6 (s, CF_3).

3.28. ***N*-(4-Chlorophenyl)-3,4-dimethoxybenzamide**

Reaction mixture was purified using basic activated alumina column chromatography eluting with 1% MeOH in DCM to give a white solid (0.310 g, 66%), m.p. = 200-201 ^{o}C. IR (cm^{-1}): 3298 (NH), 3014 (ArCH), 2846 (CH_3), 1639 (C=O), 1507, 1010, 868; ^{1}H-NMR (300 MHz, DMSO-d_6): δ 10.2 (1H, br s, NH), 7.80 (2H, br d, J = 9.0 Hz, H2`, 6`), 7.62 (1H, dd, $^{3}J_1$ = 8.4 Hz, J_2 = 1.8 Hz, H6), 7.52 (1H, d, J = 1.5 Hz, H2), 7.40 (2H, br d, J = 9.0 Hz, H3`, 5`), 7.09 (1H, d, J = 8.4 Hz, H5), 3.84 (6H, s, 2 x OCH_3); ^{13}C-NMR (75 MHz, DMSO-d_6) (assignments made with the aid of DEPT-135): δ 165.0 (C=O), 151.7 (C4), 148.3 (C3), 138.2 (C1`), 128.4 (C3`, 5`), 127.0 (C4`), 126.7 (C1), 121.9 (C2`, 6`), 121.1 (C6), 111.1 (C2), 110.9 (C5), 55.7 (OCH_3), 55.6 (OCH_3).

3.29. *N*-(4-Cyanophenyl)-3,4-dimethoxybenzamide

Reaction mixture was purified using basic activated alumina column chromatography eluting with 2% MeOH in DCM to give a white solid (0.410 g, 86%), m.p. = 207-208 ^{o}C. IR (cm^{-1}): 3330 (NH), 2940 (ArCH), 2833 (CH_3), 2228 (CN), 1645 (C=O), 1512, 1014, 830; ^{1}H-NMR (300 MHz, DMSO-d_6): δ 10.48 (1H, br s, NH), 7.99 (2H, br d, J = 8.7 Hz, H2`, 6`), 7.80 (2H, br d, J = 8.7 Hz, H3`, 5`), 7.64 (1H, dd, J_1 = 8.6 Hz, J_2 = 1.4Hz, H6), 7.53 (1H, d, J = 1.2 Hz, H2), 7.10 (1H, d, J = 8.7 Hz, H5), 3.84 (6H, s, 2 x OCH_3); ^{13}C-NMR (75 MHz, DMSO-d_6) (assignments made with the aid of DEPT-135): δ 165.5 (C=O), 152.1 (C4), 148.4 (C3), 143.7 (C1`), 133.0 (C3`, 5`), 126.3 (C1), 121.4 (C6), 120.2 (C2`, 6`), 119.1 (CN), 111.2 (C2), 110.9 (C5), 105.1 (C4`), 55.71 (OCH_3), 55.67 (OCH_3).

3.30. 3,4-Dimethoxy-*N*-(4-nitrophenyl)benzamide

Reaction mixture was purified using basic activated alumina column chromatography eluting with 2% MeOH in DCM to give a yellow solid (0.420 g, 96%), m.p. = 176-179 ^{o}C. IR (cm^{-1}): 3329 (NH), 2945 (ArCH), 2844 (CH_3), 1647 (C=O), 1593, 1012, 883; ^{1}H-NMR (300 MHz, DMSO-d_6): δ 10.66 (1H, br s, NH), 8.25 (2H, br d, ^{2}J = 9.0 Hz, H3`, 5`), 8.06 (2H, br d, J = 9.0 Hz, H2`, 6`), 7.67 (1H, br d, J = 8.4 Hz, H6), 7.56 (1H, br s, H2), 7.11 (1H, br d, J = 8.4 Hz, H5), 3.85 (6H, s, 2 x OCH_3); ^{13}C-NMR (75 MHz, DMSO-d6) (assignments made with the aid of DEPT-135): δ 165.6 (C=O), 152.2 (C4), 148.4 (C3), 145.7 (C1`), 142.3 (C4`), 126.1 (C1), 124.7 (C3`, 5`), 121.6 (C6), 119.8 (C2`, 6`), 111.3 (C2), 111.0 (C5), 55.73 (OCH_3), 55.70 (OCH_3).

3.31. 3,4,5-Trimethoxy-*N*-(4-methoxyphenyl)benzamide

Reaction mixture was purified using basic activated alumina column chromatography eluting with 100% DCM to give an off white solid (0.395 g, 78%), m.p. = 160-163 ^{o}C, lit (160-162 ^{o}C) [4]. IR (cm^{-1}): 3214 (NH), 2935 (ArCH), 2834 (CH_3), 1639 (C=O), 1581, 1233, 998; ^{1}H-NMR (300 MHz, DMSO-d_6): δ 10.02 (1H, br s, NH), 7.62 (2H, br d, J = 8.4 Hz, H2`, 6`), 7.27 (2H, br s, H2, 6), 6.94 (2H, br d, J = 8.7 Hz, H3`, 5`), 3.86 (6H, s, OCH_3), 3.75 (3H, s, OCH_3), 3.73 (s, 3H, OCH_3); ^{13}C-NMR (125 MHz, DMSO-d_6) (assignments made with the aid of DEPT-135): δ 164.4 (C=O), 155.5 (C4`), 152.5 (C3, 5), 140.4 (C4), 131.9 (C1`), 130.0 (C1), 122.2 (C2`, 6`), 113.6 (C3`, 5`), 105.0 (C2, 6), 60.0 (OCH_3), 56.0 (2 x OCH_3), 55.1 (OCH_3).

3.32. *N*-(4-Fluorophenyl)-3,4,5-trimethoxybenzamide

Reaction mixture was purified using basic activated alumina column chromatography eluting with 1% MeOH in DCM to give a yellow solid (0.402 g, 73%), m.p. = 170-173 ^{o}C. IR (cm^{-1}):

3212 (NH), 2941 (ArCH), 2830 (CH_3), 1637 (C=O), 1542, 1405; ^{1}H-NMR (300 MHz, DMSO-d_6): δ 10.26 (1H, br s, NH), 7.78 (2H, dd, J = 8.7 Hz, J = 5.1 Hz, H2`, 6`), 7.30 (H2, br s, 2H, 6), 7.20 (2H, t, J = 8.9 Hz, H3`, 5`), 3.87 (6H, s, 2 x OCH_3), 3.73 (3H, s, OCH_3); ^{13}C-NMR (125 MHz, DMSO-d_6) (assignments made with the aid of DEPT-135): δ 164.9 (C=O), 158.4 (d, J = 239.0 Hz, C4`), 152.7 (C3, 5), 140.3 (C4), 135.4 (d, J = 2.4 Hz, C1`), 129.9 (C1), 122.5 (d, J = 7.9 Hz, C2`,6`), 115.2 (d, J = 21.9 Hz, C3`, 5`), 105.3 (C2, 6), 60.1 (OCH_3), 56.1 (2 x OCH_3); ^{19}F-NMR (^{1}H-decoupled, 376 MHz, DMSO-d_6): δ − 121.2 (s, F); ^{19}F-NMR (^{1}H-coupled, 376 MHz, DMSO-d_6): δ − 121.20 − 120.21 (m, F).

3.33. *N*-(4-Iodophenyl)-3,4,5-trimethoxybenzamide

Reaction mixture was eluted through activated basic alumina column using 1% MeOH in DCM to give a white solid (0.250 g, 66%), m.p. = 144-147 ^{o}C. IR (cm^{-1}): 3277 (NH), 1650 (C=O), 1583,1325; ^{1}H-NMR (300 MHz, $CDCl_3$): δ 8.24 (1H, br s, NH), 7.54 (2H, br d, J = 7.2 Hz, H2`, 6`), 7.34 (2H, br d, J = 7.5 Hz, H3`, 5`), 6.94 (2H, br s, H2, 6), 3.80 (s, 3H, OCH_3), 3.74 (6H, s, 2 x OCH_3); ^{13}C-NMR (125 MHz, $CDCl_3$) (assignments made with the aid of DEPT-135): δ 165.8 (C=O), 153.1 (C3, 5), 141.0 (C4), 137.8 (C3`, 5`), 137.7 (C1`), 128.9 (C1), 122.2 (C2`, 6`), 104.4 (C2, 6), 87.7 (C4`), 60.8 (OCH_3), 56.1 (2 x OCH_3).

3.34. *N*-(4-Bromophenyl)-3,4,5-trimethoxybenzamide

Reaction mixture was purified using basic activated alumina column chromatography eluting with 1% MeOH in DCM to give a white solid (0.390 g, 92%), m.p. = 177-178 ^{o}C. IR (cm^{-1}): 3335 (NH), 3001 (ArCH), 2848 (CH_3), 1661 (C=O), 1604, 1587, 815; ^{1}H-NMR (300 MHz, DMSO-d_6): δ 10.26 (1H, br s, NH), 7.73 (2H, br d, J = 8.7 Hz, H2`, 6`), 7.55 (2H, br d, J = 8.7 Hz, H3`, 5`), 7.27 (2H, br s, H2, 6), 3.87 (6H, s, 2 x OCH_3), 3.73 (3H, s, OCH_3); ^{13}C-NMR (125 MHz, DMSO-d_6) (assignments made with the aid of DEPT-135): δ 165.0 (C=O), 152.6 (C3, 5), 140.4 (C4), 138.5 (C1`), 131.4 (C3`, 5`), 129.8 (C1), 122.5 (C2`, 6`), 115.4 (C4`), 105.3 (C2, 6), 60.1 (OCH_3), 56.1 (2 x OCH_3).

3.35. 3,4,5-Trimethoxy-*N*-(4-(trifluoromethyl)phenyl)benzamide

Reaction mixture was purified using basic activated alumina column chromatography eluting with DCM to give a white solid (0.390 g, 88%), m.p. = 182-184 ^{o}C. IR (cm^{-1}): 3347 (NH), 2992 (ArCH), 2837 (CH_3), 1666 (C=O), 1127, 830; ^{1}H-NMR (300 MHz, DMSO-d_6): δ 10.49 (1H, br s, NH), 8.0 (2H, br d, J = 8.4 Hz, H3`, 5`), 7.73 (2H, br d, J = 8.7 Hz, H2`, 6`), 7.30 (2H, br s, H2, 6), 3.88 (6H, s, 2 x OCH_3), 3.74 (s, 3H, OCH_3); ^{13}C-NMR (125 MHz, DMSO-d_6) (assignments made with the aid of DEPT-135): δ 165.4 (C=O), 152.7 (C3, 5), 142.8 (C4), 140.6

(C1`), 129.6 (C1), 125.9 (q, J = 3.6 Hz, C3`, 5`), 124.4 (q, J = 269.7 Hz, $CF_3$), 123.7 (q, J = 31.7 Hz, C4`), 120.3 (2`, 6`), 105.5 (C2, 6), 60.2 (OCH_3), 56.2 (2 x OCH_3); ^{19}F-NMR (^{1}H-decoupled, 376 MHz, DMSO-d_6): δ − 62.7 (s, CF_3).

3.36. *N*-(4-Chlorophenyl)-3,4,5-trimethoxybenzamide

Reaction mixture was purified using basic activated alumina column chromatography eluting with 1% MeOH in DCM to give a white solid (0.350 g, 69%), m.p. = 183-184 °C. IR (cm^{-1}): 3269 (NH), 2938 (ArCH), 2848 (CH_3), 1645 (C=O), 1584, 1530, 1120, 826; ^{1}H-NMR (300 MHz, DMSO-d_6): δ 10.30 (1H, br s, NH), 7.80 (2H, br d, J = 9.0 Hz, H2`, 6`), 7.42 (2H, br d, J = 8.7 Hz, 3`, 5`), 7.28 (2H, br s, H2, 6), 3.87 (6H, s, 2 x OCH_3), 3.73 (3H, s, OCH_3); ^{13}C-NMR (125 MHz, DMSO-d_6) (assignments made with the aid of DEPT-135): δ 165.0 (C=O), 152.7 (C3, 5), 140.4 (C4), 138.1 (C1`), 129.8 (C4`), 128.5 (C3`, 5`), 127.3 (C1), 122.1 (C2`, 6`), 105.4 (C2, 6), 60.1 (OCH_3), 56.1 (2 x OCH_3).

3.37. *N*-(4-Cyanophenyl)-3,4,5-trimethoxybenzamide

Reaction mixture was purified using basic activated alumina column chromatography eluting with 1% MeOH in DCM to give a white solid (0.471 g, 89%), m.p. = 140-142 °C. IR (cm^{-1}): 3302 (NH), 2222 (CN), 1649 (C=O), 1124, 858; ^{1}H-NMR (300 MHz, DMSO-d_6): δ 10.57 (1H, br s, NH), 7.98 (2H, br d, J = 8.7 Hz, H2`, 6`), 7.83 (2H, br d, J = 8.7 Hz, H3`, 5`), 7.29 (2H, br s, H2, 6), 3.87 (6H, s, 2 x OCH_3), 3.73 (3H, s, OCH_3); ^{13}C-NMR (125 MHz, DMSO-d_6) (assignments made with the aid of DEPT-135): δ 165.6 (C=O), 152.7 (C3, 5), 143.5 (C4), 140.7 (C1`), 133.1 (C3`, 5`), 129.5 (C1), 120.4 (C2`, 6`), 119.1 (CN), 105.5 (C2, 6), 105.4 (C4`), 60.2 (OCH_3), 56.2 (2 x OCH_3).

3.38. 3,4,5-Trimethoxy-*N*-(4-nitrophenyl)benzamide

Reaction mixture was purified using basic activated alumina column chromatography eluting with 1% MeOH in DCM to give a yellow solid (0.475 g, 99%), m.p. = 190-195 °C. IR (cm^{-1}): 3404 (NH), 3011 (ArCH), 2845 (CH_3), 1684 (C=O), 1509, 1124, 858; ^{1}H-NMR (300 MHz, DMSO-d_6): δ 10.74 (1H, br s, NH), 8.28 (2H, br d, J = 9.0 Hz, H3`, 5`), 8.06 (2H, br d, J = 9.3 Hz, H2`, 6`), 7.31 (2H, br s, H2, 6), 3.88 (6H, s, 2 x OCH_3), 3.74 (3H, s, OCH_3); ^{13}C-NMR (125 MHz, DMSO-d_6) (assignments made with the aid of DEPT-135): δ 165.7 (C=O), 152.7 (C3, 5), 145.5 (C1`), 142.5 (C4`), 140.8 (C4), 129.3 (C1), 124.8 (C3`, 5`), 120.0 (C2`, 6`), 105.6 (C2, 6), 60.2 (OCH_3), 56.2 (2 x OCH_3).

4. General procedure for the synthesis of amides C

Appropriate anilines (2-chloro-4-nitroaniline, 4-fluoroaniline) (1.0 mmol), appropriate benzoic acids (3,5-dimethoxybenzoic acid, 3,4-drimethoxybenzoic acid, 3,4,5-trimethoxybenzoic acid, 3-methoxybenzoic acid, 4-methoxybenzoic acid, 3-fluorobenzoic acid, 4-fluorobenzoic acid) (1.2 mmol), and PCl_3 (1.0 mmol) in dry acetonitrile (15.0ml) were heated in a microwave at 150 ^{o}C for 5 minutes. The reaction vials were cooled down. The precipitated solids were filtered, washed with water, and dried.

4.1. *N*-(2-Chloro-4-nitrophenyl)-3,5-dimethoxybenzamide

Obtained as a yellow solid (1.0 g, 61%), m.p. = 176-177 ^{o}C. IR (cm^{-1}): 3311 (NH), 2949 (ArCH), 2844 (CH_3), 1656 (C=O), 1591, 837; ^{1}H-NMR (300 MHz, DMSO-d_6): δ 10.27 (1H, br s, NH), 8.41 (1H, d, J = 2.1 Hz, H3`), 8.26 (1H, dd, $J_1$ = 8.9 Hz, $J_2$ = 2.0 Hz, H5`), 8.00 (1H, d, J = 8.7 Hz, H6`), 7.15 (2H, d, J = 1.5 Hz, H2, 6), 6.77 (1H, t, J = 2.0 Hz, H4), 3.83 (6H, s, 2 x $OCH_3$); $^{13}$C-NMR (125 MHz, DMSO-$d_6$) (assignments made with the aid of DEPT-135): δ 165.1 (C=O), 160.5 (C3, 5), 144.7 (C4`), 141.3 (C1`), 135.4 (C1), 128.5 (C2`), 127.1 (C3`), 124.9 (C6`), 122.9 (C5`), 105.8 (C2, 6), 104.1 (C4), 55.6 (2 x OCH_3).

4.2. *N*-(2-Chloro-4-nitrophenyl)-3,4-dimethoxybenzamide

Obtained as a yellow solid (1.55 g, 87 %), m.p. = 169-170 ^{o}C. IR (cm^{-1}): 3410 (NH), 2937 (ArCH), 2841 (CH_3), 1681 (C=O), 1583, 751; ^{1}H-NMR (300 MHz, $CDCl_3$): δ 8.83 (1H, d, $^3J_{HH}$ = 9.3 Hz, H6`), 8.66 (1H, br s, NH), 8.33 (1H, d, J = 2.4 Hz, H3`), 8.21 (1H, dd, J_1 = 9.3 Hz, J_2 = 2.4 Hz, H5`), 7.52 (1H, d, J = 1.8 Hz, H2), 7.45 (1H, dd, $J_1$ = 8.4 Hz, $J_2$ = 1.8 Hz, H6), 6.96 (1H, d, J = 8.1 Hz, H5), 3.97 (3H, s, $OCH_3$), 3.96 (3H, s, $OCH_3$); $^{13}$C-NMR (125 MHz, $CDCl_3$) (assignments made with the aid of DEPT-135): δ 164.8 (C=O), 153.0 (C4), 149.5 (C3), 142.8 (C4`), 140.6 (C1`), 126.0 (C2`), 124.7 (C3`), 123.8 (C6`), 122.3 (C1), 119.9 (C5`), 119.7 (C6), 110.7 (C2), 110.5 (C5), 56.13 (OCH_3), 56.09 (OCH_3).

4.3. *N*-(2-chloro-4-nitrophenyl)-3,4,5-trimethoxybenzamide

Obtained as a yellow solid (1.52 g, 90%), m.p. = 197 ^{o}C. IR (cm^{-1}): 3413 (NH), 2938 (ArCH), 2835 (CH_3), 1694 (C=O), 1586, 741; ^{1}H-NMR (300 MHz, DMSO-d_6): δ 10.24 (1H, br s, NH), 8.42 (1H, d, J = 2.1 Hz, H3`), 8.27 (1H, dd, $J_1$ = 8.9 Hz, $J_2$ = 2.0Hz, H5`), 7.98 (1H, d, J = 9.0 Hz, H6`), 7.34 (2H, br s, H2, 6), 3.87 (6H, s, 2 x $OCH_3$), 3.75 (3H, s, $OCH_3$); $^{13}$C-NMR (125 MHz, DMSO-$d_6$) (assignments made with the aid of DEPT-135): δ 164.9 (C=O), 152.8 (C3, 5), 144.7 (C1`), 141.4 (C4`), 140.9 (C4), 128.5 (C1`), 128.4 (C2`), 127.3 (C5`), 125.0 (C3`), 122.9 (6`), 105.5 (C2, 6), 60.2 (OCH_3), 56.1 (2 x OCH_3).

4.4. *N*-(4-Fluorophenyl)-3-methoxybenzamide

The precipitated solid was filtered, washed with water, and dried as a white solid (1.17 g, 66%). IR (cm^{-1}): 3321 (NH), 2938 (ArCH), 2835 (CH_3), 1651 (C=O), 1508, 914; ^{1}H-NMR (300 MHz, $CDCl_3$): δ 8.04 (1H, br s, NH), 7.60 (2H, dd, J_1 = 9.0 Hz, J_2 = 4.8 Hz, H2`, 6`), 7.43-7.31 (3H, m, H4, 5, 6), 7.10-7.6.99 (3H, m, H2, 3`, 5`), 3.84 (3H, s, OCH_3); ^{13}C-NMR (125 MHz, $CDCl_3$) (assignments made with the aid of DEPT-135): δ 165.7 (C=O), 159.9 (C3), 159.5 (d, J = 242.6 Hz, C4`), 136.1 (C1), 133.9 (d, J = 2.9 Hz, C1`), 129.7 (C5), 122.1 (d, J = 7.8 Hz, C2`, 6`), 118.7 (C6), 118.0 (C4), 115.6 (d, J = 22.4 Hz, C3`, 5`), 112.5 (C2), 55.4 (OCH_3); ^{19}F-NMR (^{1}H-decoupled, 376 MHz, $CDCl_3$): δ − 120.7 (s, F).

4.5. *N*-(4-Fluorophenyl)-4-methoxybenzamide

Filtered through a 5 cm layer of activated basic alumina and was obtained as a pale-yellow solid (1.3 g, 74%). IR (cm^{-1}): 3334 (NH), 2970 (ArCH), 2841 (CH_3), 1651 (C=O), 1605, 928; ^{1}H-NMR (300 MHz, DMSO-d_6): δ 10.14 (1H, br s, NH), 7.95 (2H, d, J = 8.7 Hz, H2, 6), 7.79 (2H, dd, J_1 = 9.0 Hz, J_2 = 5.1Hz, H2`, 6`), 7.18 (2H, t, J = 8.9 Hz, H3`, 5`), 7.06 (2H, d, J = 8.7 Hz, H3, 5), 3.82 (3H, s, OCH_3); ^{13}C-NMR (125 MHz, DMSO-d_6) (assignments made with the aid of DEPT-135): δ 162.8 (C=O), 158.2 (d, J = 238.3 Hz, C4`), 135.7 (d, J = 2.1 Hz, C1`), 131.3 (C2, 6), 129.6 (C1), 122.1 (d, J = 7.8 Hz, C2`, 6`), 115.1 (d, J = 21.9 Hz, C3`, 5`), 113.8 (C3, 5), 55.4 (OCH_3); ^{19}F-NMR (^{1}H-decoupled, 376 MHz, DMSO-d_6): − 121.6 (s, F).

4.6. 3-Fluoro-*N*-(4-fluorophenyl)benzamide

The precipitated solid was filtered, washed with water, and dried as a pale-yellow solid (1.2 g, 72%), m.p. = 140-141 ^oC. IR (cm^{-1}): 3333 (NH), 3085 (ArCH), 1640 (C=O), 800; ^{1}H-NMR (300 MHz, DMSO-d_6): δ 10.38 (1H, br s, NH), 8.84-8.75 (4H, m, H2, 6, 2`, 6`), 7.58 (1H, td, J_1 = 8.0 Hz, J_2 = 6.0 Hz, H5), 7.44 (1H, td, J_1 = 8.5 Hz, J_2 = 2.2Hz, H4), 7.20 (2H, t, J = 9.0 Hz, H3`, 5`), ^{13}C-NMR (125 MHz, DMSO-d_6) (assignments made with the aid of DEPT-135): δ 164.1 (d, J = 2.1 Hz, C=O), 162.0 (d, J = 243.0 Hz, C3), 158.4 (d, J = 239.1 Hz, C4`), 137.1 (d, J = 6.8 Hz, C1), 135.3 (d, J = 2.6 Hz, C1`), 130.6 (d, J = 8.0 Hz, C5), 123.9 (d, J = 2.4 Hz, C6), 122.3 (d, J = 7.8 Hz, C2`, 6`), 118.5 (d, J = 21.1 Hz, C4), 115.2 (d, J = 21.9 Hz, C3`, 5`), 114.5 (d, J = 22.6 Hz, C2); ^{19}F-NMR (^{1}H-decoupled, 376 MHz, DMSO-d_6): δ − 114.9 (s, F at C3), − 120.9 (s, F at C4`).

4.7. 4-Fluoro-*N*-(4-fluorophenyl)benzamide

Filtered through a 5 cm layer of activated basic alumina and was obtained as a pale-yellow solid (1.25 g, 75%), m.p. = 186-188 ^oC. IR (cm^{-1}): 3360 (NH), 1649 (C=O), 1601, 760; ^{1}H-

NMR (300 MHz, DMSO-d_6): δ 10.32 (1H, br s, NH), 8.04 (2H, dd, J_1 = 8.7 Hz, J_2 = 5.4Hz, H2, 6), 7.79 (2H, dd, J_1 = 9.0 Hz, J_2 = 5.1Hz, H2`, 6`), 7.37 (2H, t, J = 8.9 Hz, H3, 5), 7.19 (2H, t, J = 9.0 Hz, H3`, 5`); ^{13}C-NMR (125 MHz, DMSO-d_6) (assignments made with the aid of DEPT-135): δ 164.4 (C=O), 164.1 (d, J = 247.6 Hz, C4), 158.3 (d, J = 238.6 Hz, C4`), 135.4 (d, J = 2.1 Hz, C1`), 131.2 (d, J = 2.8 Hz, C1), 130.4 (d, J = 9.0 Hz, C2, 6), 122.2 (d, J = 7.8Hz, C2`, 6`), 115.4 (d, J = 18.9 Hz, C3`, 5`), 115.2 (d, J = 19.3Hz, C3, 5); ^{19}F-NMR (^{1}H-decoupled, 376 MHz, DMSO-d_6): δ − 111.1 (s, F at C4), − 121.1 (s, F at C4`).

5. General procedure for synthesis of phenylbenzothioamide

Substituted-phenylbenzamide (1.0 mmol) and Lawesson's reagent (0.6 mmol) in dry toluene (4.0 ml) were heated in a microwave at 150 °C for 5 min. The formation of benzothioamides was identified using TLC (30% ethyl acetate in hexane). The reaction mixture was purified by column chromatography. Details of purification are specified for each compound.

5.1. 3,5-Dimethoxy-*N*-(4-methoxyphenyl)benzothioamide [1]

Purified using silica gel column chromatography eluting with 7% ethyl acetate in hexane to give a yellow solid (0.208 g, 99%), m.p. = 158-159 °C. IR (cm^{-1}): 3142 (NH), 2938 (ArCH), 2838 (CH_3), 1600, 1510, 1060 (C=S), 828; ^{1}H-NMR (300 MHz, $CDCl_3$): δ 8.92 (1H, br s, NH), 7.64 (2H, br d, J = 8.7 Hz, H2`, 6`), 6.98-6.95 (4H, m, H2, 6, 3`, 5`), 6.58 (1H, t, J = 2.1 Hz, H4), 3.85 (9H, s, 3 x OCH_3); ^{13}C-NMR (125 MHz, $CDCl_3$) (assignments made with the aid of DEPT-135): δ 197.8 (C=S), 160.9 (C3, 5), 158.2 (C4`), 145.0 (C1), 131.9 (C1`), 125.5 (C2`, 6`), 114.2 (C3`, 5`), 104.8 (C2, 6), 103.1 (C4), 55.6 (2 x OCH_3), 55.4 (OCH_3).

5.2. *N*-(4-Fluorophenyl)-3,5-dimethoxybenzothioamide

purified using silica gel column chromatography eluting with 5% ethyl acetate in hexane to give a yellow solid (0.204 g, 77%), m.p. = 137-139 °C. IR (cm^{-1}): 3150 (NH), 2959 (ArCH), 2838 (CH_3), 1593, 1505, 1155 (C=S), 834; ^{1}H-NMR (300 MHz, $CDCl_3$): δ 8.91 (1H, br s, NH), 7.63 (2H, dd, J_1 = 8.4 Hz, J_2 = 4.8 Hz, H2`, 6`), 7.05 (2H, t, J = 8.4Hz, H3`, 5`), 6.88 (2H, d, J = 1.5 Hz, H2, 6), 6.50 (1H, t, J = 2.1 Hz, H4), 3.76 (6H, s, 2 x OCH_3); ^{13}C-NMR (125 MHz, $CDCl_3$) (assignments made with the aid of DEPT-135): δ 197.4 (C=S), 159.8 (d, J = 246.0 Hz, C4`), 159.7 (C3, 5), 143.8 (C1), 133.8 (d, J = 2.5 Hz, C1`), 124.9 (d, J = 8.3 Hz, C2`, 6`), 114.8 (d, J = 22.6 Hz, C3`, 5`), 103.8 (C2, 6), 102.2 (C4), 54.6 (2 x OCH_3); ^{19}F-NMR (^{1}H-decupled, 376 MHz, $CDCl_3$): δ − 116.9 (s, F); ^{19}F-NMR (^{1}H-coupled, 376 MHz, $CDCl_3$): δ − 116.9 (tt, J_1 = 8.6 Hz, J_2 = 4.1 Hz, F).

5.3. *N*-(4-Iodophenyl)-3,5-dimethoxybenzothioamide
Purified using silica gel column chromatography eluting with 5% ethyl acetate in hexane to give a yellow solid (0.095 g, 75%), m.p. = 111-113 °C. IR (cm^{-1}): 3146 (NH), 2992 (ArCH), 2831 (CH), 1593, 1510, 1420, 1343, 1150 (C=S), 822; ^{1}H-NMR (300 MHz, $CDCl_3$): δ 8.98 (1H, br s, NH), 7.73 (2H, br d, J = 7.5 Hz, H3`, 5`), 7.55 (2H, br d, J = 6.0 Hz, H2`, 6`), 6.92 (2H, br s, H2, 6), 6.56 (1H, br s, H4), 3.82 (6H, s, 2 x OCH_3); ^{13}C-NMR (125 MHz, $CDCl_3$) (assignments made with the aid of DEPT-135): δ 198.0 (C=S), 160.7 (C3, 5), 145.1 (C1), 138.5 (C1`), 138.0 (C3`, 5`), 125.2 (C2`, 6`), 104.8 (C2, 6), 103.2 (4), 91.0 (C4`), 55.6 (2 x OCH_3).

5.4. *N*-(4-Bromophenyl)-3,5-dimethoxybenzothioamide
Purified using silica gel column chromatography eluting with 7% ethyl acetate in hexane to give a yellow solid (0.190 g, 91%), m.p. = 136-137 °C. IR (cm^{-1}): 3153 (NH), 2963 (ArCH), 2833 (CH_3), 1592, 1512, 1337, 1219, 1090 (C=S), 827; ^{1}H-NMR (300 MHz, $CDCl_3$): δ 8.99 (1H, br s, NH), 7.67 (2H, br d, J = 7.8 Hz, H2`, 6`), 7.54 (2H, br d, J = 8.1 Hz, H3`, 5`), 6.93 (br s, 2H, H2, 6), 6.57 (1H, br s, H4), 3.83 (6H, s, 2 x OCH_3); ^{13}C-NMR (125 MHz, $CDCl_3$) (assignments made with the aid of DEPT-135): δ 198.1 (C=S), 160.7 (C3, 5), 145.0 (C1), 137.8 (C1`), 132.1 (C3`, 5`), 125.1 (C2`, 6`), 119.9 (C4`), 104.8 (C2, 6), 103.3 (C4), 55.6 (2 x OCH_3).

5.5. 3,5-Dimethoxy-*N*-(4-(trifluoromethyl)phenyl)benzothioamide
Purified using silica gel column chromatography eluting with 5% ethyl acetate in hexane to give a yellow solid (0.220 g, 83%), m.p. = 125-127 °C. IR (cm^{-1}): 3159 (NH), 2994 (ArCH), 2839 (CH_3), 1593, 1521, 1466, 1152 (C=S), 836; ^{1}H-NMR (300 MHz, $CDCl_3$): δ 9.08 (1H, br s, NH), 7.94 (2H, br s, H3`, 5`), 7.69 (2H, br d, J = 8.4 Hz, H2`, 6`), 6.94 (2H, br s, H2, 6), 6.59 (1H, t, J = 2.1 Hz, H4), 3.84 (6H, s, 2 x OCH_3); ^{13}C-NMR (125 MHz, $CDCl_3$) (assignments made with the aid of DEPT-135): δ 198.6 (C=S), 160.8 (C3, 5), 141.8 (C1, 1`), 128.4 (q, J = 32.3 Hz, C4`), 126.3 (q, J = 3.6 Hz, C3`, 5`), 123.8 (q, J = 270.2 Hz, CF_3), 123.2 (C2`, 6`), 104.9 (C4), 103.4 (C2, 6), 55.7 (2 x OCH_3); ^{19}F NMR with (^{1}H-decoupled, 376MHz, $CDCl_3$): δ − 65.7 (s, CF_3).

5.6. *N*-(4-Chlorophenyl)-3,5-dimethoxybenzothioamide
Purified using silica gel column chromatography eluting with 5% ethyl acetate in hexane to give a yellow solid (0.180 g, 81%), m.p. = 146-149 °C. IR (cm^{-1}): 3132 (NH), 2954 (ArCH), 2855 (CH_3), 1580, 1486, 1486, 1154 (C=S); ^{1}H-NMR (300 MHz, $CDCl_3$): δ 8.98 (1H, br s, NH), 7.73 (2H, br d, J = 7.8 Hz, H2`, 6`), 7.40 (2H, br d, J = 8.1 Hz, H3`, 5`), 6.94 (2H, br s, H2, 6), 6.57 (1H, br s, H4), 3.84 (6H, s, 2 x OCH_3); ^{13}C-NMR (125 MHz, $CDCl_3$) (assignments

made with the aid of DEPT-135): δ 198.2 (C=S), 160.8 (C3, 5), 145.0 (C1), 137.3 (C1`), 132.1 (C4`), 129.1 (C3`, 5`), 124.9 (C2`, 6`), 104.8 (C2, 6), 103.3 (C4), 55.6 (2 x OCH_3).

5.7. *N*-(4-Cyanophenyl)-3,5-dimethoxybenzothioamide

Purified using silica gel column chromatography eluting 15% ethyl acetate in hexane to give a yellow solid (0.170 g, 64%). IR (cm^{-1}): 3143 (NH), 2969 (ArCH), 2838 (CH_3), 2231(CN), 1592, 1512, 1166 (C=S), 730; ^{1}H-NMR (300 MHz, $CDCl_3$): δ 9.17 (1H, br s, NH), 7.99 (2H, br d, J = 6.9 Hz, H2`, 6`), 7.70 (2H, d, J = 8.7 Hz, H3`, 5`), 6.92 (2H, d, J = 1.8 Hz, H2, 6), 6.59 (1H, t, J = 2.0 Hz, H4), 3.83 (6H, s, 2 x OCH_3); ^{13}C-NMR (75 MHz, $CDCl_3$) (assignments made with the aid of DEPT-135): δ 198.7 (C=S), 160.9 (C3, 5), 145.0 (C1), 142.7 (C1`), 133.1 (C3`, 5`), 123.0 (C2`, 6`), 118.4 (CN), 109.5 (4`), 104.9 (C2, 6), 103.5 (C4), 55.7 (2 x OCH_3).

5.8. 3,5-Dimethoxy-*N*-(4-nitrophenyl)benzothioamide

Purified using silica gel column chromatography eluting with 5% ethyl acetate in hexane to give a yellow solid (**259**) (0.148 g, 94%), m.p. = 179-180 ^{o}C. IR (cm^{-1}): 3267 (NH), 3089 (ArCH), 2842 (CH_3), 1589, 1510, 1160 (C=S), 850, 749; ^{1}H-NMR (300 MHz, DMS0-d_6): 12.09 (1H, br s, NH), 8.31 (2H, d, J = 9.0 Hz, H3`, 5`), 8.21 (2H, br d, J = 8.7 Hz, H2`, 6`), 6.98 (2H, d, J = 1.5 Hz, H2, 6), 6.70 (1H, t, J = 2.1 Hz, H4), 3.81 (6H, s, 2 x OCH_3); ^{13}C-NMR (125 MHz, DMS0-d_6) (assignments made with the aid of DEPT-135): δ 198.5 (C=S), 159.9 (C3, 5), 145.6 (C1), 144.5 (C1`), 144.2 (C4`), 124.2 (C2`, 6`), 123.9 (C3`, 5`), 105.7 (C2, 6), 102.9 (C4), 55.5 (2 x OCH_3).

5.9. 2,4-Dimethoxy-*N*-(4-methoxyphenyl)benzothioamide

Purified using silica gel column chromatography eluting with 5% ethyl acetate in hexane to give a yellow solid (0.148 g, 94%), m.p. = 121-123 ^{o}C. IR (cm^{-1}): 3318 (NH), 2958 (ArCH), 2827 (CH_3), 1596, 1508, 1161, 1162 (C=S), 975; ^{1}H-NMR (300 MHz, $CDCl_3$): δ 10.79 (1H, br s, NH), 8.68 (1H, d, J = 9.0 Hz, br s, H6), 7.59 (2H, br d, J = 8.7 Hz, H2`, 6`), 6.95 (2H, brd, J = 9.0Hz, H3`, 5`), 6.62 (1H, dd, J_1 = 9.0 Hz, J_2 = 2.1Hz, H5), 6.49 (1H, d, J = 2.1 Hz, H3), 3.98 (3H, s, OCH), 3.87 (3H, s, OCH_3), 3.83 (3H, s, OCH_3); ^{13}C-NMR (125 MHz, $CDCl_3$) (assignments made with the aid of DEPT-135): δ 192.9 (C=S), 162.4 (C4), 157.0 (C2), 155.7 (C4`), 136.9 (C6), 131.6 (C1`), 125.2 (C2`, 6`), 119.8 (C1), 112.9 (3`, 5`), 104.7 (C5), 97.4 (C3), 55.3 (OCH_3), 54.6 (OCH_3), 54.4 (OCH_3).

5.10. *N*-(4-Fluorophenyl)-2,4-dimethoxybenzothioamide

Purified using silica gel column chromatography eluting with 8% ethyl acetate in hexane to give a yellow solid (0.145 g, 92%). IR (cm^{-1}): 3301 (NH), 2939 (ArCH), 2840 (CH_3), 1596,

1564, 1504, 1191(C=S), 929; ^{1}H-NMR (300 MHz, $CDCl_3$): δ 10.85 (1H, br s, NH), 8.68 (1H, d, J = 9.0 Hz, H6), 7.66 (2H, dd, J_1 = 8.7 Hz, J_2 = 4.8 Hz, H2`, 6`), 7.11 (2H, t, J = 8.7 Hz, H3`, 5`), 6.63 (1H, dd, J_1 = 9.0 Hz, J_2 = 2.4Hz, H5), 6.49 (1H, d, J = 2.1 Hz, H3), 3.99 (3H, s, OCH_3), 3.87 (3H, s, OCH_3); ^{13}C-NMR (75 MHz, $CDCl_3$) (assignments made with the aid of DEPT-135): δ 194.5 (C=S), 163.7 (C4), 160.7 (d, J = 245.1 Hz, C4`), 156.7 (C2), 138.0 (C6), 135.6 (d, J = 3.0 Hz, C1`), 126.7 (d, J = 8.3 Hz, C2`, 6`), 120.7 (C1), 115.6 (d, J = 22.6 Hz, C3`, 5`), 105.8 (C5), 98.5 (C3), 56.3 (OCH_3), 55.6 (OCH_3); ^{19}F-NMR (^{1}H-decoupled, 376 MHz, $CDCl_3$): δ − 117.8 (s, F); ^{19}F-NMR (^{1}H-coupled, 376 MHz, $CDCl_3$): δ − 117.8 − 117.9 (m, F).

5.11. *N*-(4-Iodophenyl)-2,4-dimethoxybenzothioamide

Purified using silica gel column chromatography eluting with 5% ethyl acetate in hexane to give a yellow solid (0.157 g, 100%). IR (cm^{-1}): 3297 (NH), 2924 (ArCH), 2835 (CH_3), 1602, 1568, 1497, 1177 (C=S), 922, 720; ^{1}H-NMR (300 MHz, $CDCl_3$): δ 10.88 (1H, br s, NH), 8.64 (1H, d, J = 9.0 Hz, H6), 7.72 (2H, br d, J = 8.4 Hz, H3`, 5`), 7.53 (2H, br d, J = 8.7 Hz, H2`, 6`), 6.62 (1H, dd, J_1 = 9.2 Hz, J_2 = 2.3 Hz, H5), 6.48 (1H, d, J = 2.1 Hz, H3), 3.98 (3H, s, OCH_3), 3.87 (3H, s, OCH_3); ^{13}C-NMR (75 MHz, $CDCl_3$) (assignments made with the aid of DEPT-135): δ 194.0 (C=S), 164.0 (C4), 156.6 (C2), 139.3 (C1`), 138.0 (C6), 137.8 (C3`, 5`), 126.3 (C2`, 6`), 120.9 (C1), 105.9 (C5), 98.5 (C3), 90.7 (C4`), 56.4 (OCH_3), 55.6 (OCH_3).

5.12. *N*-(4-Bromophenyl)-2,4-dimethoxybenzothioamide

Purified using silica gel column chromatography eluting with 8% ethyl acetate in hexane to give a yellow solid (0.148 g, 95%), m.p. = 122-124 ^{o}C. IR (cm^{-1}): 3236 (NH), 2963 (ArCH), 2830 (CH_3), 1599, 1488, 1160 (C=S), 824; ^{1}H-NMR (300 MHz, $CDCl_3$): δ 10.89 (1H, br s, NH), 8.66 (1H, d, $^3J_{HH}$ = 8.7 Hz, H6), 7.65 (2H, br d, $^3J_{HH}$ = 8.7 Hz, H2`, 6`), 7.53 (2H, br d, $^3J_{HH}$ = 8.7 Hz, H3`, 5`), 6.63 (1H, dd, $^3J_{HH}$ = 9.0 Hz, $^4J_{HH}$ = 2.4 Hz, H5), 6.50 (1H, d, $^4J_{HH}$ = 2.4 Hz, H3), 4.0 (3H, s, OCH_3), 3.87 (3H, s, OCH_3); ^{13}C-NMR (75 MHz, $CDCl_3$) (assignments made with the aid of DEPT-135): δ 194.1 (C=S), 163.7 (C4), 156.6 (C2), 138.6 (C1`), 138.0 (C6), 131.8 (C3`, 5`), 126.1 (C2`, 6`), 120.9 (C4`), 119.6 (C1`), 105.9 (C5), 98.6 (C3), 56.4 (OCH_3), 55.6 (OCH_3).

5.13. 2,4-Dimethoxy-*N*-(4-(trifluoromethyl)phenyl)benzothioamide

Purified using silica gel column chromatography eluting with 5% ethyl acetate in hexane to give a yellow solid (0.185 g, 89%), m.p. = 130-131 ^{o}C. IR (cm^{-1}): 3298 (NH), 2943 (ArCH), 2846 (CH_3), 1600, 1561, 1466, 1318, 1198 (C=S), 836; ^{1}H-NMR (300 MHz, $CDCl_3$): δ 11.05

(1H, br s, NH), 8.65 (1H, d, J = 9.0 Hz, H6), 7.93 (2H, br d, J = 8.4 Hz, H3`, 5`), 7.67 (2H, br d, J = 8.1 Hz, H2`, 6`), 6.64 (1H, dd, J_1 = 9.0 Hz, J_2 = 2.1 Hz, H5), 6.50 (1H, d, J = 1.5 Hz, H3), 4.01 (3H, s, OCH_3), 3.88 (3H, s, OCH_3); ^{13}C-NMR (125 MHz, $CDCl_3$) (assignments made with the aid of DEPT-135): δ 194.4 (C=S), 163.9 (C4), 156.6 (C2), 142.5 (C1`), 138.1 (C6), 127.8 (q, J = 32.8 Hz, C4`), 125.9 (q, J = 3.6Hz, C3`, 5`), 124.3 (2`, 6`), 123.9 (q, J = 270.4 Hz, CF_3), 121.0 (C1), 106.0 (C5), 98.6 (C3), 56.4 (OCH_3), 55.7 (OCH_3); ^{19}F-NMR (^{1}H-decoupled, 376 MHz, $CDCl_3$): − 65.6 (s, CF_3).

5.14. *N*-(4-Chlorophenyl)-2,4-dimethoxybenzothioamide

Purified using silica gel column chromatography eluting with 5% ethyl acetate in hexane to give a yellow solid (0.172 g, 82%), m.p. = 141-143 °C. IR (cm^{-1}): 3299 (NH), 2936 (ArCH), 2835(CH_3), 1603, 1561, 1492, 1160 (C=S), 972, 824; ^{1}H-NMR (300 MHz, $CDCl_3$): δ 10.90 (1H, br s, NH), 8.66 (1H, d, J = 9.0 Hz, H6), 7.69 (2H, br d, J = 8.7 Hz, H2`, 6`), 7.38 (2H, br d, J = 8.7 Hz, H3`, 5`), 6.63 (1H, dd, J_1 = 9.0 Hz, J_2 = 2.4 Hz, H5), 6.49 (1H, d, J = 2.1Hz, H3), 4.0 (3H, s, OCH_3), 3.88 (3H, s, OCH_3) ; ^{13}C-NMR (125 MHz, $CDCl_3$) (assignments made with the aid of DEPT-135): δ 194.3 (C=S), 163.7 (C4), 156.7 (C2), 138.11 (C1`), 138.09 (C6), 131.8 (C4`), 128.9 (C3`, 5`), 125.9 (C2`, 6`), 120.9 (C1), 105.9 (C5), 98.6 (C3), 56.4 (OCH_3), 55.7 (OCH_3).

5.15. *N*-(4-Cyanophenyl)-2,4-dimethoxybenzothioamide

Purified using silica gel column chromatography eluting with DCM to give a yellow solid (0.155 g, 98%). IR (cm^{-1}): 3310 (NH), 3008 (ArCH), 2222 (CN), 1594, 1506, 1160 (C=S), 975; ^{1}H-NMR (300 MHz, $CDCl_3$): δ 11.13 (1H, br s, NH), 8.63 (1H, d, J = 9.3 Hz, H6), 8.01 (2H, br d, J = 7.8 Hz, H2`, 6`), 7.70 (2H, br d, J = 8.4 Hz, H3`, 5`), 6.65 (1H, dd, J_1 = 9.0 Hz, J_2 = 2.1Hz, H5), 6.50 (1H, br s, H3), 4.02 (3H, s, OCH_3), 3.88 (s, 3H, OCH_3); ^{13}C-NMR (125 MHz, $CDCl_3$) (assignments made with the aid of DEPT-135): δ 194.3 (C=S), 164.0 (C4), 156.5 (C2), 143.4 (C1`), 133.2 (C6), 132.8 (C3`, 5`), 124.0 (C2`, 6`), 121.0 (C1), 118.7 (CN), 109.1 (C4`), 106.1 (C5), 98.6 (C3), 56.5 (OCH_3), 55.7 (OCH_3).

5.16. 2,4-Dimethoxy-*N*-(4-nitrophenyl)benzothioamide

Purified using silica gel column chromatography eluting initially with 15% ethyl acetate in hexane, increasing to 50% ethyl acetate in hexane to give an orange a yellow solid; ^{1}H-NMR (300 MHz, $CDCl_3$): δ 11.23 (1H, br s, NH), 8.62 (1H, d, J = 7.5Hz, H6), 8.28 (2H, br d , J = 9.0Hz, H3`, 5`), 8.07 (2H, br d, J = 9.0 Hz, H2`, 6`), 6.64 (1H, dd, J_1 = 8.7 Hz, J_2 = 1.8 Hz, H5), 6.51 (1H, br s, H3), 4.03 (3H, s, OCH_3), 3.89 (3H, s, OCH_3).

5.17. 2,5-Dimethoxy-*N*-(4-methoxyphenyl)benzothioamide

Purified using silica gel column chromatography eluting with 8% ethyl acetate in hexane to give a yellow solid (0.145 g, 91%), m.p. = 96-98 ^{o}C. IR (cm^{-1}): 3296 (NH), 2938 (ArCH), 2837 (CH_3), 1605, 1564, 1508, 1183 (C=S), 828; ^{1}H-NMR (300 MHz, $CDCl_3$): δ 10.79 (1H, br s, NH), 8.12 (1H, d, J = 3.0 Hz, H6), 7.64 (2H, br d, J = 8.7 Hz, H2`, 6`), 6.98 (1H, dd, J_1 = 8.6 Hz, J_2 = 3.3 Hz, H4), 6.94 (2H, br d, J = 9.0 Hz, H3`, 5`), 6.91 (1H, d, J = 9.0 Hz, H3), 3.93 (3H, s, OCH_3), 3.83 (3H, s, OCH_3), 3.82 (3H, s, OCH_3); ^{13}C-NMR (75 MHz, $CDCl_3$) (assignments made with the aid of DEPT-135): δ 193.8 (C=S), 157.9 (C4`), 153.6 (C2), 149.1 (C5), 132.4 (C1`), 128.7 (C1), 125.7 (C2`, 6`), 118.8 (C4), 118.7 (C6), 113.8 (C3`, 5`), 113.4 (C3), 56.9 (OCH_3), 56.0 (OCH_3), 55.3 (OCH_3).

5.18. *N*-(4-Fluorophenyl)-2,5-dimethoxybenzothioamide

Purified using silica gel column chromatography eluting with 5% ethyl acetate in hexane to give a yellow solid (0.152 g, 96%). IR (cm^{-1}): 3338 (NH), 2965 (ArCH), 2837 (CH_3), 1596, 1501, 1160 (C=S), 923, 812; ^{1}H-NMR (300 MHz, $CDCl_3$): δ 10.86 (1H, br s, NH), 8.14 (1H, d, J = 3.0 Hz, H6), 7.71 (2H, dd, J_1 = 8.9 Hz, J_2 = 5.0 Hz, H2`, 6`), 7.12 (2H, t, J = 8.7 Hz, H3`, 5`), 7.01 (1H, dd, J_1 = 9.0 Hz, J_2 = 3.0 Hz, H4), 6.93 (1H, d, J = 9.0 Hz, H3), 3.96 (3H, s, OCH_3), 3.84 (3H, s, OCH_3); ^{13}C-NMR (125 MHz, $CDCl_3$) (assignments made with the aid of DEPT-135): δ 194.7 (C=S), 160.8 (d, J = 245.5 Hz, C4`), 153.8 (C2), 149.3 (C5), 135.4 (d, J = 3.3 Hz, C1`), 128.5 (C1), 126.4 (d, J = 8.4 Hz, C2`, 6`), 119.3 (C4), 119.0 (C6), 115.7 (d, J = 22.5 Hz, C3`, 5`), 113.5 (C3), 57.1 (OCH_3), 55.8 (OCH_3); ^{19}F-NMR (^{1}H-decoupled, 376 MHz, $CDCl_3$): δ −117.4 (s, F); ^{19}F-NMR (^{1}H-coupled, 376 MHz, $CDCl_3$): δ − 117.5 − 117.4 (m, F).

5.19. *N*-(4-Iodophenyl)-2,5-dimethoxybenzothioamide

Purified using silica gel column chromatography eluting with 5% ethyl acetate in hexane to give a yellow solid (0.145 g, 93%). IR (cm^{-1}): 3281 (NH), 2938 (ArCH), 2835 (CH_3), 1595, 1531, 1488, 1162 (C=S), 732; ^{1}H-NMR (300 MHz, $CDCl_3$): δ 10.90 (1H, br s, NH), 8.11 (1H, d, J = 3.0 Hz, H6), 7.74 (2H, br d, J = 8.7 Hz, H3`, 5`), 7.58 (2H, br d, J = 8.7 Hz, H2`, 6`), 7.02 (1H, dd, J_1 = 9.0 Hz, J_2 = 3.3 Hz, H4), 6.93 (1H, d, J = 9.0 Hz, H3), 3.96 (3H, s, OCH_3), 3.85 (3H, s, OCH_3); ^{13}C-NMR (75 MHz, $CDCl_3$) (assignments made with the aid of DEPT-135): δ 194.3 (C=S), 154.0 (C2), 149.2 (C5), 139.1 (C1`), 137.9 (3`, 5`), 128.8 (C1), 125.9 (C2`, 6`), 119.4 (C4), 119.0 (C6), 113.6 (C3), 90.8 (C4`), 57.2 (OCH_3), 55.9 (OCH_3).

5.20. *N*-(4-Bromophenyl)-2,5-dimethoxybenzothioamide

Purified using silica gel column chromatography eluting with 5% ethyl acetate in hexane to give a yellow solid (0.150 g, 93%). IR (cm^{-1}): 3271 (NH), 3088 (ArCH), 2834 (CH_3), 1605, 1551, 1486, 1177 (C=S), 998; ^1H-NMR (300 MHz, $CDCl_3$): δ 10.90 (1H, br s, NH), 8.11 (1H, d, J = 3.0 Hz, H6), 7.69 (2H, br d, J = 8.7 Hz, H2`, 6`), 7.54 (2H, br d, J = 9.0 Hz, H3`, 5`), 7.01 (1H, dd, J_1 = 9.0 Hz, J_2 = 3.0 Hz, H4), 6.93 (1H, d, J = 9.0 Hz, H3), 3.96 (3H, s, OCH_3), 3.84 (3H, s, OCH_3); ^{13}C-NMR (75 MHz, $CDCl_3$) (assignments made with the aid of DEPT-135): δ 194.3 (C=S), 153.9 (C5), 149.2 (C2), 138.4 (C1`), 131.9 (C3`, 5`), 128.7 (C1), 125.7 (C2`, 6`), 121.8 (C4`), 119.3 (C4), 119.0 (C3), 113.6 (C6), 57.1 (OCH_3), 55.8 (OCH_3).

5.21. 2,5-Dimethoxy-*N*-(4-(trifluoromethyl)phenyl)benzothioamide

Purified using silica gel column chromatography eluting with 5% ethyl acetate in hexane to give a yellow solid (0.155 g, 90%), m.p. = 113-114 ^oC. IR (cm^{-1}): 3302 (NH), 2961 (ArCH), 1490, 1516, 1321, 1160 (C=S); ^1H-NMR (300 MHz, $CDCl_3$): δ 11.06 (1H, br s, NH), 8.12 (1H, d, J = 3.0 Hz, H6), 7.97 (2H, br d, J = 8.4 Hz, H3`, 5`), 7.69 (2H, br d, J = 8.4 Hz, H2`, 6`), 7.04 (1H, dd, J_1 = 9.0 Hz, J_2 = 3.0 Hz, H4), 6.96 (1H, d, J = 9.0 Hz, H3), 3.99 (3H, s, OCH_3), 3.86 (3H, s, OCH_3); ^{13}C-NMR (125 MHz, $CDCl_3$) (assignments made with the aid of DEPT-135): δ 194.7 (C=S), 153.9 (C2), 149.1 (C5), 142.3 (C1`), 128.6 (C1), 128.2 (q, J = 32.7 Hz, C4`), 126.0 (q, J = 3.7 Hz, C3`, 5`), 123.85 (q, J = 270.3 Hz, CF_3), 123.83 (C2`, 6`), 119.4 (C6), 119.0 (C4), 113.5 (C3), 57.1 (OCH_3), 55.8 (OCH_3); ^{19}F-NMR (^1H-decoupled, 376 MHz, $CDCl_3$): δ − 65.6 (s, CF_3).

5.22. *N*-(4-Chlorophenyl)-2,5-dimethoxybenzothioamide

Purified using silica gel column chromatography eluting with 5% ethyl acetate in hexane to give a yellow solid (0.148 g, 94%), m.p. = 141-143 ^oC. IR (cm^{-1}): 3237 (NH), 2960 (ArCH), 2837 (CH_3), 1598, 1490, 1321, 1161 (C=S), 703; ^1H-NMR (300 MHz, $CDCl_3$): δ 10.91 (1H, br s, NH), 8.13 (1H, d, J = 3.0 Hz, H6), 7.74 (2H, br d, J = 8.7 Hz, H2`, 6`), 7.40 (2H, br d, J = 8.7 Hz, H3`, 5`), 7.02 (1H, dd, J_1 = 9.0 Hz, J_2 = 3.0Hz, H4), 6.94 (1H, d, J = 9.0 Hz, H3), 3.97 (3H, s, OCH_3), 3.85 (3H, s, OCH_3); ^{13}C-NMR (75 MHz, $CDCl_3$) (assignments made with the aid of DEPT-135): δ 194.5 (C=S), 154.0 (C2), 149.2 (C5), 137.9 (C1`), 131.9 (C4`), 129.0 (C3`, 5`), 128.7 (C1), 125.5 (C2`, 6`), 119.4 (C4), 119.0 (C6), 113.6 (C3), 57.2 (OCH_3), 55.9 (OCH_3).

5.23. *N*-(4-Cyanophenyl)-2,5-dimethoxybenzothioamide

Purified using silica gel column chromatography eluting initially with 10% ethyl acetate in hexane, increasing to 50% of ethyl acetate in hexane to give a yellow solid (0.145 g, 95%). IR (cm^{-1}): 3349 (NH), 2944 (ArCH), 2838 (CH_3), 2219 (CN), 1602, 1534, 1487, 1184 (C=S), 720; ^{1}H-NMR (300 MHz, $CDCl_3$): δ 11.15 (1H, br s, NH), 8.06 (1H, d, $^4J_{HH}$ = 3.0 Hz, H6), 8.04 (2H, br d, J = 8.7 Hz, H2`, 6`), 7.70 (2H, br d, J = 8.7 Hz, H3`, 5`), 7.03 (1H, dd, J_1 = 8.7 Hz, J_2 = 3.0 Hz, H4), 6.95 (1H, d, J = 9.0 Hz, H3), 3.98 (3H, s, OCH_3), 3.84 (3H, s, OCH_3); ^{13}C-NMR (125 MHz, $CDCl_3$) (assignments made with the aid of DEPT-135): δ 194.7 (C=S), 154.0 (C2), 149.1 (C5), 143.1 (C1`), 132.9 (C3`, 5`), 128.8 (C1), 123.6 (C2`, 6`), 119.6 (C4), 119.1 (C6), 118.5 (CN), 113.6 (C3), 109.3 (C4`), 57.2 (OCH_3), 55.8 (OCH_3).

5.24. 2,5-Dimethoxy-*N*-(4-nitrophenyl)benzothioamide

Purified using silica gel column chromatography eluting with 5% of ethyl acetate in hexane, the obtained yellow solid was taken to the next step of synthesis without further purification.

5.25. 3,4-Dimethoxy-*N*-(4-methoxyphenyl)benzothioamide

Purified using silica gel column chromatography eluting with 10% ethyl acetate in hexane to give a yellow solid (0.145 g, 92%), m.p. = 159-160 ^oC. IR (cm^{-1}): 3152 (NH), 2991 (ArCH), 2836 (CH_3), 1597, 1508, 1168 (C=S), 765; ^{1}H-NMR (300 MHz, $CDCl_3$): δ 8.97 (1H, br s, NH), 7.63-7.54 (3H, m, H2`, 6`, 2), 7.36 (1H, br d, J = 8.7 Hz, H6), 6.94 (2H, d, J = 8.7 Hz, H3`, 5`), 6.84 (1H, d, J = 8.4 Hz, H5), 3.94 (3H, s, OCH_3), 3.92 (s, 3H, OCH_3), 3.83 (s, 3H, OCH_3); ^{13}C-NMR (125 MHz, $CDCl_3$) (assignments made with the aid of DEPT-135): δ 197.3 (C=S), 158.1 (C4`), 151.9 (C4), 148.7 (C3), 135.4 (C1), 132.1 (C1`), 125.8 (C2`, 6`), 118.1 (C6), 114.1 (C3`, 5`), 111.7 (C2), 110.0 (C5), 56.1 (OCH_3), 56.0 (OCH_3), 55.4 (OCH_3).

5.26. *N*-(4-Fluorophenyl)-3,4-dimethoxybenzothioamide

Purified using silica gel column chromatography eluting initially with 10% ethyl acetate in hexane, increasing to 20% of ethyl acetate in hexane to give a yellow solid (0.154 g, 97%), m.p. = 163-165 ^oC. IR (cm^{-1}): 3175 (NH), 2997 (ArCH), 2841 (CH), 1593, 1506, 1171(C=S), 812; ^{1}H-NMR (300 MHz, $CDCl_3$): δ 8.96 (1H, br s, NH), 7.66 (2H, br s, H2`, 6`), 7.58 (1H, br s, H2), 7.36 (1H, d, J = 7.5 Hz, H6), 7.12 (2H, t, J = 8.1 Hz, 3`, 5`), 6.85 (1H, d, J = 8.1 Hz, H5), 3.93 (6H, s, 2 x OCH_3); ^{13}C-NMR (125 MHz, $CDCl_3$) (assignments made with the aid of DEPT-135): δ 197.9 (C=S), 160.8 (d, J = 245.8 Hz, C4`), 152.0 (C4), 148.8 (C3), 135.3 (C1`), 135.1 (C1), 126.2 (d, J = 7.5 Hz, C2`, 6`), 118.2 (C6), 115.8 (d, J = 22.6 Hz, C3`, 5`), 111.7

(C2), 110.1 (C5), 56.1 (OCH_3), 56.0 (OCH_3); ^{19}F-NMR (^{1}H-decoupled, 376 MHz, $CDCl_3$): δ − 117.1 (s, F).

5.27. *N*-(4-Iodophenyl)-3, 4-dimethoxybenzothioamide
Purified using silica gel column chromatography eluting with 5% ethyl acetate in hexane to give a yellow solid (0.107 g, 93%), m.p. = 198-200 °C. IR (cm^{-1}): 3303 (NH), 2961 (ArCH), 2837 (CH_3), 1594, 1504, 1168 (C=S), 726; ^{1}H-NMR (300 MHz, $CDCl_3$): δ 8.94 (1H, br s, NH), 7.73 (2H, br d, J = 8.4 Hz, H3`, 5`), 7.60-7.40 (3H, m, H2`, 6`, 2), 7.33 (1H, d, J = 8.1 Hz, H6), 6.84 (1H, br d, J = 8.4 Hz, H5), 3.93 (6H, s, 2 x OCH_3); ^{13}C-NMR (125 MHz, $CDCl_3$) (assignments made with the aid of DEPT-135): δ 197.4 (C=S), 152.1 (C4), 148.8 (C3), 138.9 (C1`), 138.0 (C3`, 5`), 129.0 (C1), 125.5 (C2`, 6`), 111.7 (C6), 110.1 (C2, 5), 90.9 (C4`) 56.12 (OCH_3), 56.06 (OCH_3).

5.28. *N*-(4-Bromophenyl)-3,4-dimethoxybenzothioamide
Purified using silica gel column chromatography eluting with 10% ethyl acetate in hexane to give a yellow solid (0.145 g, 92%), m.p. = 196-198 °C. IR (cm^{-1}): 3143 (NH), 2965 (ArCH), 2840 (CH_3), 1592, 1504, 1091 (C=S); ^{1}H-NMR (300 MHz, $CDCl_3$): δ 8.96 (1H, br s, NH), 7.60 (1H, br s, H2), 7.53 (4H, d, J = 7.8 Hz, H2`, 6`, 3`, 5`), 7.33 (1H, d, J = 7.8 Hz, H6), 6.84 (1H, br d, J = 8.4 Hz, H5), 3.93 (6H, s, 2 x OCH_3); ^{13}C-NMR (125 MHz, $CDCl_3$) (assignments made with the aid of DEPT-135): δ 197.7 (C=S), 152.1 (C4), 148.8 (C3), 138.1 (C1`, 1), 132.1 (C3`, 5`), 125.4 (C2`, 6`), 119.8 (C4`), 111.7 (C6), 110.1 (C2, 5), 56.1 (OCH_3), 56.07 (OCH_3).

5.29. 3,4-Dimethoxy-*N*-(4-(trifluoromethyl)phenyl)benzothioamide
Purified using silica gel column chromatography eluting with initially with 8% ethyl acetate in hexane, increasing to 15% of ethyl acetate in hexane to give a yellow solid (0.150 g, 87%), m.p. = 146-148 °C. IR (cm^{-1}): 3168 (NH), 2993 (ArCH), 2841 (CH_3), 1590, 1516, 1172 (C=S), 860; ^{1}H-NMR (300 MHz, $CDCl_3$): δ 9.07 (1H, br s, NH), 7.88 (2H, br d, J = 7.5 Hz, H3`, 5`), 7.68 (2H, br d, J = 8.4 Hz, H2`, 6`), 7.54 (1H, d, J = 1.5 Hz, H2), 7.35 (1H, dd, J_1 = 8.3 Hz, J_2 = 2.0 Hz, H6), 6.85 (1H, d, J = 8.4 Hz, H5), 3.94 (6H, s, 2 x OCH_3), 3.936 (3H, s, OCH_3); ^{13}C-NMR (75 MHz, $CDCl_3$) (assignments made with the aid of DEPT-135): δ 198.1 (C=S), 152.3 (C4), 149.0 (C3), 142.1 (C1`), 135.4 (C1), 128.3 (q, J = 32.5 Hz, C4`), 126.2 (q, J = 3.8 Hz, C3`, 5`), 123.8 (q, J = 269.9 Hz, CF_3), 123.4 (C2`, 6`), 118.3 (C6), 111.8 (C2), 110.2 (C5), 56.15 (OCH_3), 56.10 (OCH_3); ^{19}F-NMR (^{1}H-decoupled, 376 MHz, $CDCl_3$): δ − 65.6 (s, CF_3).

5.30. ***N*-(4-Chlorophenyl)-3,4-dimethoxybenzothioamide**
Purified using silica gel column chromatography eluting initially with 7% ethyl acetate in hexane, increasing to 15% ethyl acetate in hexane to give a yellow solid (0.149 g, 95%), m.p. = 160-162 ^{o}C. IR (cm^{-1}): 3162 (NH), 2968 (ArCH), 2838 (CH_3), 1591, 1487, 1198 (C=S); ^{1}H-NMR (300 MHz, $CDCl_3$): δ 8.98 (1H, br s, NH), 7.66 (2H, br s, H2`, 6`), 7.54 (1H, br s, H2), 7.38 (2H, br d, J = 8.4 Hz, H3`, 5`), 7.32 (1H, br s, H6), 6.84 (1H, br d, J = 8.4 Hz, H5), 3.92 (6H, s, 2 x OCH_3); ^{13}C-NMR (125 MHz, $CDCl_3$) (assignments made with the aid of DEPT-135): δ 197.6 (C=S), 152.1 (C4), 148.8 (C3), 137.6 (C1`, 1), 131.9 (C4`), 129.1 (C3`, 5`), 125.2 (C2`, 6`), 111.7 (C6), 110.1 (C2, 5), 56.1 (OCH_3), 56.0 (OCH_3).

5.31. ***N*-(4-Cyanophenyl)-3,4-dimethoxybenzothioamide**
Purified using silica gel column chromatography eluting initially with 10% ethyl acetate in hexane, increasing to 50% of ethyl acetate in hexane to give a yellow solid (0.126 g, 82%), m.p. = 199-202 °C. IR (cm^{-1}): 3317 (NH), 2934 (ArCH), 2844 (CH_3), 2223 (CN), 1586, 1508, 1177 (C=S), 870; ^{1}H-NMR (300 MHz, DMSO-d6): δ 11.70 (1H, br s, NH), 8.09 (2H, br d, J = 8.4 Hz, H2`, 6`), 7.80 (1H, br d, J = 8.4 Hz, H3`, 5`), 7.50 (1H, br d, J = 7.8 Hz, H6), 7.48 (1H, br s, H2), 6.99 (1H, br d, J = 9.0 Hz, H5), 3.84 (6H, s, 2 x OCH_3); ^{13}C-NMR (75 MHz, DMSO-d6) (assignments made with the aid of DEPT-135): δ 197.4 (C=S), 151.6 (C4), 147.7 (3), 144.1 (C1`), 134.6 (C1), 132.3 (C3`, 5`), 123.9 (C2`, 6`), 120.8 (C6), 118.4 (CN), 111.5 (C2), 110.3 (C5), 107.6 (C4`), 55.6 (OCH_3), 55.5 (OCH_3).

5.32. 3,4-Dimethoxy-*N*-(4-nitrophenyl)benzothioamide
Purified using silica gel column chromatography eluting initially with 15% ethyl acetate in hexane, increasing to 50% of ethyl acetate in hexane to give a brown solid (0.149 g, 95%), m.p. = 186-188 ^{o}C. IR (cm^{-1}): 3288 (NH), 2936 (ArCH), 2853 (CH_3), 1592, 1503, 1168 (C=S), 849; ^{1}H-NMR (300 MHz, DMSO-d_6): δ 11.88 (1H, br s, NH), 8.30 (2H, br d, J = 9.3 Hz, H3`, 5`), 8.17 (2H, br d, J = 9.3 Hz, H2`, 6`), 7.53 (1H, dd, J_1 = 7.5 Hz, $^{4}J_2$ = 1.8 Hz, H6), 7.52 (1H, br s, H2), 7.06 (1H, d, $^{3}J_{HH}$ = 8.7 Hz, H5), 3.84 (3H, s, OCH_3), 3.83 (3H, s, OCH_3); ^{13}C-NMR (125 MHz, DMSO-d_6) (assignments made with the aid of DEPT-135): 197.9 (C=S), 151.9 (C4), 147.8 (C3), 146.1 (C1`), 144.0 (C4`), δ 134.6 (C1), 124.2 (C2`, 6`), 124.0 (C3`, 5`), 121.2 (C6), 111.5 (C2), 110.6 (C5), 55.8 (OCH_3), 55.6 (OCH_3).

5.33. 3,4,5-Trimethoxy-*N*-(4-methoxyphenyl)benzothioamide
Purified using silica gel column chromatography eluting with 10% ethyl acetate in hexane to give a yellow solid (0.149 g, 95%), m.p. = 132-135 ^{o}C. IR (cm^{-1}): 3254 (NH), 2932 (ArCH),

2831 (CH_3), 1588, 1505, 1177 (C=S), 830; ^{1}H-NMR (300 MHz, $CDCl_3$): δ 9.42 (1H, br s, NH), 7.65 (2H, br d, J = 8.7 Hz, H2`, 6`), 6.96 (2H, br s, H2, 6), 6.95 (2H, br d, J = 7.5 Hz, H3`, 5`), 3.85 (6H, s, 2 x OCH_3), 3.84 (6H, s, 2 x OCH_3); ^{13}C-NMR (75 MHz, $CDCl_3$) (assignments made with the aid of DEPT-135): δ 197.7 (C=S), 158.0 (C4`), 152.5 (C3, 5), 139.9 (C4), 138.7 (C1), 132.2 (C1`), 125.5 (C2`, 6`), 113.9 (C3`, 5`), 104.1 (C2, 6), 60.7 (OCH_3), 56.0 (2 x OCH_3), 55.3 (OCH_3).

5.34. *N*-(4-Fluorophenyl)-3,4,5-trimethoxybenzothioamide

Purified using silica gel column chromatography eluting with hexane to give a yellow solid (0.155 g, 96%), m.p. = 166-1698 °C. IR (cm^{-1}): 3298 (NH), 2943 (ArCH), 2829 (CH_3), 1584, 1505, 1155 (C=S), 997; ^{1}H-NMR (300 MHz, $CDCl_3$): δ 9.05 (1H, br s, NH), 7.72 (2H, dd, J_1 = 7.5 Hz, J_2 = 4.5 Hz, H2`, 6`), 7.14 (2H, t, J = 8.3 Hz, H3`, 5`), 7.03 (2H, br s, H2, 6), 3.90 (6H, s, 2 x OCH_3), 3.87 (3H, s, OCH_3); ^{13}C-NMR (125 MHz, $CDCl_3$) (assignments made with the aid of DEPT-135): δ 198.6 (C=S), 160.7 (d, J = 245.9 Hz, C4`), 152.8 (C3, 5), 140.2 (C4), 138.8 (C1), 135.1 (d, J = 1.9 Hz, C1`), 125.9 (d, J = 8.1 Hz, C2`, 6`), 115.8 (d, J = 22.6 Hz, C3`, 5`), 104.0 (C2, 6), 60.9 (OCH_3), 56.1 (2 x OCH_3); ^{19}F-NMR (^{1}H-decoupled, 376 MHz, $CDCl_3$): δ − 117.0 (s, F); ^{19}F-NMR (^{1}H-coupled, 376 MHz, $CDCl_3$): δ − 117.0 (tt, J_1 = 7.9 Hz, J_2 = 4.9 Hz, F).

5.35. *N*-(4-Iodophenyl)-3,4,5-trimethoxybenzothioamide

Purified using silica gel column chromatography eluting with 5% ethyl acetate in hexane to give a yellow solid (0.149 g, 96%), m.p. = 130-131 °C. IR (cm^{-1}): 3303 (NH), 2934 (ArCH), 2834 (CH_3), 1584, 1496, 1160 (C=S), 722; ^{1}H-NMR (300 MHz, $CDCl_3$): δ 9.41 (1H, br s, NH), 7.72 (2H, br d, J = 8.1 Hz, H3`, 5`), 7.57 (2H, br d, J = 5.7 Hz, H2`, 6`), 6.87 (2H, br s, H2, 6), 3.81 (9H, s, 3 x OCH_3); ^{13}C-NMR (75 MHz, $CDCl_3$) (assignments made with the aid of DEPT-135): δ 198.3 (C=S), 152.7 (C3, 5), 140.1 (C4), 138.9 (C1, 1`), 137.9 (C3`, 5`), 125.3 (C2`, 6`), 104.1 (C2, 6), 90.9 (C4`), 60.9 (OCH_3), 56.1 (2 x OCH_3).

5.36. *N*-(4-Bromophenyl)-3,4,5-trimethoxybenzothioamide

Purified using silica gel column chromatography eluting with 10% ethyl acetate in hexane to give a yellow solid (0.154 g, 99%), m.p. = 147-149 °C. IR (cm^{-1}): 3274 (NH), 2938 (ArCH), 2838 (CH_3), 1608, 1580, 1490, 1186 (C=S), 830; ^{1}H-NMR (300 MHz, $CDCl_3$): δ 9.18 (1H, br s, NH), 7.68 (2H, br s, H2`, 6`), 7.55 (2H, br d, J = 8.1 Hz, H3`, 5`), 6.95 (2H, br s, H2, 6), 3.87 (6H, s, 2 x OCH_3), 3.85 (3H, s, OCH_3); ^{13}C-NMR (125 MHz, $CDCl_3$) (assignments made

with the aid of DEPT-135): δ 198.3 (C=S), 152.9 (C3, 5), 140.4 (C4), 138.1 (C1, 1`), 132.1 (C3`, 5`), 125.3 (C2`, 6`), 119.8 (C4`), 104.2 (C2, 6), 60.9 (OCH_3), 56.2 (2 x OCH_3).

5.37. 3,4,5-Trimethoxy-*N*-(4-(trifluoromethyl)phenyl)benzothioamide

Purified using silica gel column chromatography eluting with 10% ethyl acetate in hexane to give a yellow solid (0.156 g, 95%), m.p. = 154-157 ^{o}C. IR (cm^{-1}): 3210 (NH), 2941 (ArCH), 2838 (CH_3), 1586, 1497, 1162 (C=S); ^{1}H-NMR (300 MHz, $CDCl_3$): δ 9.41 (1H, br s, NH), 7.96 (2H, br d, J = 6.3 Hz, H3`, 5`), 7.70 (2H, br d, J = 8.4 Hz, H2`, 6`), 6.92 (2H, br s, H2, 6), 3.85 (9H, s, 3 x OCH_3); ^{13}C-NMR (75 MHz, $CDCl_3$) (assignments made with the aid of DEPT-135): δ 199.0 (C=S), 152.8 (C3, 5), 142.2 (C4), 140.3 (C1`), 138.9 (C1), 128.3 (q, J = 32.7 Hz, C4`), 126.2 (q, J = 3.8 Hz, C3`, 5`), 123.8 (q, J = 270.3 Hz, CF_3), 123.3 (C2`, 6`), 104.1 (C2, 6), 60.9 (OCH_3), 56.1 (2 x OCH_3); ^{19}F-NMR (^{1}H-decoupled, 376 MHz, $CDCl_3$): δ − 65.6 (s, CF_3).

5.38. *N*-(4-Chlorophenyl)-3,4,5-trimethoxybenzothioamide

Purified using silica gel column chromatography eluting with hexane to give a yellow solid (0.149 g, 96%), m.p. = 154-156 ^{o}C. IR (cm^{-1}); 3277 (NH), 2939 (ArCH), 2839 (CH_3), 1579, 1529, 1494, 1184 (C=S), 993, 831; ^{1}H-NMR (300 MHz, $CDCl_3$): δ 9.29 (1H, br s, NH), 7.75 (2H, br d, J = 6.6 Hz, H2`, 6`), 7.40 (2H, br d, J = 8.4 Hz, H3`, 5`), 6.92 (2H, br s, H2, 6), 3.85 (3H, s, OCH_3), 3.84 (6H, s, 2 x OCH_3); ^{13}C-NMR (75 MHz, $CDCl_3$) (assignments made with the aid of DEPT-135): δ 198.5 (C=S), 152.8 (C3, 5), 140.3 (C4), 137.7 (C1, 1`), 132.0 (C4`), 129.1 (C3`, 5`), 125.0 (C2`, 6`) ,104.1 (C2, 6), 60.9 (OCH_3), 56.2 (2 x OCH_3).

5.39. *N*-(4-Cyanophenyl)-3,4,5-trimethoxybenzothioamide

Purified using silica gel column chromatography eluting initially with 10% ethyl acetate in hexane, increasing to 50% ethyl acetate in hexane to give a yellow solid (0.155 g, 99%), m.p. = 202-205 ^{o}C. IR (cm^{-1}): 3269 (NH), 2942 (ArCH), 2837 (CH3), 2229 (CN), 1581, 1503, 1185 (C=S), 994; ^{1}H-NMR (300 MHz, $CDCl_3$): δ 9.39 (1H, br s, NH), 8.02 (2H, br d, J = 8.4 Hz, H2`, 6`), 7.72 (2H, br d, J = 8.4 Hz, H3`, 5`), 6.93 (2H , br s, H2, 6), 3.86 (9H, s, 3 x OCH_3); ^{13}C-NMR (75 MHz, $CDCl_3$) (assignments made with the aid of DEPT-135): δ 198.9 (C=S), 152.9 (C3, 5), 143.0 (C1`), 140.6 (C4), 138.8 (C1), 133.1 (C3`, 5`), 123.2 (C2`, 6`), 118.4 (CN), 109.5 (C4`), 104.2 (C2, 6), 60.9 (OCH_3), 56.2 (2 x OCH_3).

5.40. 3,4,5-Trimethoxy-*N*-(4-nitrophenyl)benzothioamide

Purified using silica gel column chromatography eluting initially with 10% ethyl acetate in hexane, increasing to 50% of ethyl acetate in hexane to give a yellow solid (0.155 g, 97%),

m.p. = 179-181 °C. IR (cm^{-1}): 3335 (NH), 2943 (ArCH), 2836 (CH_3), 1584, 1507, 1182 (C=S); ^{1}H-NMR (300 MHz, $CDCl_3$): δ 9.62 (1H, br s, NH), 8.30 (2H, br d, J = 9.0 Hz, H3`, 5`), 8.10 (2H, br d, J = 9.0 Hz, H2`, 6`), 6.88 (2H, br s, H2, 6), 3.85 (3H, s, OCH_3), 3.84 (6H, s, 2 x OCH_3); ^{13}C-NMR (75 MHz, $CDCl_3$) (assignments made with the aid of DEPT-135): δ 199.3 (C=S), 152.7 (C3, 5), 144.8 (C1`, 4`), 140.1 (C4), 139.1 (C1), 124.7 (C2`, 6`), 122.8 (C3`, 5`), 104.0 (C2, 6), 60.9 (OCH_3), 56.1 (2 x OCH_3).

5.41. 3,5-Difluoro-*N*-(4-methoxyphenyl)benzothioamide

Purified using silica gel column chromatography eluting with 5% ethyl acetate in hexane to give a yellow solid (0.265 g, 97%). IR (cm^{-1}): 3291 (NH), 3021 (ArCH), 2839 (CH_3), 1590, 1508, 1449, 1152 (C=S); ^{1}H-NMR (300 MHz, DMSO-d_6): δ 11.80 (1H, br s, NH), 7.73 (2H, br d, J = 9.0 Hz, H2`, 6`), 7.53 (2H, dd, J_1 = 8.6 Hz, J_2 = 1.7 Hz, H2, 6), 7.44 (1H, tt, J_1 = 9.1 Hz, J_2 = 2.3 Hz, H4), 7.50 (2H, br d, J = 9.0 Hz, H3`, 5`), 3.78 (3H, s, OCH_3); ^{13}C-NMR (125 MHz, DMSO-d_6) (assignments made with the aid of DEPT-135): δ 193.0 (t, J = 2.2 Hz, C=S), 161.7 (dd, J_1 = 245.3 Hz, J_2 = 13.3 Hz, C3, 5), 157.5 (C4`), 145.4 (t, J = 8.9 Hz, C1), 132.6 (C1`), 125.6 (C2`, 6`), 113.6 (C3`, 5`), 110.9 (dd, J_1 = 20.4 Hz, J_2 = 6.7 Hz, C2, 6), 105.7 (t, J = 25.8 Hz, C4), 55.3 (OCH_3); ^{19}F-NMR with (^{1}H-decoupled, 376 MHz, DMSO-d_6): δ − 111.2 (s, 2 x F); ^{19}F-NMR (^{1}H-coupled, 376 MHz, DMSO-d_6): δ − 111.2 (t, J = 8.1 Hz, 2F).

5.42. 3,5-Difluoro-*N*-(4-fluorophenyl)benzothioamide

Purified using silica gel column chromatography eluting with 5% ethyl acetate in hexane to give a yellow solid (0.185 g, 87%). IR (cm^{-1}): 3287 (NH), 2924 (ArCH), 2852 (CH_3), 1593, 1506, 1153 (C=S), 906, 830; ^{1}H-NMR (300 MHz, $CDCl_3$): δ 8.91 (1H, br s, NH), 7.69 (2H, dd, J_1 = 8.3 Hz, J_2 = 4.7 Hz, H2`, 6`), 7.37 (2H, br d, J = 5.4 Hz, H2, 6), 7.15 (2H, t, J = 8.4 Hz, H3`, 5`), 6.96 (1H, t, J = 8.3 Hz, H4); ^{13}C-NMR (125 MHz, $CDCl_3$) (assignments made with the aid of DEPT-135): δ 195.4 (C=S), 162.8 (dd, J_1 = 249.5 Hz, J_2 = 12.4 Hz, C3, 5), 161.0 (d, J = 246.8 Hz, C4`), 145.4 (t, J = 8.5 Hz, C1), 134.4 (d, J = 2.8 Hz, C1`), 126.0 (d, J = 8.4 Hz, C2`, 6`), 116.0 (d, J = 22.9 Hz, C3`, 5`), 110.1 (dd, J_1 = 20.4 Hz, J_2 = 7.3 Hz, C2, 6), 106.4 (t, J = 25.1 Hz, C4); ^{19}F-NMR (^{1}H-decoupled, 376 MHz, $CDCl_3$): δ − 110.9 (s, 2F), -116.2 (s, F); ^{19}F-NMR (^{1}H-coupled, 376 MHz, $CDCl_3$): δ − 110.9 (t, J = 6.8 Hz, 2F), − 116.9 (tt, J_1 = 6.8 Hz, J_2 = 4.5 Hz, F).

5.43. *N*-(4-Fluorophenyl)-3-methoxybenzothioamide

Purified using silica gel column chromatography eluting with 5% ethyl acetate in hexane to give a yellow solid (0.493 g, 88%). IR (cm^{-1}): 3150 (NH), 2959 (ArCH), 2833 (CH_3), 1595,

1503, 1174 (C=S), 757; ^{1}H-NMR (300 MHz, $CDCl_3$): 9.10 (1H, br s, NH), 7.68 (2H, dd, J_1 = 7.7 Hz, J_2 = 4.7 Hz, H2`, 6`), 7.40 (1H, br s, H2), 7.36-7.26 (2H, m, H5, 6), 7.11 (2H, t, J = 7.8 Hz, H3`, 5`), 7.06-7.00 (1H, m, H4), 3.85 (3H, s, OCH_3); ^{13}C-NMR (125 MHz, $CDCl_3$) (assignments made with the aid of DEPT-135): δ 198.5 (C=S), 160.7 (d, J = 245.9 Hz, C4`), 159.5 (C3), 143.9 (C1), 134.8 (d, J = 2.4 Hz, C1`), 129.6 (C5), 126.0 (d, J = 8.1 Hz, C2`, 6`), 118.2 (C6), 117.3 (C4), 115.8 (d, J = 22.6 Hz, C3`, 5`), 112.7 (C2), 55.4 (OCH_3); ^{19}F-NMR (^{1}H-decoupled, 376 MHz, $CDCl_3$): δ − 116.8 (s, F).

5.44. *N*-(4-Fluorophenyl)-4-methoxybenzothioamide

Purified using silica gel column chromatography eluting initially with 5% ethyl acetate in hexane, increasing to 30% of ethyl acetate in hexane to give a yellow solid (0.52 g, 89%), m.p. = 205-208 °C. IR (cm^{-1}): 3150 (NH), 2924 (ArCH), 2853 (CH_3), 1597, 1507, 1176 (C=S), 991, 838; ^{1}H-NMR (300 MHz, DMSO-d_6): δ 11.54 (1H, br s, NH), 7.90 (2H, d, $^3J_{HH}$ = 8.7 Hz, H2, 6), 7.78 (2H, dd, J_1 = 8.7 Hz, J_2 = 5.1Hz, H2`, 6`), 7.26 (2H, t, J = 8.9 Hz, H3`, 5`), 7.01 (2H, d, J = 8.7 Hz, H3, 5), 3.83 (3H, s, OCH_3); ^{13}C-NMR (125 MHz, DMSO-d_6) (assignments made with the aid of DEPT-135): δ 196.7 (C=S), 161.7 (C4), 159.7 (d, J = 241.8 Hz, C4`), 136.5 (d, J = 2.6 Hz, C1`), 134.3 (C1), 129.5 (C2, 6), 126.7 (d, $^3J_{CF}$ = 8.3 Hz, C2`, 6`), 115.1 (d, J = 22.4 Hz, C3`, 5`), 113.2 (C3, 5), 55.5 (OCH_3); ^{19}F-NMR (^{1}H-decoupled, 376 MHz, DMSO-d_6): δ − 117.9 (s, F).

5.45. 3-Fluoro-*N*-(4-fluorophenyl)benzothioamide

Purified using silica gel column chromatography eluting initially with 5% ethyl acetate in hexane, increasing to 30% of ethyl acetate in hexane to give a yellow solid (0.51 g, 96%), m.p. = 109-112 °C. IR (cm^{-1}): 3210 (NH), 3061 (ArCH), 1584, 1144 (C=S), 982, 714; ^{1}H-NMR (300 MHz, $CDCl_3$): δ 8.96 (1H, br s, NH), 7.69 (2H, dd, J_1 = 8.4 Hz, J_2 = 4.8 Hz, H2`, 6`), 7.58 (2H, br d, J = 7.8 Hz, H2, 4), 7.38 (1H, ddd ~ td, J_1 = 7.5 Hz, J_2 = 6.3 Hz, H5), 7.22 (1H, d, J = 8.4 Hz, H6), 7.13 (2H, t, J = 8.6 Hz, H3`, 5`); ^{13}C-NMR (125 MHz, $CDCl_3$) (assignments made with the aid of DEPT-135): δ 197.1 (C=S), 162.5 (d, J = 246.9 Hz, C4`), 160.9 (d, J = 246.5 Hz, C3), 144.7 (d, J = 7.1 Hz, C1), 134.7 (d, J = 1.9 Hz, C1`), 130.3 (d, J = 7.9 Hz, C5), 126.0 (d, J = 8.1 Hz, C2`, 6`), 121.8 (d, J = 1.6 Hz, C6), 118.3 (d, J = 21.1 Hz, C4), 116.0 (d, J = 22.9 Hz, C3`, 5`), 114.5 (d, J = 23.5 Hz, C2); ^{19}F-NMR (^{1}H-decoupled, 376 MHz, $CDCl_3$): δ − 114.7 (s, F at C3), − 116.5 (s, F).

5.46. 4-Fluoro-*N*-(4-fluorophenyl)benzothioamide

Purified using silica gel column chromatography eluting with 5% ethyl acetate in hexane to give a yellow solid (0.53 g, 93%), m.p. = 166-169 °C. IR (cm^{-1}): 3209 (NH), 2924 (ArCH), 1595, 1506, 1196 (C=S), 920; ^{1}H-NMR (300 MHz, $CDCl_3$): δ 8.92 (1H, br s, NH), 7.86 (2H, br s, H2`, 6`), 7.66 (2H, br s, H2, 6), 7.16-7.01 (4H, m, H3, 5, 3`, 5`); ^{13}C-NMR (125 MHz, $CDCl_3$) (assignments made with the aid of DEPT-135): δ 197.5 (C=S), 164.7 (d, J = 251.0 Hz, C4), 160.9 (d, J = 246.0 Hz, C4`), 138.8 (C1), 134.8 (C1`), 128.9 (d, J = 8.6 Hz, C2`, 6`), 126.1 (d, J = 8.3 Hz, C2, 6), 116.0 (d, J = 22.8 Hz, C3`, 5`), 115.6 (d, J = 21.8 Hz, C3, 5); ^{19}F-NMR (^{1}H-decoupled, 376 MHz, $CDCl_3$): δ − 111.4 (s, F), − 116.6 (s, F).

5.47. *N*-(2-Chloro-4-nitrophenyl)-3,5-dimethoxybenzothioamide

Purified using silica gel column chromatography eluting initially with 5% ethyl acetate in hexane, increasing to 10% of ethyl acetate in hexane to give a yellow solid (0.385 g, 58%). IR (cm^{-1}): 3354 (NH), 3076 (ArCH), 2843 (CH_3), 1598, 1499, 1163 (C=S), 801; ^{1}H-NMR (300 MHz, $CDCl_3$): δ 9.57 (1H, br s, NH), 9.29 (1H, d, J = 9.3 Hz, H6`), 8.38 (1H, d, J = 2.4 Hz, H3`), 8.22 (1H, dd, J_1 = 9.3 Hz, J_2 = 2.4 Hz, H5`), 6.99 (2H, d, J = 2.1 Hz, H2, 6), 6.63 (1H, t, J = 2.1 Hz, H4), 3.85 (6H, s, 2 x $OCH_3$); $^{13}$C-NMR (125 MHz, $CDCl_3$) (assignments made with the aid of DEPT-135): δ 198.3 (C=S), 160.9 (C3, 5), 144.9 (C1), 144.3 (C4`), 140.9 (C1`), 125.8 (C2`), 124.9 (C6`), 122.9 (C3`), 122.6 (C5`), 104.9 (C2, 6), 103.8 (C4), 55.6 (2 x OCH_3).

5.48. *N*-(2-Chloro-4-nitrophenyl)-3,4-dimethoxybenzothioamide

Reaction mixture was purified using silica gel column chromatography eluting initially with 10% ethyl acetate in hexane, increasing to 30% of ethyl acetate in hexane to give a yellow solid (0.365 g, 58%). IR (cm^{-1}): 3360 (NH), 2963 (ArCH), 2844 (CH_3), 1585, 1504, 1196 (C=S), 795; ^{1}H-NMR (300 MHz, $CDCl_3$): δ 9.53 (1H, br s, NH), 9.28 (1H, d, J = 9.3 Hz, H6`), 8.37 (1H, d, J = 2.7 Hz, H3`), 8.21 (1H, dd, J_1 = 9.0 Hz, J_2 = 2.4 Hz, H5`), 7.59 (1H, d, J = 2.1Hz, H2), 7.44 (1H, dd, $J_1$ = 8.6 Hz, $J_2$ = 2.3Hz, H6), 6.90 (1H, d, $J_1$ = 8.7 Hz, H5), 3.96 (3H, s, $OCH_3$), 3.95 (3H, s, $OCH_3$); $^{13}$C-NMR (125 MHz, $CDCl_3$) (assignments made with the aid of DEPT-135): δ 197.3 (C=S), 152.8 (C4), 149.1 (C3), 144.1 (C4`), 141.1 (C1`), 135.5 (C1), 125.7 (C2`), 124.8 (C6`), 122.9 (C3`), 122.5 (C5`), 118.3 (C6), 111.8 (C2), 110.2 (C5), 56.2 (OCH_3), 56.1 (OCH_3).

5.49. *N*-(2-Chloro-4-nitrophenyl)-3,4,5-trimethoxybenzothioamide

Purified using silica gel column chromatography eluting initially with 10% ethyl acetate in hexane, increasing to 25% of ethyl acetate in hexane to give a yellow solid (0.380 g, 61%),

m.p. = 170-172 °C. IR (cm^{-1}): 3289 (NH), 2942 (ArCH), 2839 (CH_3), 1584, 1500, 1165 (C=S), 999; ^{1}H-NMR (300 MHz, $CDCl_3$): δ 9.53 (1H, br s, NH), 9.28 (1H, d, J = 9.0 Hz, H6`), 8.39 (1H, d, J = 2.4 Hz, H3`), 8.23 (1H, dd, J_1 = 9.2 Hz, J_2 = 2.3Hz, H5`), 7.13 (2H, br s, H2, 6), 3.94 (6H, s, 2 x $OCH_3$), 3.92 (3H, s, $OCH_3$); $^{13}$C-NMR (125 MHz, $CDCl_3$) (assignments made with the aid of DEPT-135): δ 198.0 (C=S), 153.2 (C3, 5), 144.3 (C4`), 141.7 (C1`), 141.1 (C4), 138.3 (C1), 125.8 (C2`), 124.9 (C6`), 122.9 (C3`), 122.5 (C5`), 104.5 (C2, 6), 61.0 (OCH_3), 56.4 (2 x OCH_3).

6. Synthesis of *N*-Ribosyldihydronicotinamide (NRH)

NADH (0.5 g, 0.7048 mmol) was dissolved in sodium bicarbonate and sodium carbonate buffer (0.4 M, pH 10) and incubated at 37 °C; phosphodiesterase I (type IV phosphodiesterase I from *Crotalusatrox* western diamondback rattlesnake) (0.1 unit) and alkaline phosphatase (from bovin intestinal mucosa) (500 unit) were added to the reaction mixture. The reaction mixture was incubated for 16 hours and then it was freeze dried. The dried powder was extracted with methanol and this extract was concentrated using a rotary evaporator. The resultant dry powder was dissolved in water and purified by preparative HPLC performed on a Microsorb C18 (21.2 X 250 mm) column, eluted with 10% methanol in water over 12 minutes with 0.1ml injection volume and a 21ml/min flow rate. The peak at 350 nm from each injection was collected and freeze dried.[5] The obtained NRH was stored at 4 °C[5]. ^{1}H-NMR (400 MHz, D_2O): δ 6.99 (1H, d, J = 1.2 Hz, H2), 5.95 (1H, dq, J_1 = 8.0 Hz, J_2 = 1.6 Hz, H6), 4.83 (1H, dt, J_1 = 8.4 Hz, J_2 = 3.4 Hz, H5), 4.71(1H, d, J = 6.8 Hz, H1`), 4.05 (1H, t, J = 6.4 Hz, H2`), 3.98 (1H, dd, J_1 = 5.6 Hz, J_2 = 2.8 Hz, H3`), 3.82 (1H, q, $^3J$ = 3.7 Hz, H4`), 3.60 (1H, dd, $^2J_1$ = 12.4 Hz, J_2 = 3.6 Hz, H5`), 3.53 (1H, dd, $J_1$ = 12.6 Hz, $J_2$ = 4.6 Hz, H5`), 2.91(2H, dd, J_1 = 3.2 Hz, J_2 = 1.6 Hz, H4); ^{13}C-NMR (100 MHz, D_2O) (assignment made with aid of DEPT-135): δ 172.9 (C=O), 137.8 (C2), 125.2 (C6), 105.1(C5), 100.9 (C3), 94.8 (C1`), 83.5 (C4`), 70.9 (C2`), 70.1 (C3`), 61.5 (C5`), 21.9 (C4).

7.LCMS Analysis

LC data was obtained using a Waters ACQUITY UPLC PDA detector scanning between 210-400 nm. Mass spectrometry data was acquired using a Waters ACQUITY QDa detector scanning in the positive (ES+) and negative (ES–) modes between m/z 100-1000. Separation of components was achieved using a Waters ACQUITY UPLC BEH C18 1.7 µm 2.1 × 50 mm column coupled to a Waters ACQUTY UPLC BEH C18 1.7 µm VanGuard pre-column 2.1 ×

5 mm. Columns were maintained at 40 °C throughout acquisition. The table below details a schematic for the standard four-minute run on the instrument using a flow rate of 0.60 mL min^{-1}:

Start time / min	End time / min	H_2O:MeCN (% V/V)
0	0.50	95:5
0.50	2.50	95:5 to 5:95
2.50	3.00	5:95
3.00	3.10	5:95 to 95:5
3.100	4.00	95:5

All solvents obtained were of LC-MS grade (Fisher Optima) and were modified by the addition of 0.1% v/v formic acid (Fisher Optima) referred to as method 1 or NH_4OH (Fisher, 28% v/v aq. solution) referred to method 2. Samples were prepared in MeOH (Fisher Optima) and a 2.0 µL aliquot was extracted from each sample for analysis. Samples were maintained at 10 °C prior to acquisition. Solvents were combined using a Waters ACQUITY UPLC H-Class Quaternary Solvent Manager (QSM) with in-built degasser. Inert gas was provided using a Genius NM32LA nitrogen generator. The switch method consisted of 95:5 H_2O:MeCN (with appropriate acidic or basic modifiers) running at 0.60 mL min^{-1} for two minutes immediately prior to acquisition. Data was processed using MassLynx V4.1. Values of purity were obtained through analysis of the peak areas in the LC trace between 0.40 min. and 3.50 min.

Table S1. Predicted transport and toxicity properties of most potent NQO2 inhibitors. Organ toxicity and toxicity end point, BBB (Blood Brain Barrier) and metabolism predicted by ProTox-3.0 server [6,7]. HERG blocking predicted by PRED-hERG web server [8]. BBB and Metabolism predicted by SwissADME [9] as well. Prediction probabilities indicated for selected compounds/properties in parentheses.

Cmpd	HERG blocker [8]	Cardiotoxic [6,7]	Hepatotoxic (probability) [6,7]	Nephrotoxic (probability) [6,7]	Neurotoxic [6,7]	Cytotoxic (probability) [6,7]	Carcinogenic (probability) [6,7]	BBB permeant [9]	Metabolism (CYP1A2 inhibitor)[9]
**15**	No	No	Yes (0.64)	Yes (0.58)	No	No	No	No	Yes
**49**	No	No	Yes (0.61)	No	No	Yes (0.55)	Yes (0.51)	No	Yes
**40**	No	No	Yes (0.62)	No	No	No	No	No	Yes
**48**	No	No	Yes (0.61)	No	No	No	Yes (0.61)	No	Yes
**46**	No	No	Yes (0.68)	Yes (0.51)	No	No	No	Yes	Yes
**3**	No	No	Yes (0.60)	No	No	No	No	Yes	Yes
**5**	No	No	Yes (0.65)	No	No	No	No	No	Yes
**41**	No	No	Yes (0.69)	Yes (0.51)	No	No	No	Yes	Yes
**52**	No	No	Yes (0.61)	Yes (0.57)	No	No	No	No	Yes

8. References

1. Bertini, S.; Calderone, V.; Carboni, I.; Maffei, R.; Martelli, A.; Martinelli, A.; Minutolo, F.; Rajabi, M.; Testai, L.; Tuccinardi, T.; et al. Synthesis of Heterocycle-Based Analogs of Resveratrol and their Antitumor and Vasorelaxing Properties. *Bioorg. Med. Chem.* **2010**, *18*, 6715–6724, doi:10.1016/j.bmc.2010.07.059.

2. Colombo, M.; Bossolo, S.; Aramini, A. Phosphorus Trichloride-Mediated and Microwave-Assisted Synthesis of a Small Collection of Amides Bearing Strong Electron-Withdrawing Group Substituted Anilines. *J. Comb. Chem.* **2009**, *11*, 335–337, doi:10.1021/cc900011z.

3. Cheng, C.G.; Sun, G.L.; Wan, J.P.; Sun, C.R. Facile CuI-Catalyzed Arylation of Azoles and Amides Using Simple Enaminones as Efficient Ligands. *Synlett.* **2009**, *16*, 2663–2668, doi:10.1055/s-0029-1217958.

4. Liou, J.P.; Wu, Z.Y.; Kuo, C.C.; Chang, C.Y.; Lu, P.Y.; Chen, C.M.; Hsieh, H.P.; Chang, J.Y. Discovery of 4-Amino and 4-Hydroxy-1-Aroylindoles as Potent Tubulin Polymerization Inhibitors. *J. Med. Chem.* **2008**, *51*, 4351–4355, doi:10.1021/jm800150d.

5. Long 2nd, D.J.; Iskander, K.; Gaikwad, A.; Arin, M.; Roop, D.R.; Knox, R.; Barrios, R.; Jaiswal, A.K. Disruption of Dihydronicotinamide Riboside:Quinone Oxidoreductase 2 (NQO2) Leads to Myeloid Hyperplasia of Bone Marrow and Decreased Sensitivity to Menadione Toxicity. *J. Biol. Chem.* **2002**, *277*, 46131–46139, doi:10.1074/jbc.M208675200.

6. Banerjee, P.; Kemmler, E.; Dunkel, M.; Preissner, R. ProTox 3.0: A Webserver for the Prediction of Toxicity of Chemicals. *Nucleic Acids Res* **2024**, *52*, W513–W520, doi:10.1093/NAR/GKAE303.

7. Banerjee, P.; Eckert, A.O.; Schrey, A.K.; Preissner, R. ProTox-II: A Webserver for the Prediction of Toxicity of Chemicals. *Nucleic Acids Res* **2018**, *46*, W257–W263, doi:10.1093/NAR/GKY318.

8. Braga, R.C.; Alves, V.M.; Silva, M.F.B.; Muratov, E.; Fourches, D.; Lião, L.M.; Tropsha, A.; Andrade, C.H. Pred-HERG: A Novel Web-Accessible Computational Tool for Predicting Cardiac Toxicity. *Mol Inform* **2015**, *34*, 698–701, doi:10.1002/MINF.201500040.

9. Daina, A.; Michielin, O.; Zoete, V. SwissADME: A Free Web Tool to Evaluate Pharmacokinetics, Drug-Likeness and Medicinal Chemistry Friendliness of Small Molecules. *Scientific Reports 2017 7:1* **2017**, *7*, 1–13, doi:10.1038/srep42717.

9. NMR Spectra and LCMS data of phenylbenzothiazoles 3-58

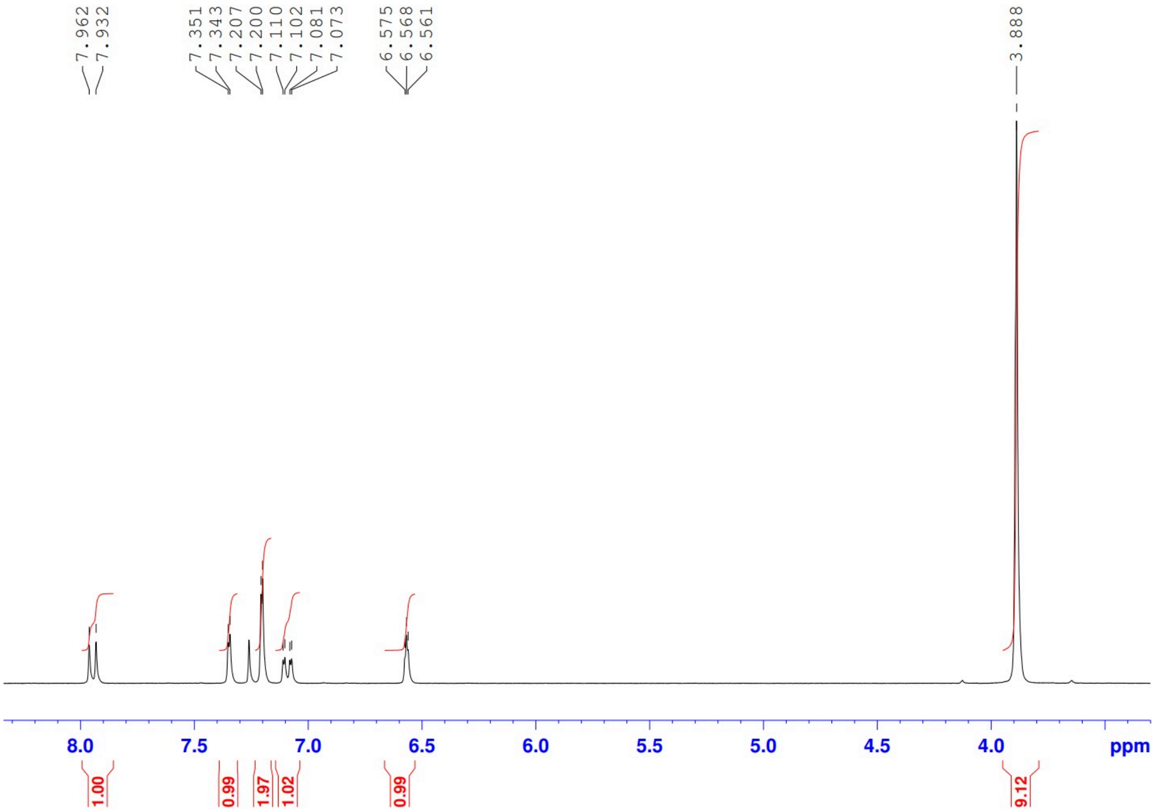

Figure S1. ^{1}**H-NMR (300 MHz, CDCl spectrum of 3)**

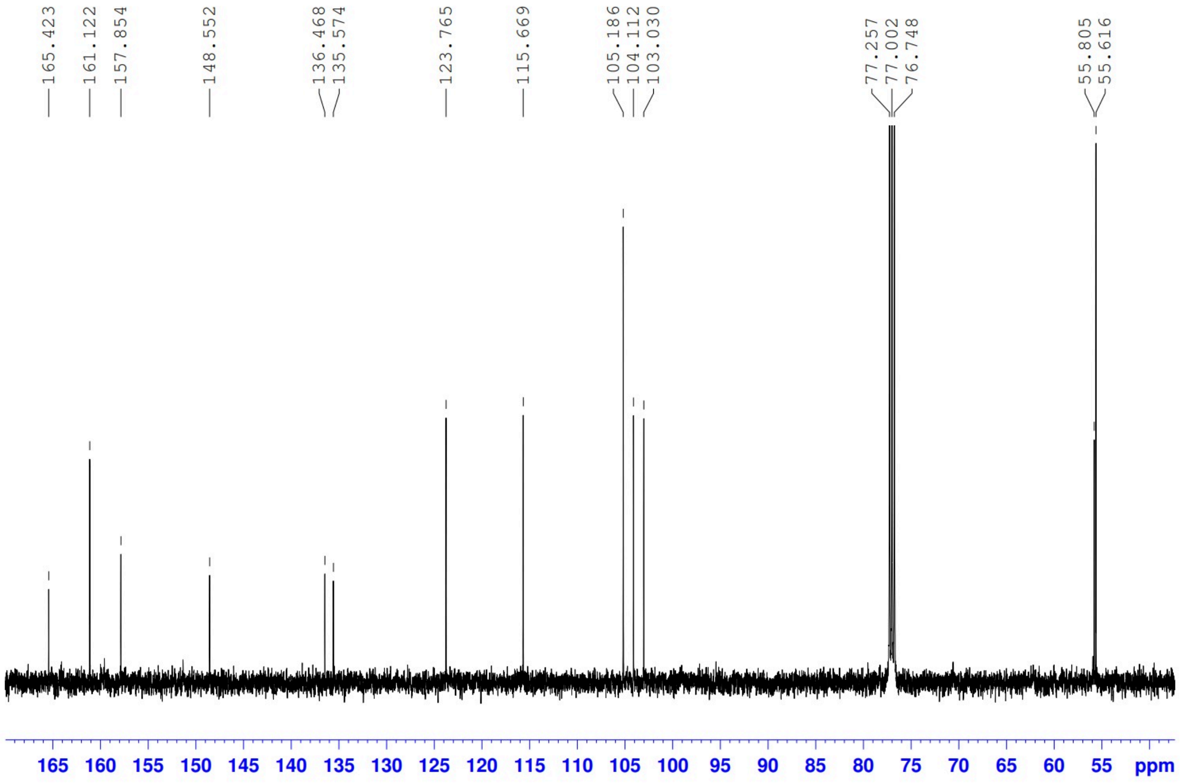

Figure S2. ^{13}**C-NMR (125 MHz, CDCl$_3$) spectrum of 3**

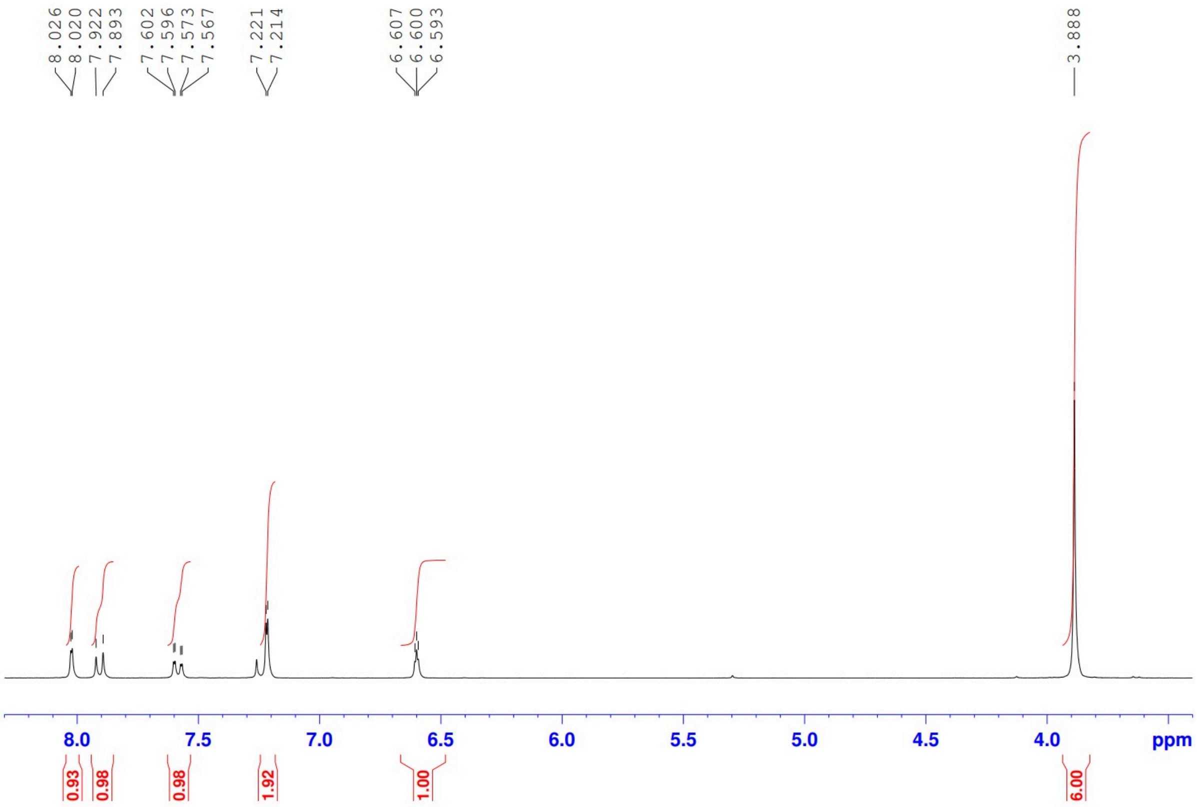

Figure S3. ^{1}**H-NMR (300 MHz, CDCl$_3$) spectrum of 4**

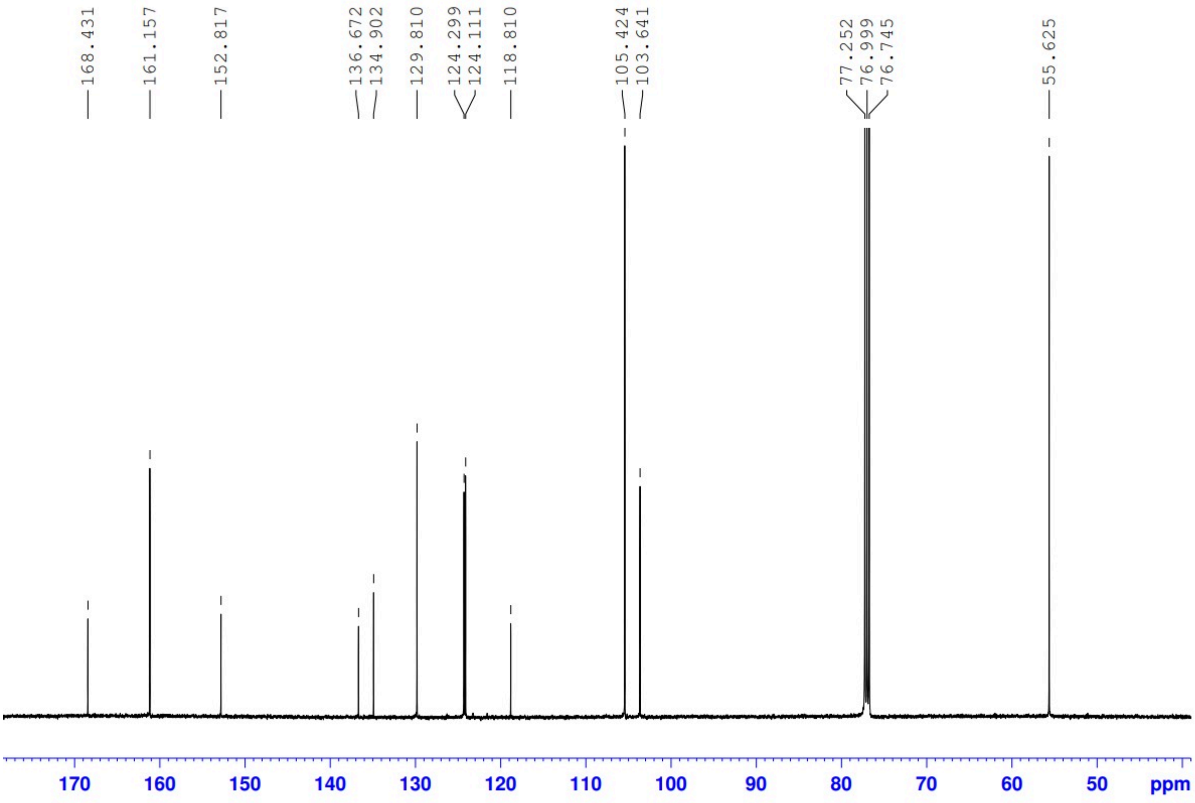

Figure S4. ^{13}**C-NMR (125 MHz, CDCl$_3$) spectrum of 4**

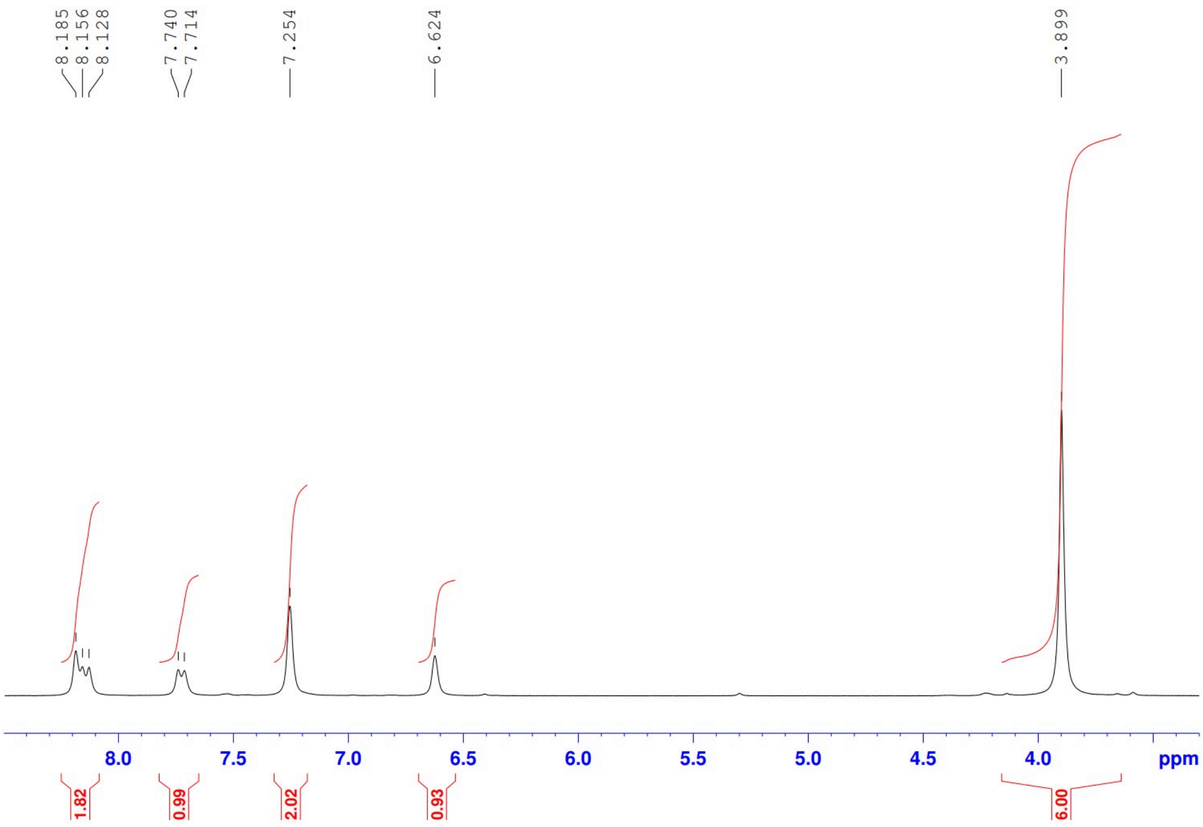

Figure S5. ^{1}**H-NMR (300 MHz, CDCl$_3$) spectrum of 5**

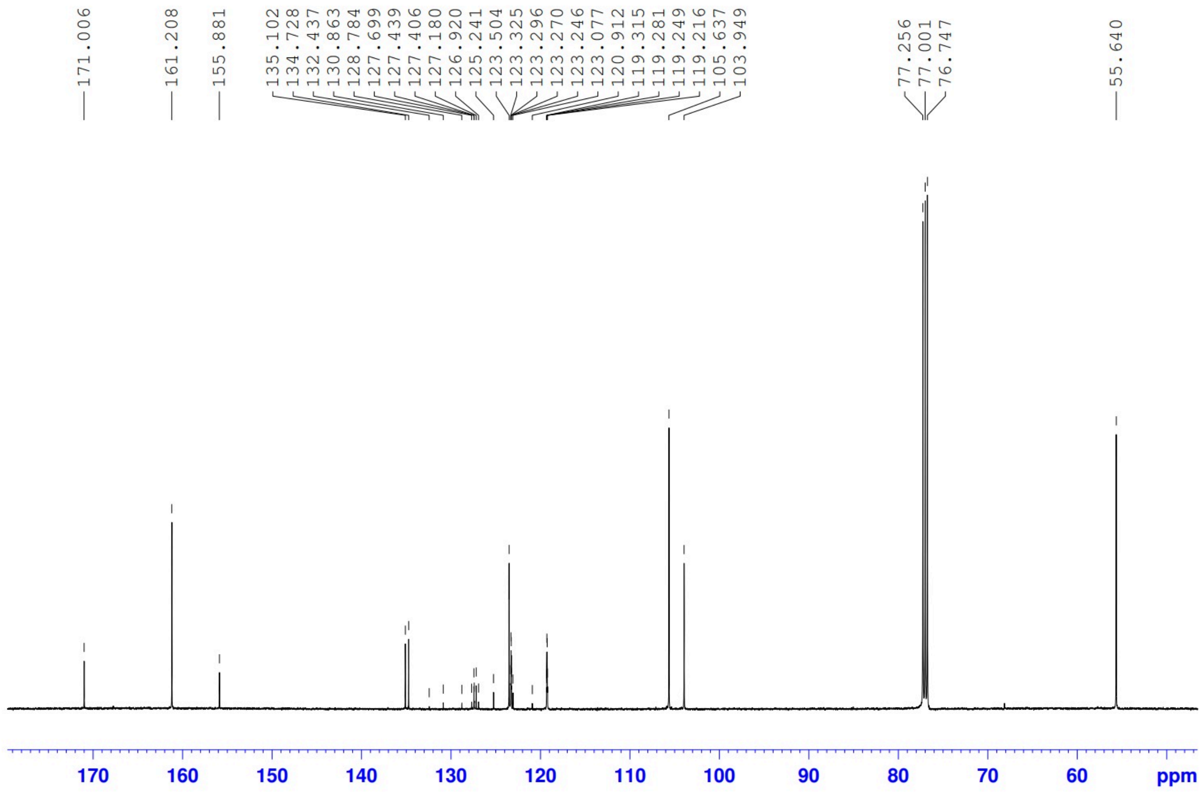

Figure S6. ^{13}**C-NMR (125 MHz, CDCl$_3$) spectrum of 5**

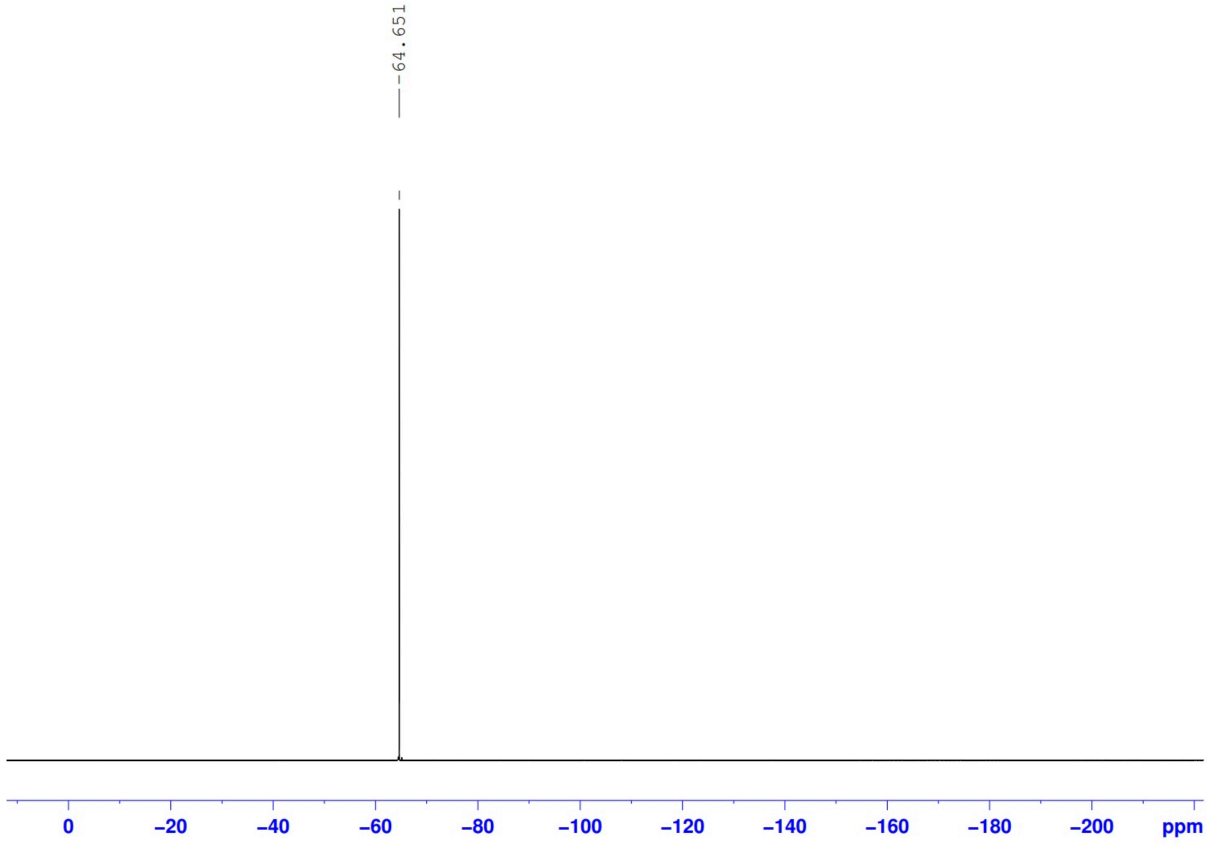

Figure S7. ^{19}**F-NMR (376 MHz, CDCl$_3$) spectrum of 5**

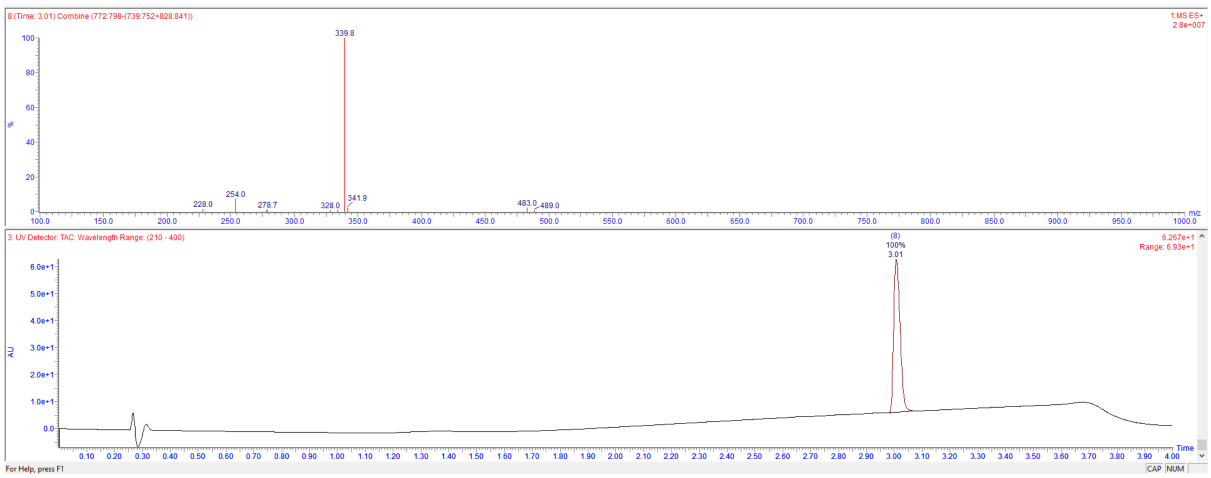

Figure S8. LCMS of 5

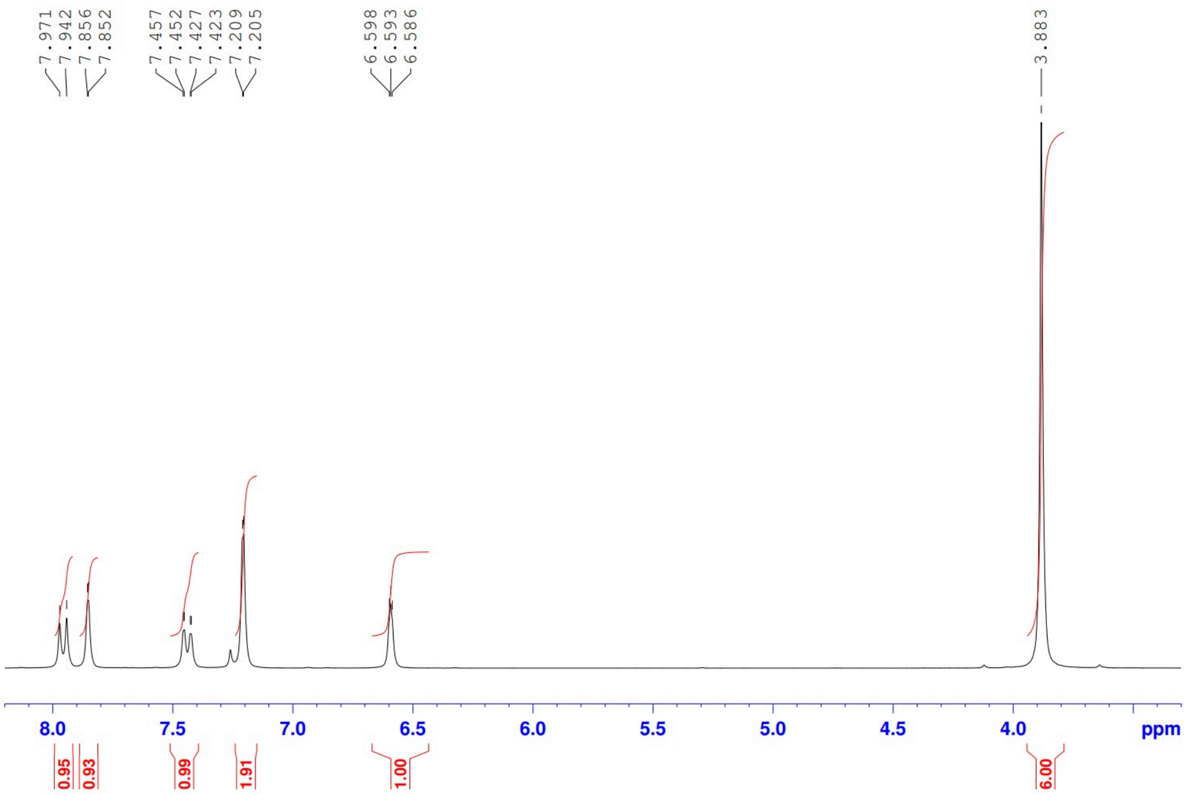

Figure S9. [1]**H-NMR (300 MHz, $CDCl_3$) spectrum of 6**

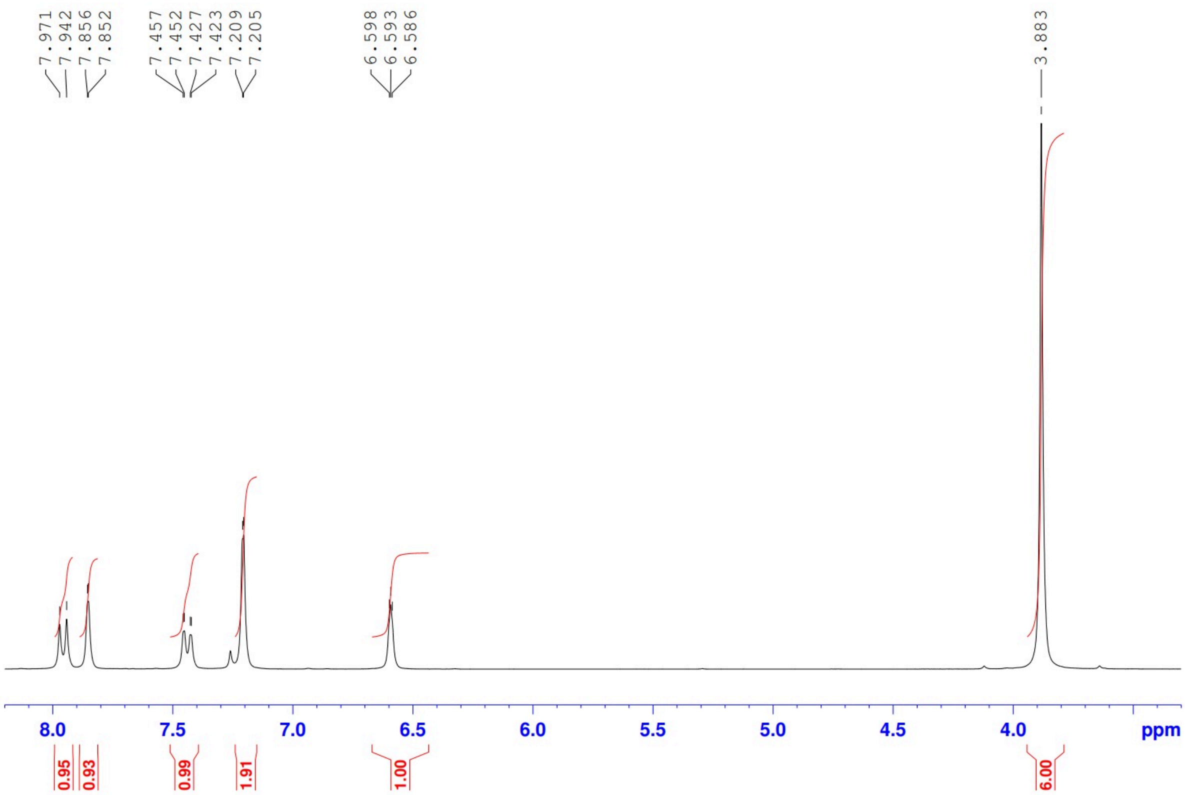

Figure S10. [13]**C-NMR (125 MHz, $CDCl_3$) spectrum of 6**

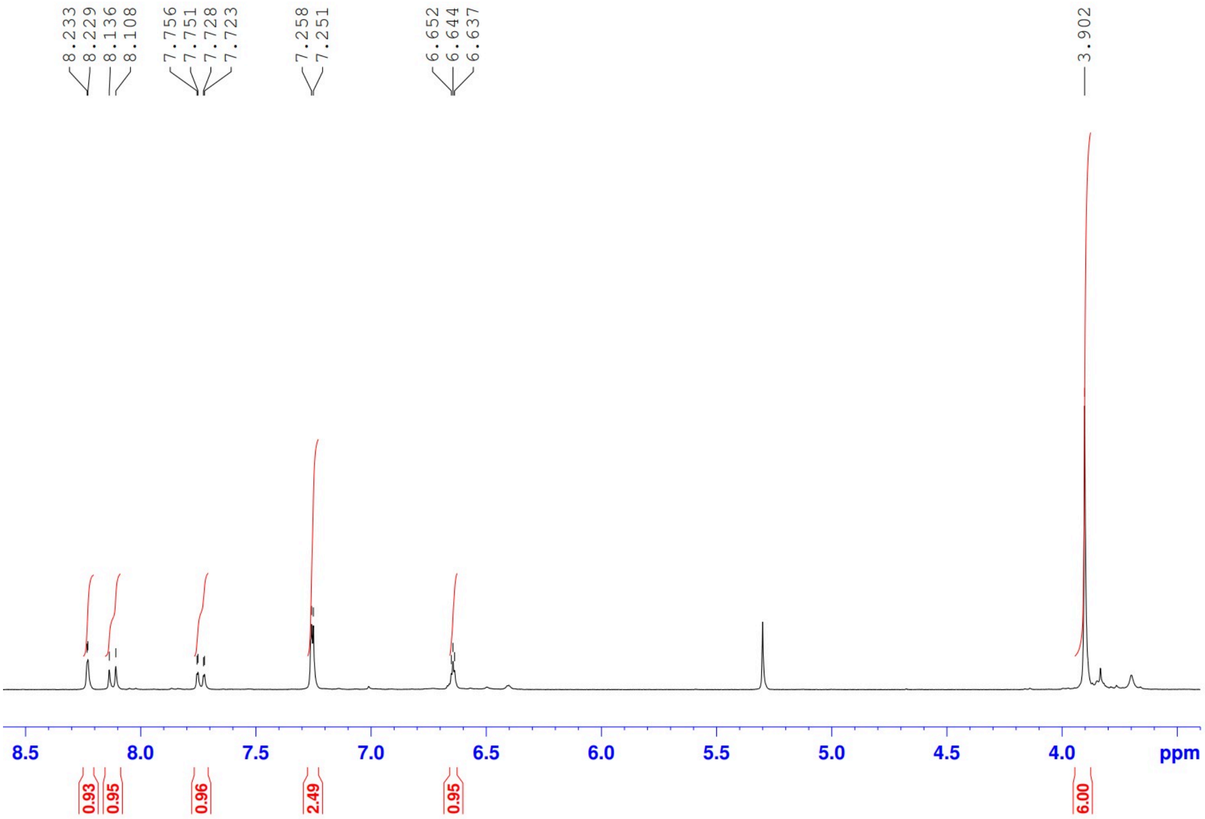

Figure S11. ^{1}**H-NMR (300 MHz, CDCl$_3$) spectrum of 7**

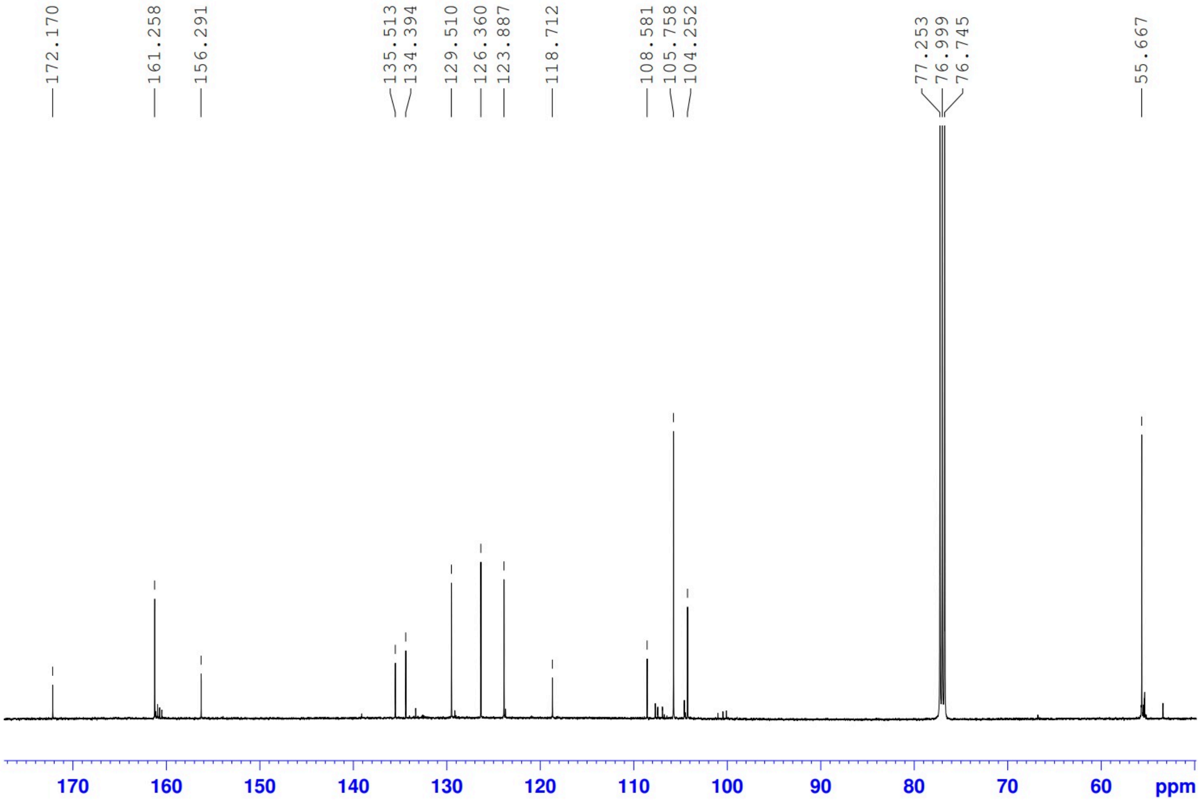

Figure S12. ^{13}**C-NMR (125 MHz, CDCl$_3$) spectrum of 7**

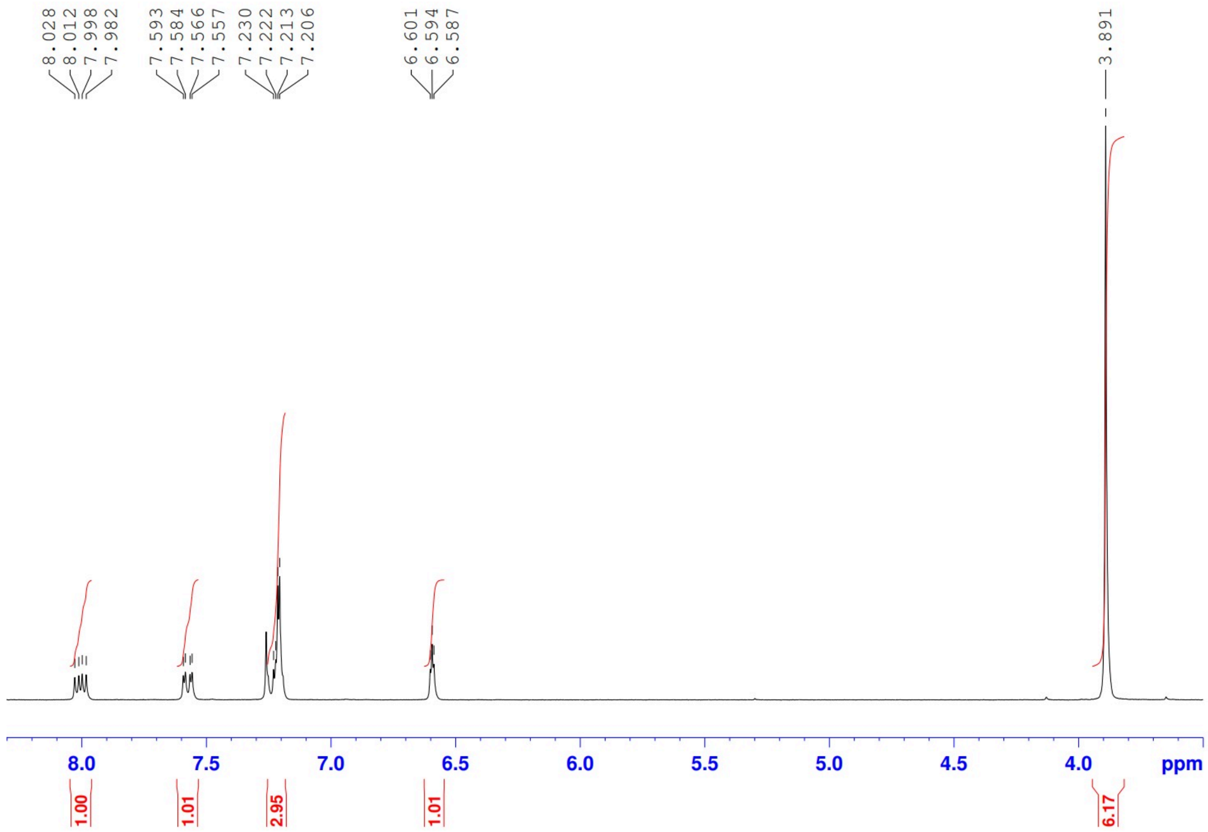

Figure S13. ^{1}H-NMR (300 MHz, $CDCl_3$) spectrum of **8**

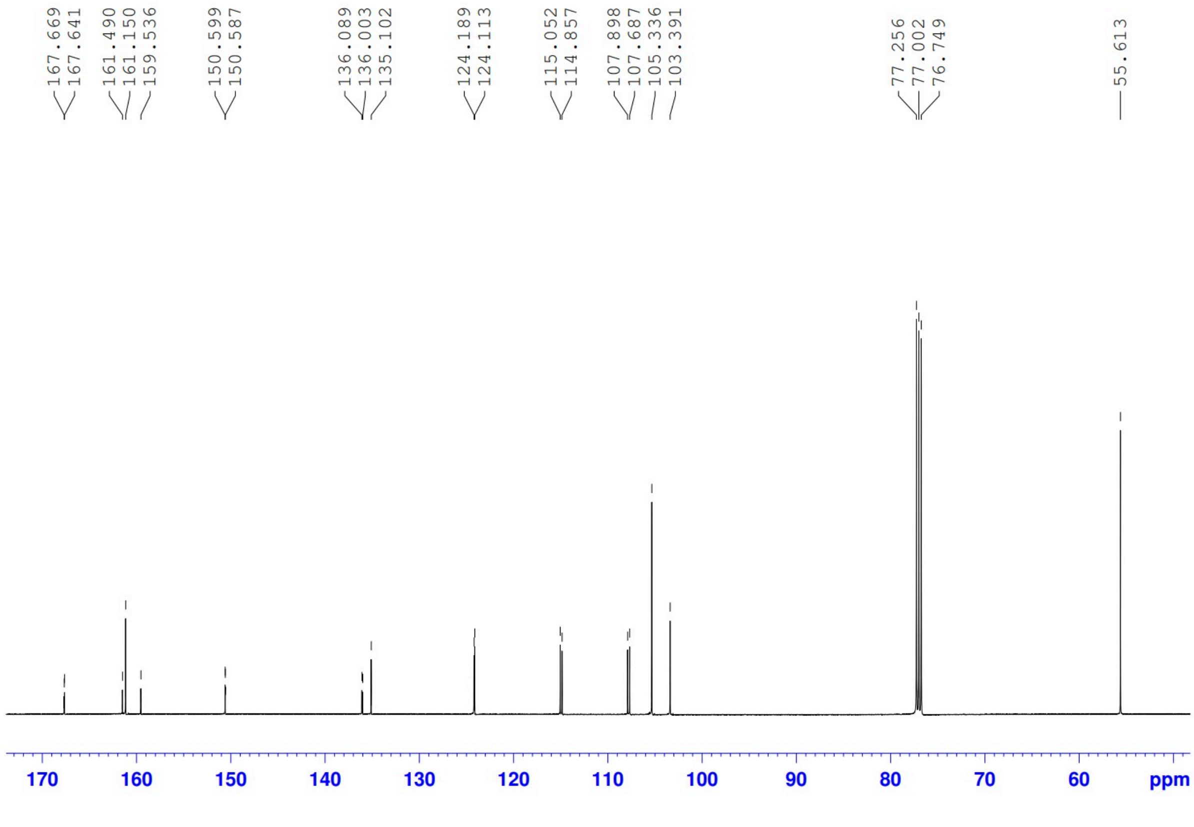

Figure S14. ^{13}C-NMR (125 MHz, $CDCl_3$) spectrum of **8**

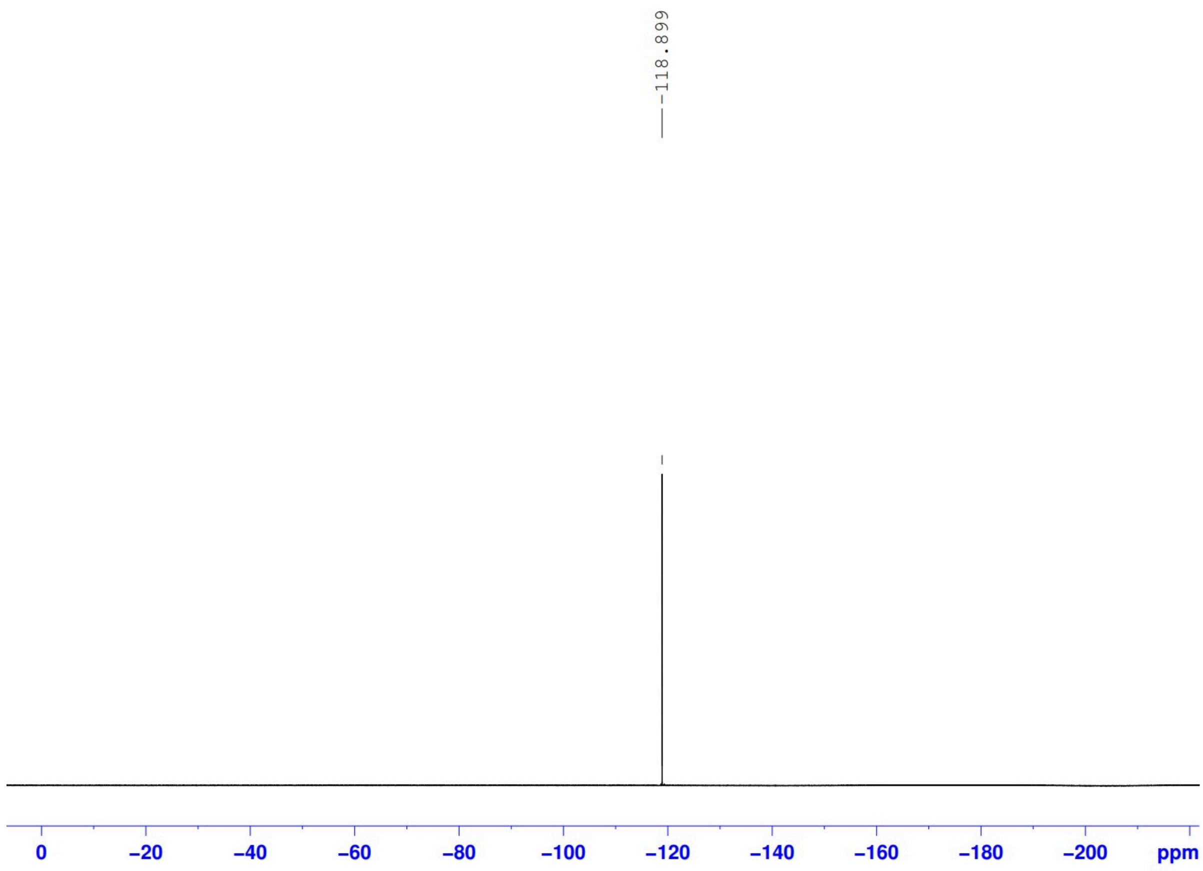

Figure S15. [19]**F-NMR (376 MHz, CDCl$_3$) spectrum of 8**

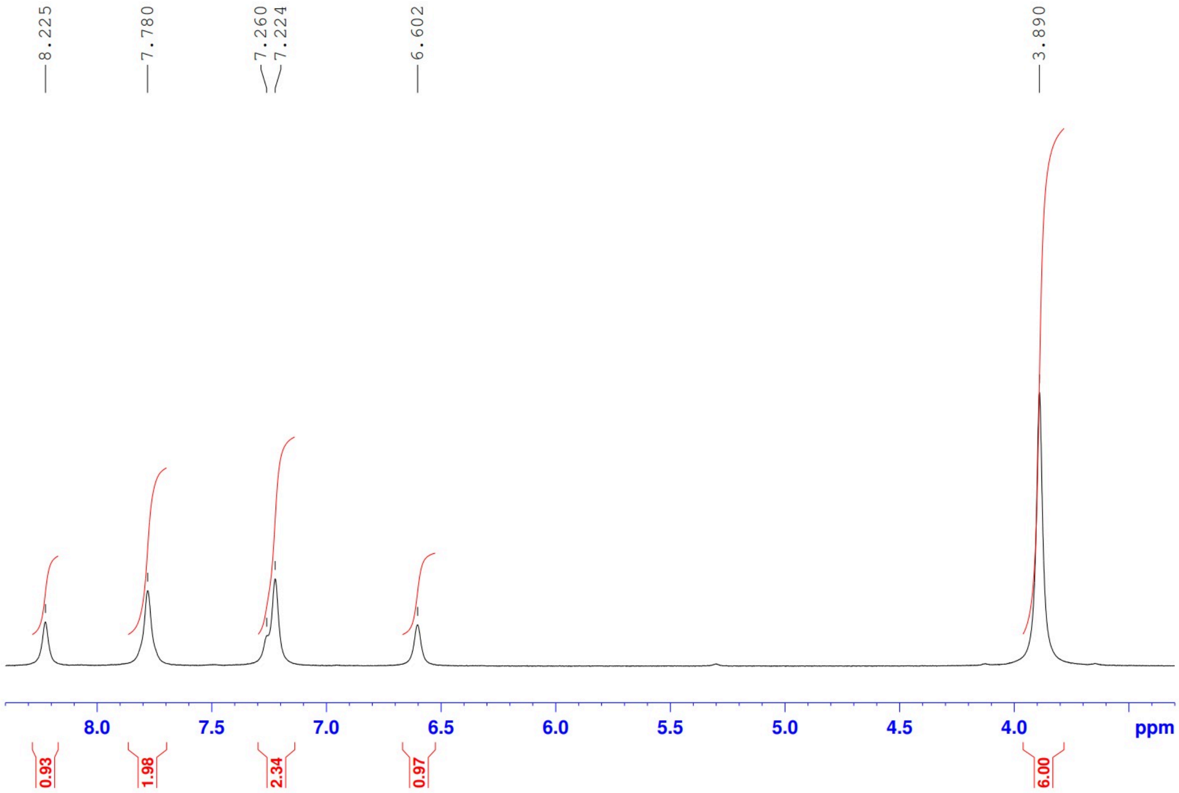

Figure S16. [1]**H-NMR (300 MHz, CDCl$_3$) spectrum of 9**

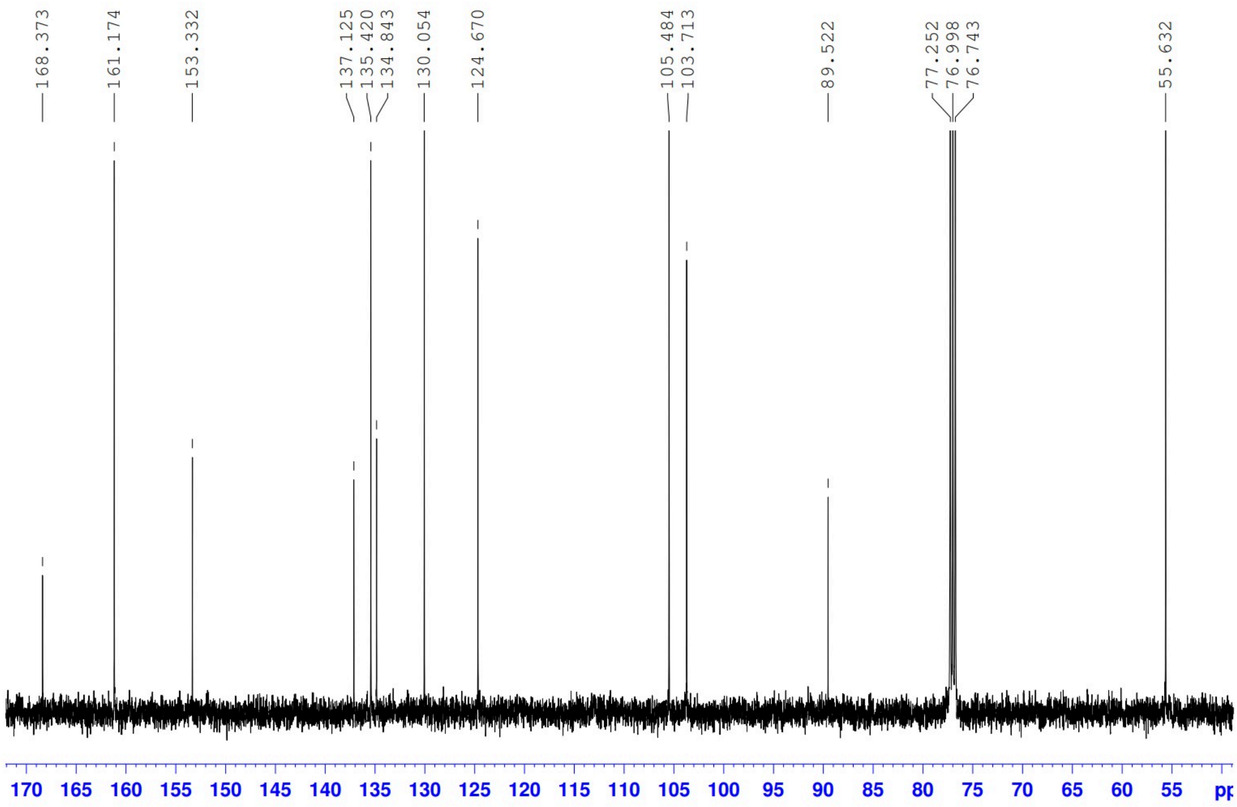

Figure S17. **^{13}C-NMR (125 MHz, CDCl₃) spectrum of 9**

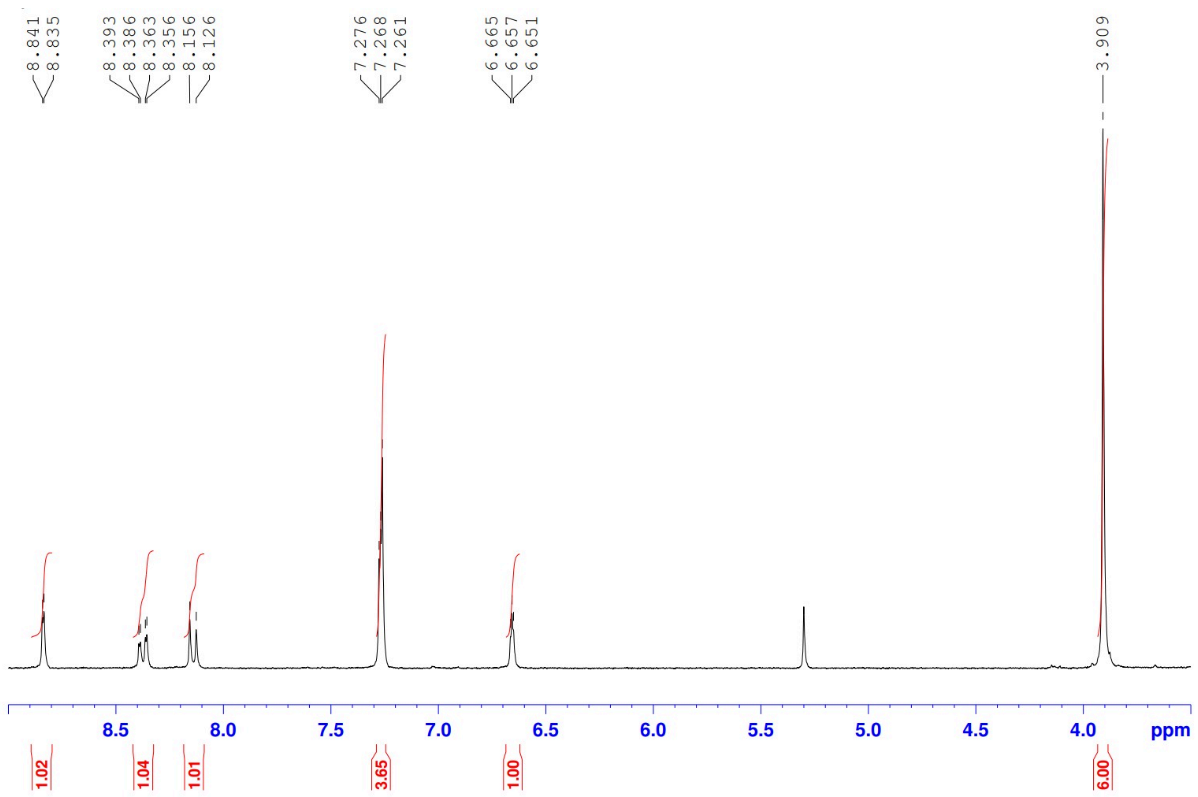

Figure S18. **^{1}H-NMR (300 MHz, CDCl₃) spectrum of 10**

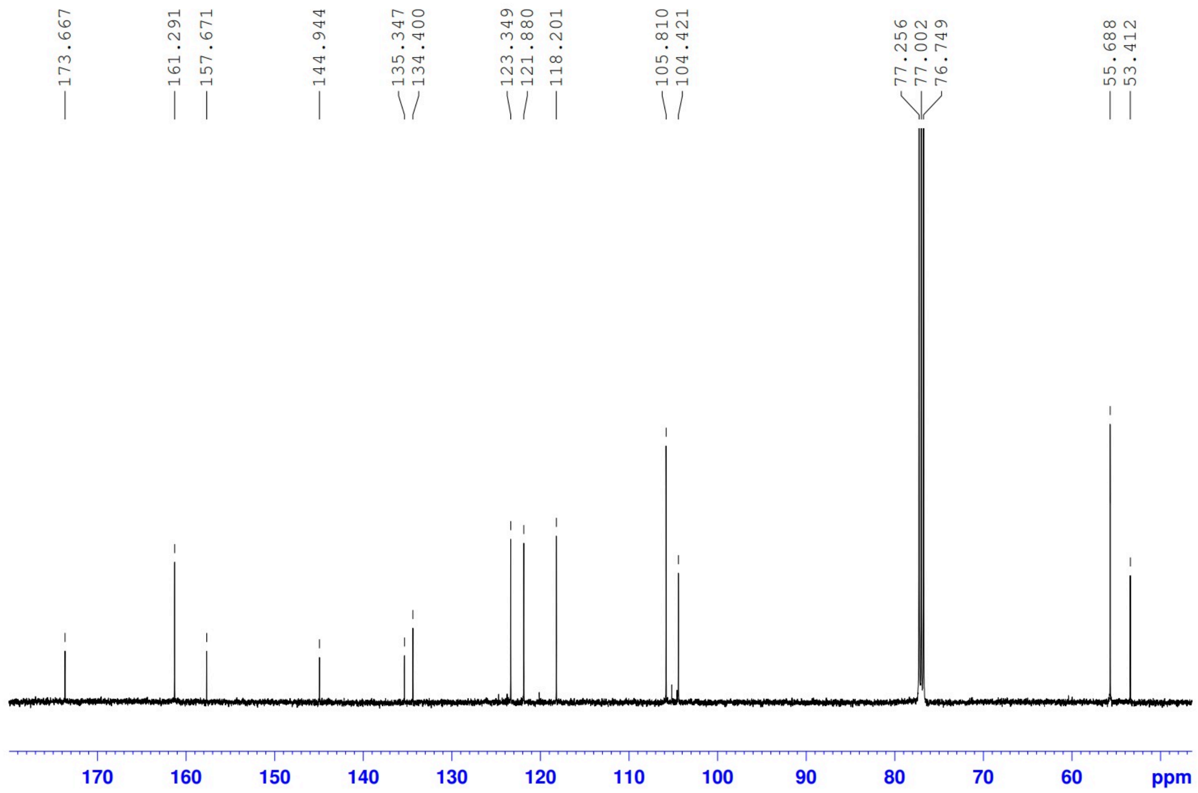

Figure S19. ^{13}**C-NMR (125 MHz, $CDCl_3$) spectrum of 10**

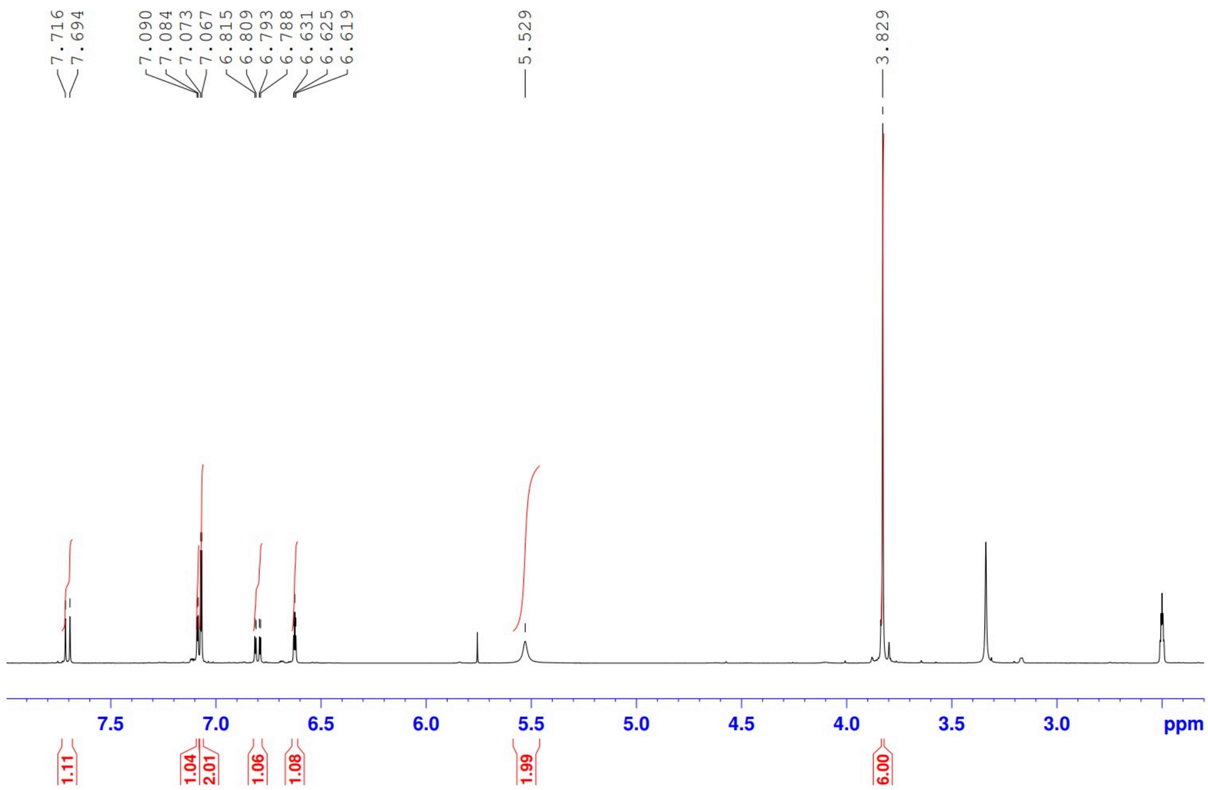

Figure S20. ^{1}**H-NMR (400 MHz, DMSO-d_6) spectrum of 11**

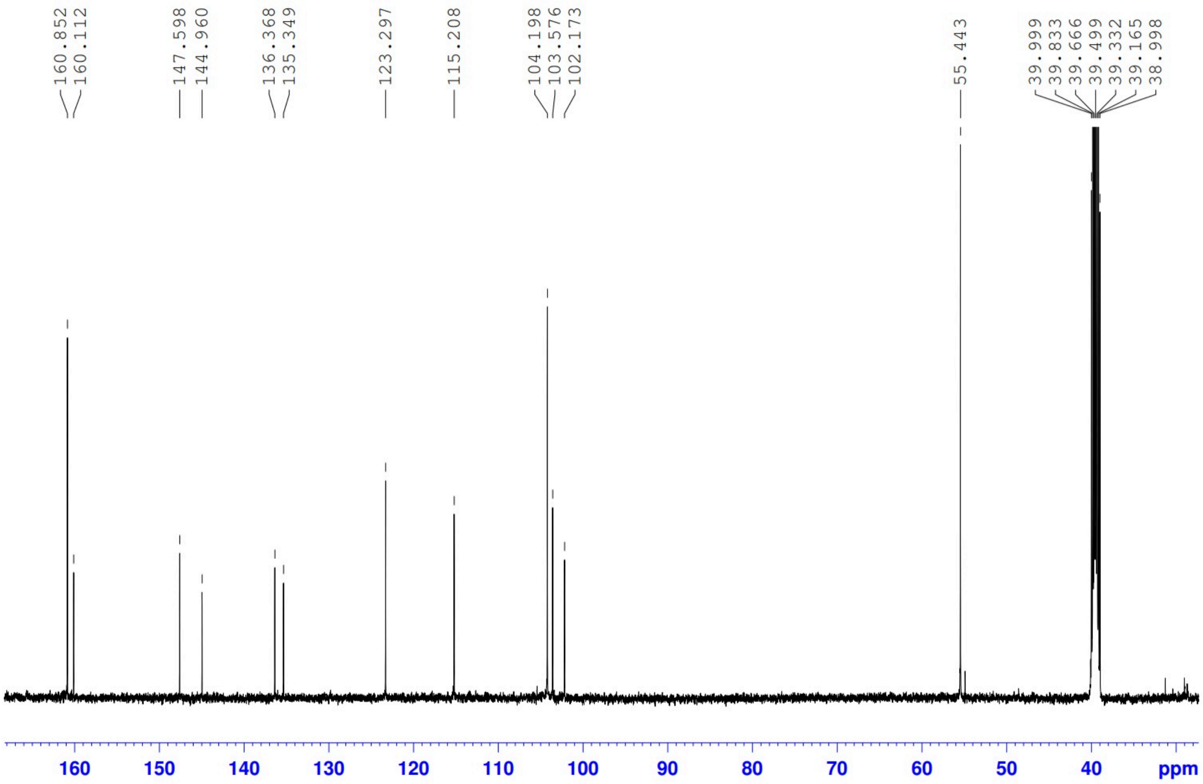

Figure S21. ^{13}**C-NMR (125 MHz, DMSO-d$_6$) spectrum of 11**

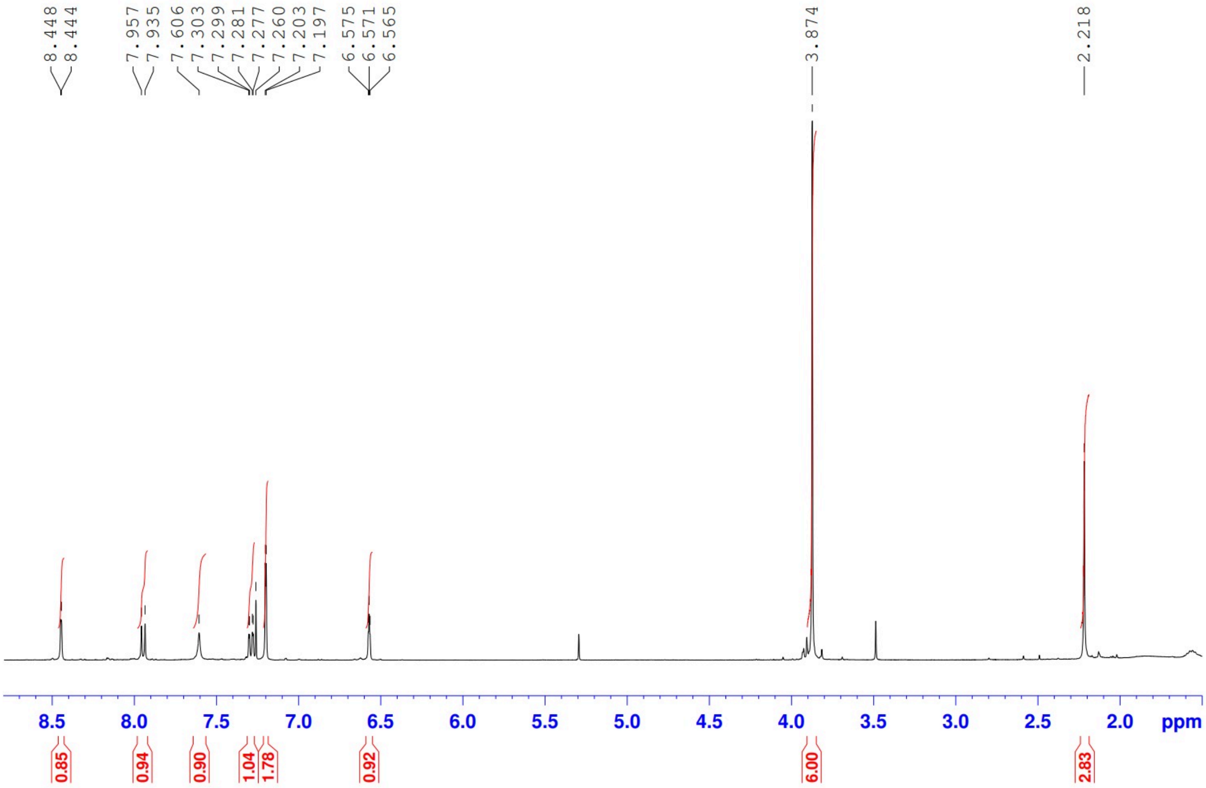

Figure S22. ^{1}**H-NMR (400 MHz, CDCl$_3$) spectrum of 12**

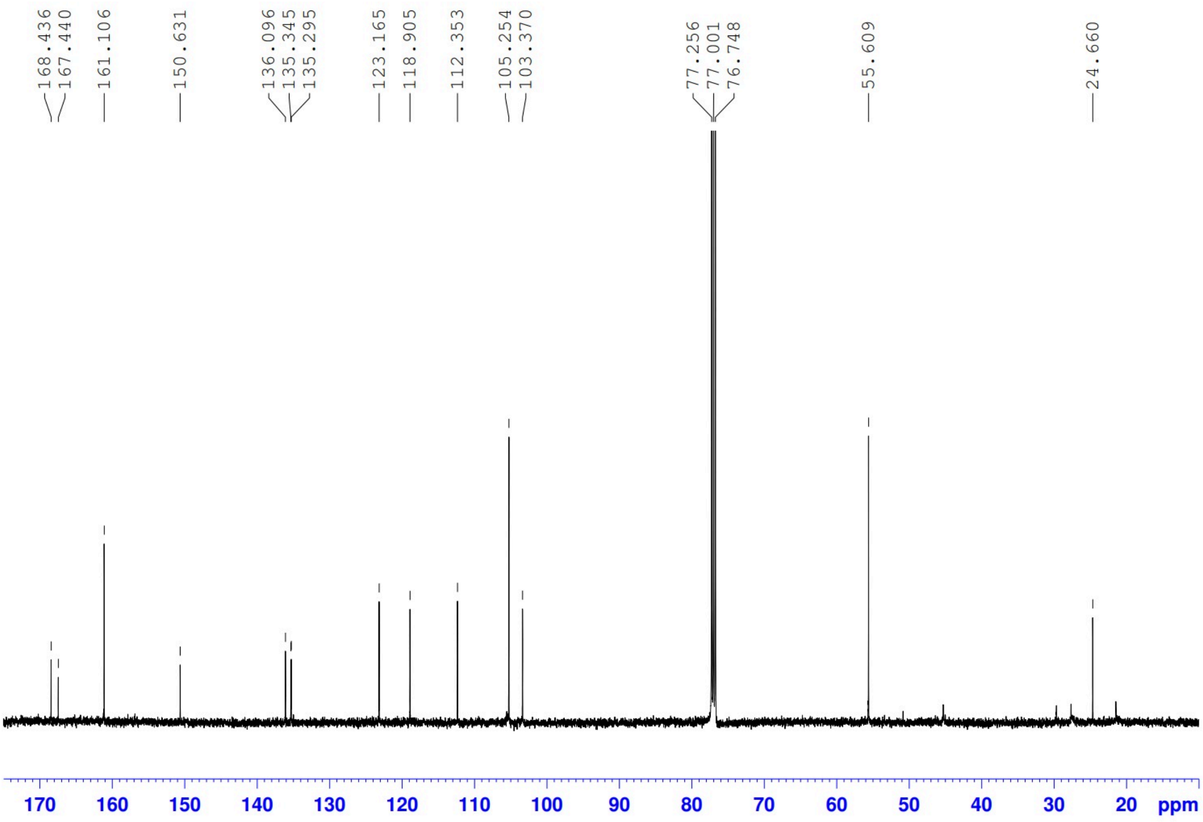

Figure S23. ^{13}**C-NMR (125 MHz, $CDCl_3$) spectrum of 12**

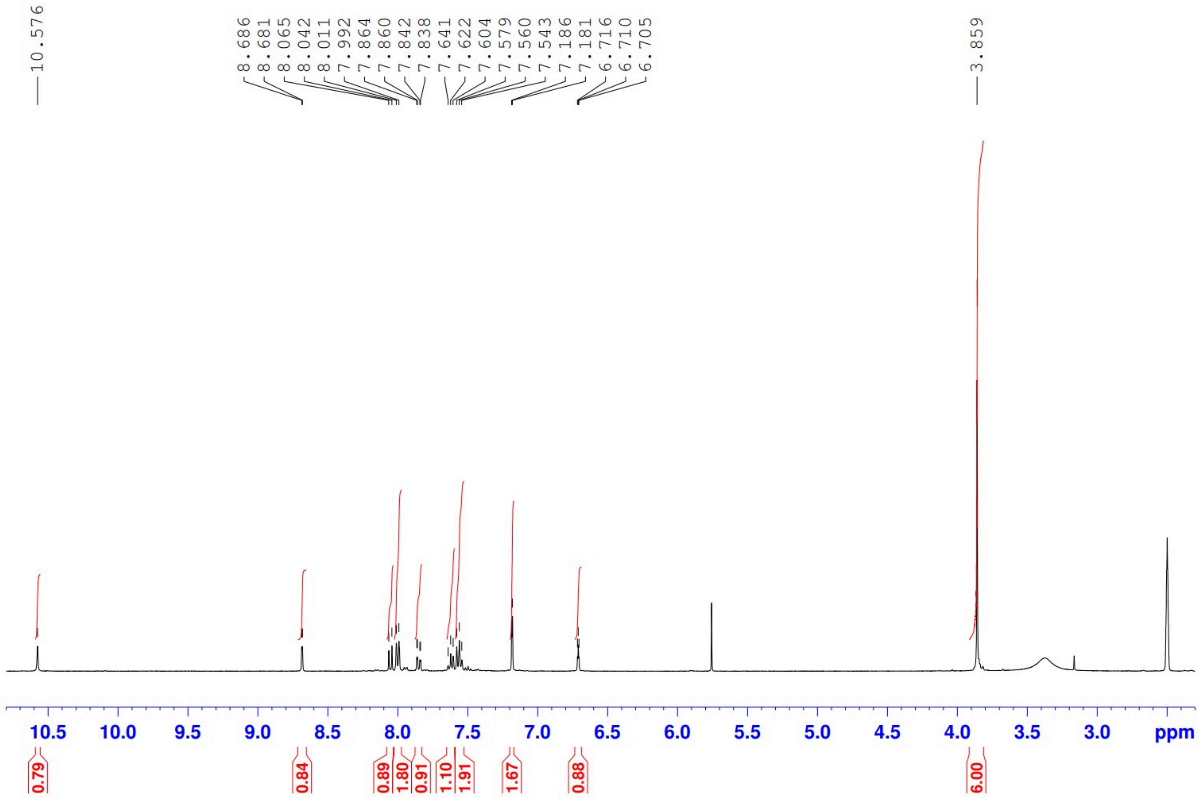

Figure S24. ^{1}**H-NMR (400 MHz, DMSO-d_6) spectrum of 13**

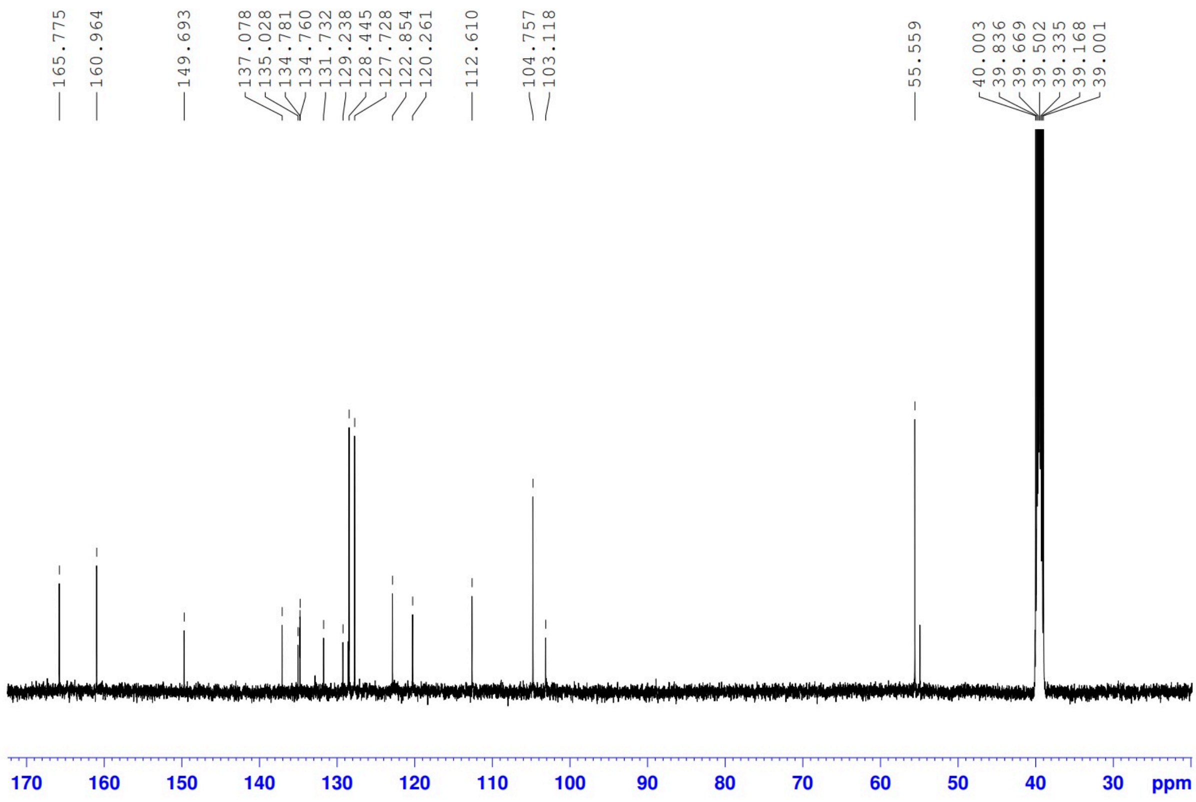

Figure S25. **^{13}C-NMR (125 MHz, DMSO-d_6) spectrum of 13**

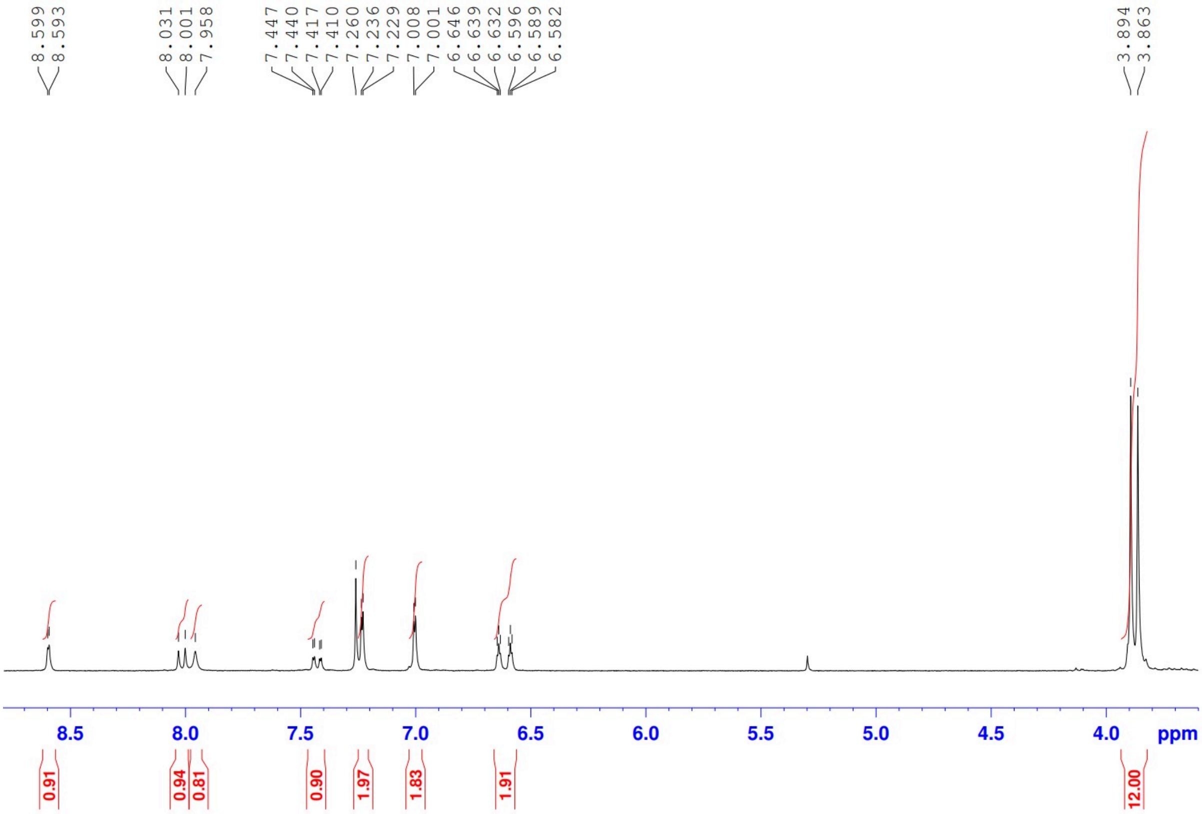

Figure S26. **^{1}H-NMR (300 MHz, $CDCl_3$) spectrum of 14**

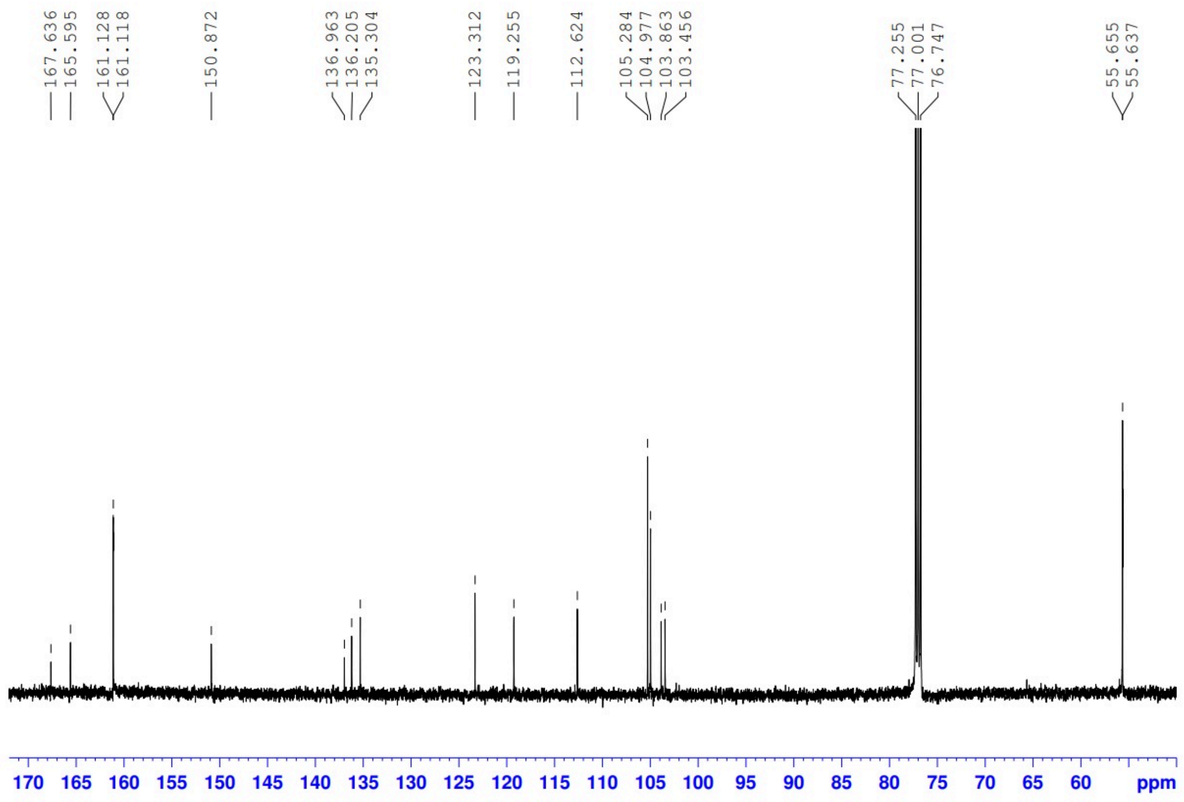

Figure S27. ^{13}C-NMR (125 MHz, $CDCl_3$) spectrum of 14

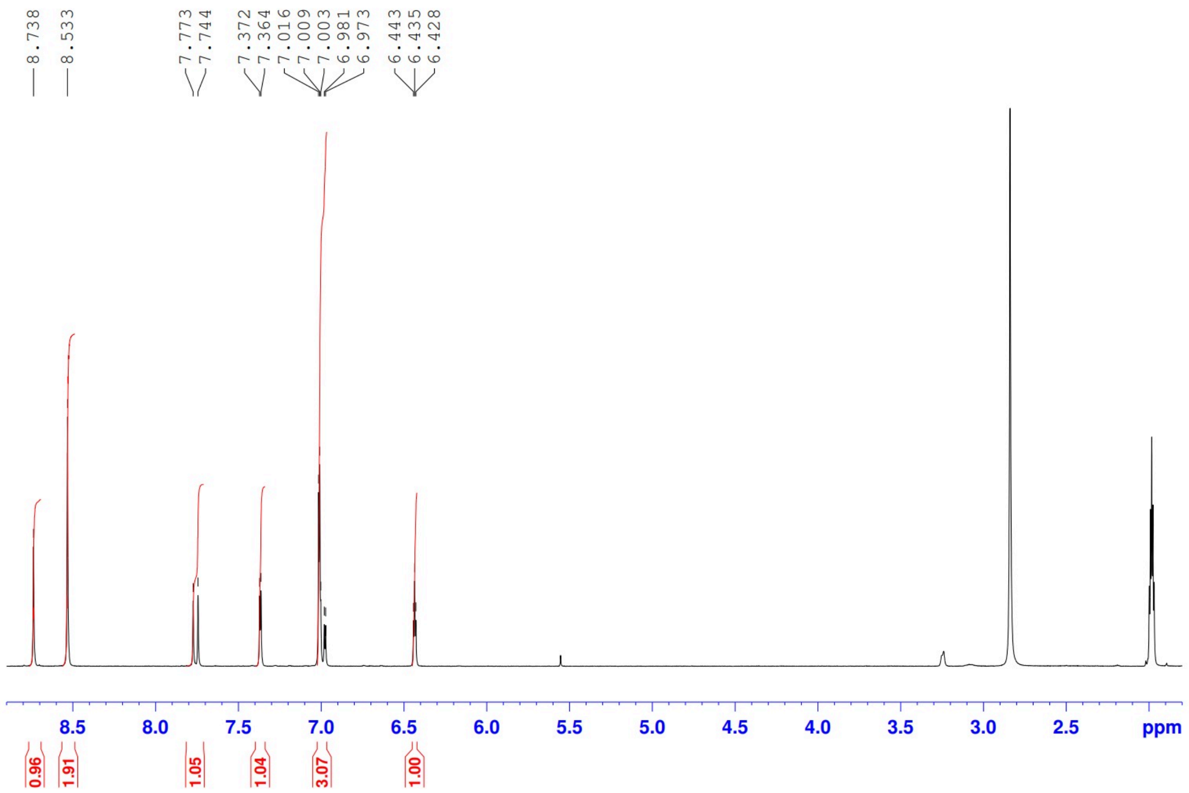

Figure S28. ^{1}H-NMR (300 MHz, acetone-d_6) spectrum of 15

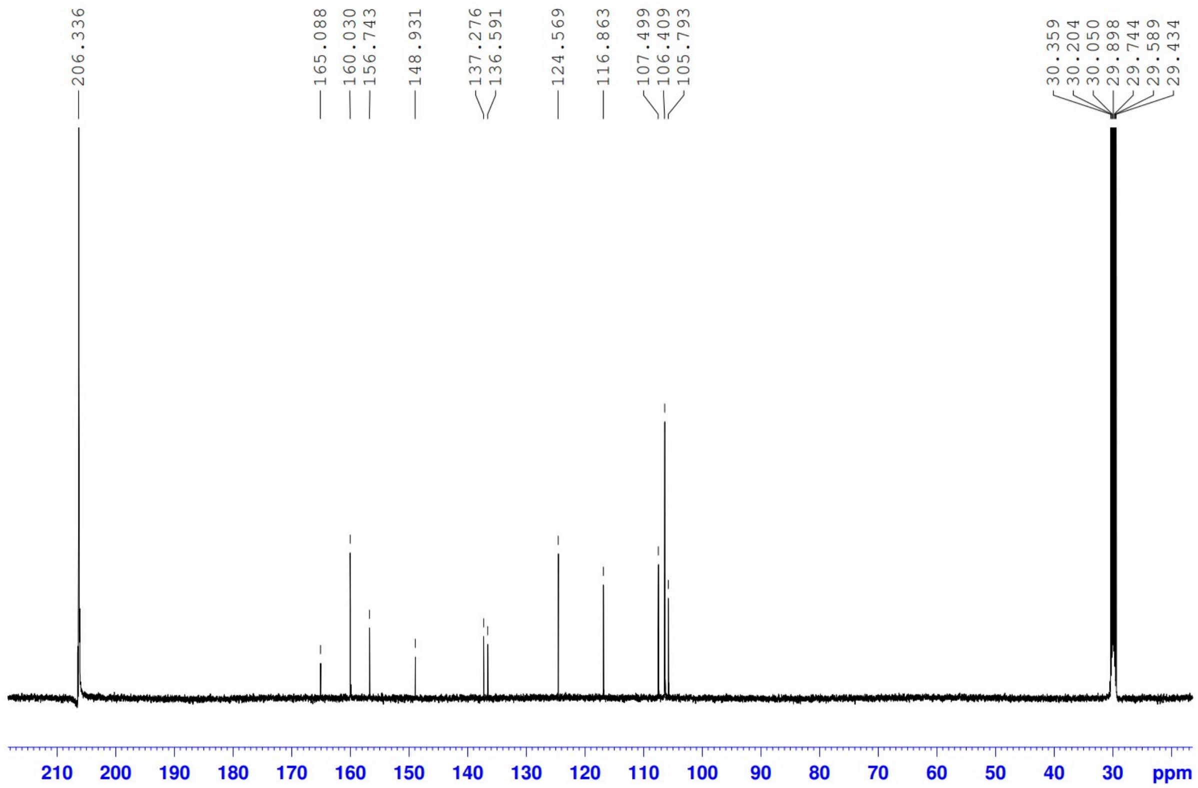

Figure S29. ^{13}**C-NMR (125 MHz, acetone-d$_6$) spectrum of 15**

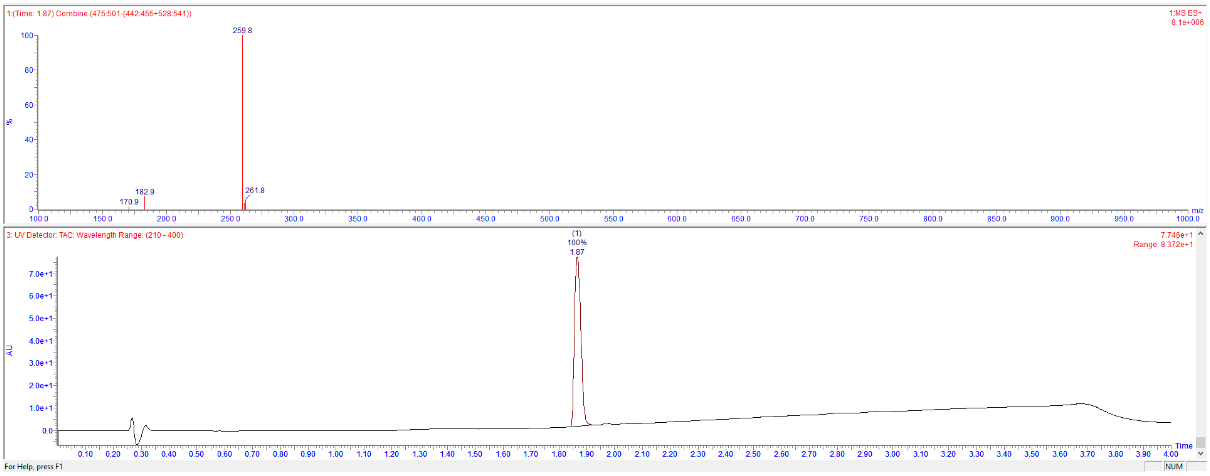

Figure S30. LCMS of 15

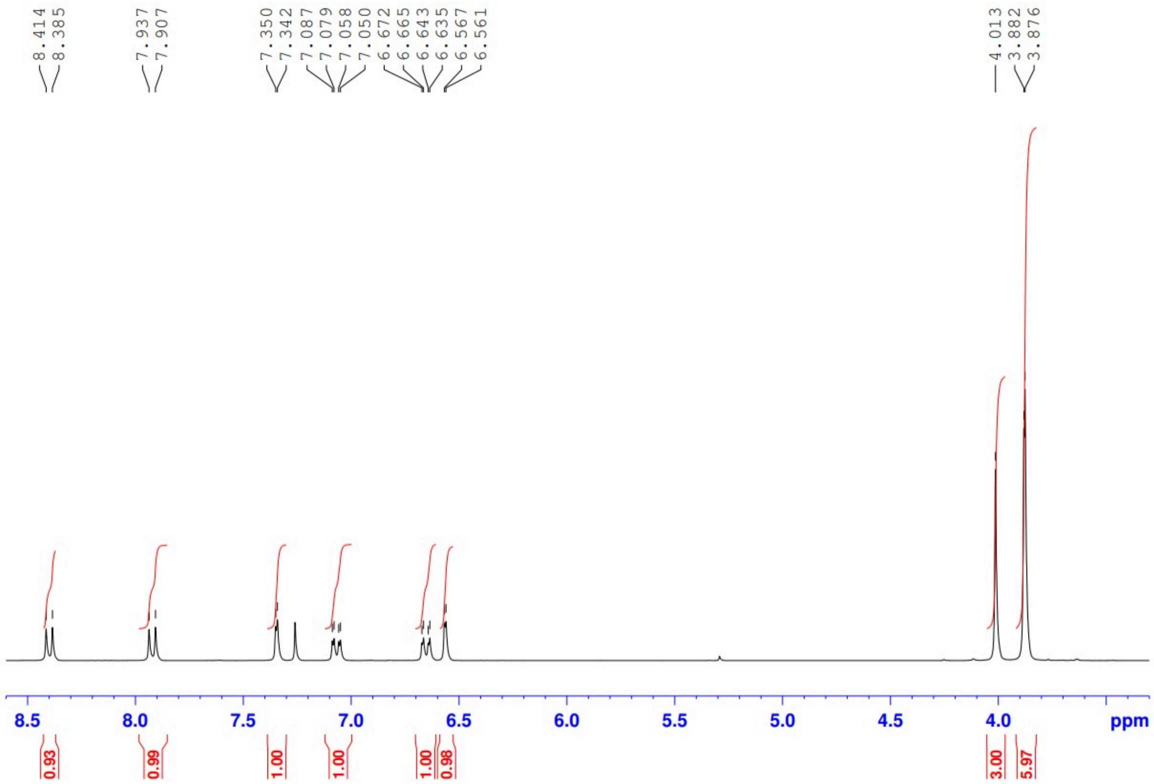

Figure S31. ^{1}H-NMR (300 MHz, $CDCl_3$) spectrum of 16

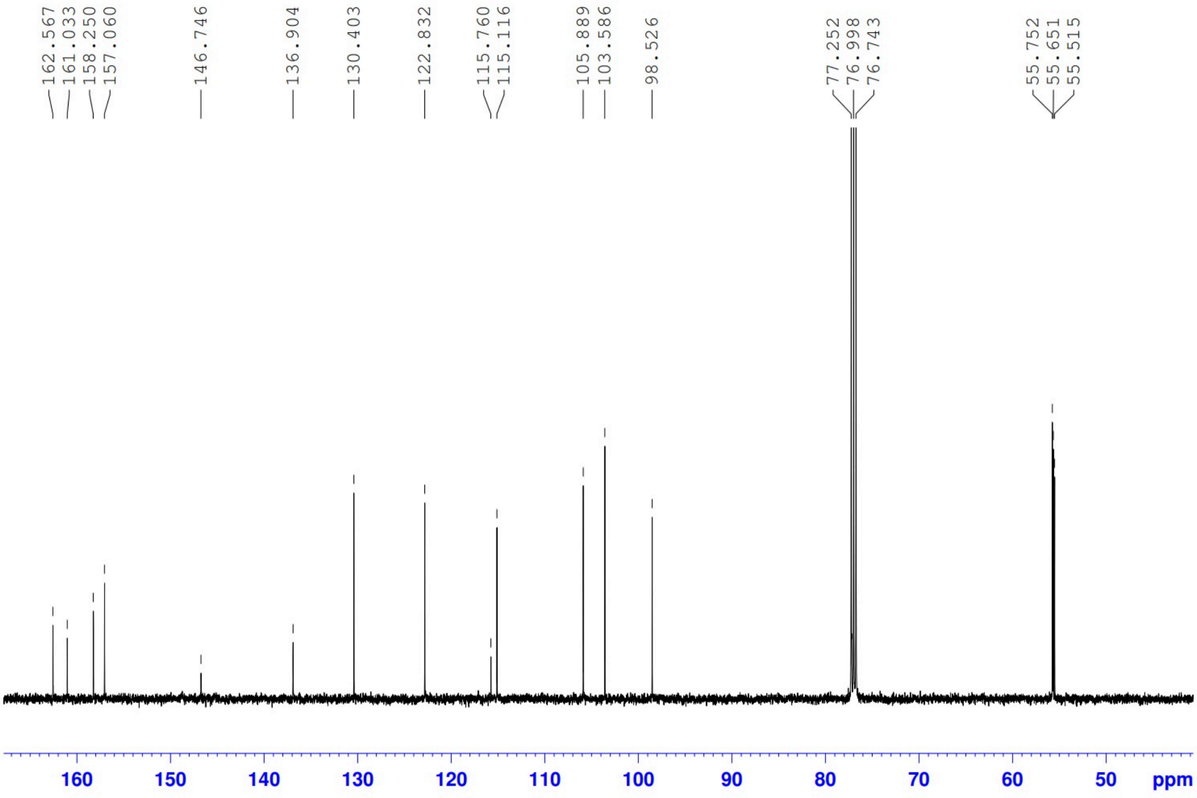

Figure S32. ^{13}C-NMR (125 MHz, $CDCl_3$) spectrum of 16

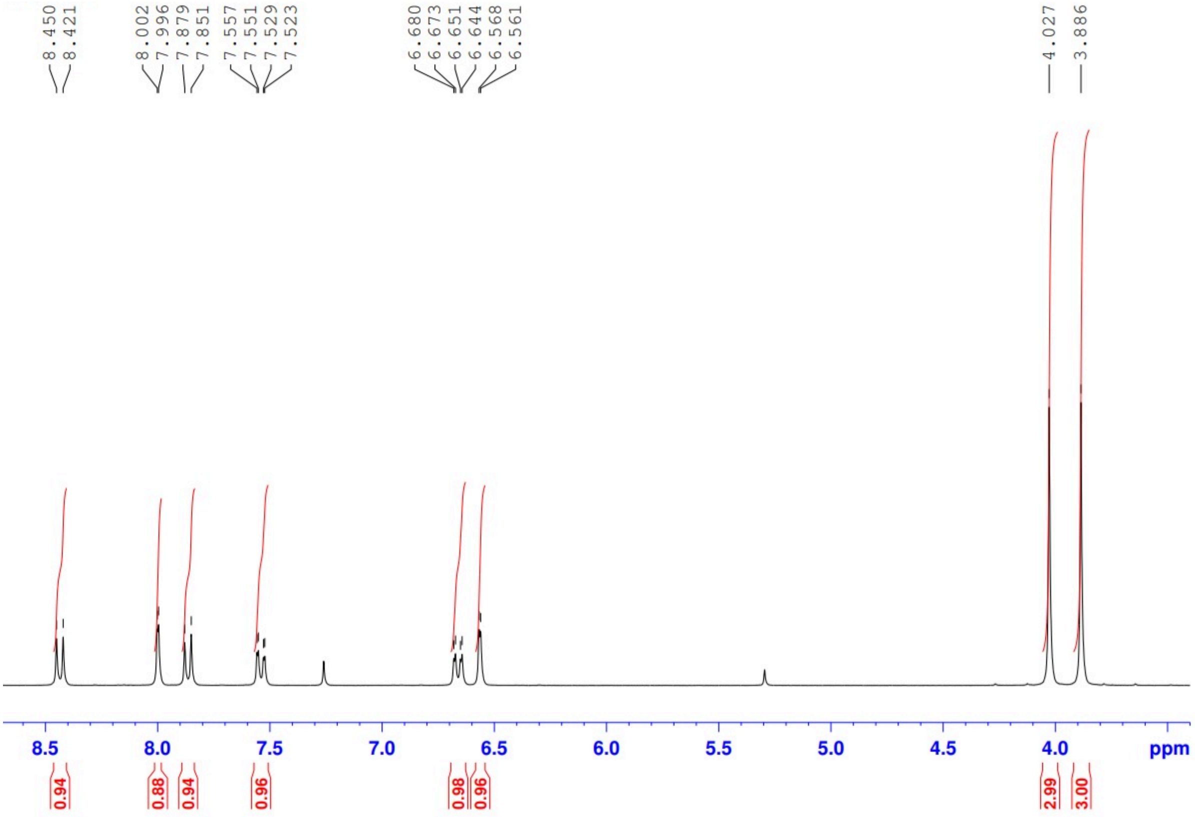

Figure S33. ^{1}H-NMR (300 MHz, $CDCl_3$) spectrum of 17

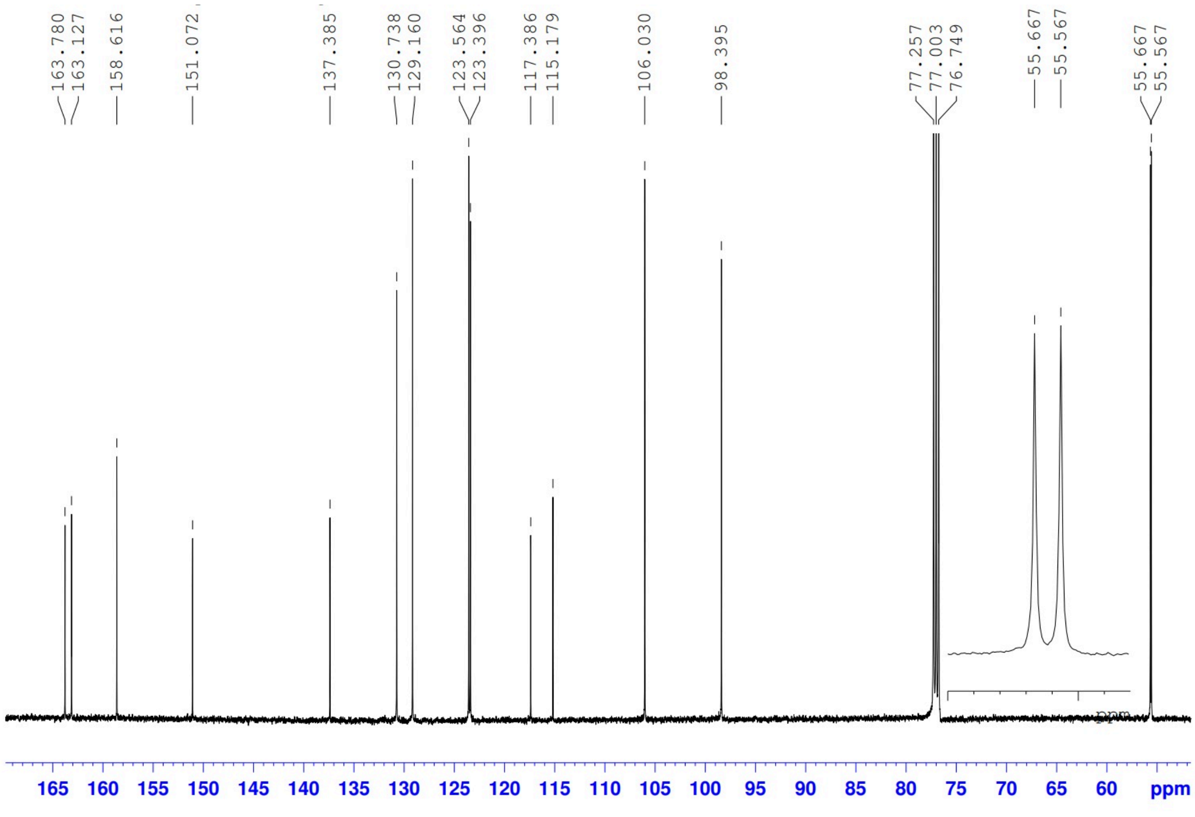

Figure S34. ^{13}C-NMR (125 MHz, $CDCl_3$) spectrum of 17

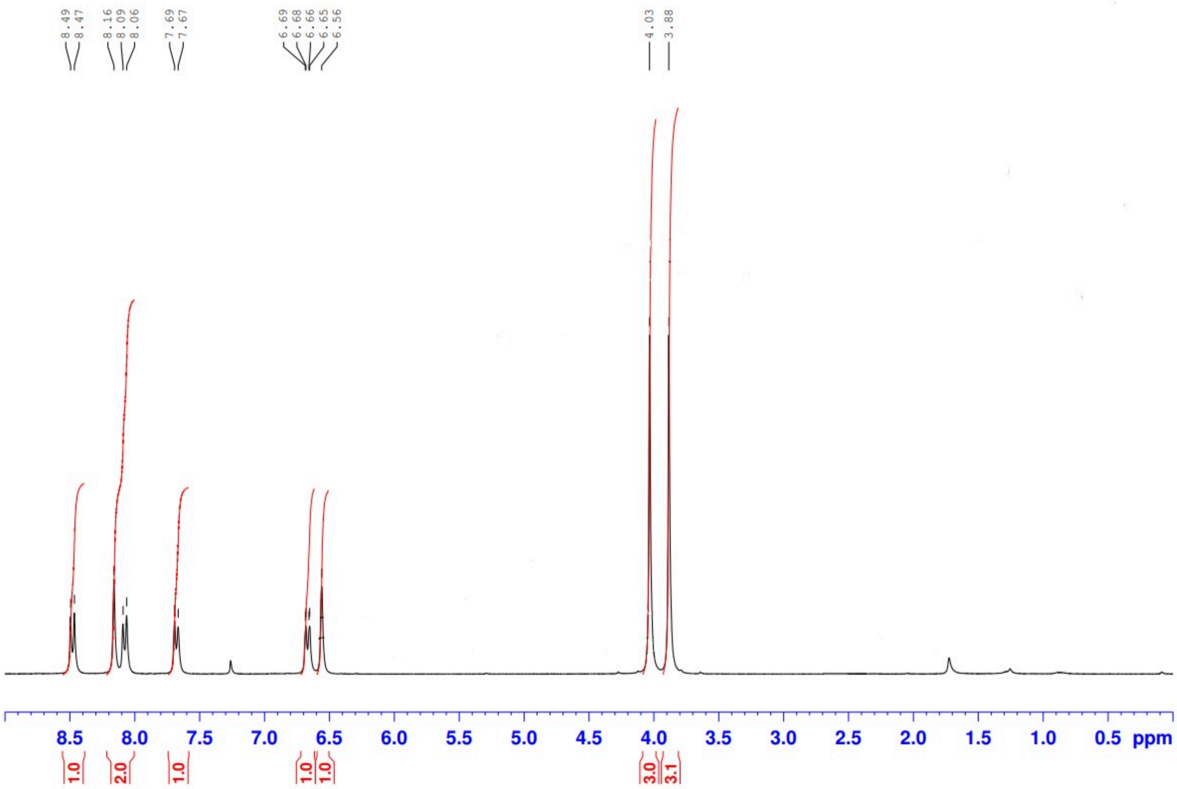

Figure S35. ¹H-NMR (300 MHz, $CDCl_3$) spectrum of 18

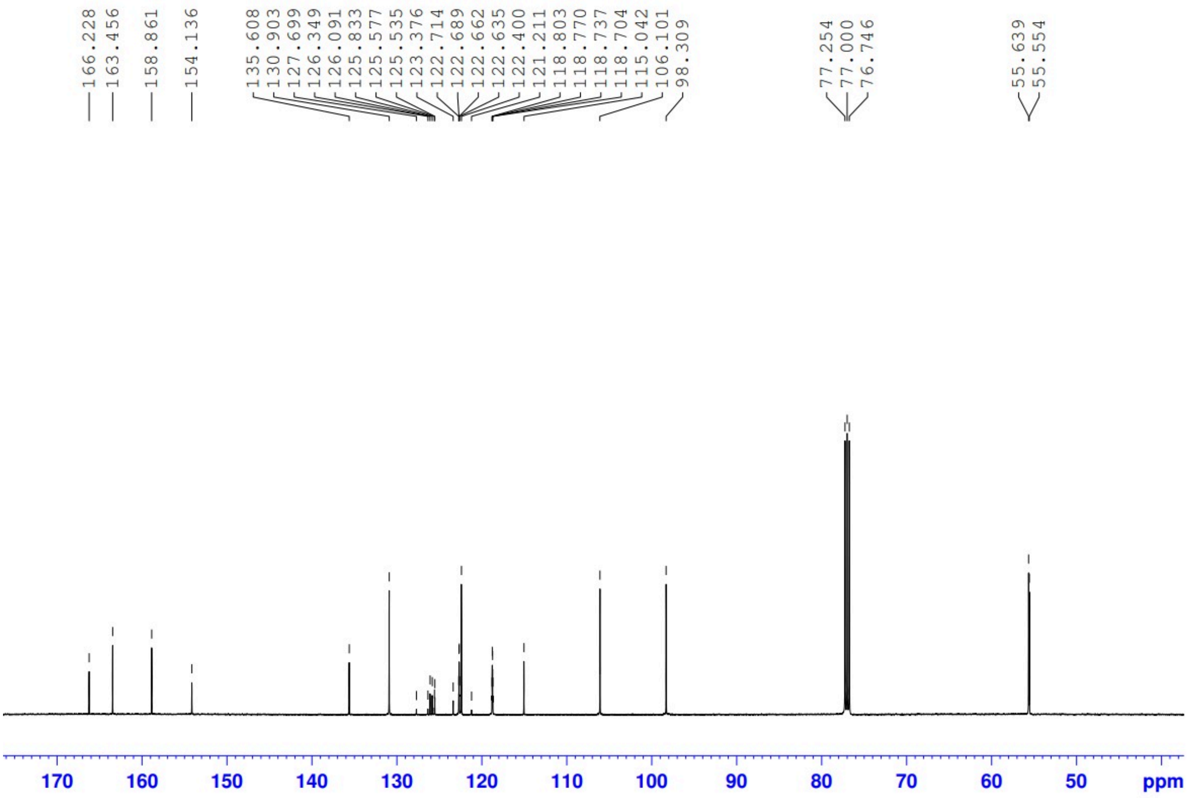

Figure S36. ¹³C-NMR (125 MHz, $CDCl_3$) spectrum of 18

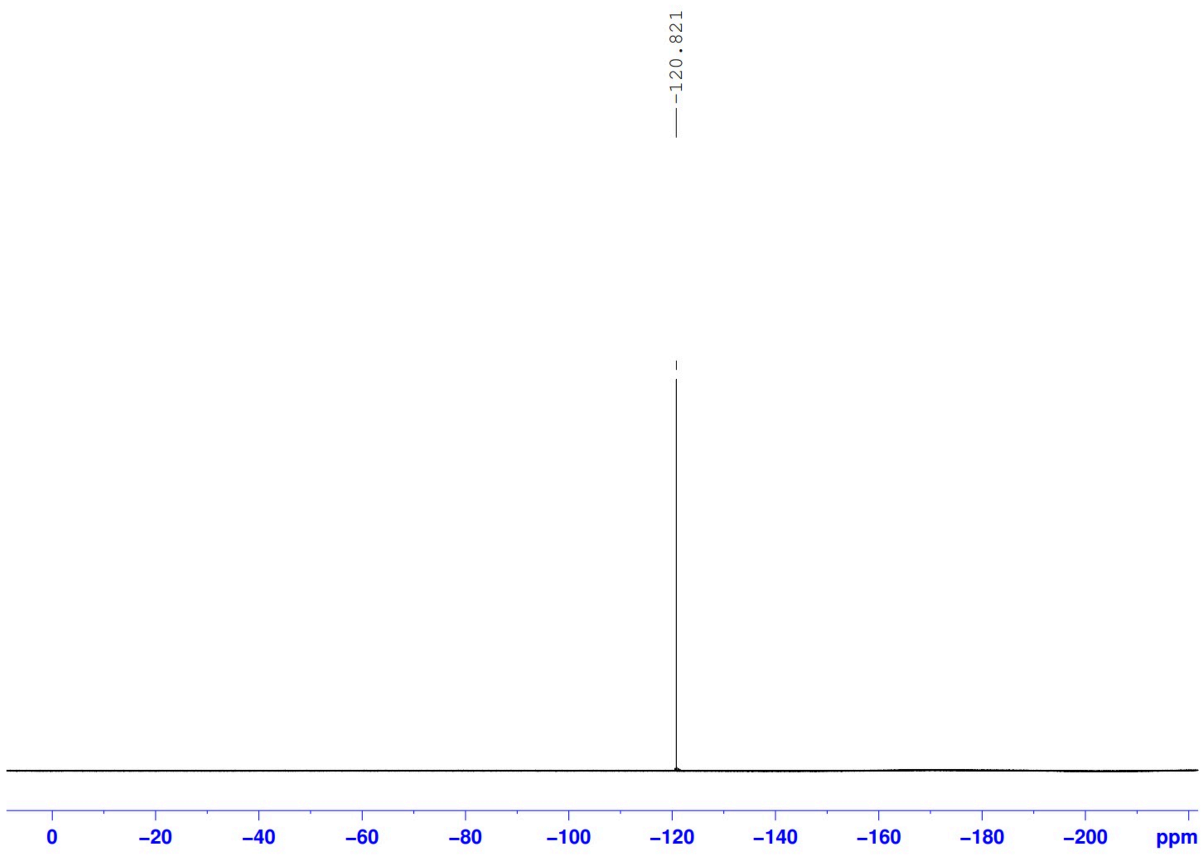

Figure S37. ^{19}F-NMR (376 MHz, $CDCl_3$) spectrum of 18

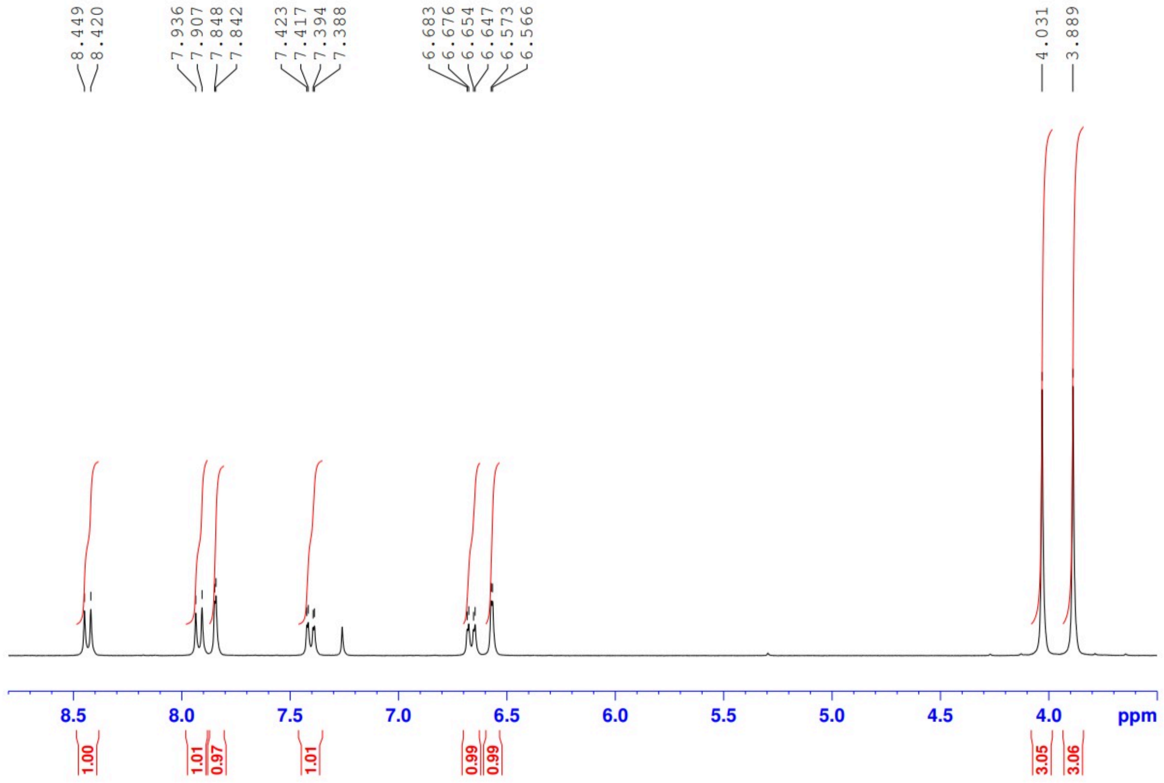

Figure S38. ^{1}H-NMR (300 MHz, $CDCl_3$) spectrum of 19

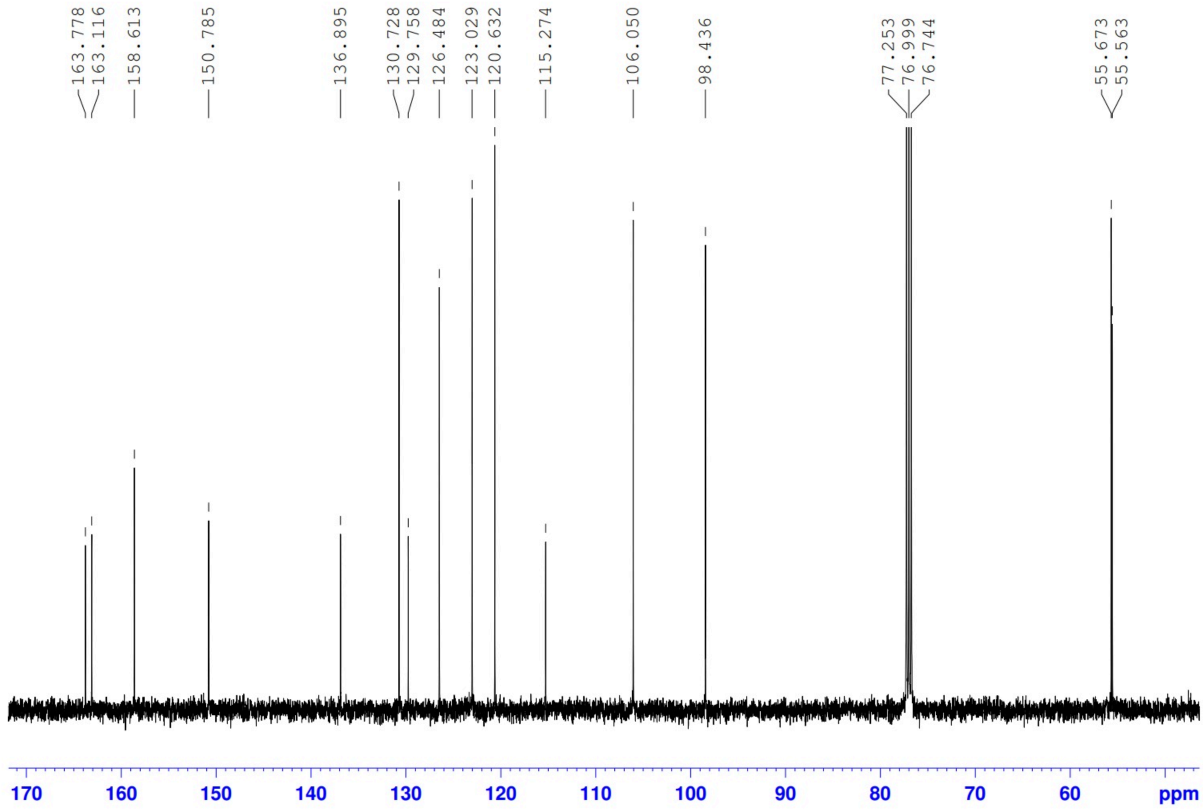

Figure S39. ^{13}**C-NMR (125 MHz, CDCl$_3$) spectrum of 19**

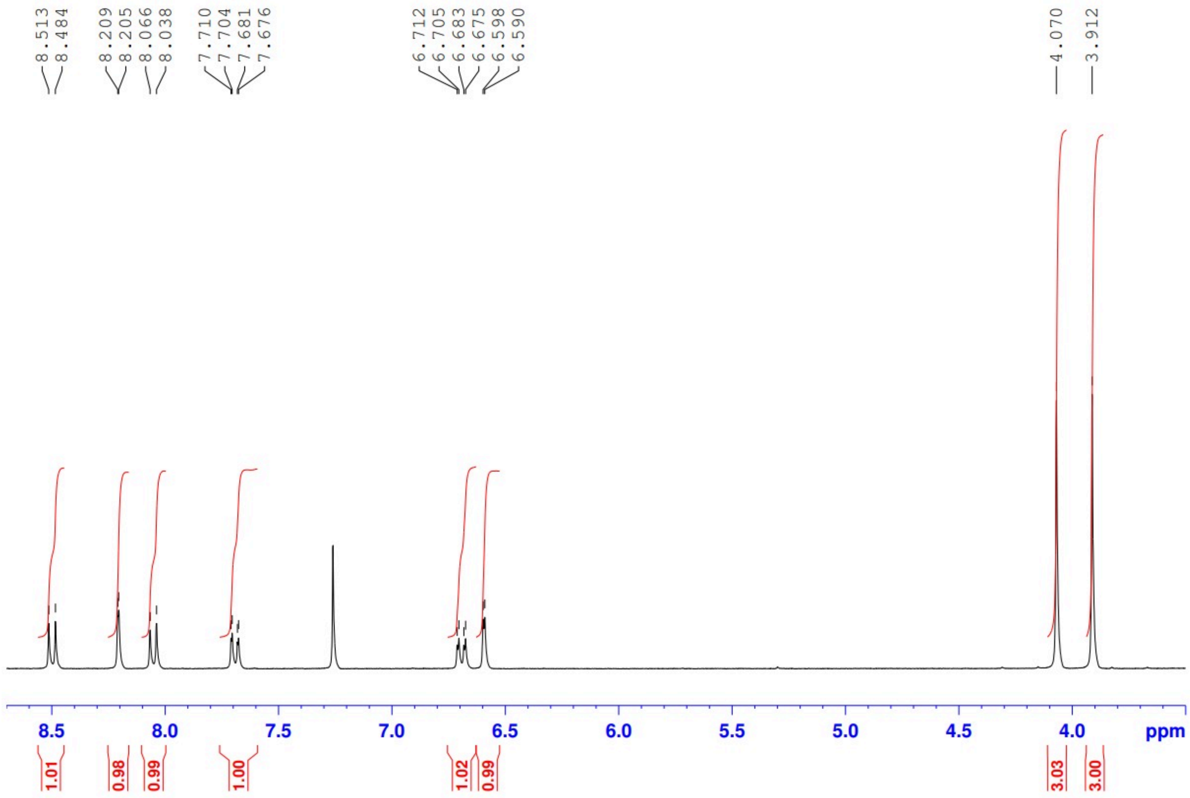

Figure S40. ^{1}**H-NMR (300 MHz, CDCl$_3$) spectrum of 20**

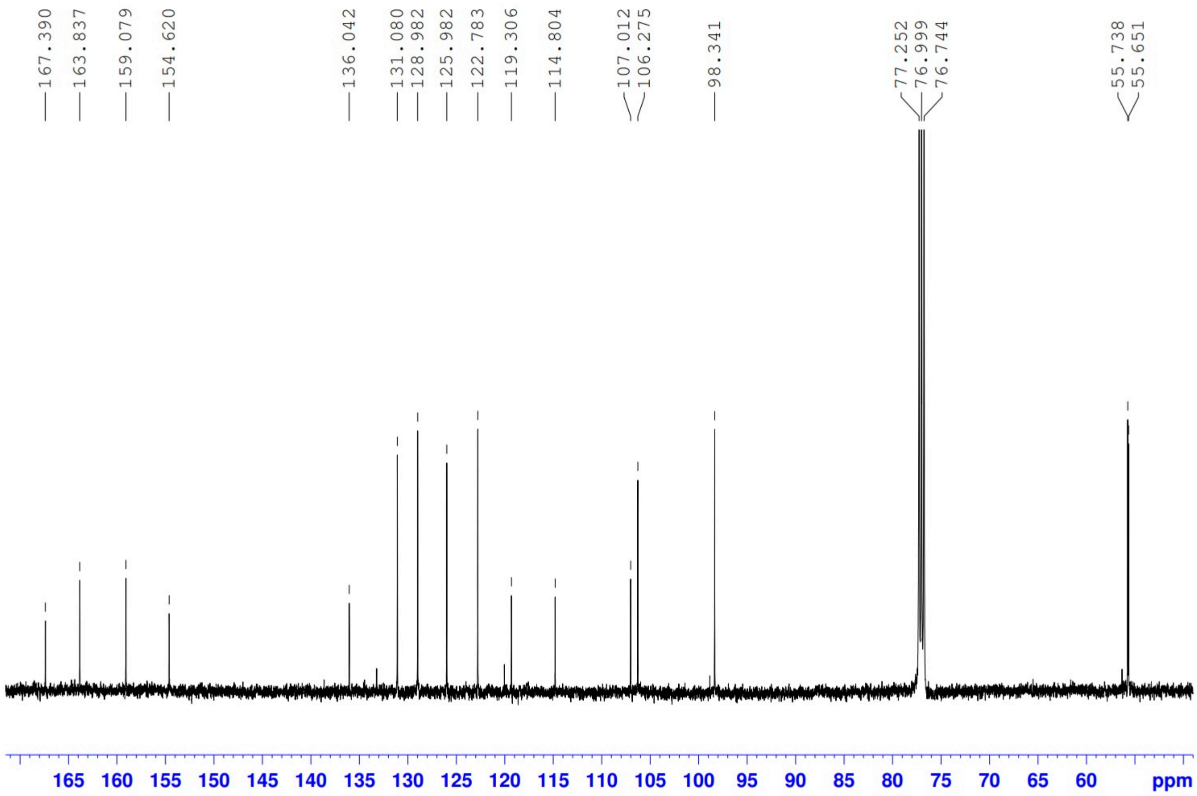

Figure S41. ^{13}**C-NMR (125 MHz, CDCl$_3$) spectrum of 20**

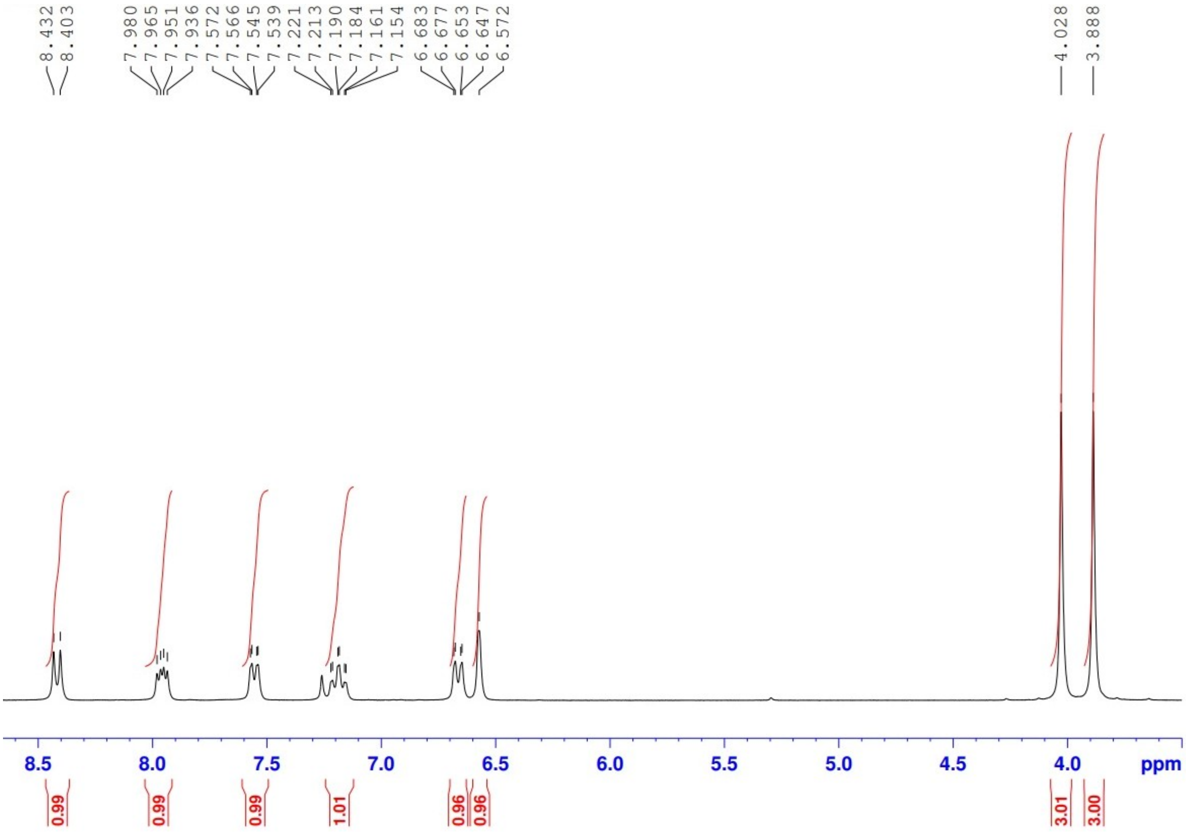

Figure S42. ^{1}**H-NMR (300 MHz, CDCl$_3$) spectrum of 21**

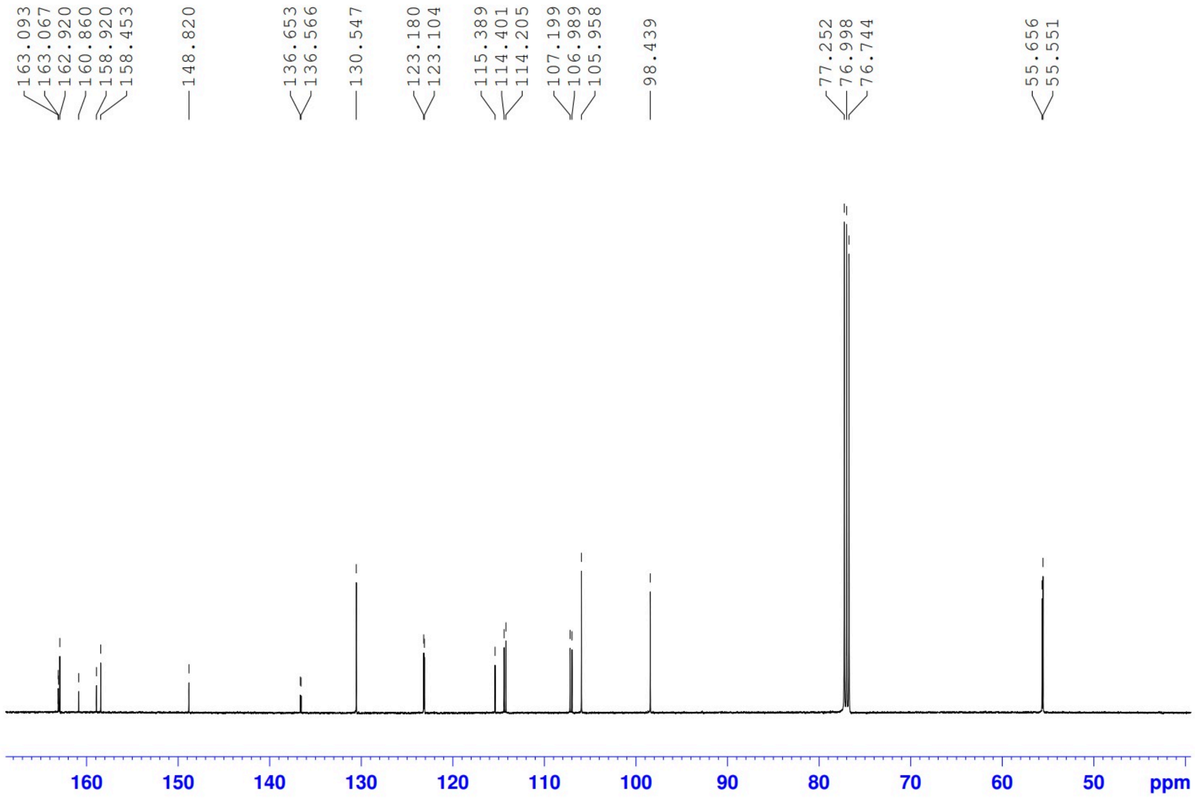

Figure S43. ^{13}C-NMR (125 MHz, $CDCl_3$) spectrum of 21

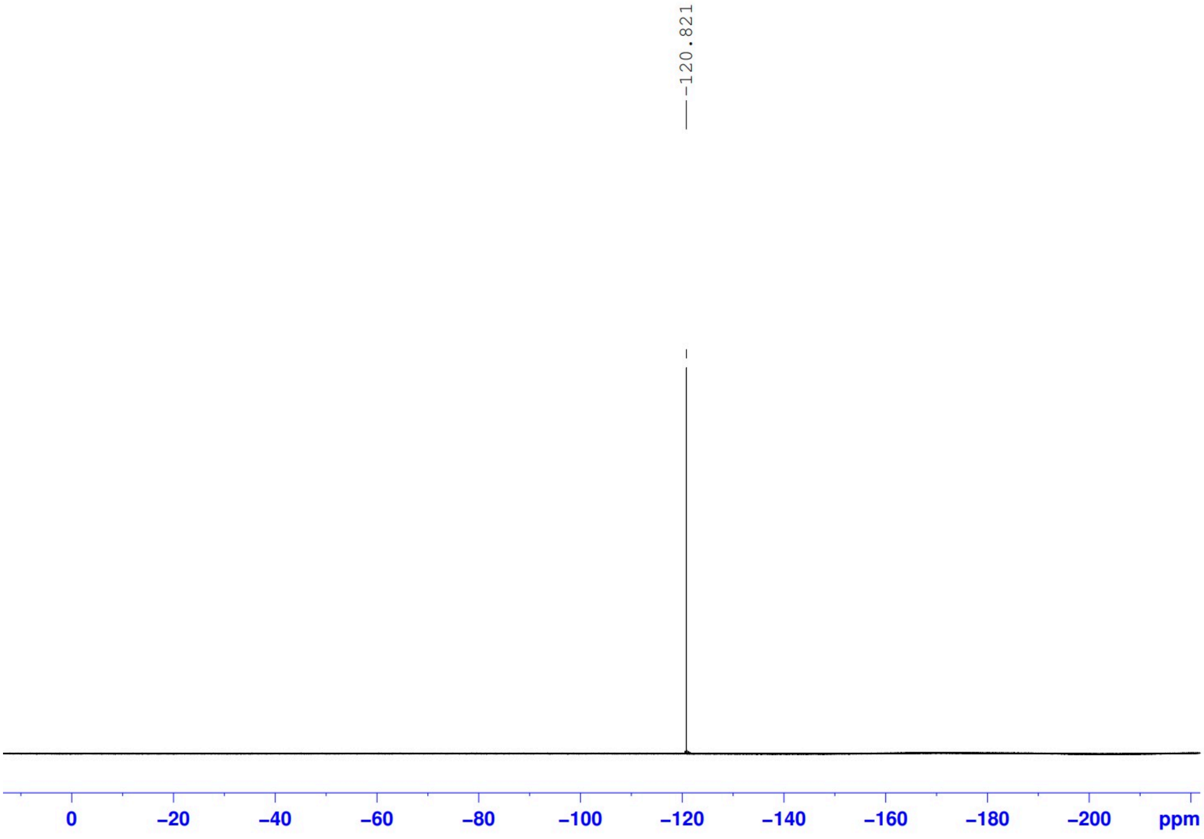

Figure S44.^{19}F-NMR (376 MHz, $CDCl_3$) spectrum of 21

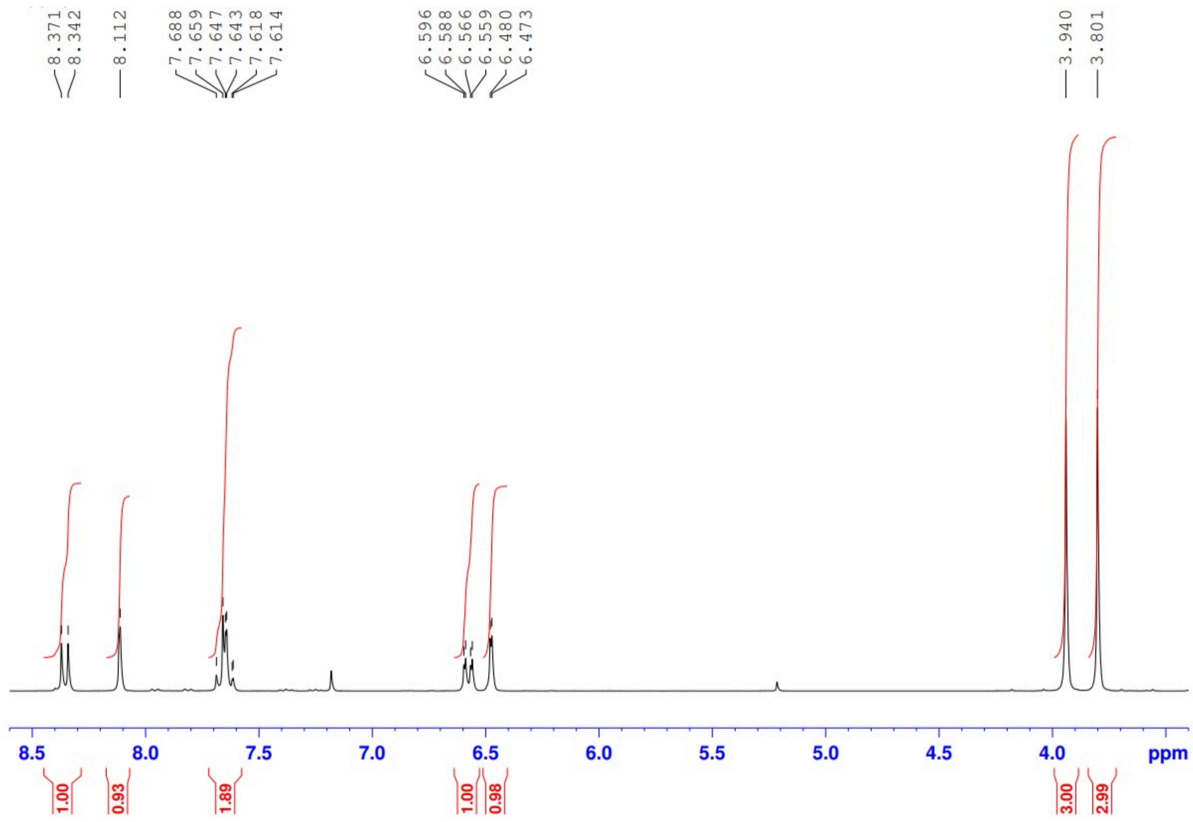

Figure S45. ^{1}**H-NMR (300 MHz, CDCl$_3$) spectrum of 22**

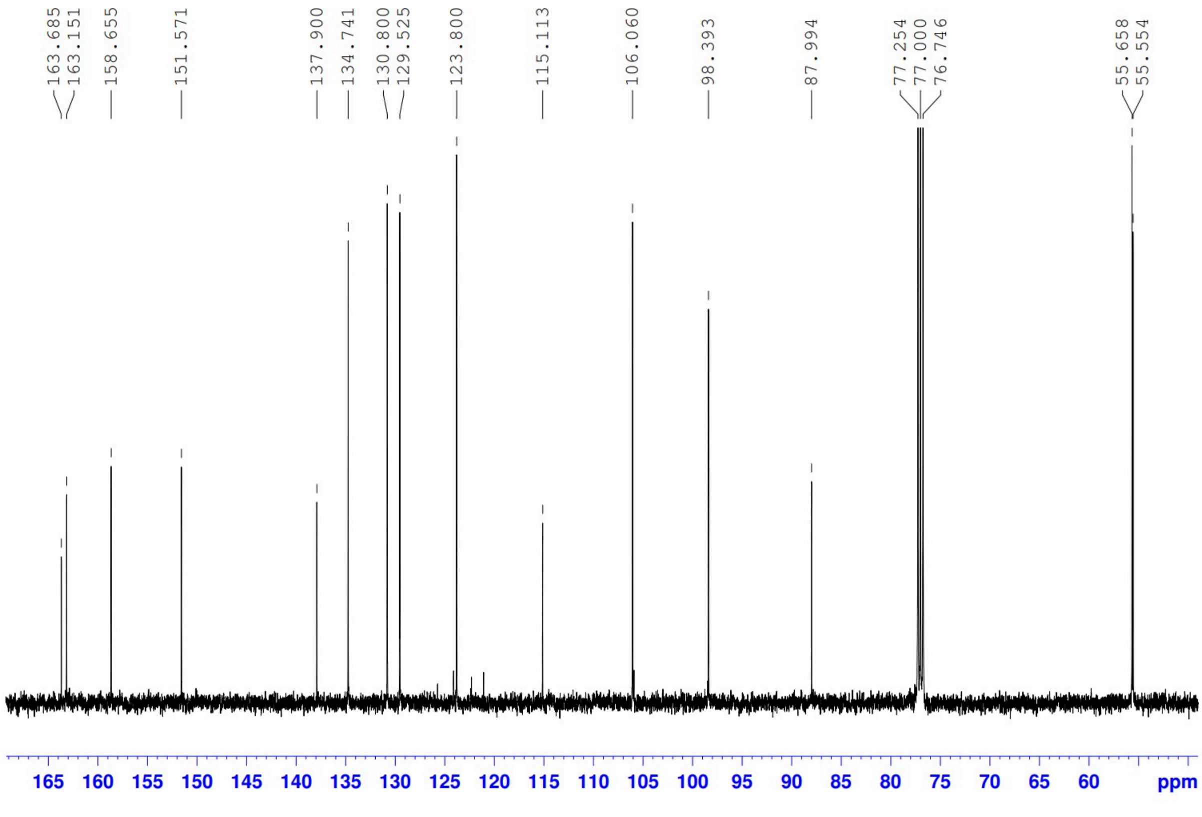

Figure S46. ^{13}**C-NMR (125 MHz, CDCl$_3$) spectrum of 22**

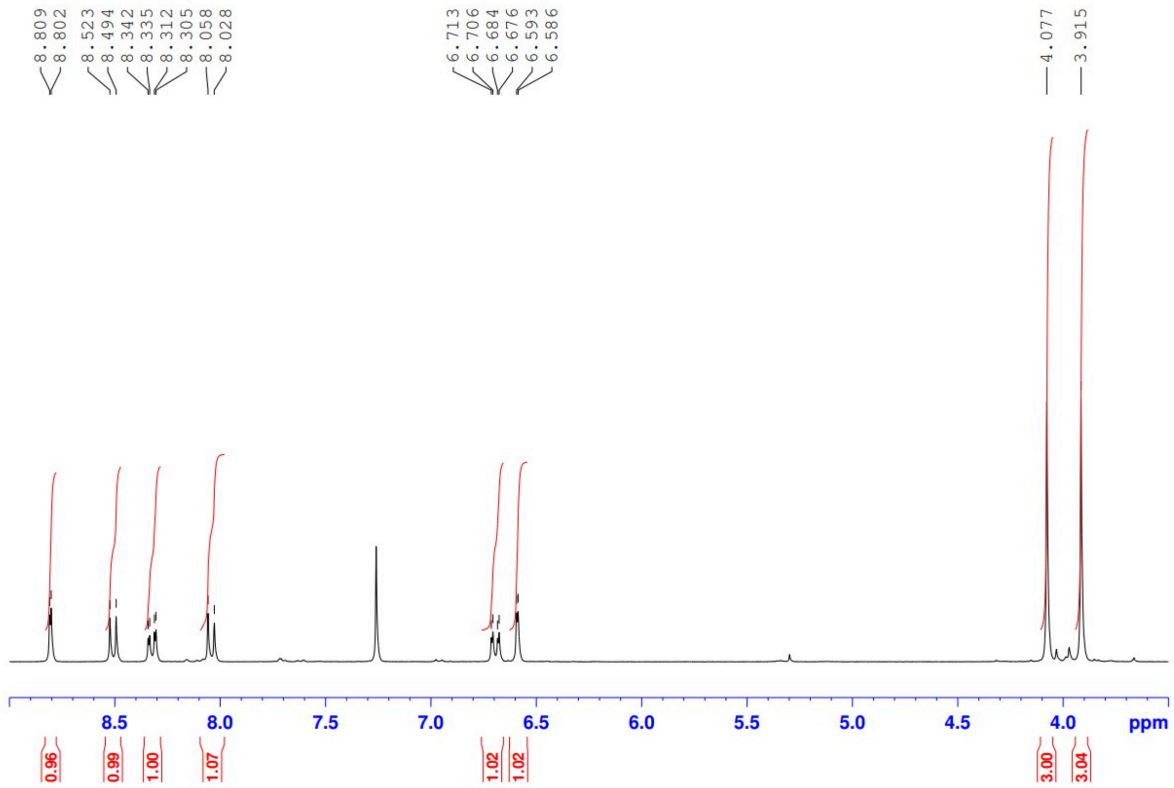

Figure S47. **^{1}H-NMR (300 MHz, CDCl$_3$) spectrum of 23**

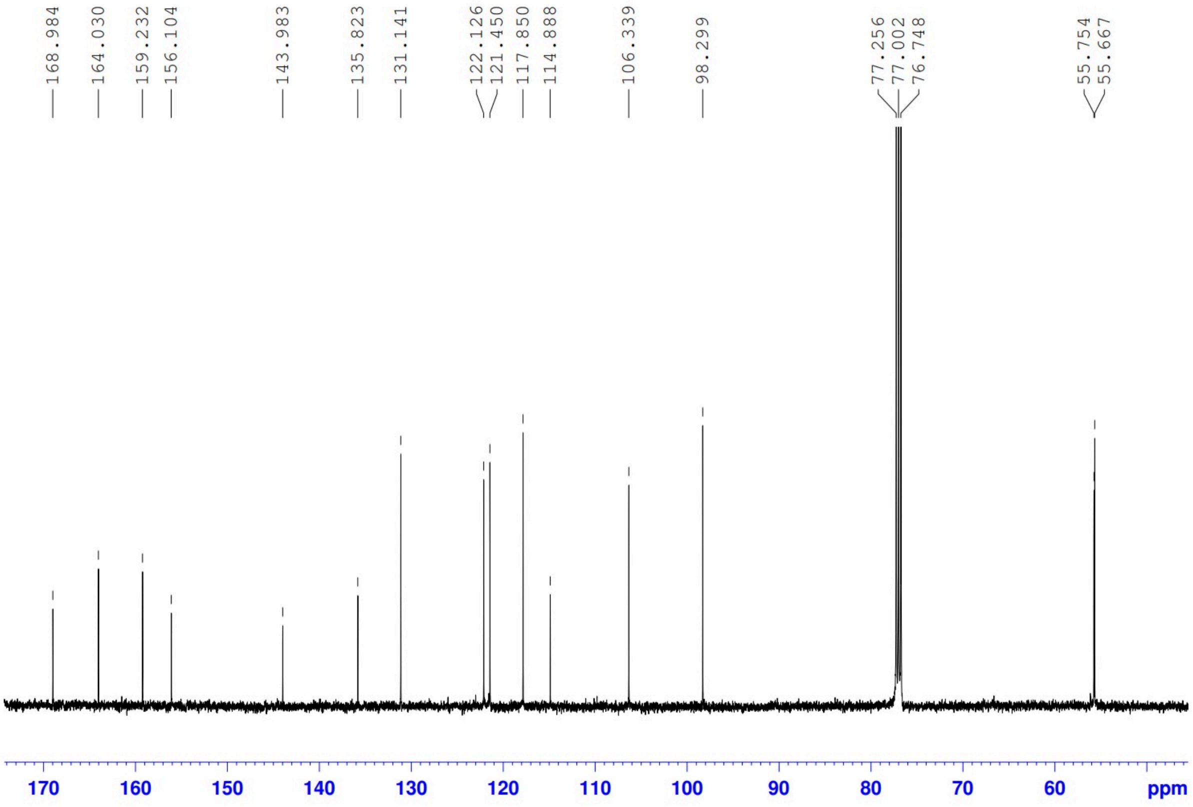

Figure S48. **^{13}C-NMR (125 MHz, CDCl$_3$) spectrum of 23**

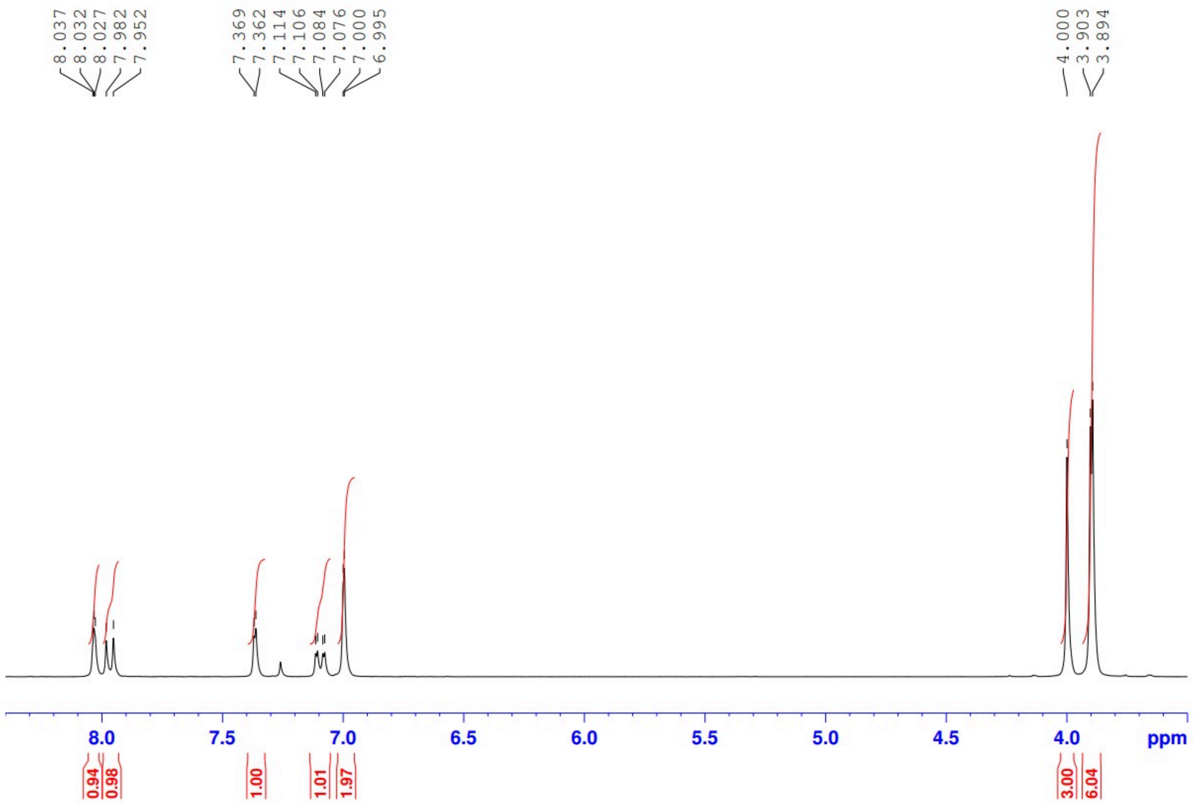

Figure S49. ^{1}**H-NMR (300 MHz, CDCl**$_3$**) spectrum of 24**

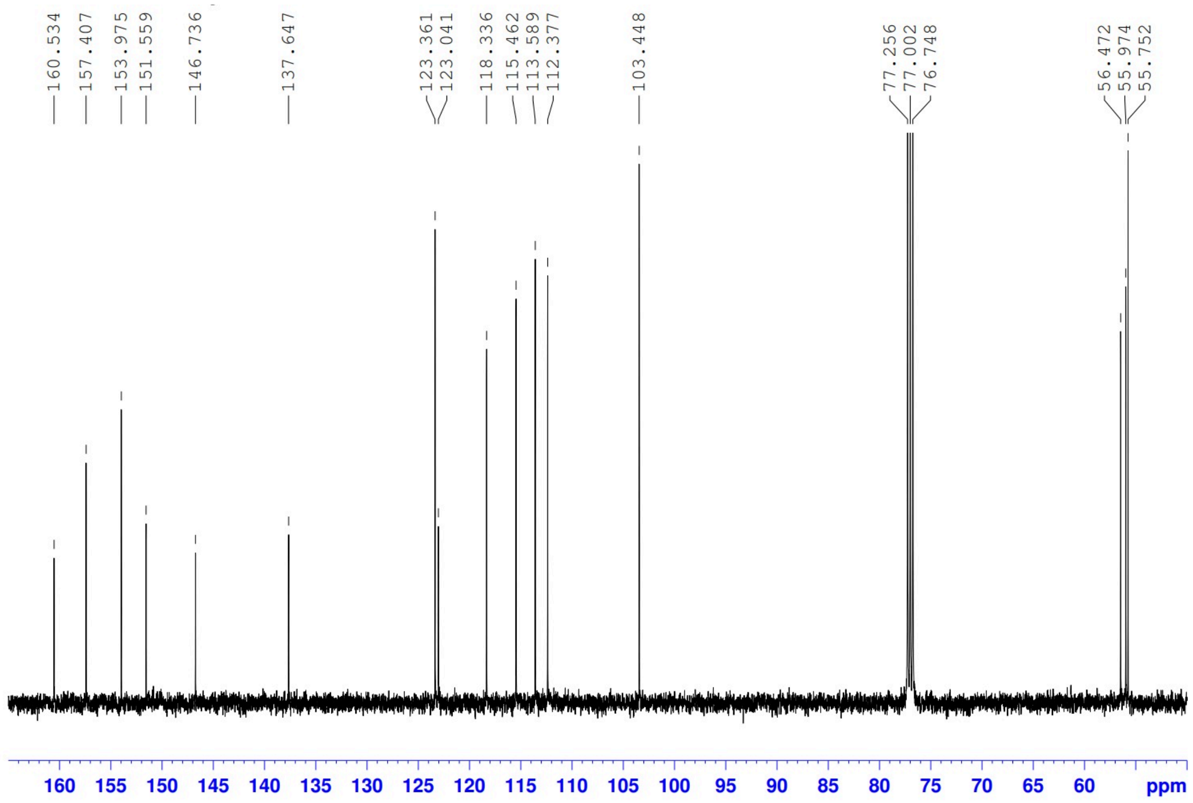

Figure S50. ^{13}**C-NMR (125 MHz, CDCl**$_3$**) spectrum of 24**

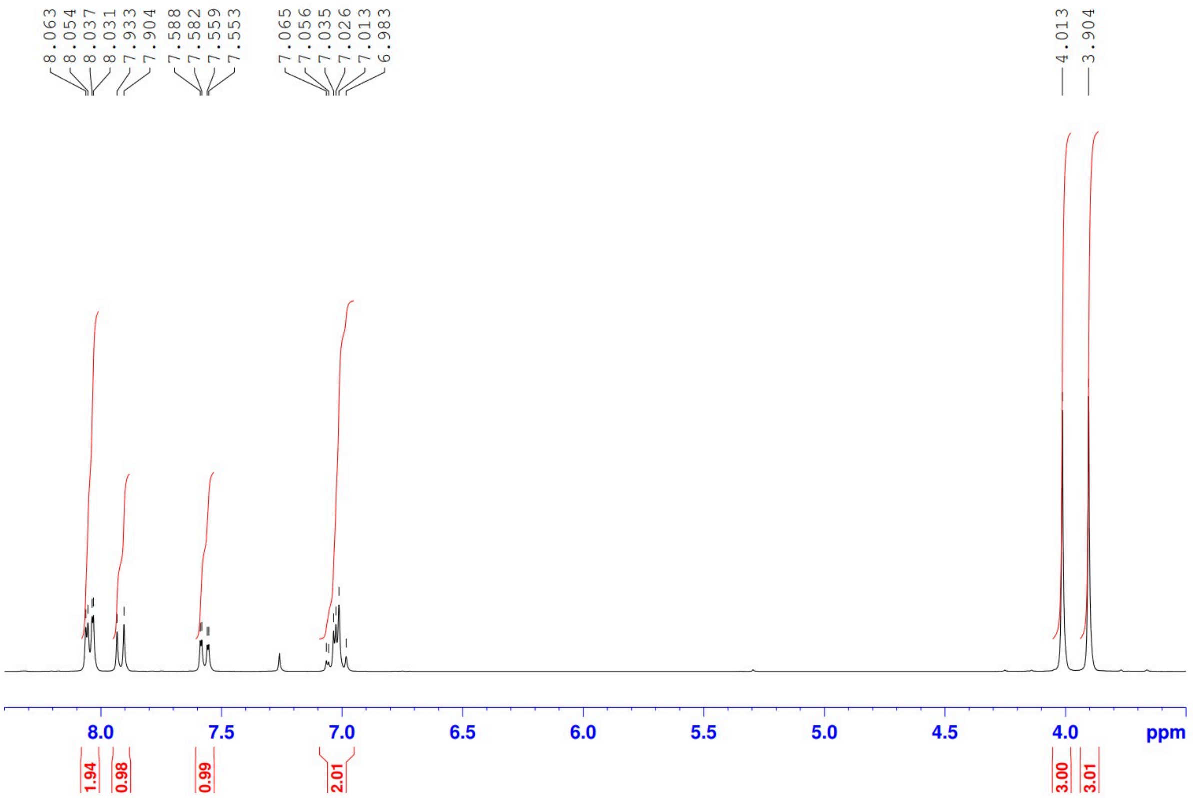

Figure S51. **^{1}H-NMR (300 MHz, $CDCl_3$) spectrum of 25**

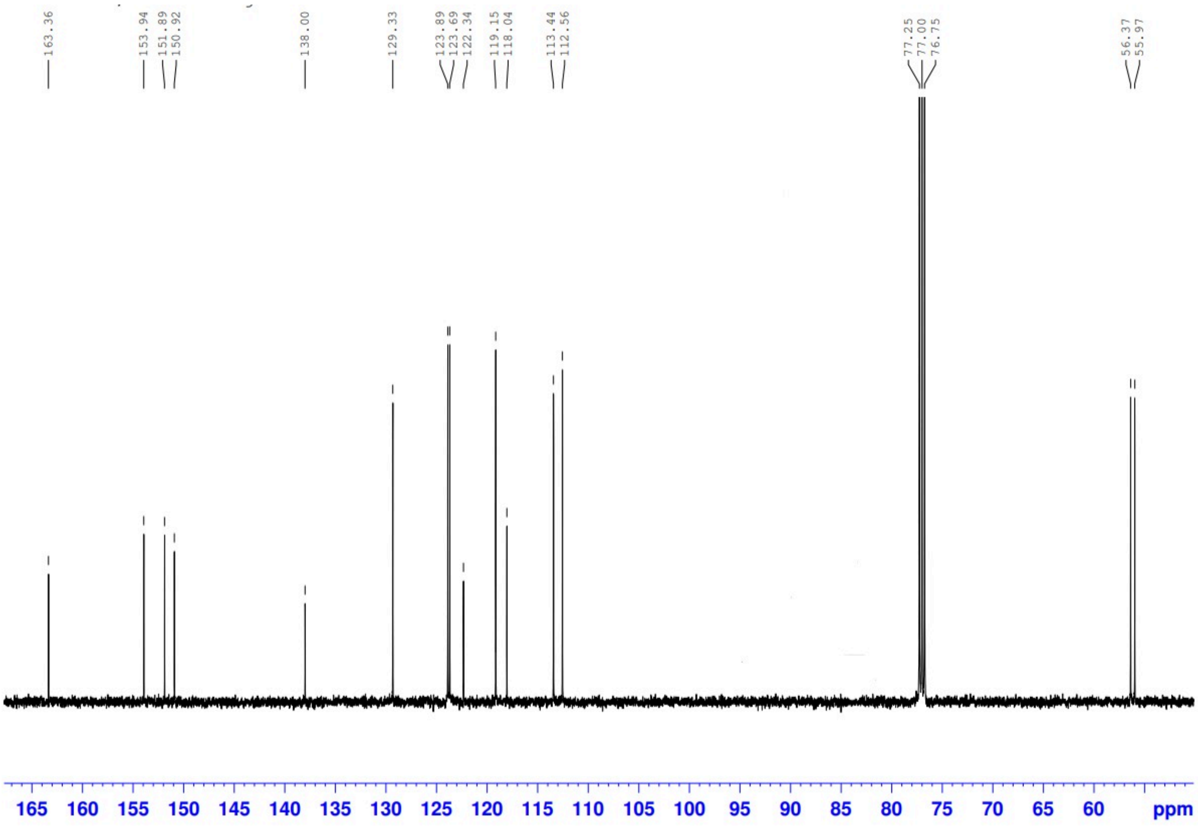

Figure S52. **^{13}C-NMR (125 MHz, $CDCl_3$) spectrum of 25**

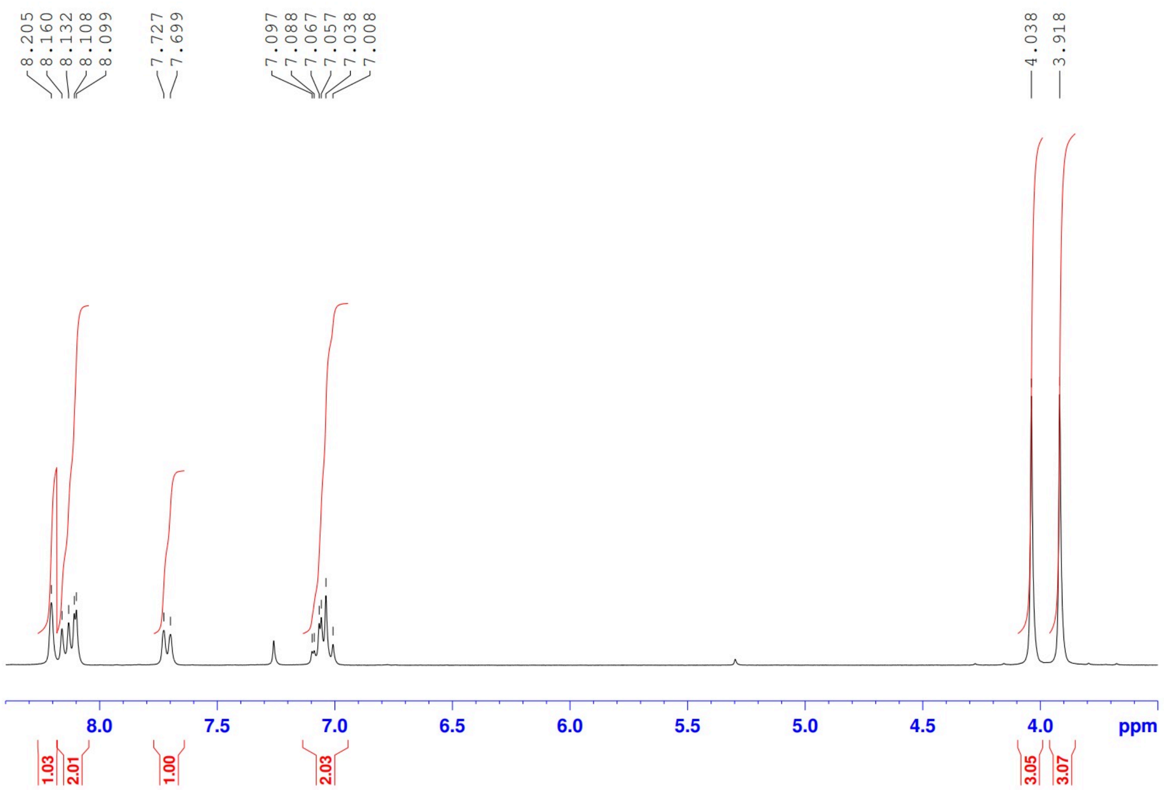

Figure S53. ^{1}**H-NMR (300 MHz, CDCl$_3$) spectrum of 26**

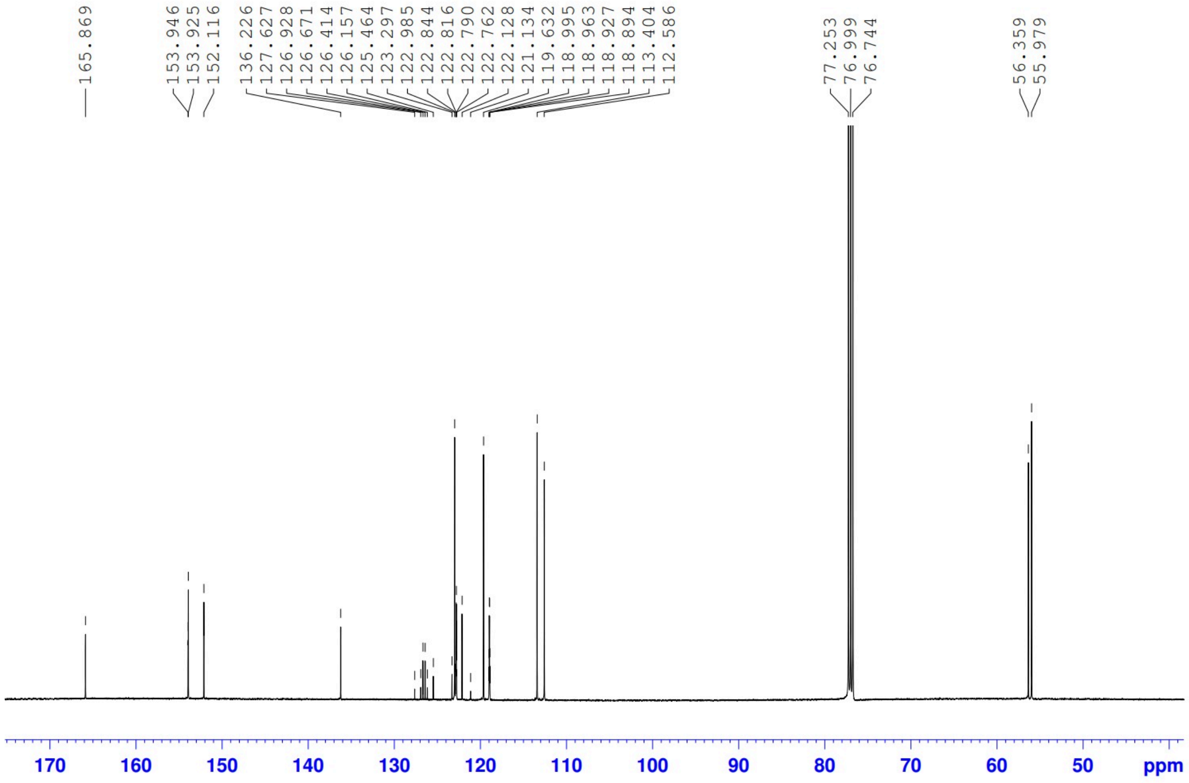

Figure S54. ^{13}**C-NMR (125 MHz, CDCl$_3$) spectrum of 26**

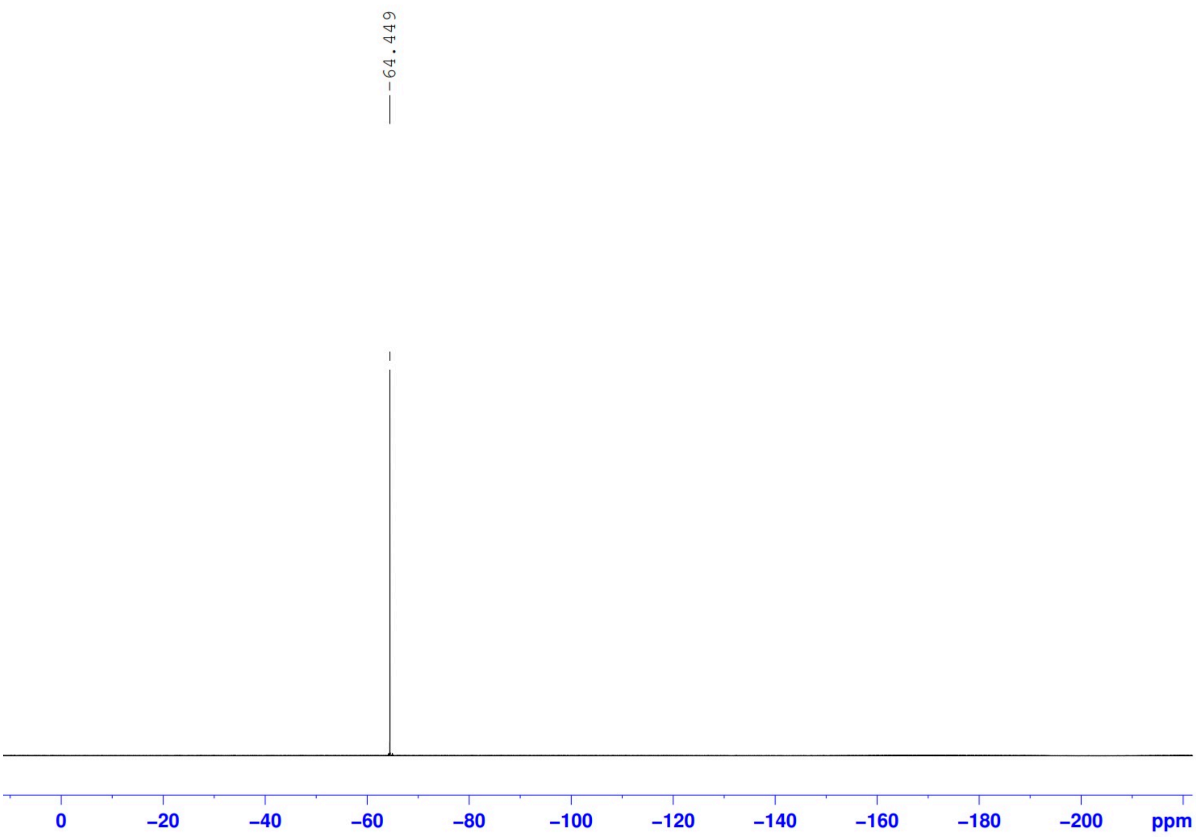

Figure S55. ^{19}**F-NMR (376 MHz, $CDCl_3$) spectrum of 26**

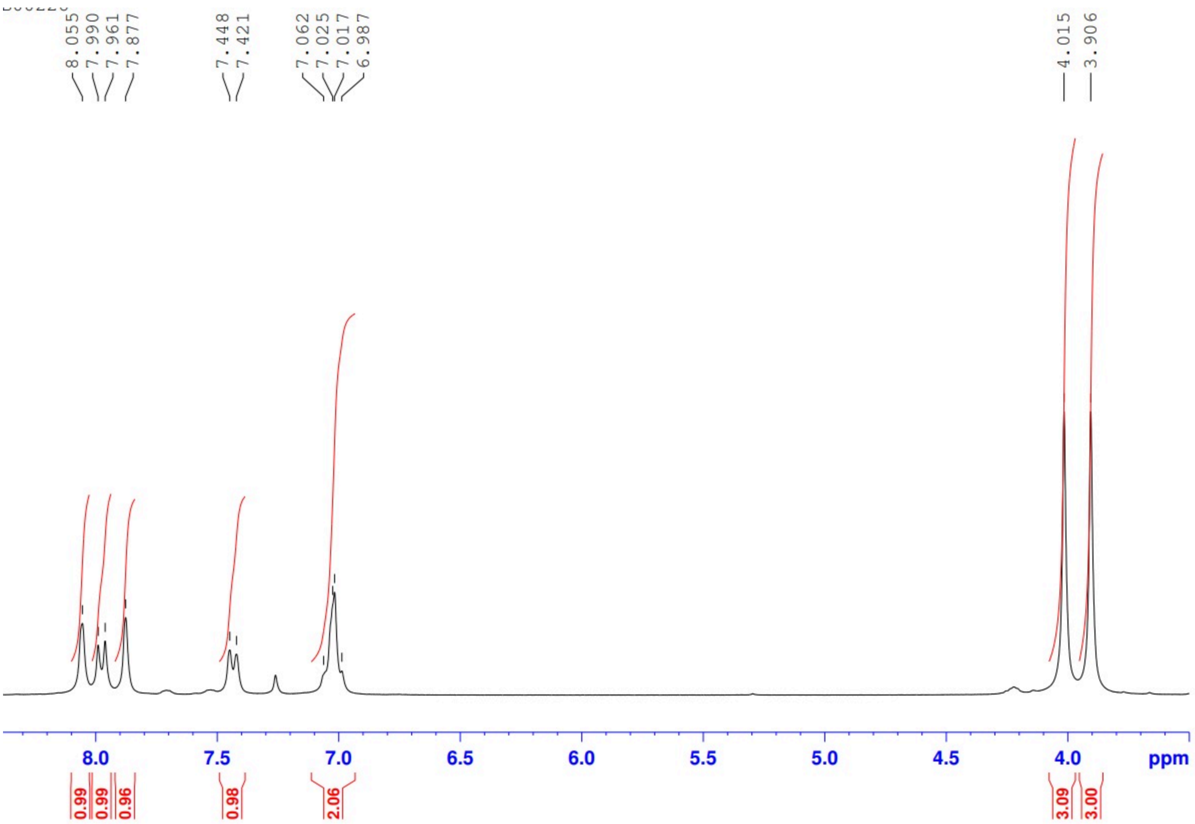

Figure S56. ^{1}**H-NMR (300 MHz, $CDCl_3$) spectrum of 27**

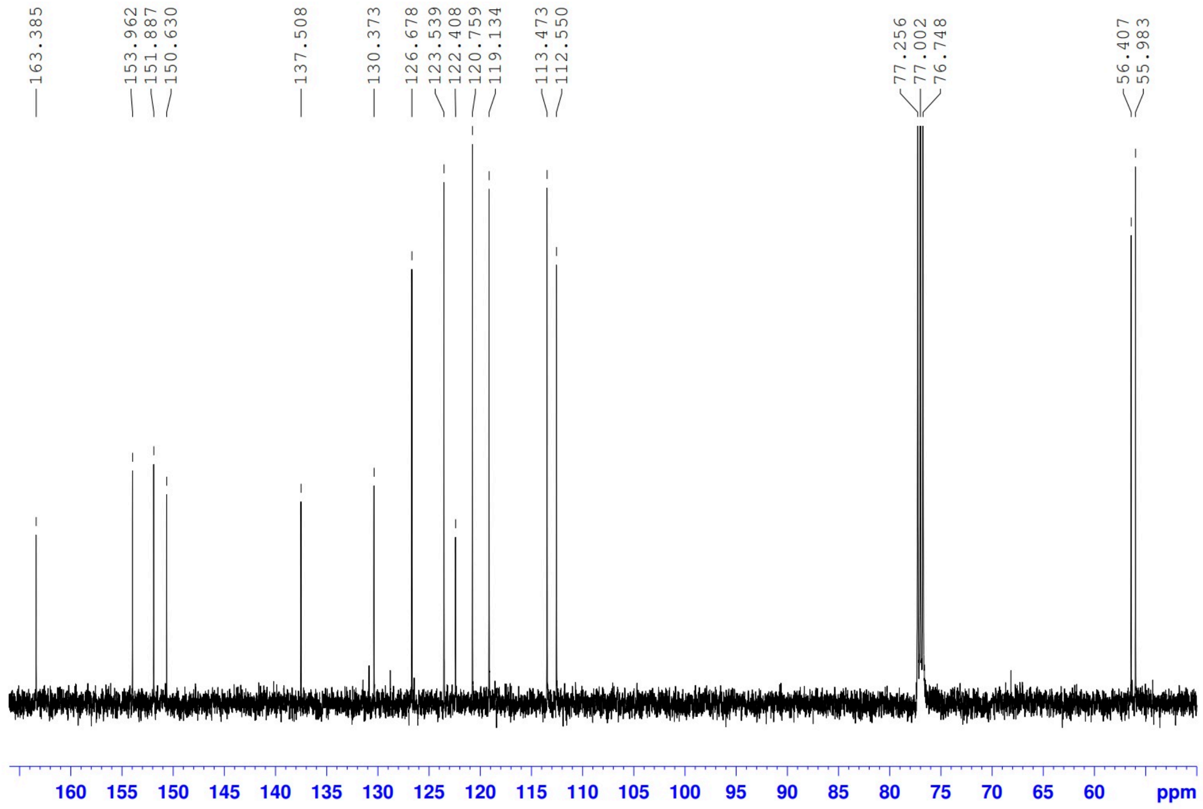

Figure S57. **^{13}C-NMR (125 MHz, $CDCl_3$) spectrum of 27**

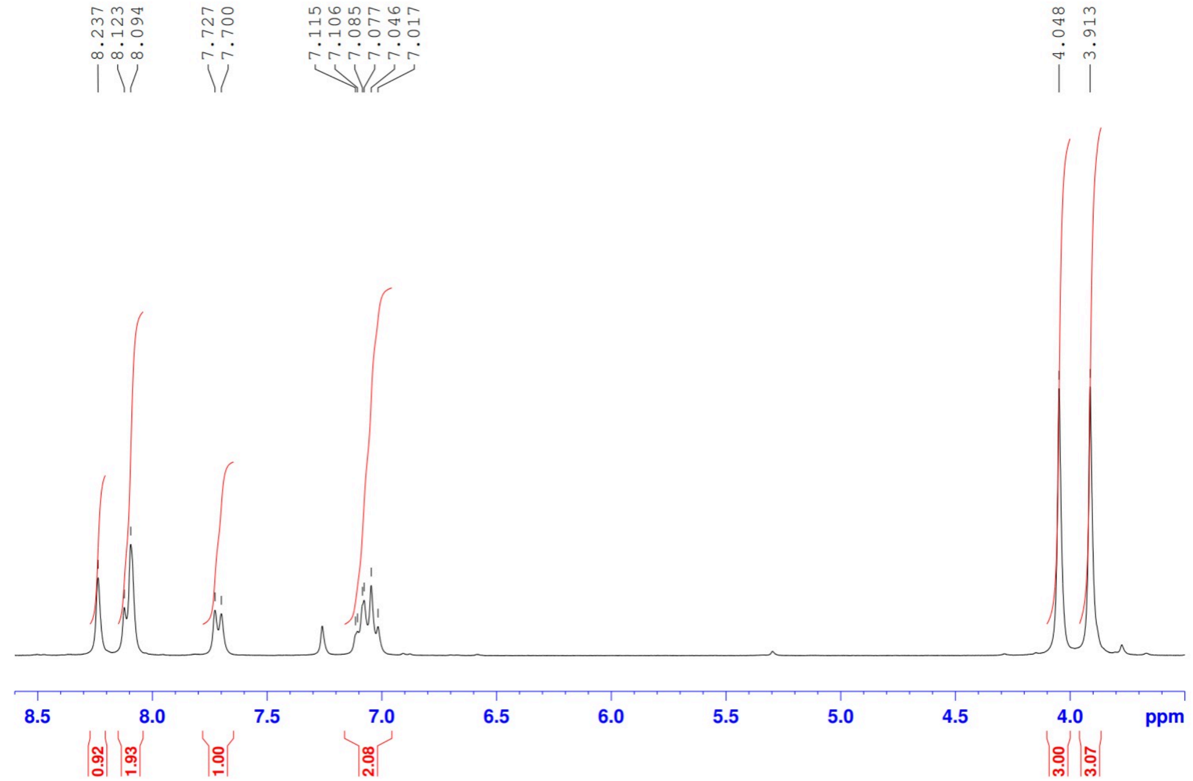

Figure S58. **^{1}H-NMR (300 MHz, $CDCl_3$) spectrum of 28**

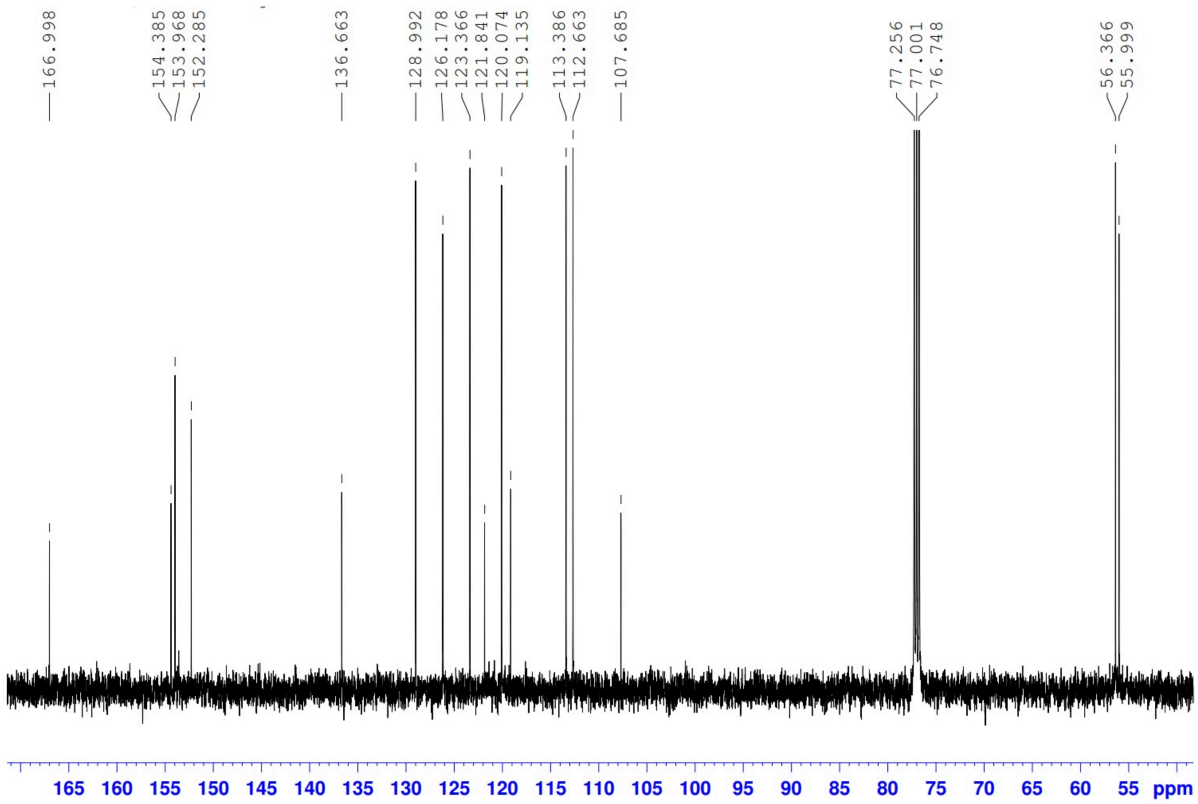

Figure S59. ^{13}C-NMR (125 MHz, $CDCl_3$) spectrum of 28

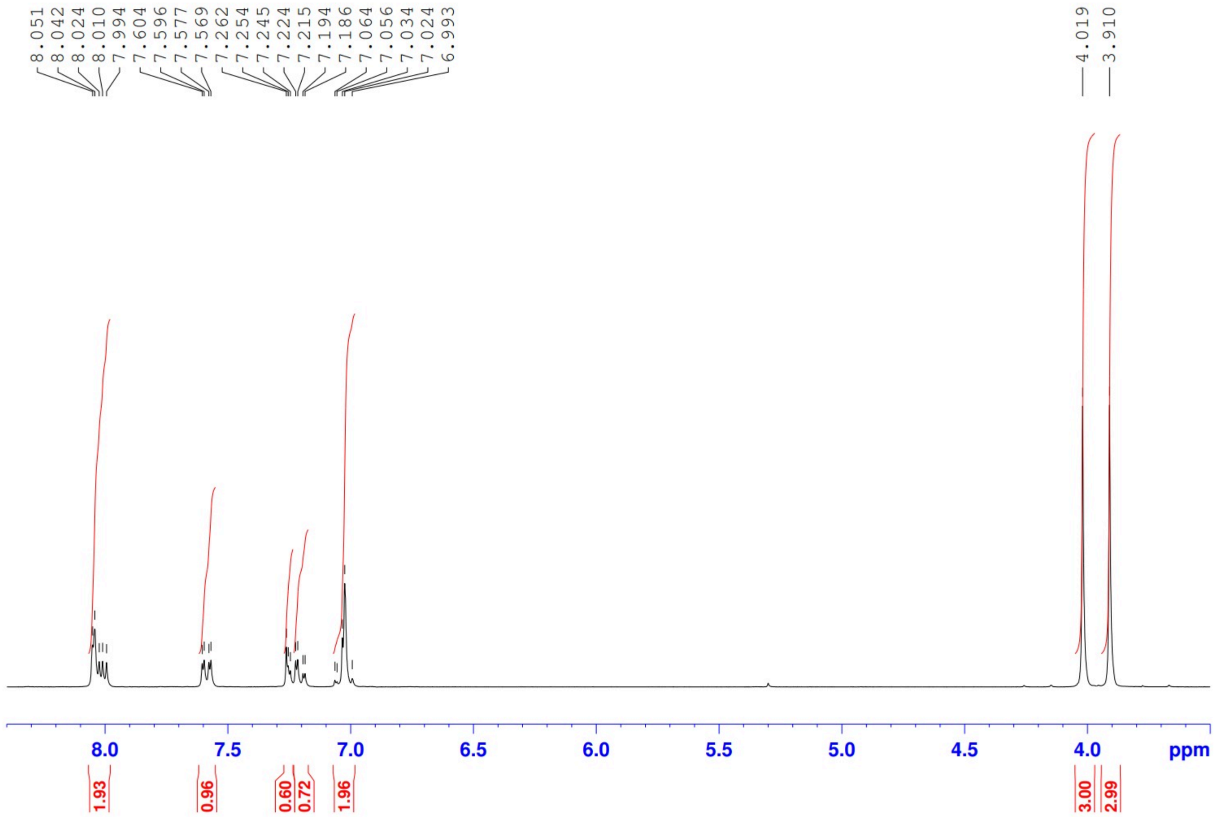

Figure S60. ^{1}H-NMR (300 MHz, $CDCl_3$) spectrum of 29

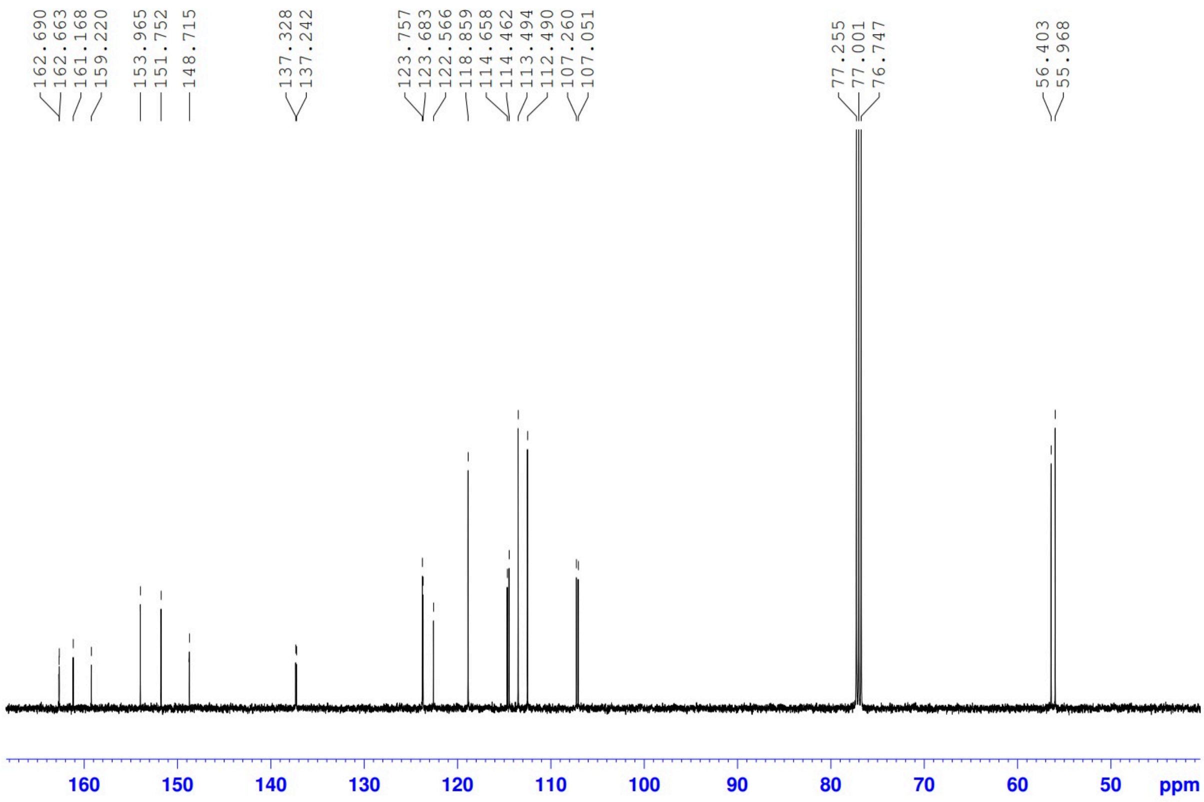

Figure S61. **^{13}C-NMR (125 MHz, $CDCl_3$) spectrum of 29**

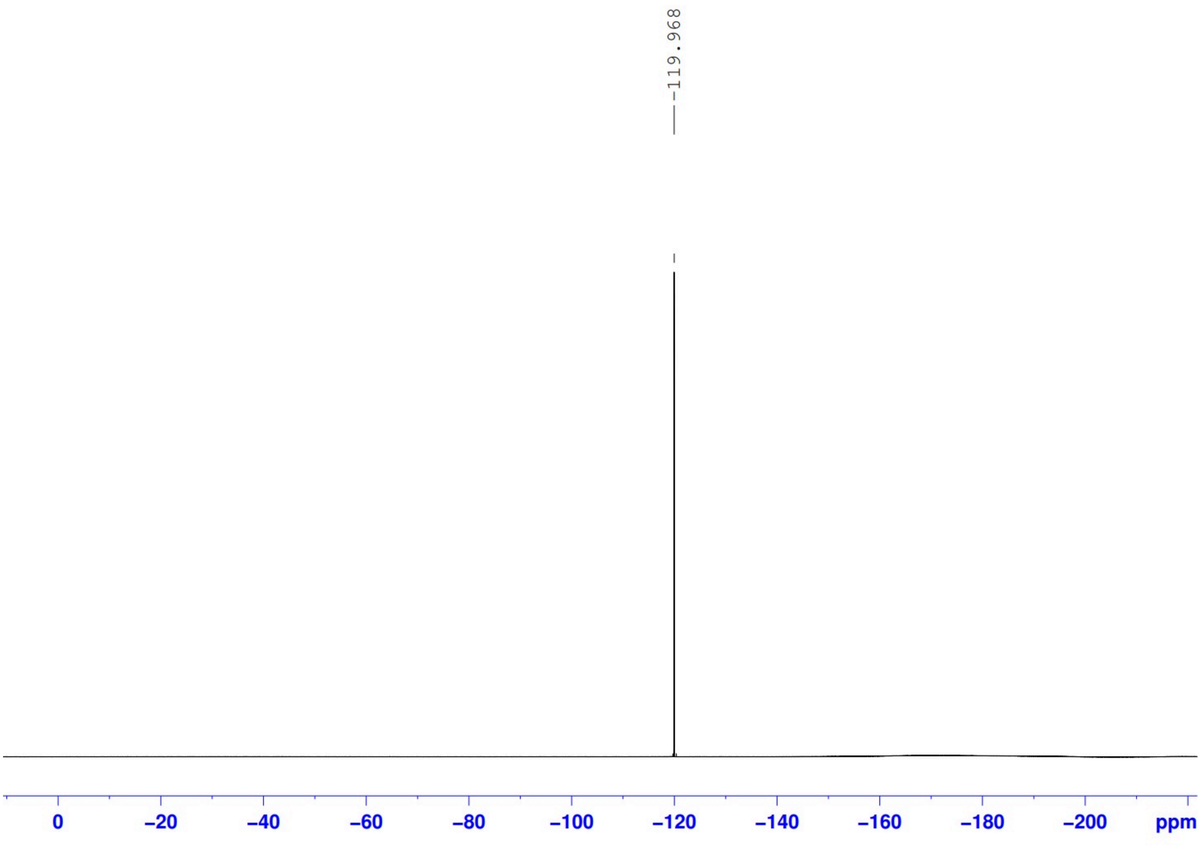

Figure S62. **^{19}F-NMR (376 MHz, $CDCl_3$) spectrum of 29**

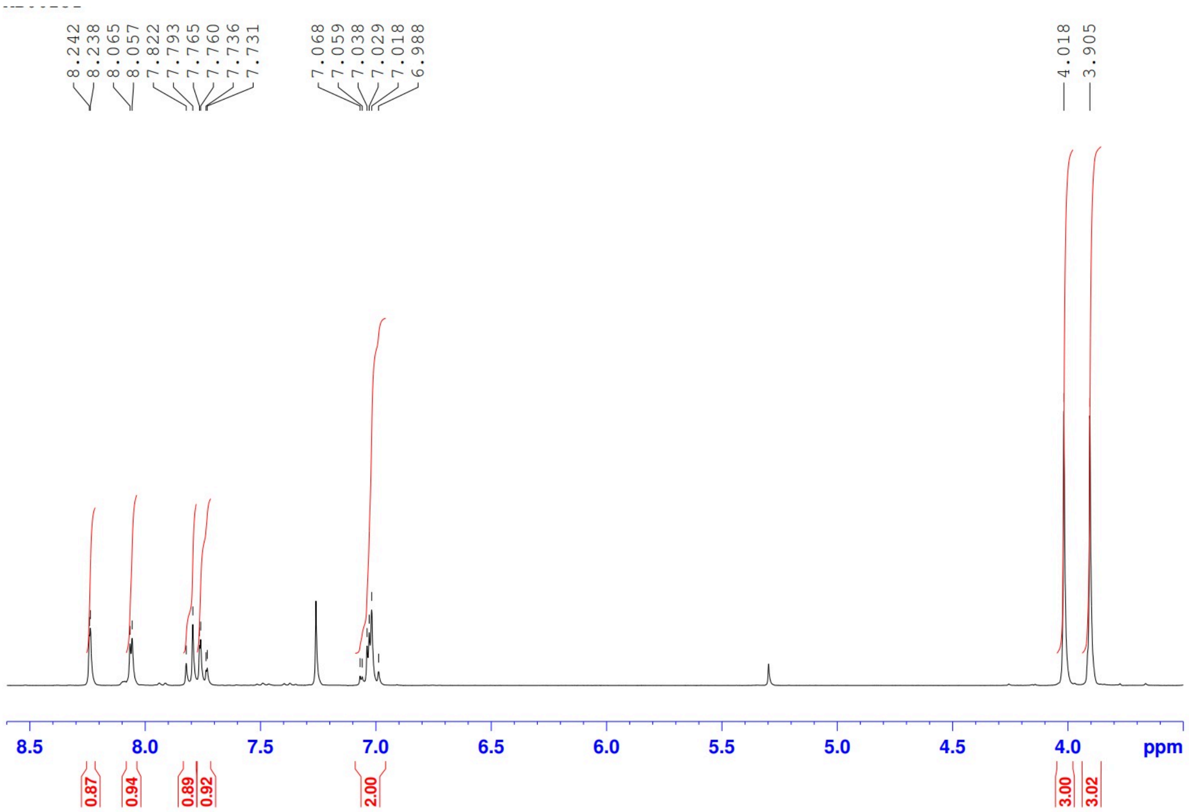

Figure S63. ^{1}H-NMR (300 MHz, $CDCl_3$) spectrum of 30

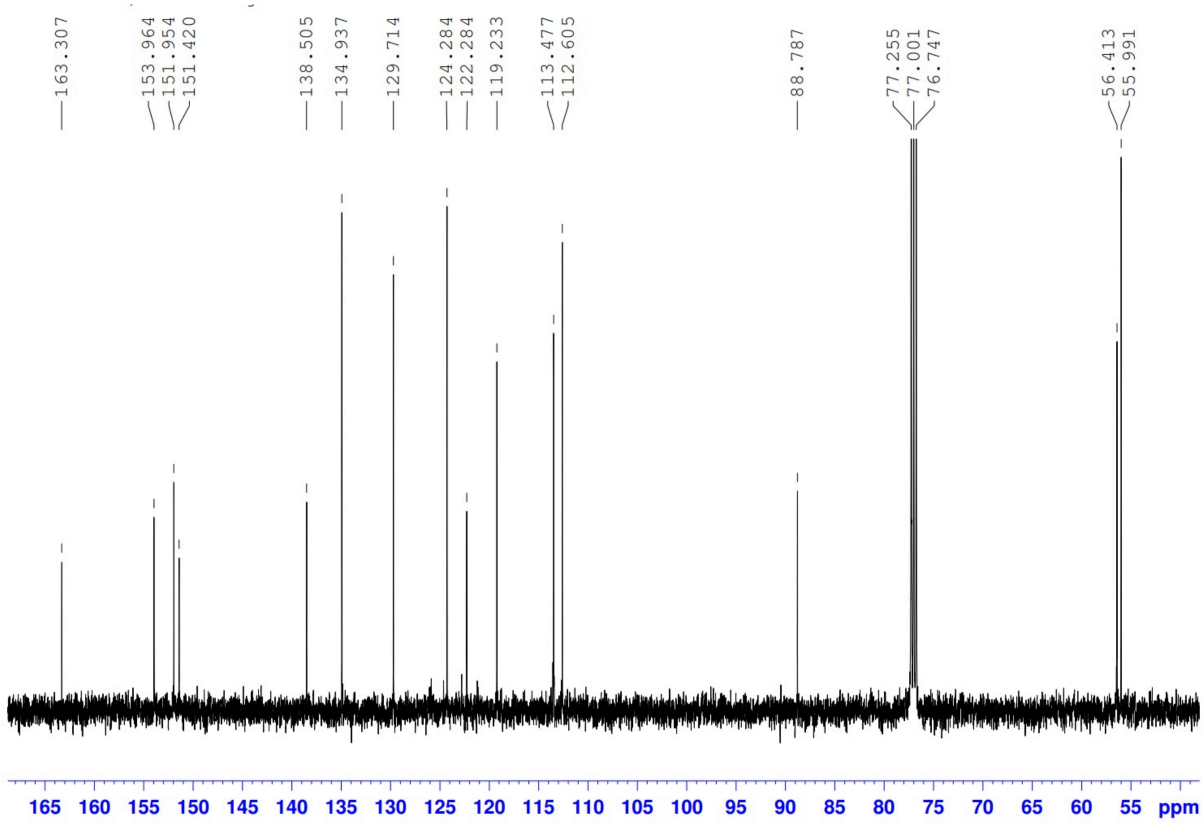

Figure S64. ^{13}C-NMR (125 MHz, $CDCl_3$) spectrum of 30

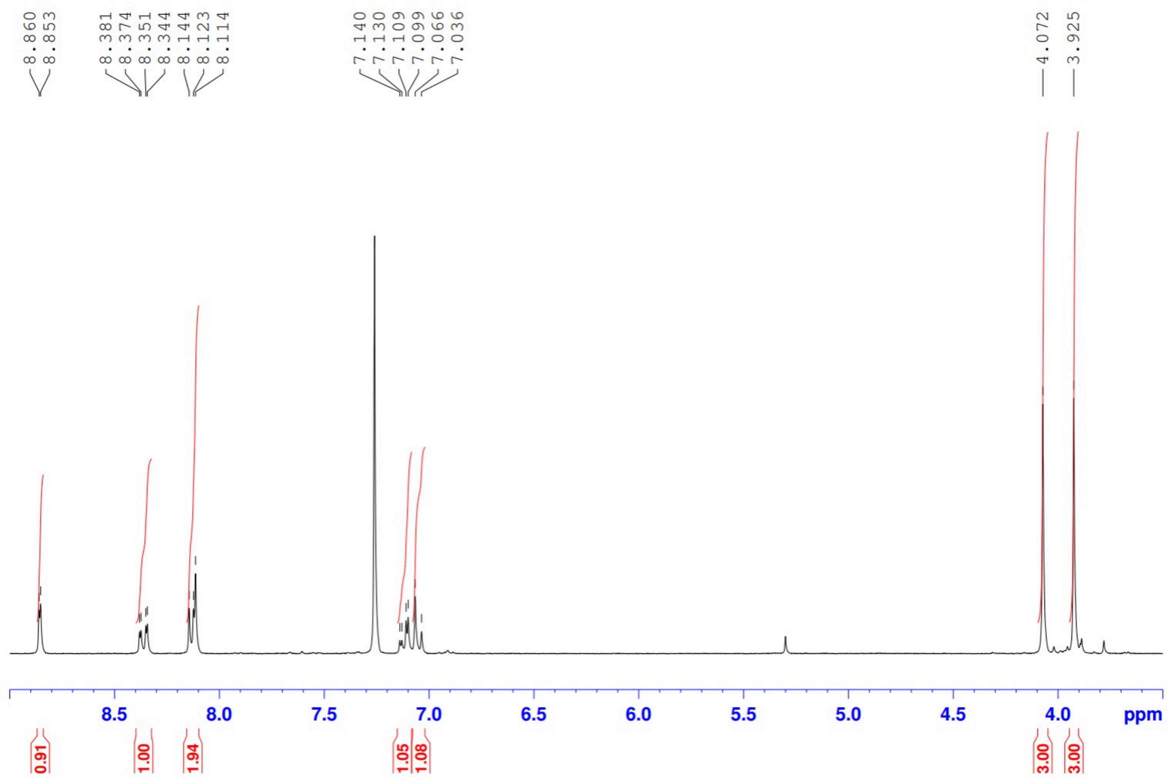

Figure S65. ^{1}H-NMR (300 MHz, CDCl$_3$) spectrum of 31

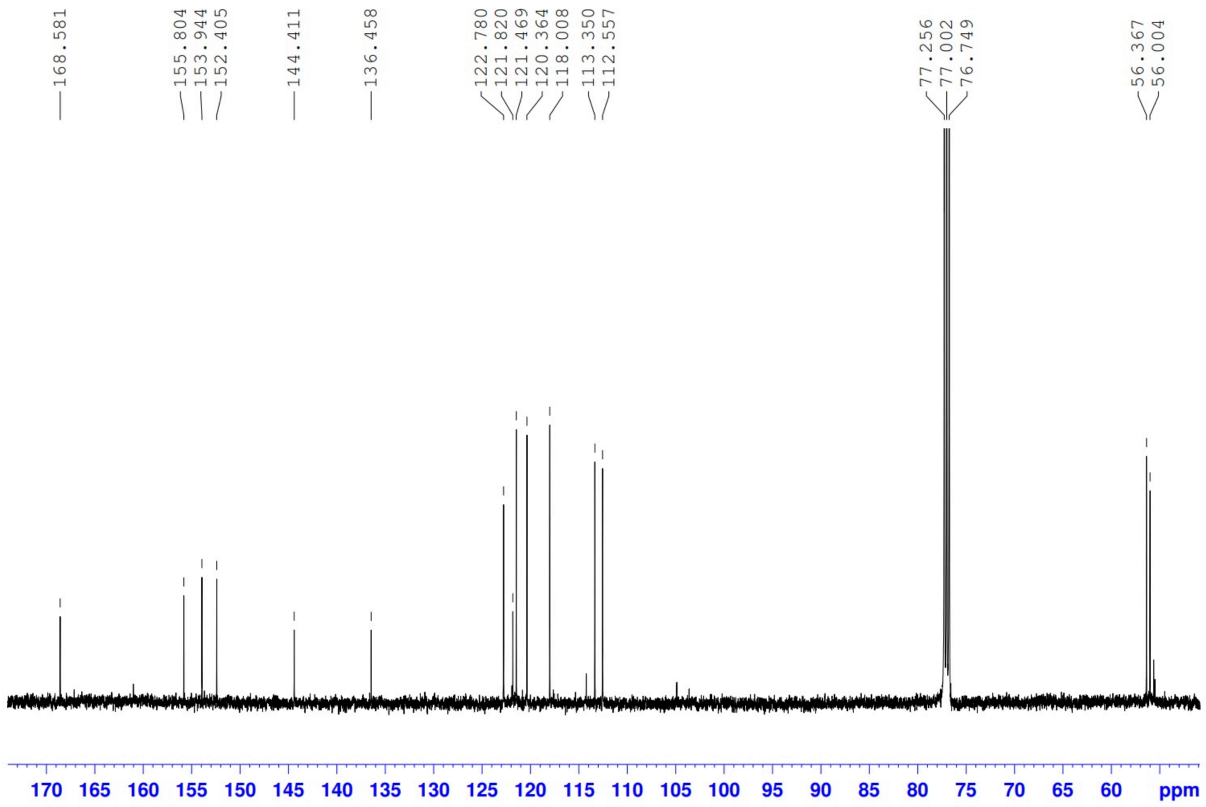

Figure S66. ^{13}C-NMR (125 MHz, CDCl$_3$) spectrum of 31

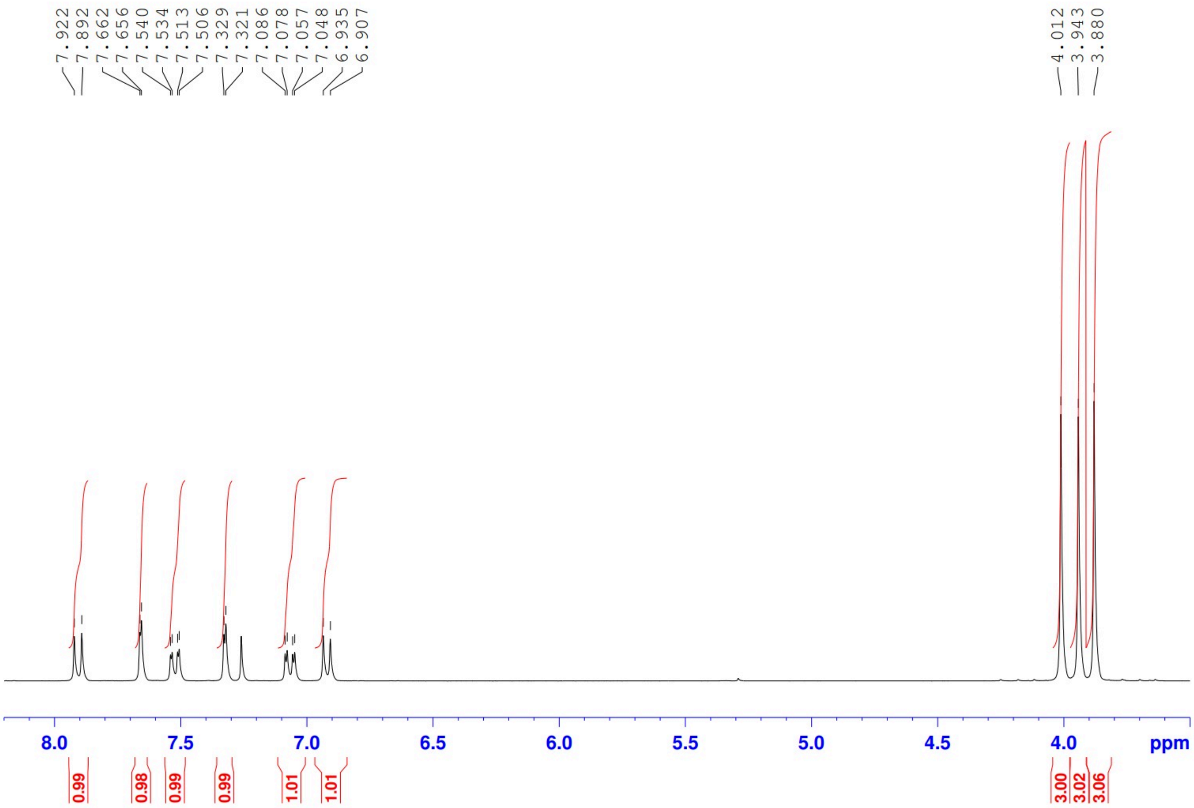

Figure S67. [1]**H-NMR (300 MHz, CDCl₃) spectrum of 32**

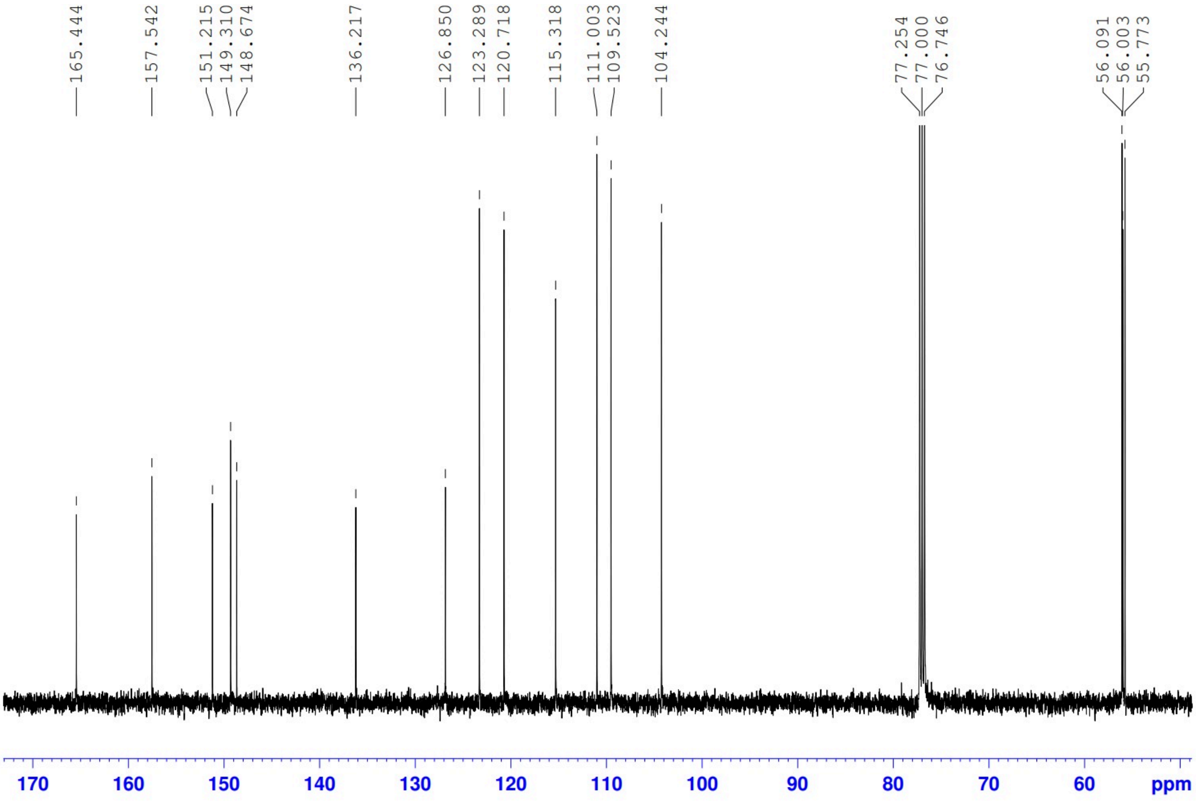

Figure S68. [13]**C-NMR (125 MHz, CDCl₃) spectrum of 32**

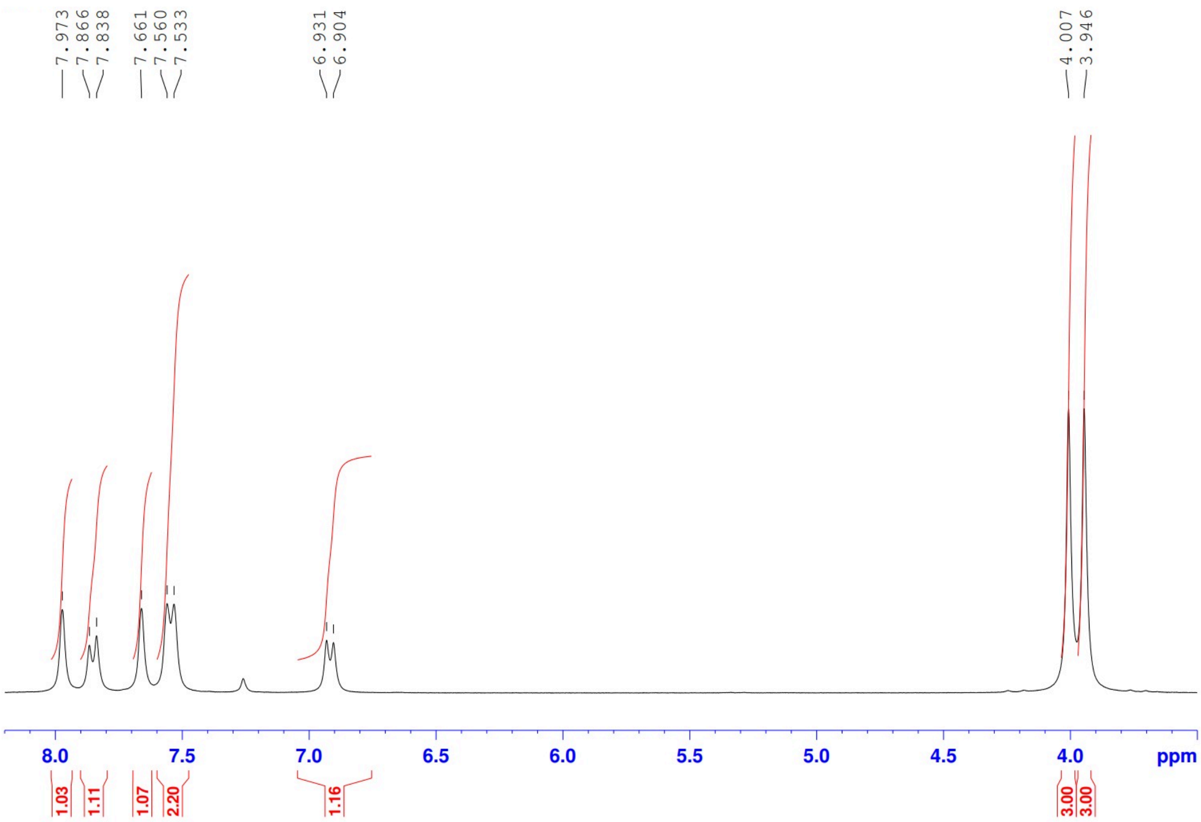

Figure S69. [1]**H-NMR (300 MHz, CDCl$_3$) spectrum of 33**

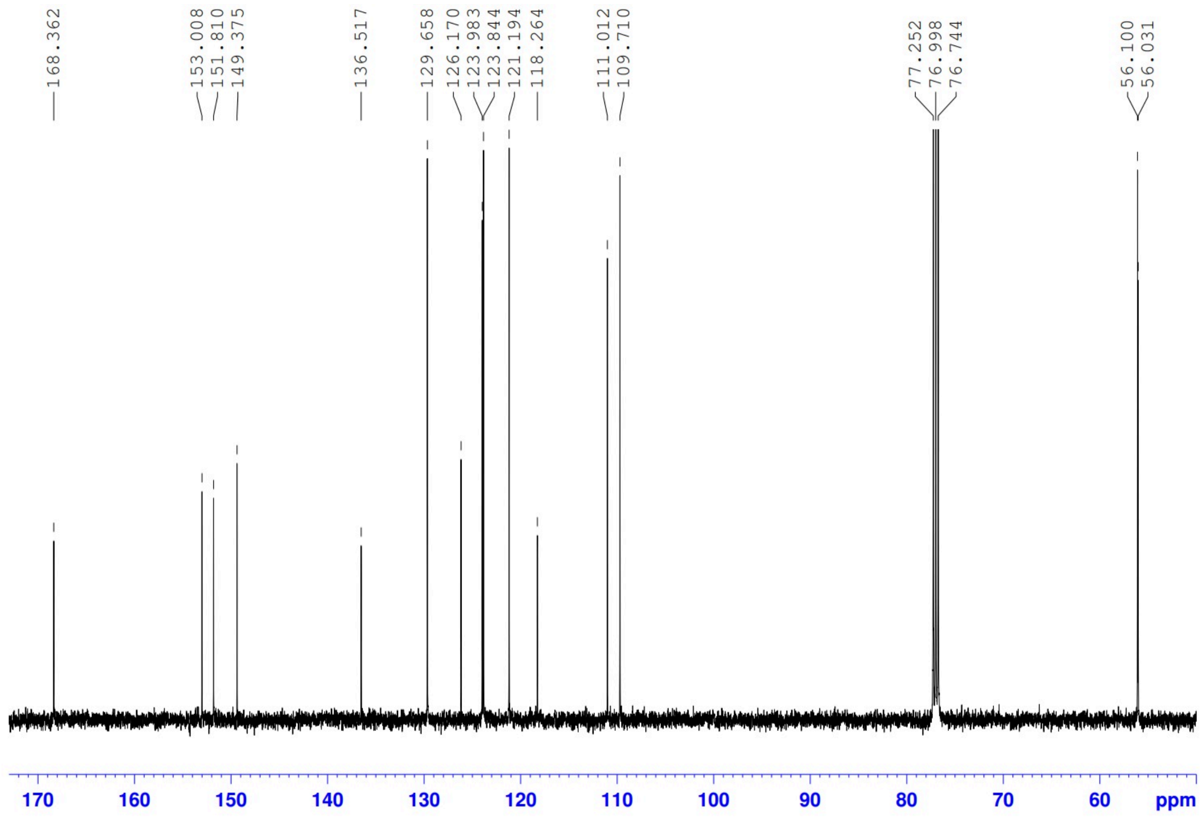

Figure S70. [13]**C-NMR (125 MHz, CDCl$_3$) spectrum of 33**

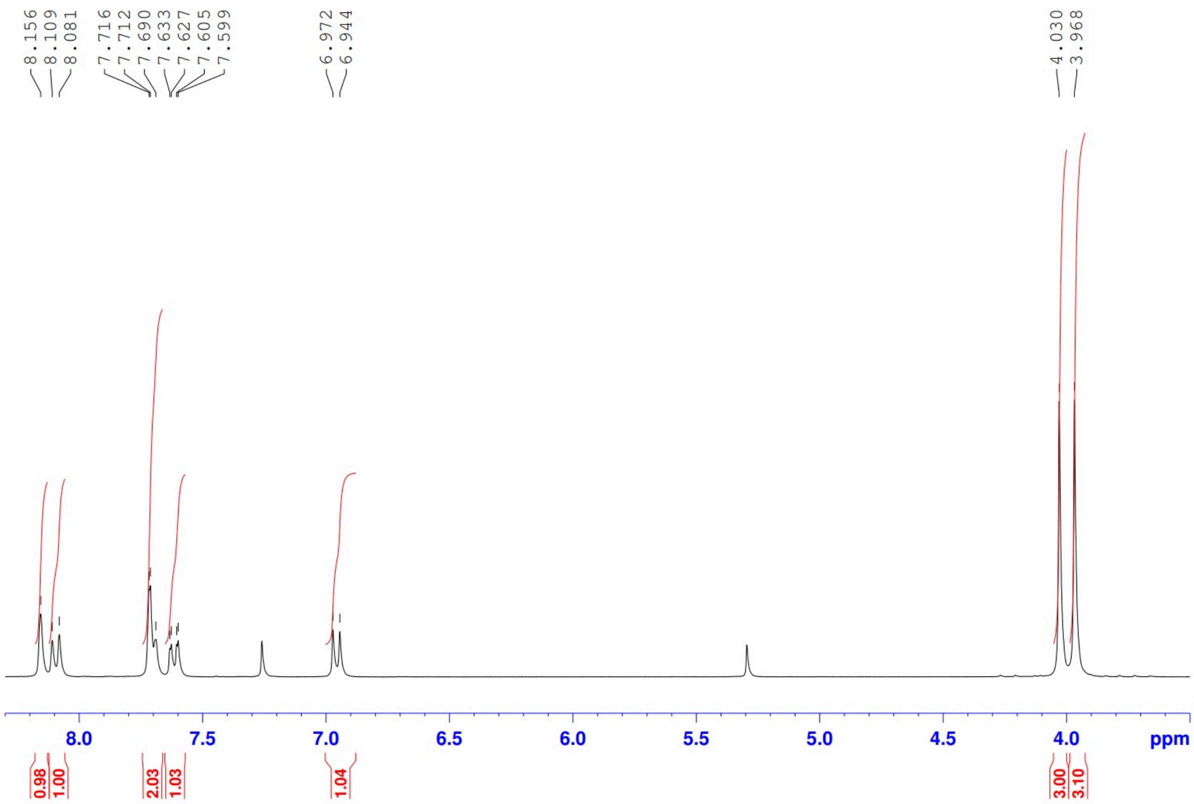

Figure S71. ^{1}H-NMR (300 MHz, $CDCl_3$) spectrum of 34

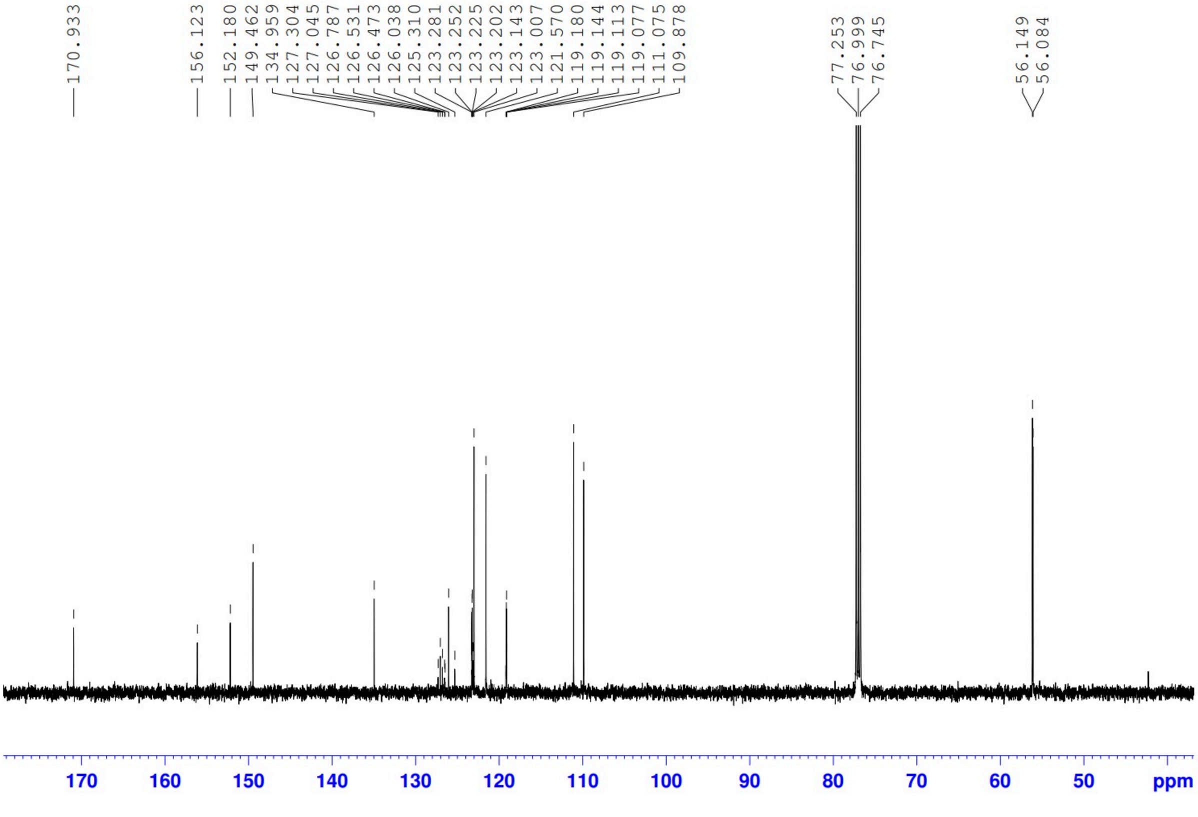

Figure S72. ^{13}C-NMR (125 MHz, $CDCl_3$) spectrum of 34

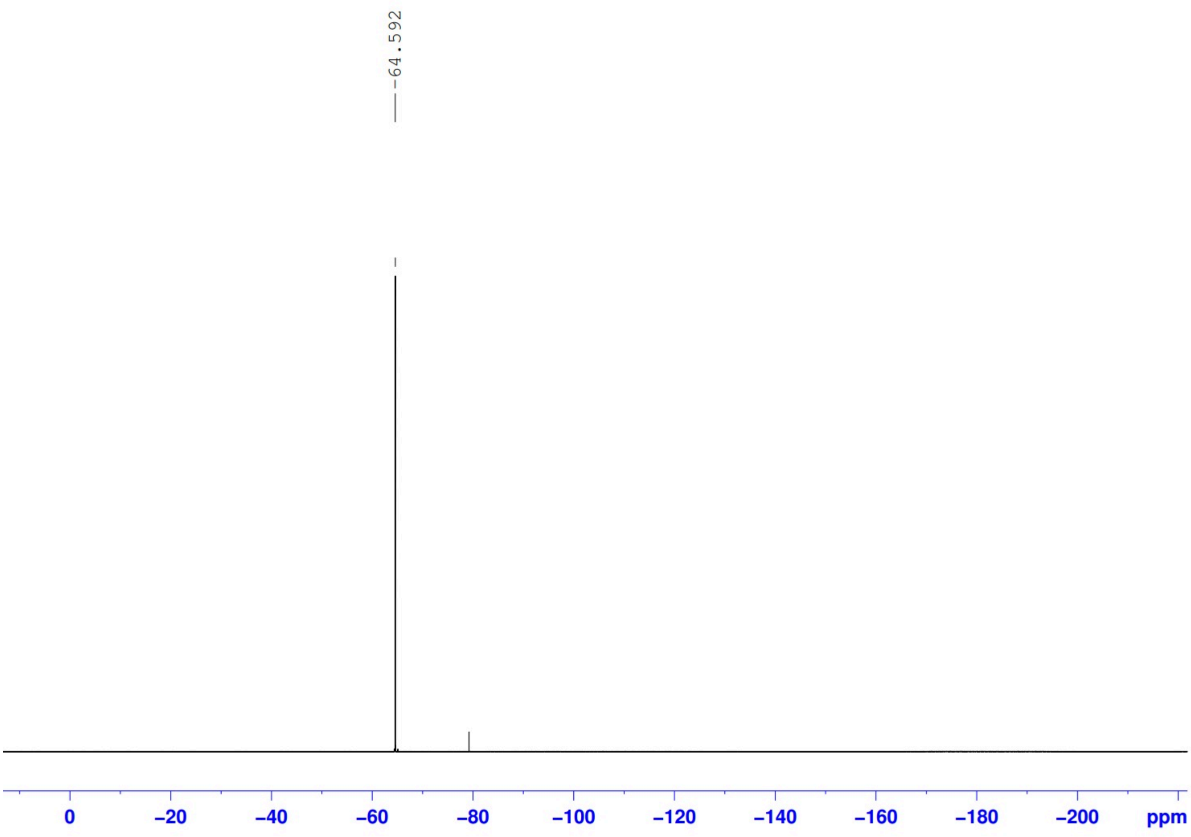

Figure S73. ^{19}F-NMR (376 MHz, $CDCl_3$) spectrum of 34

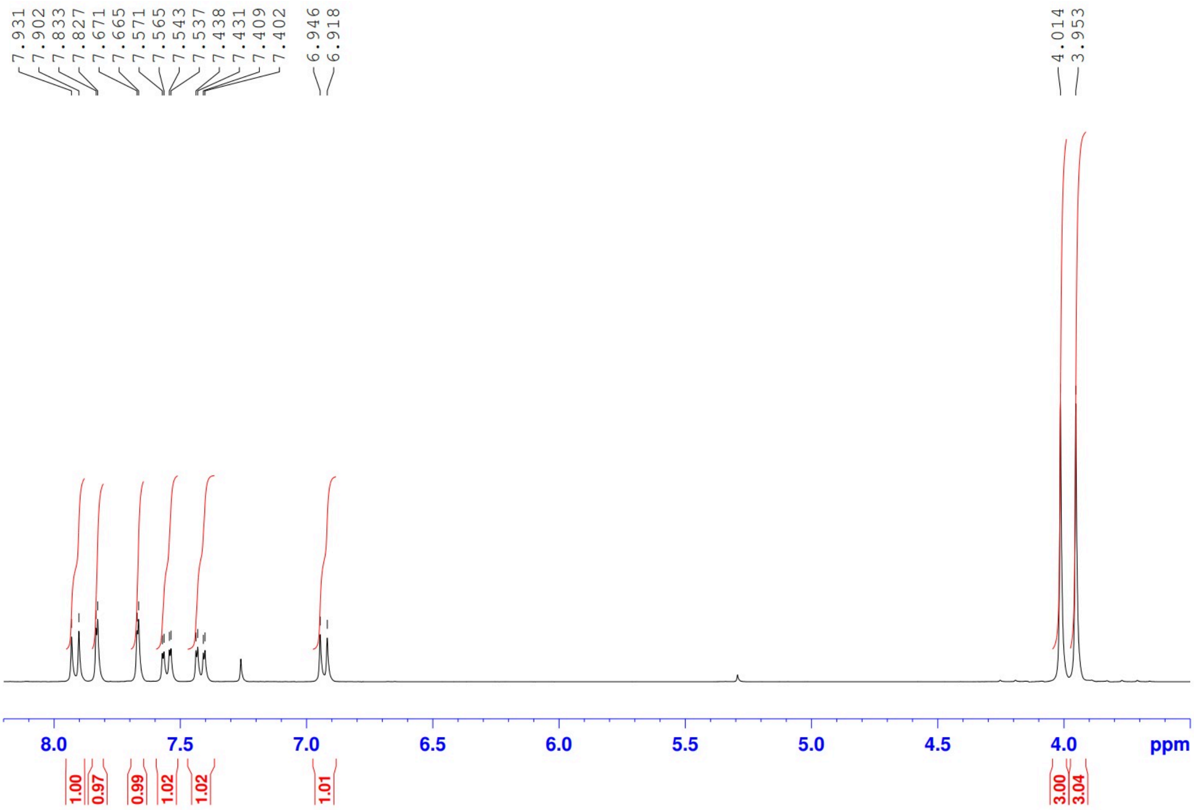

Figure S74. ^{1}H-NMR (300 MHz, $CDCl_3$) spectrum of 35

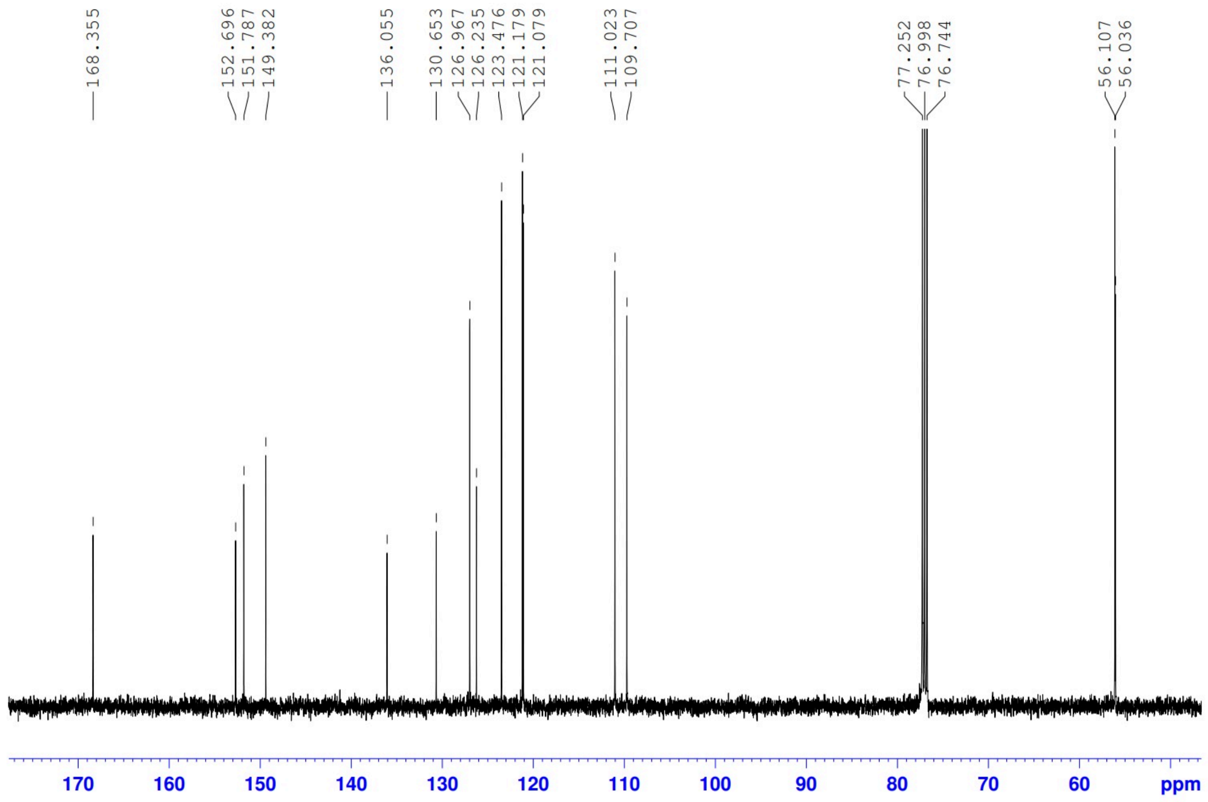

Figure S75. ^{13}C-NMR (125 MHz, CDCl$_3$) spectrum of 35

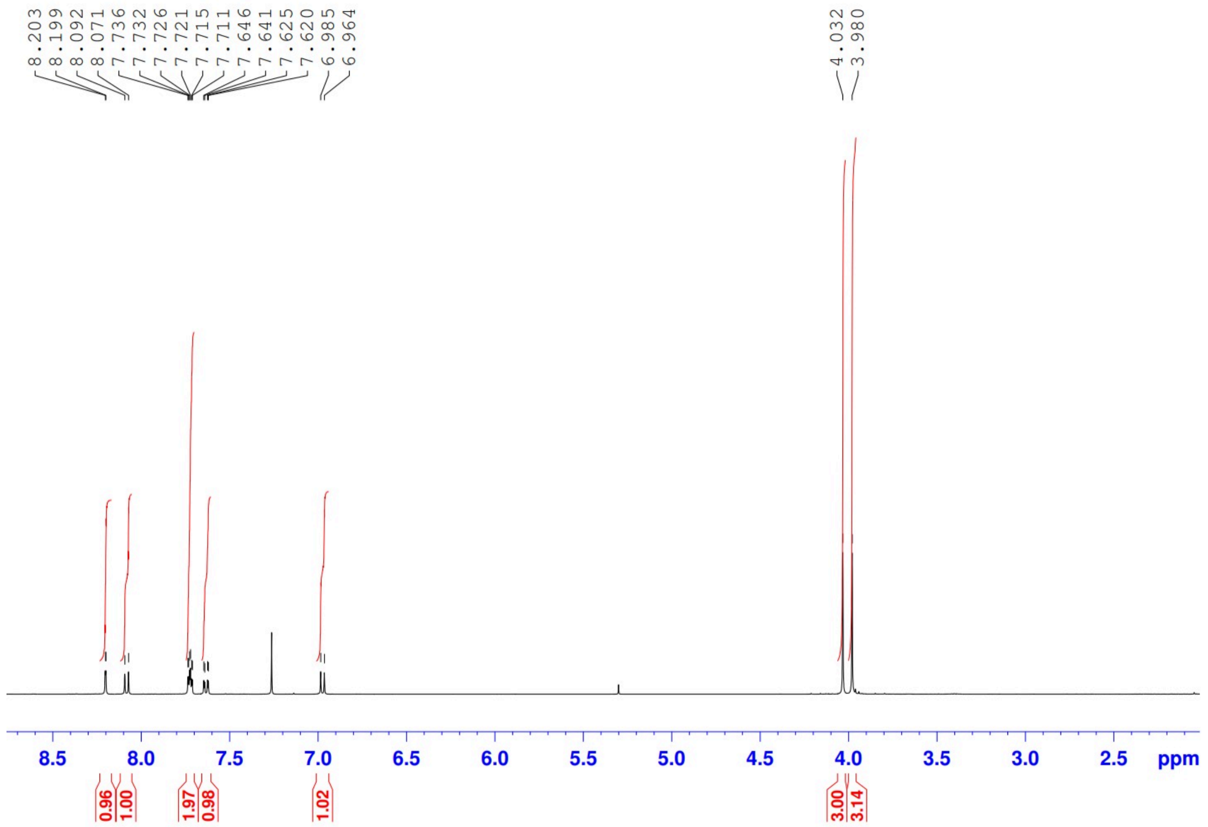

Figure S76. ^{1}H-NMR (400 MHz, CDCl$_3$) spectrum of 36

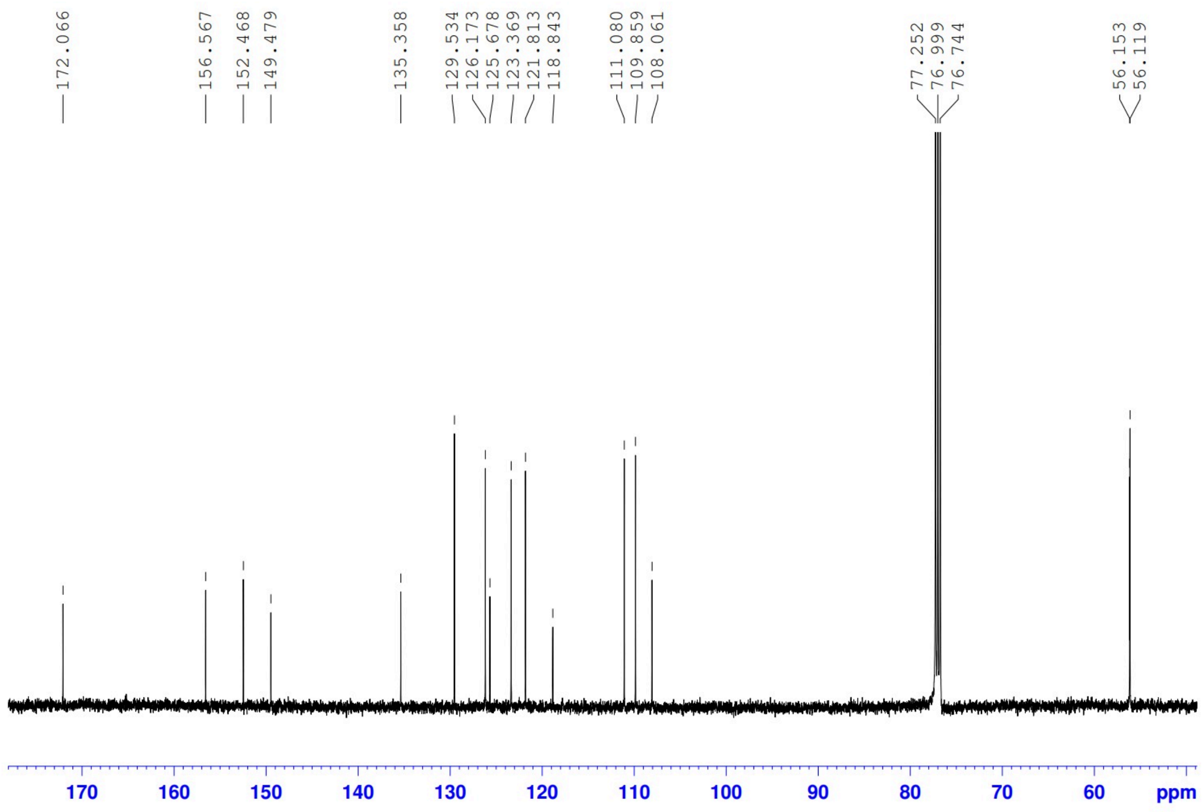

Figure S77. ^{13}C-NMR (125 MHz, CDCl₃) spectrum of 36

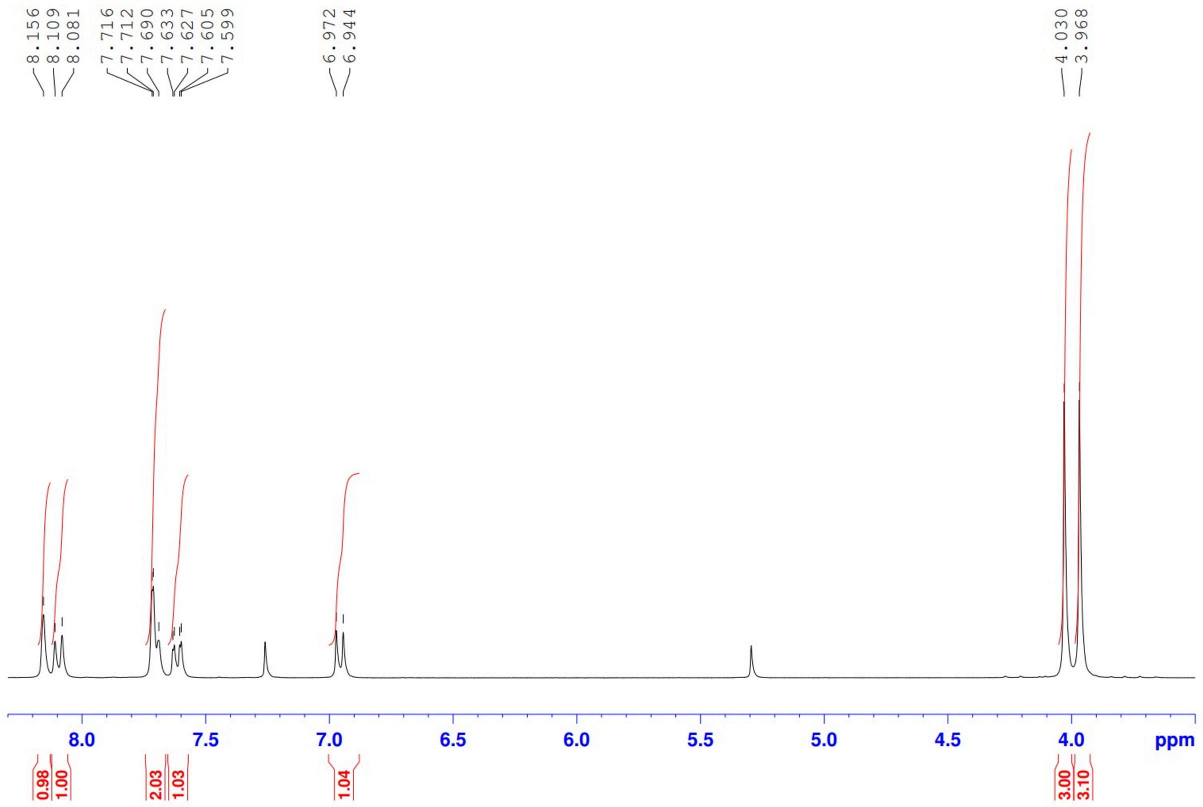

Figure S78. ^{1}H-NMR (300 MHz, CDCl₃) spectrum of 37

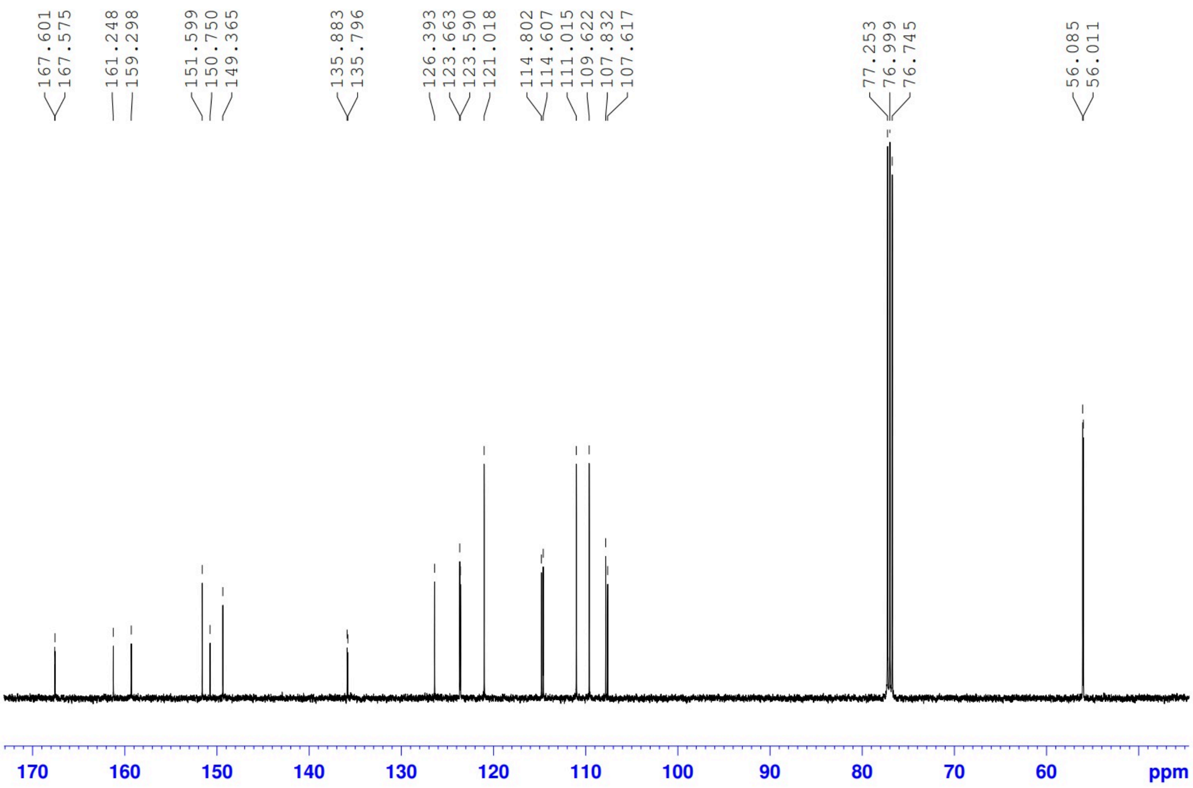

Figure S79. ^{13}**C-NMR (125 MHz, CDCl$_3$) spectrum of 37**

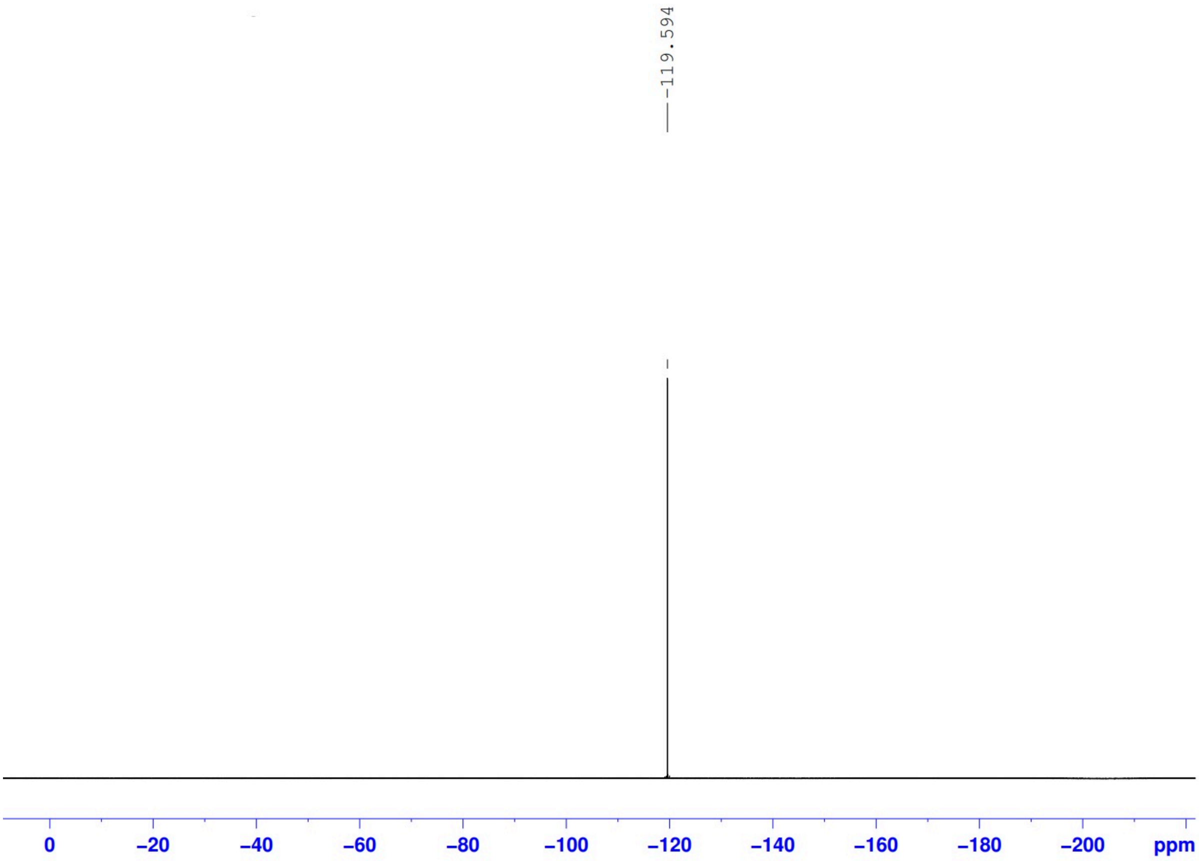

Figure S80. ^{19}**F-NMR (376 MHz, CDCl$_3$) spectrum of 37**

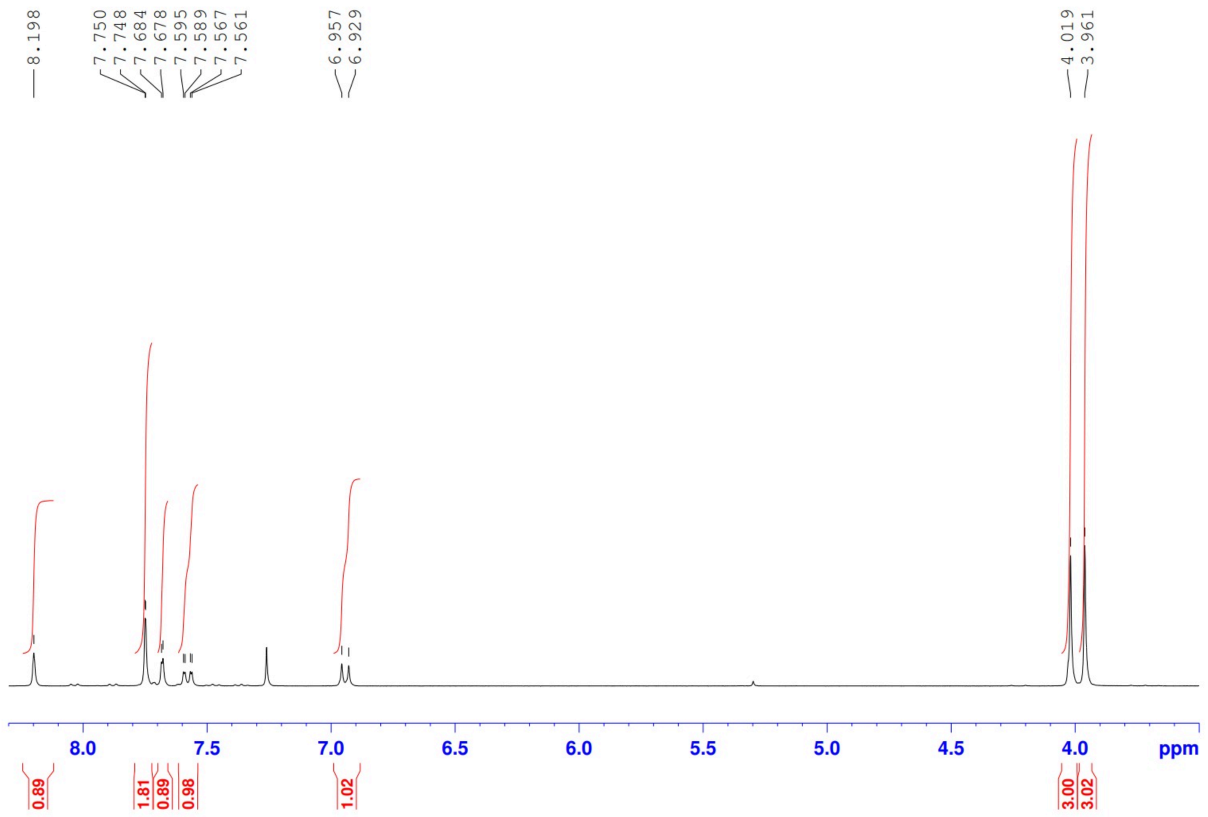

Figure S81. **^{1}H-NMR (300 MHz, $CDCl_3$) spectrum of 38**

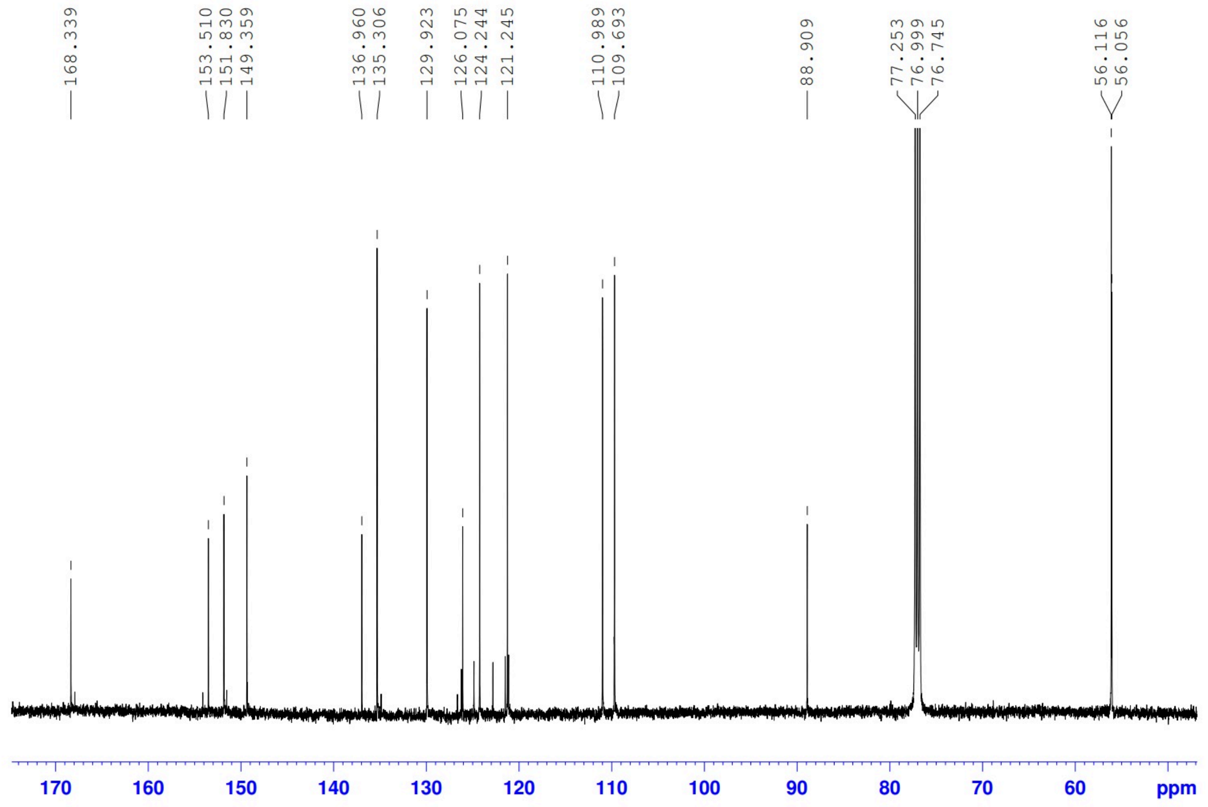

Figure S82. **^{13}C-NMR (125 MHz, $CDCl_3$) spectrum of 38**

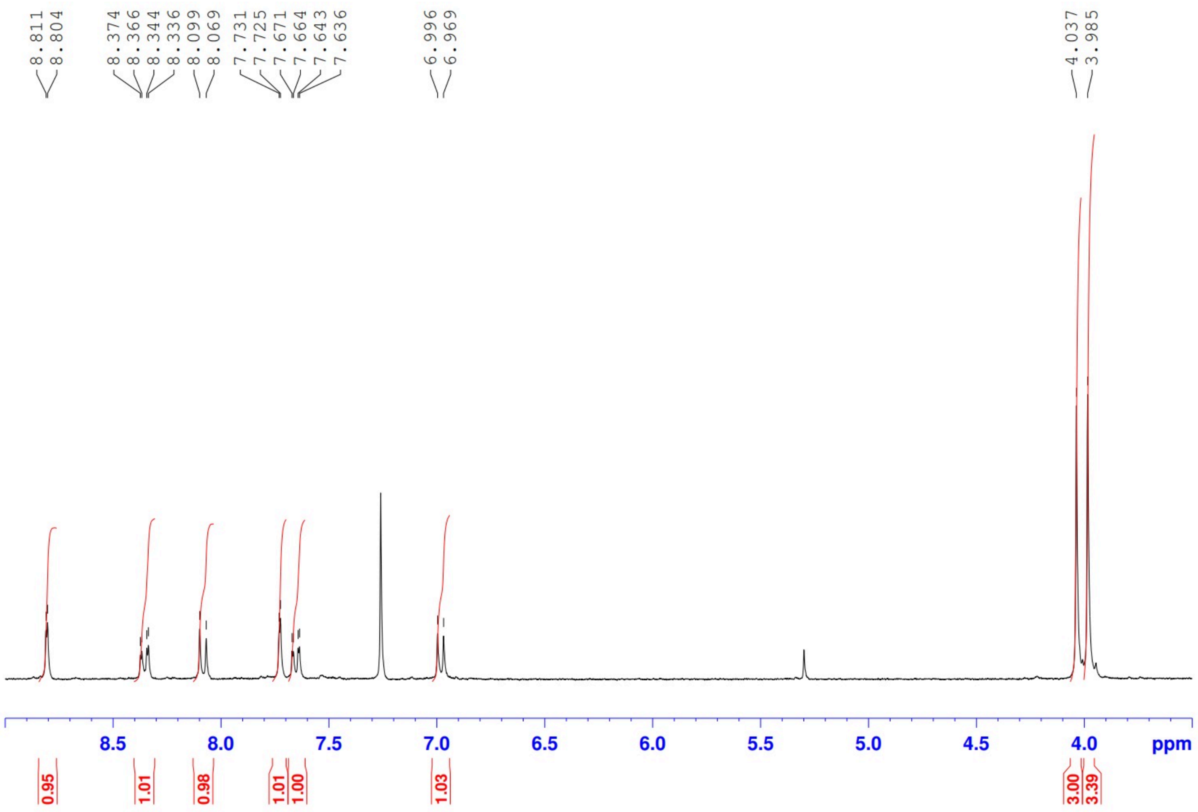

Figure S83. [1]**H-NMR (300 MHz, CDCl$_3$) spectrum of 39**

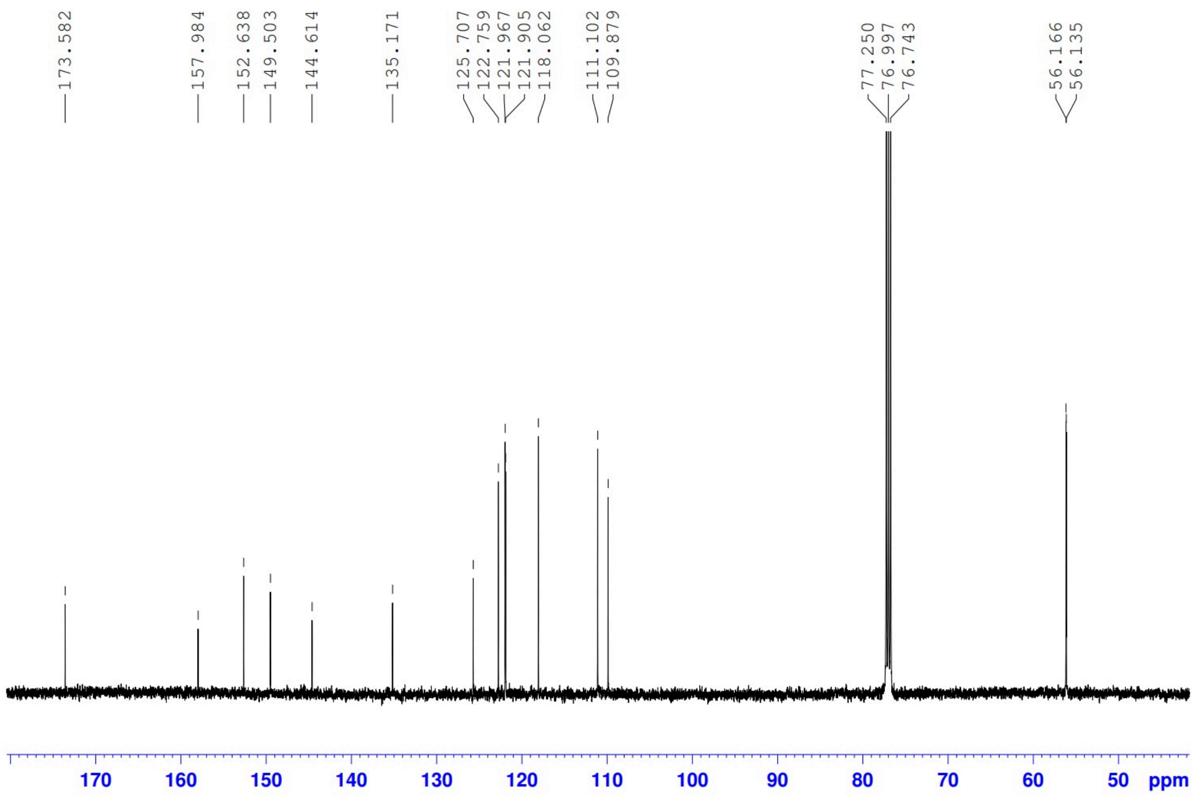

Figure S84. [13]**C-NMR (125 MHz, CDCl$_3$) spectrum of 39**

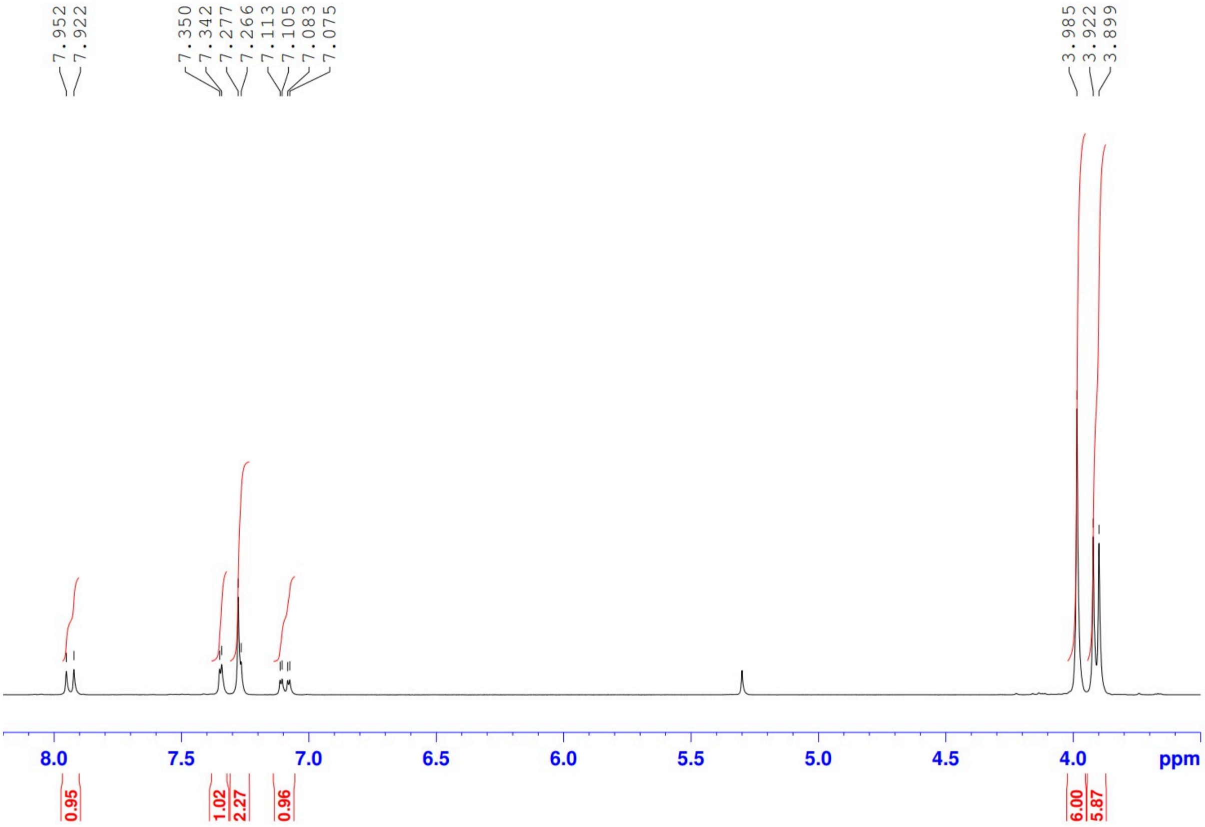

Figure S85. [1]**H-NMR (300 MHz, CDCl₃) spectrum of 40**

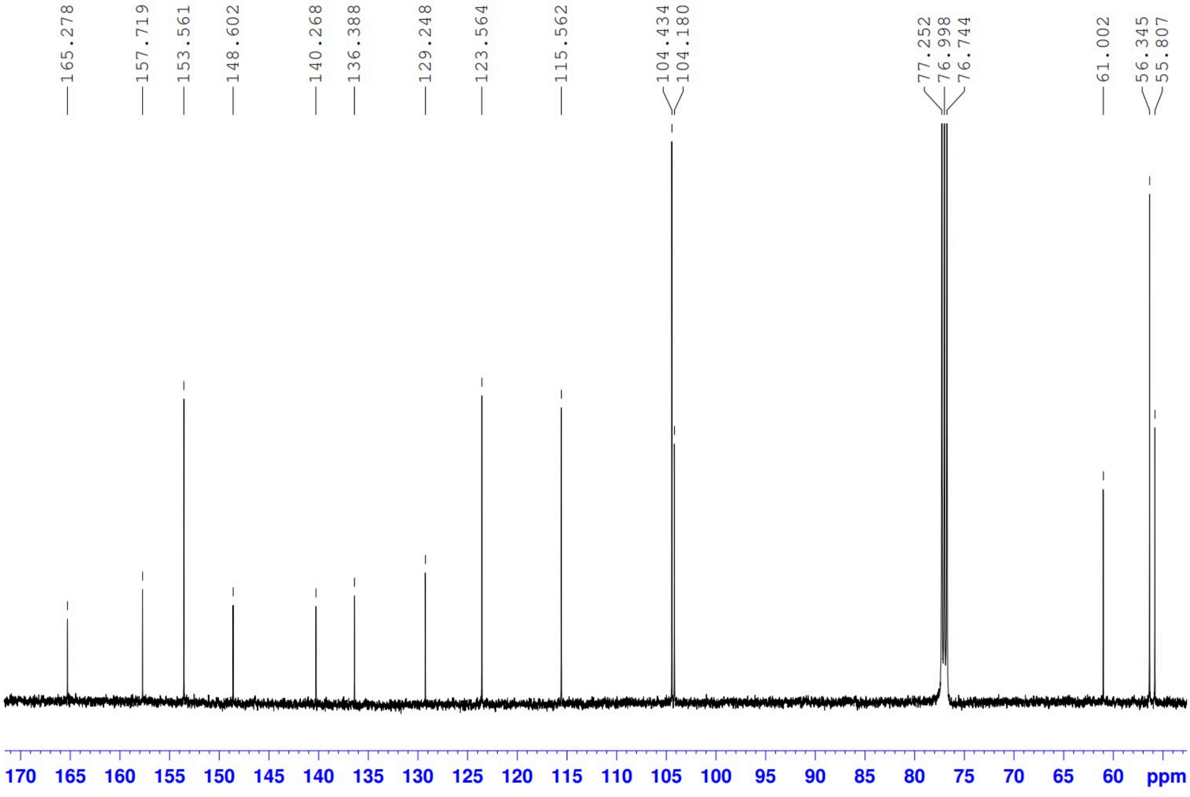

Figure S86. [13]**C-NMR (125 MHz, CDCl₃) spectrum of 40**

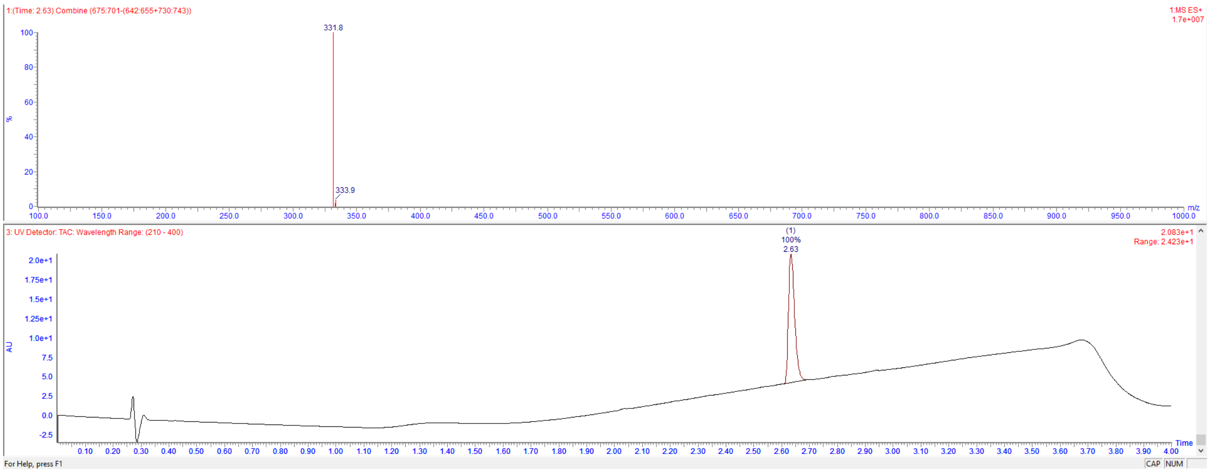

Figure S87. LCMS of 40

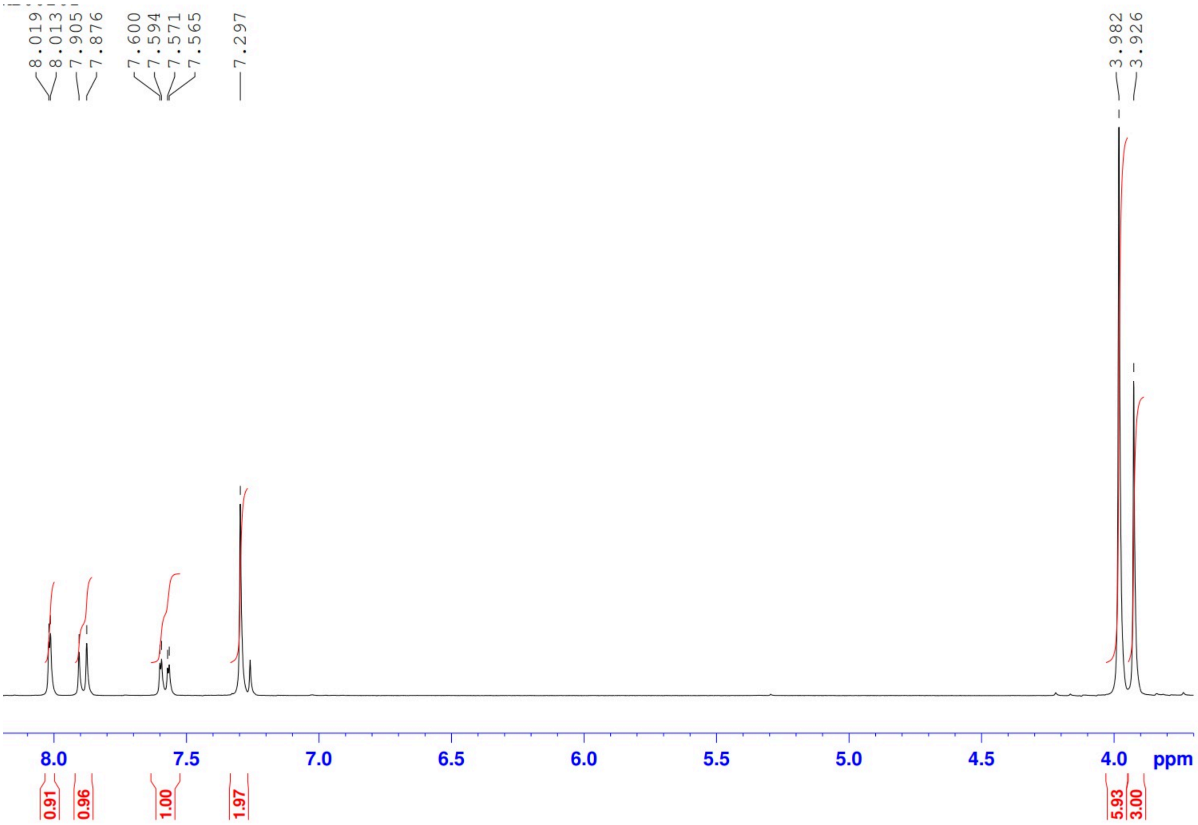

Figure S88. ^{1}H-NMR (300 MHz, $CDCl_3$) spectrum of 41

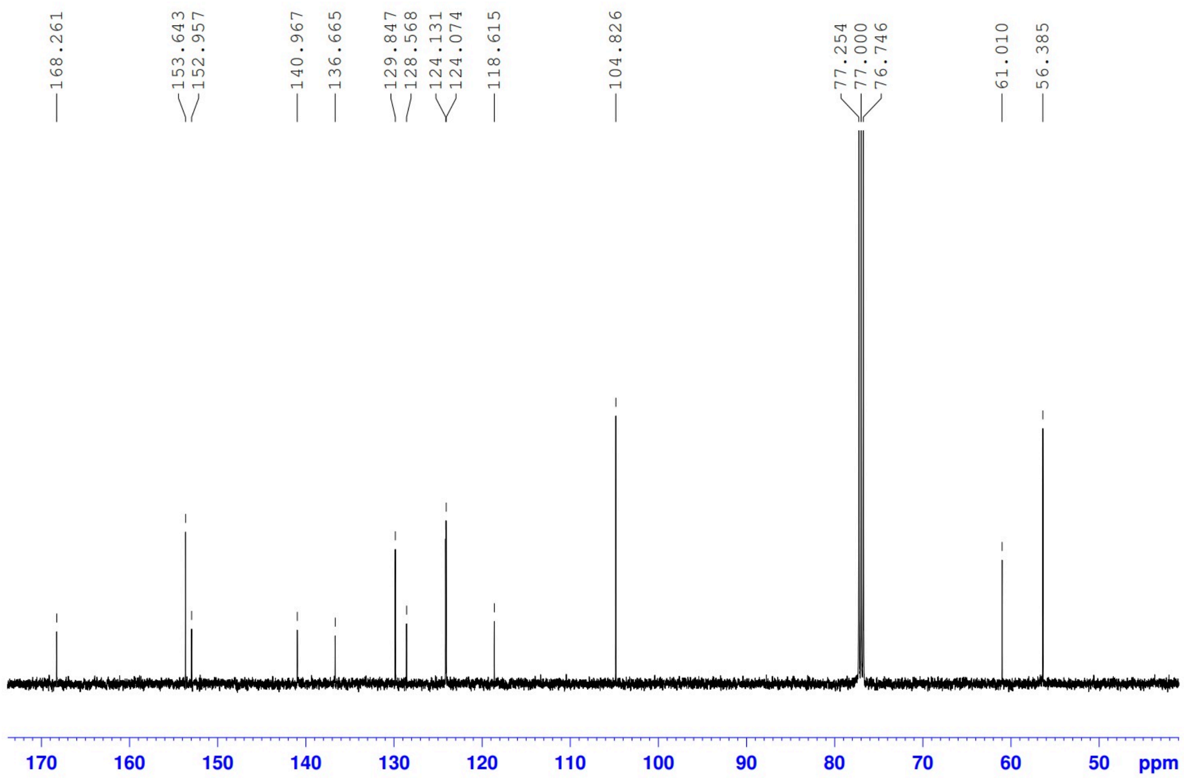

Figure S89. ^{13}**C-NMR (125 MHz, CDCl$_3$) spectrum of 41**

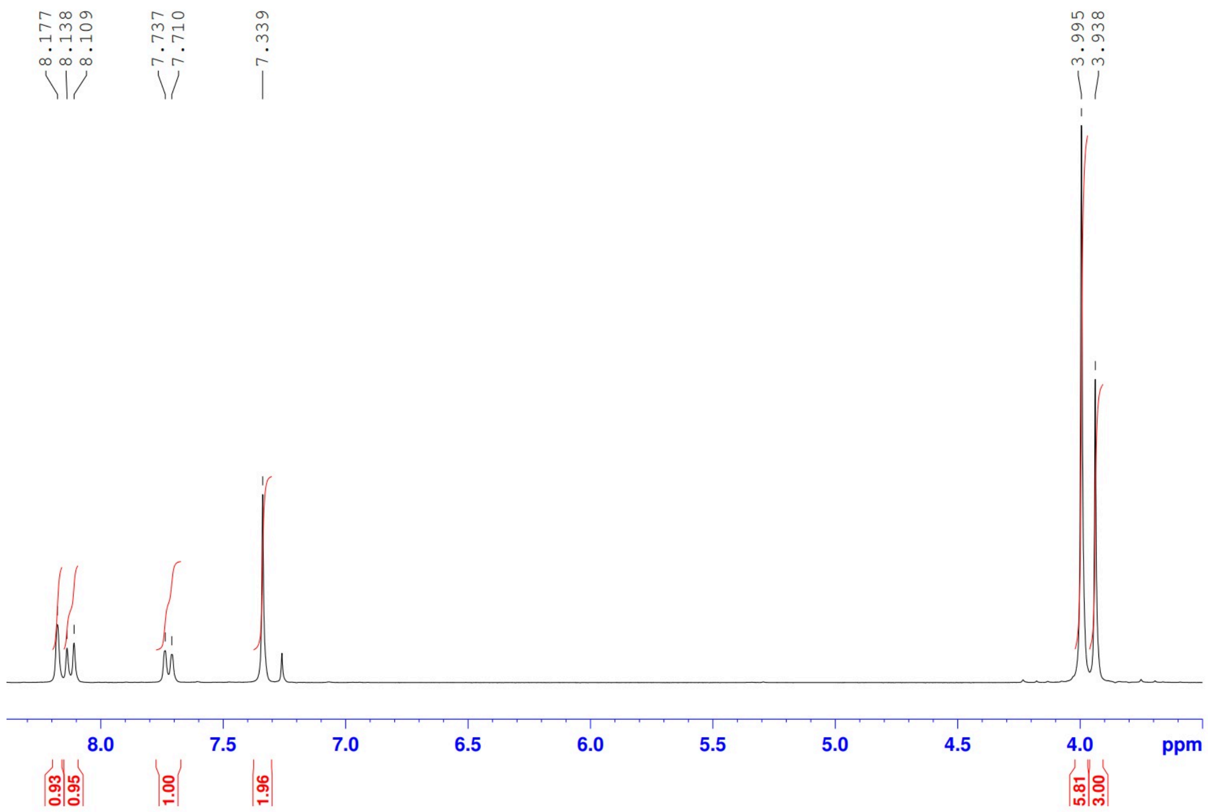

Figure S90. ^{1}**H-NMR (300 MHz, CDCl$_3$) spectrum of 42**

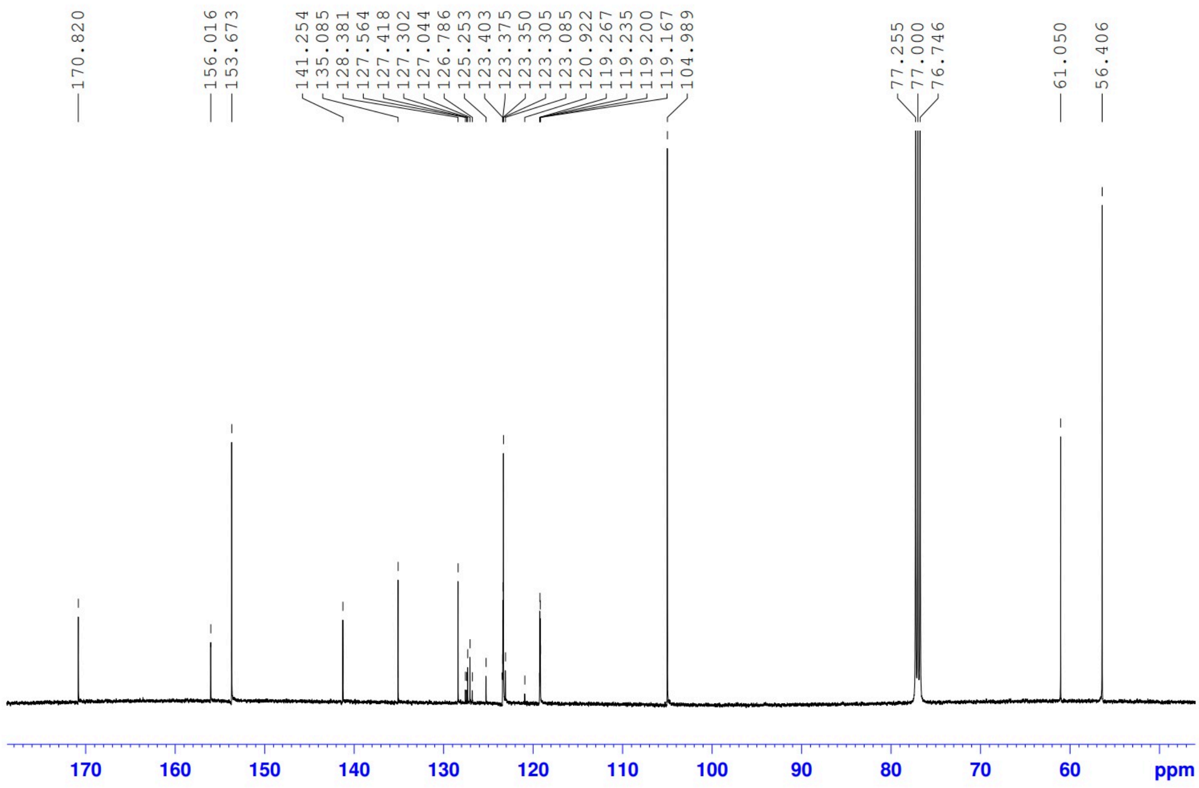

Figure S91. ^{13}C-NMR (125 MHz, $CDCl_3$) spectrum of **42**

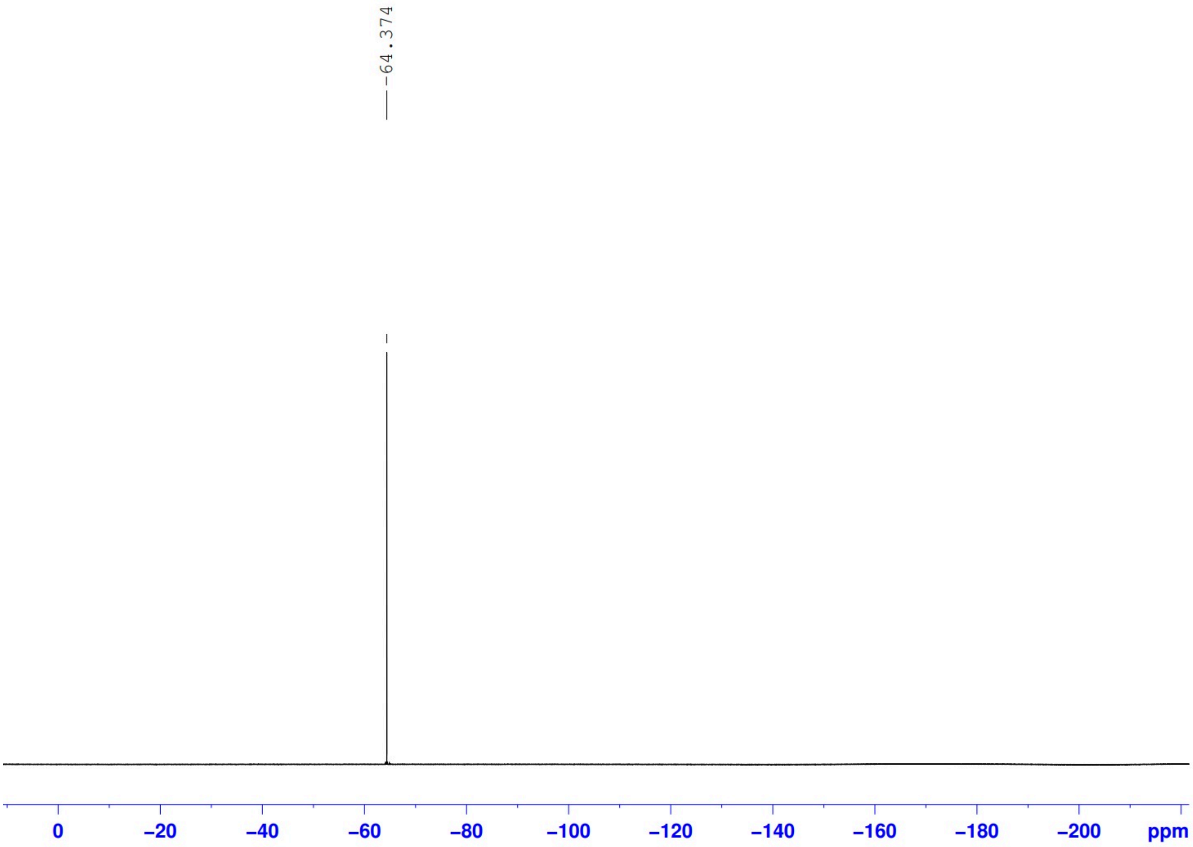

Figure S92. ^{19}F-NMR (376 MHz, $CDCl_3$) spectrum of **42**

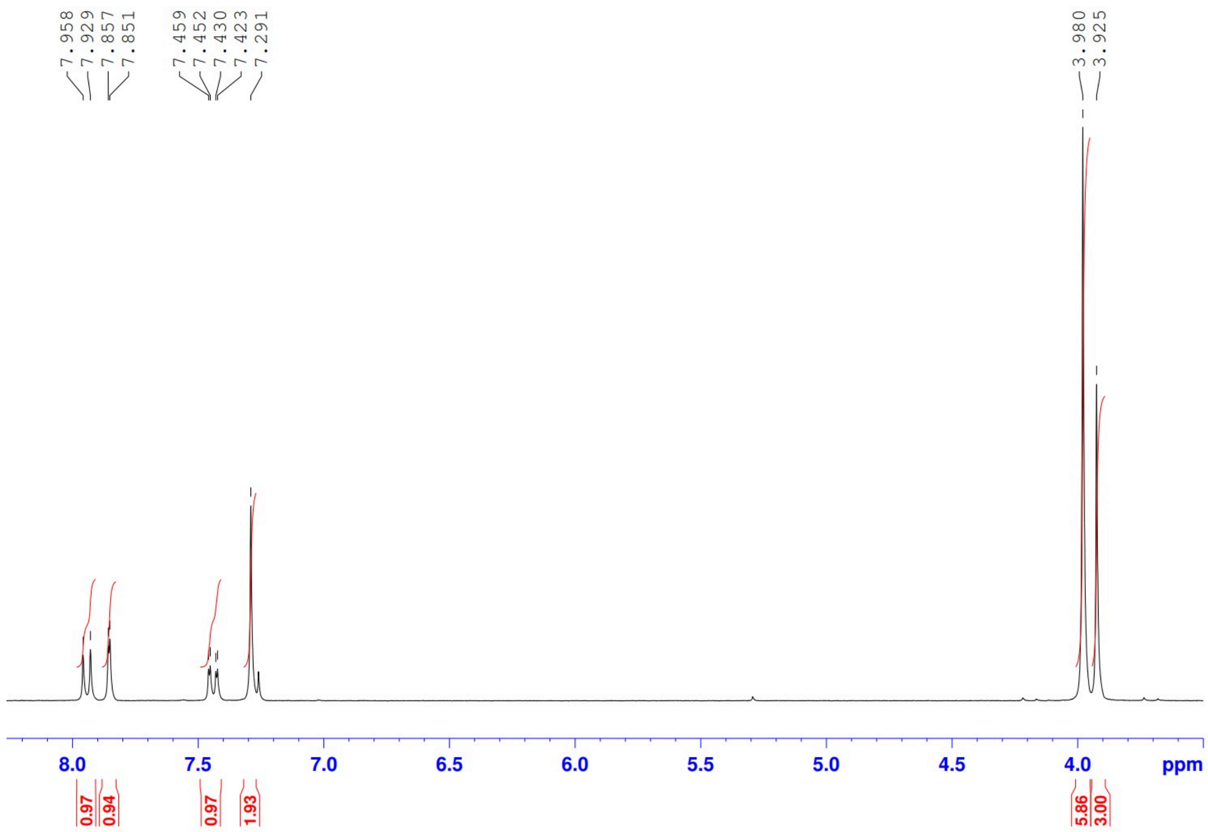

Figure S93. ^{1}**H-NMR (300 MHz, CDCl$_3$) spectrum of 43**

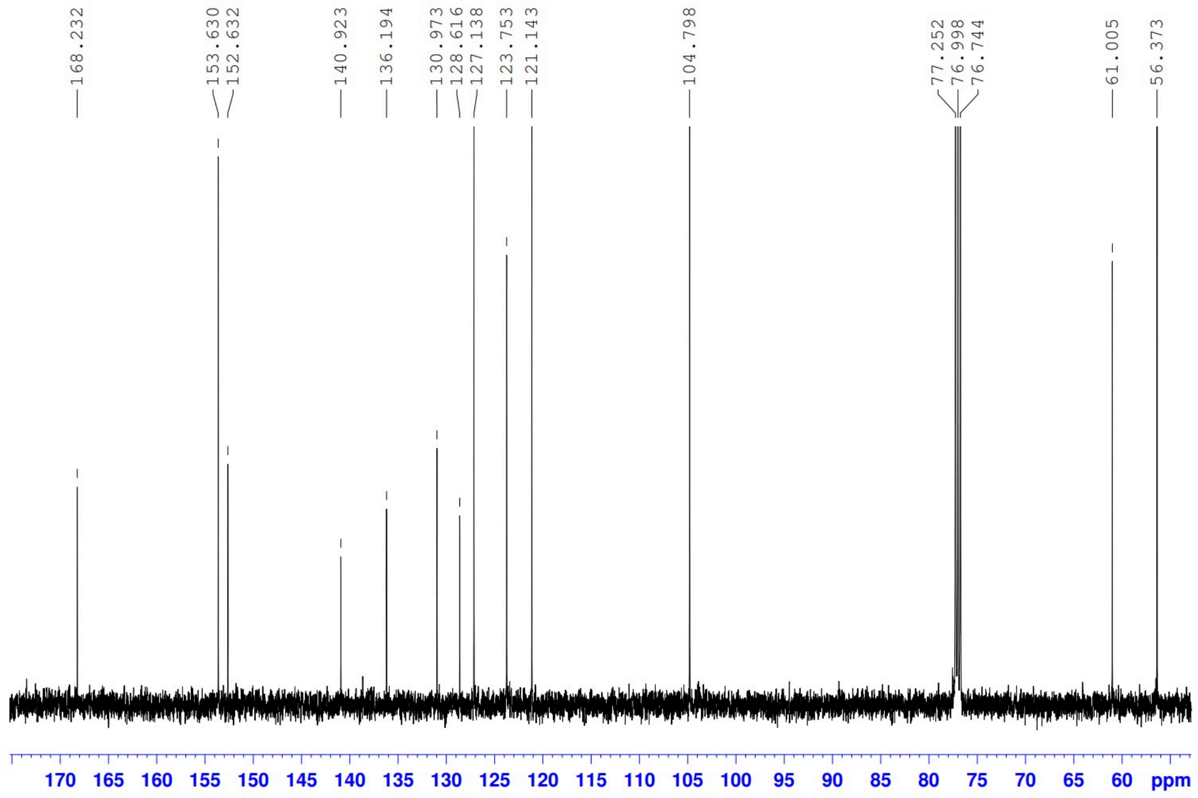

Figure S94. ^{13}**C-NMR (125 MHz, CDCl$_3$) spectrum of 43**

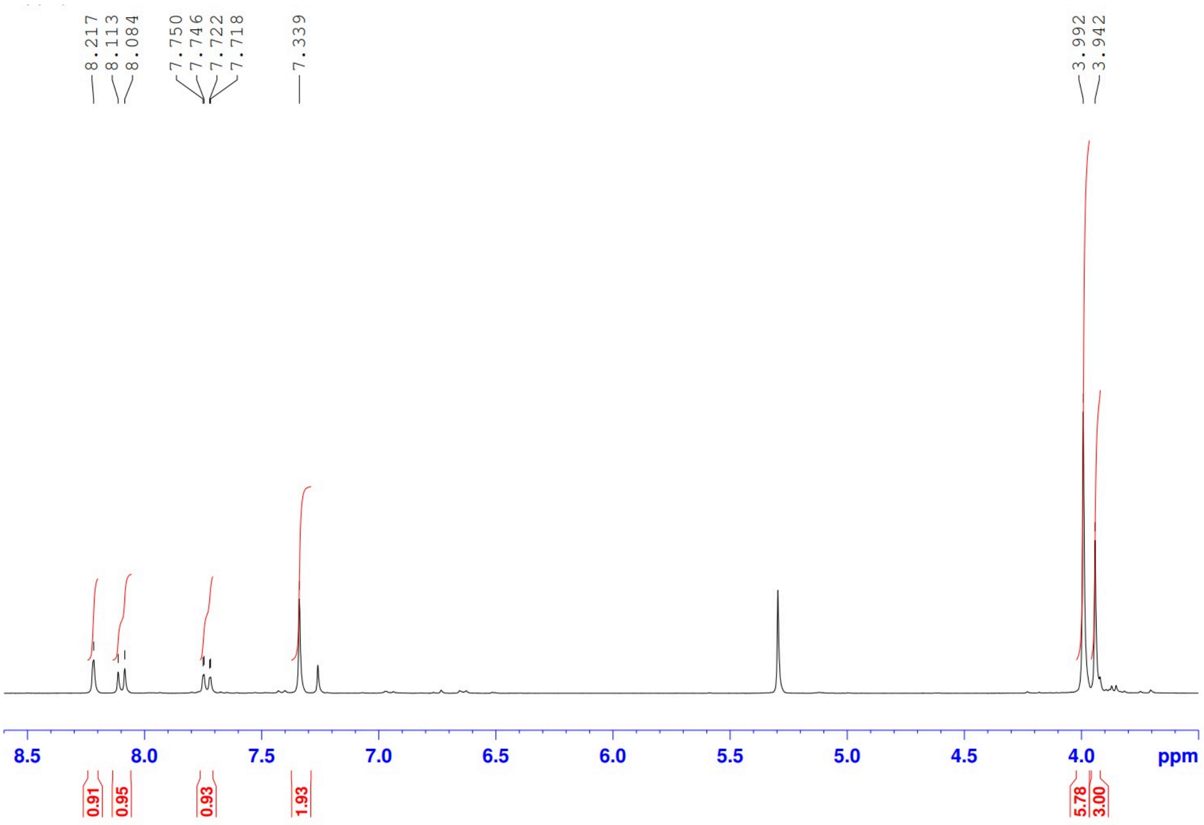

Figure S95. [1]**H-NMR (300 MHz, CDCl₃) spectrum of 44**

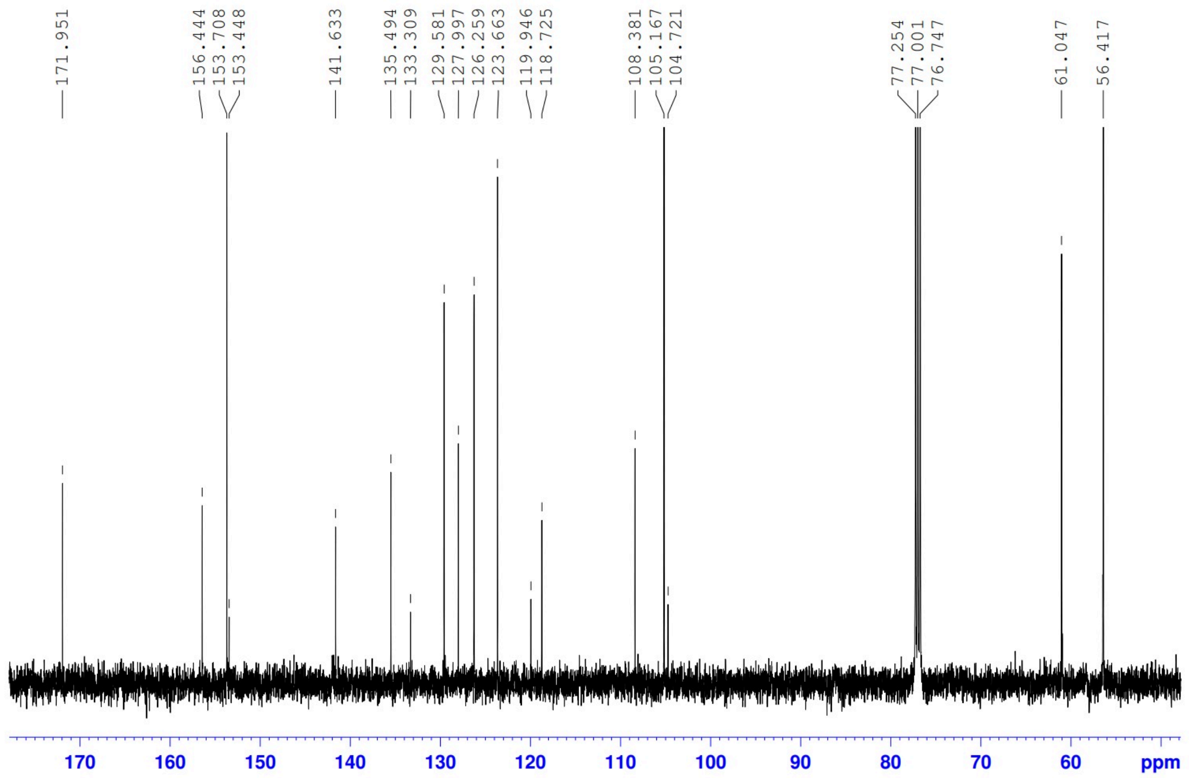

Figure S96. [13]**C-NMR (125 MHz, CDCl₃) spectrum of 44**

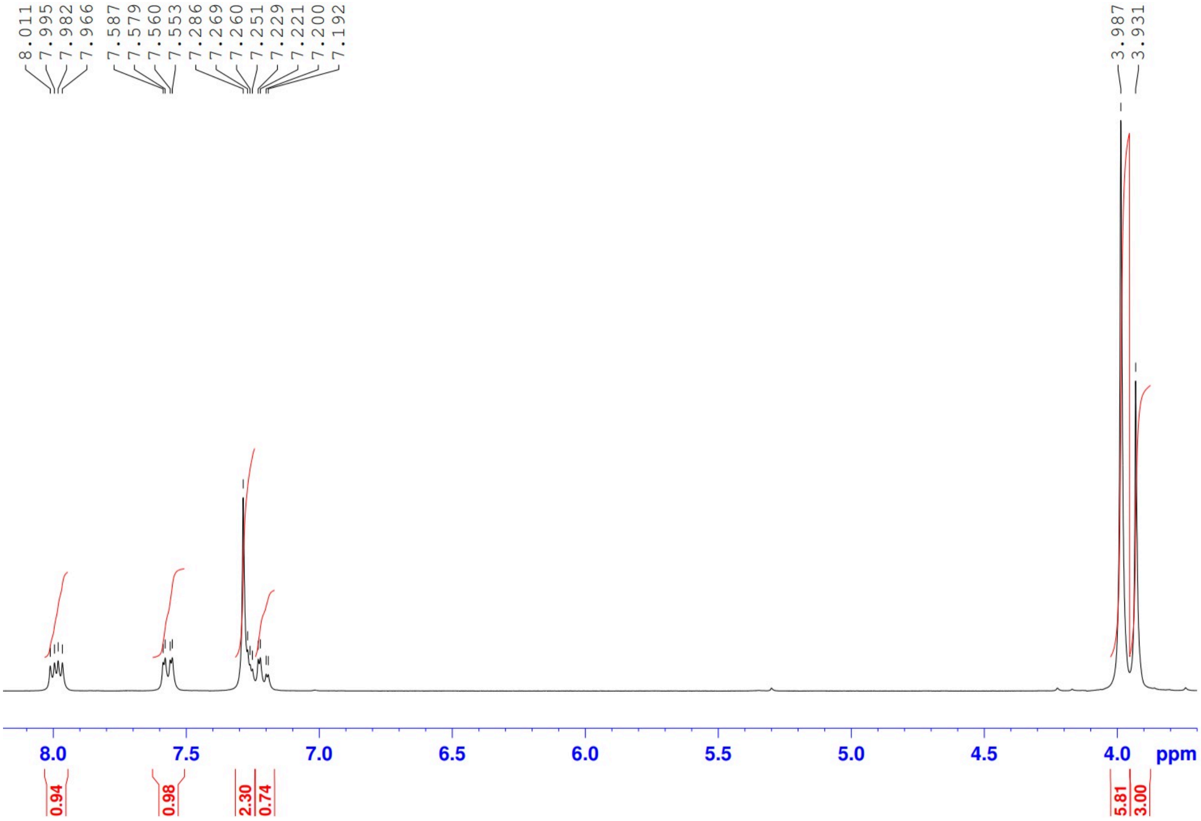

Figure S97. **^{1}H-NMR (300 MHz, $CDCl_3$) spectrum of 45**

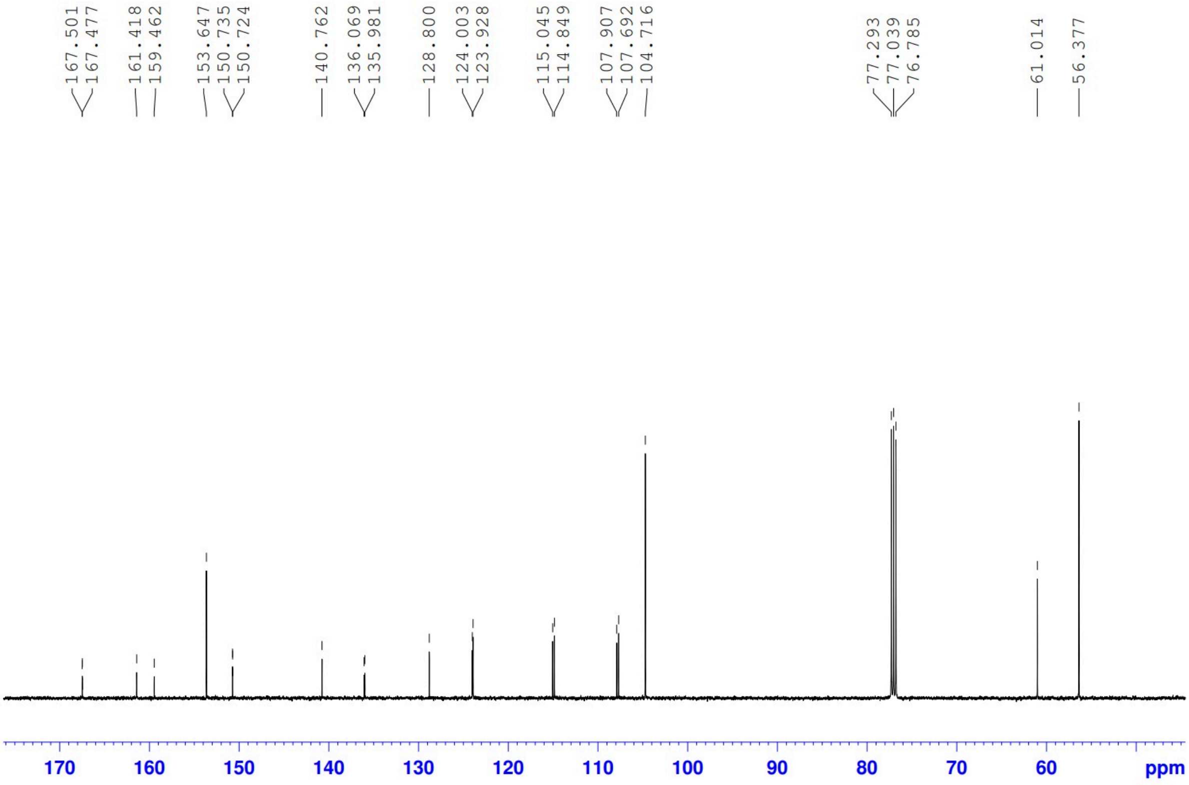

FigureS 98. **^{13}C-NMR (125 MHz, $CDCl_3$) spectrum of 45**

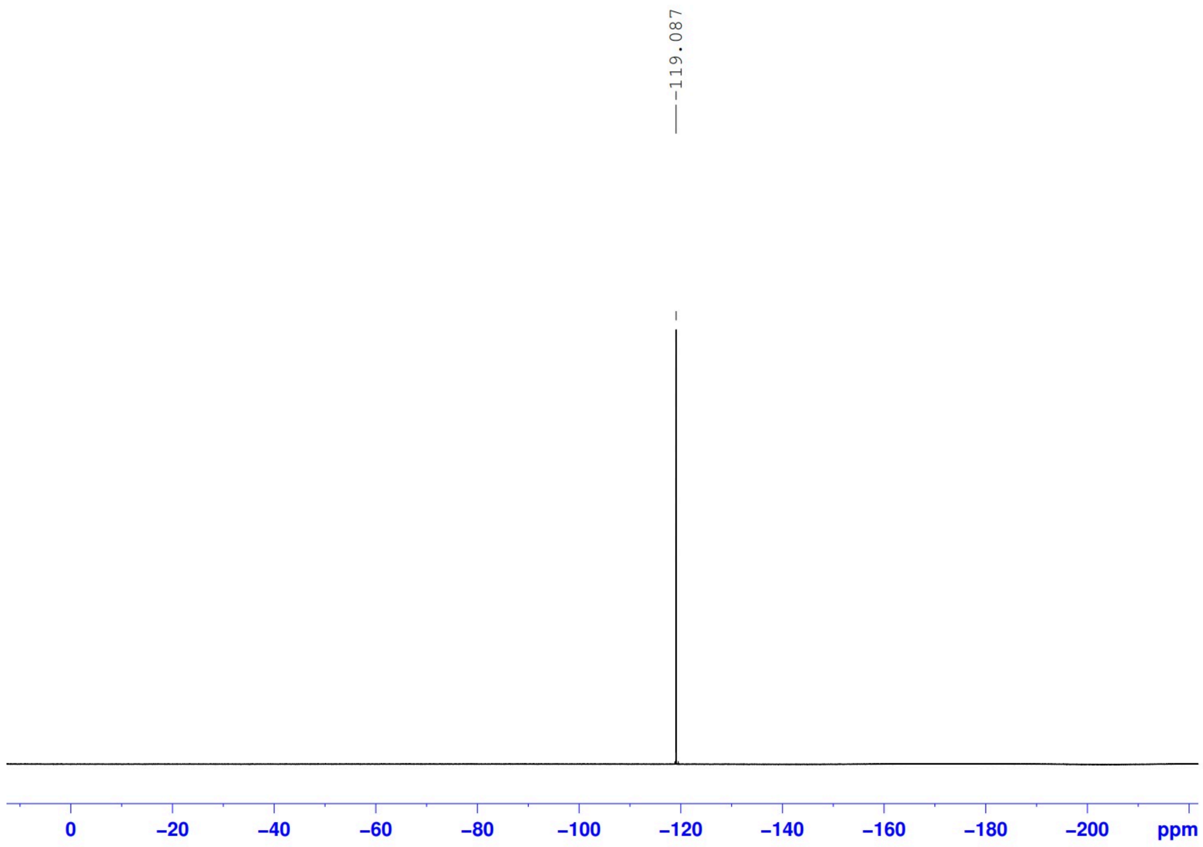

Figure S99. **^{19}F-NMR (376 MHz, CDCl$_3$) spectrum of 45**

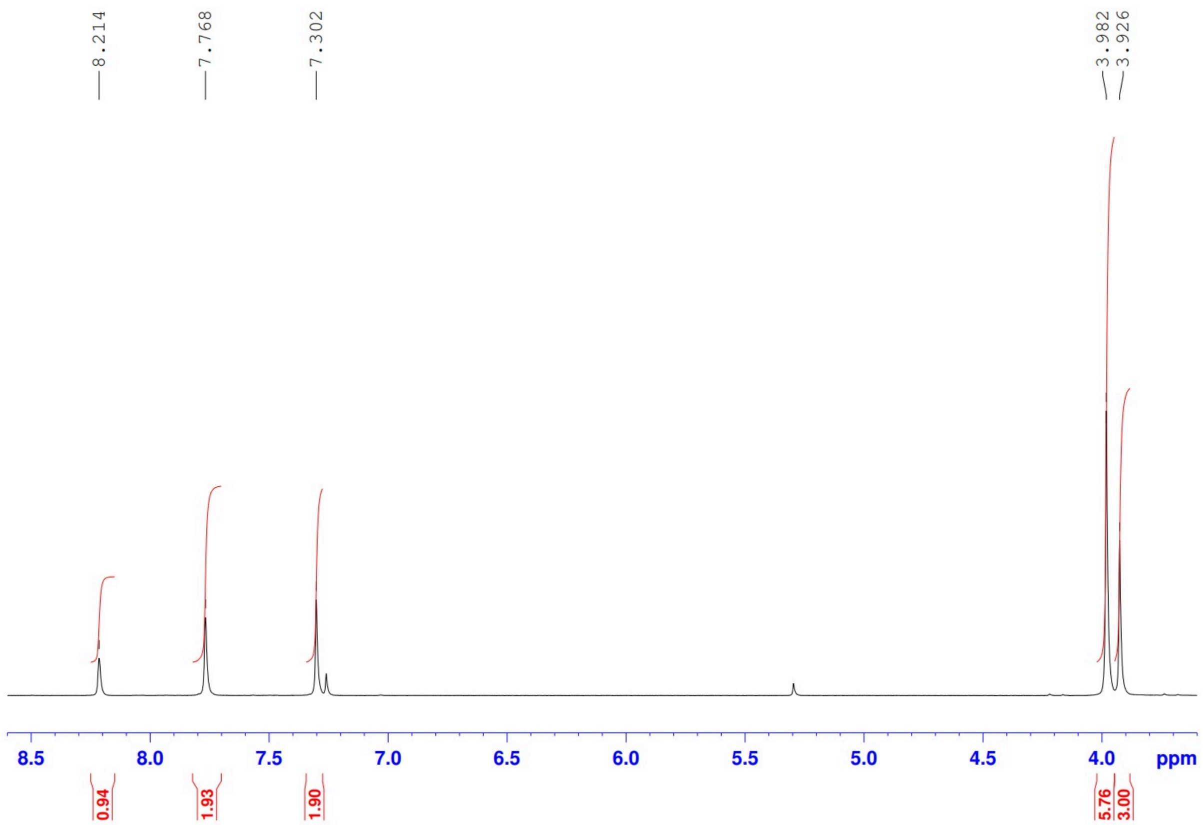

Figure S100. **^{1}H-NMR (300 MHz, CDCl$_3$) spectrum of 46**

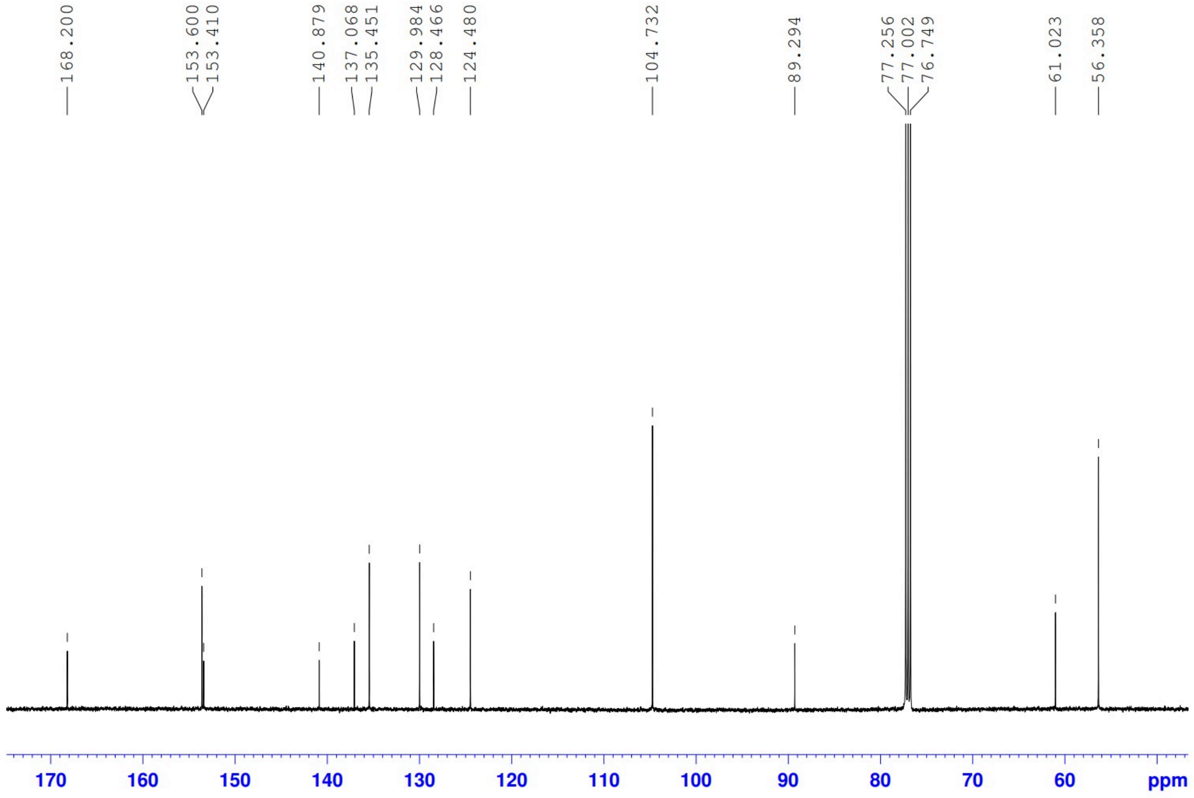

Figure S101. ^{13}**C-NMR (125 MHz, CDCl$_3$) spectrum of 46**

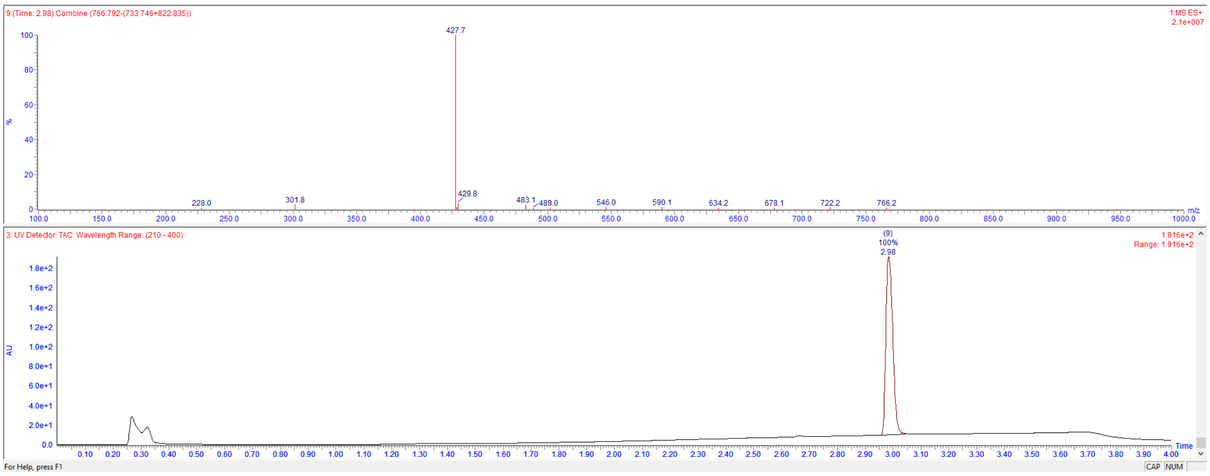

Figure S102. LCMS spectrum of 46

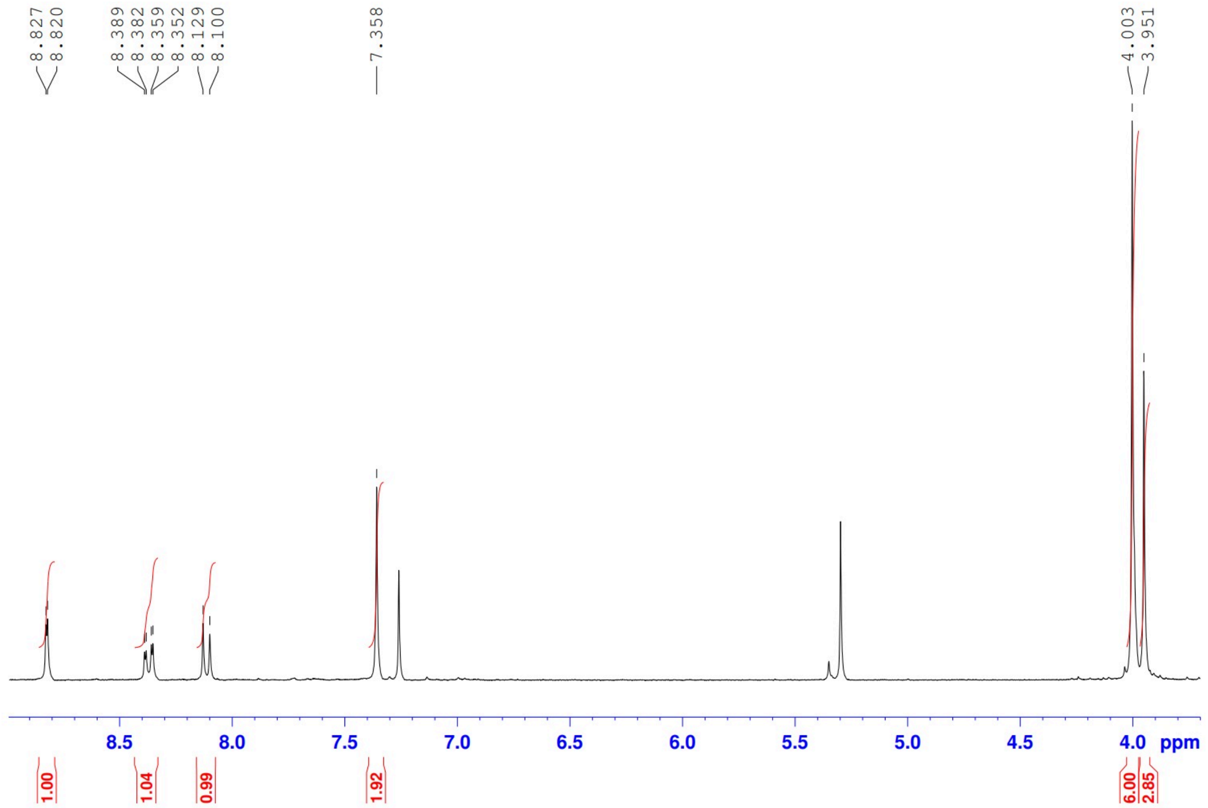

Figure S103. **^{1}H-NMR (300 MHz, CDCl$_3$) spectrum of 47**

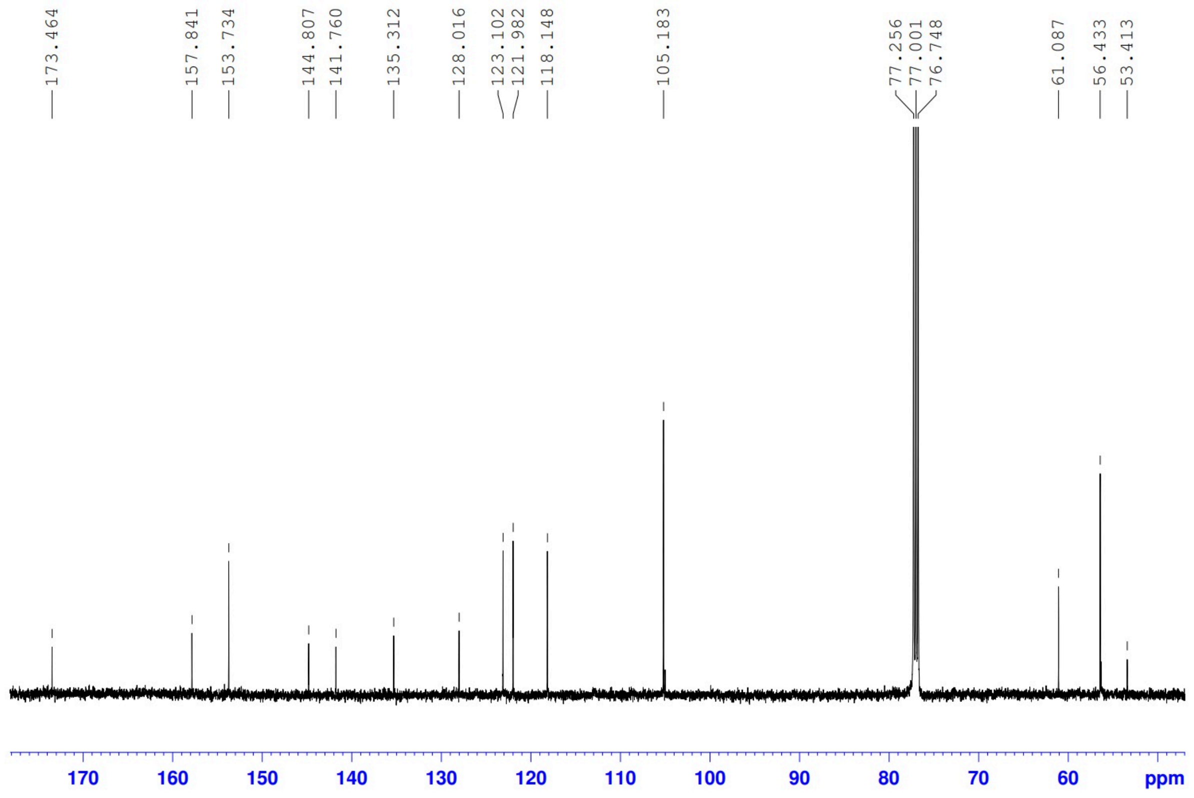

Figure S104. **^{13}C-NMR (125 MHz, CDCl$_3$) spectrum of 47**

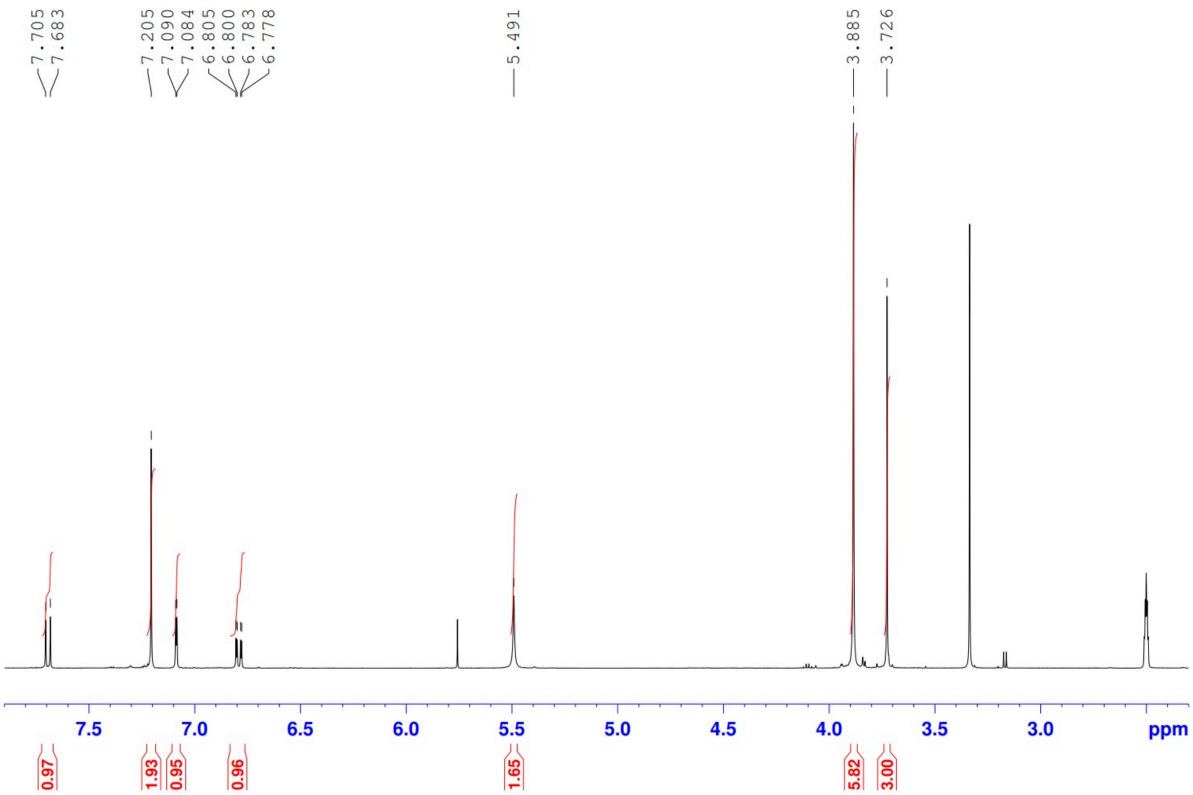

Figure S105. ^{1}**H-NMR (400 MHz, DMSO-d**$_6$**) spectrum of 48**

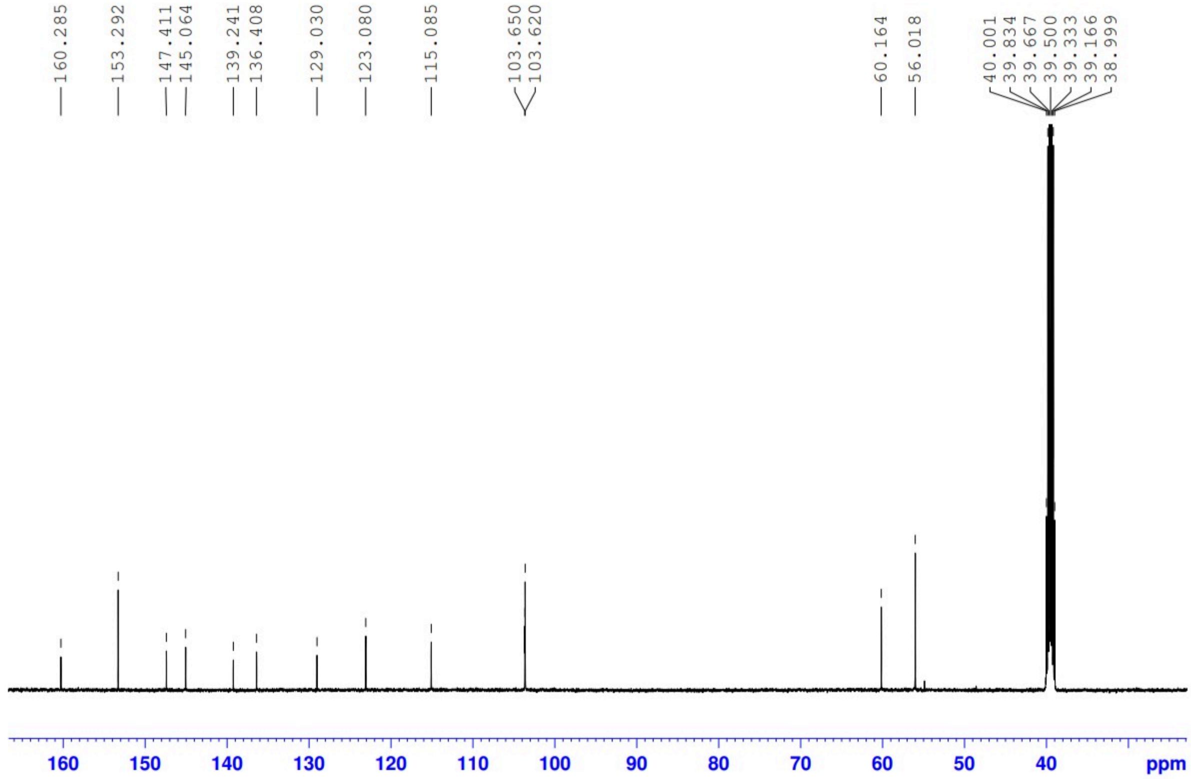

Figure S106. ^{13}**C-NMR (125 MHz, DMSO-d**$_6$**) spectrum of 48**

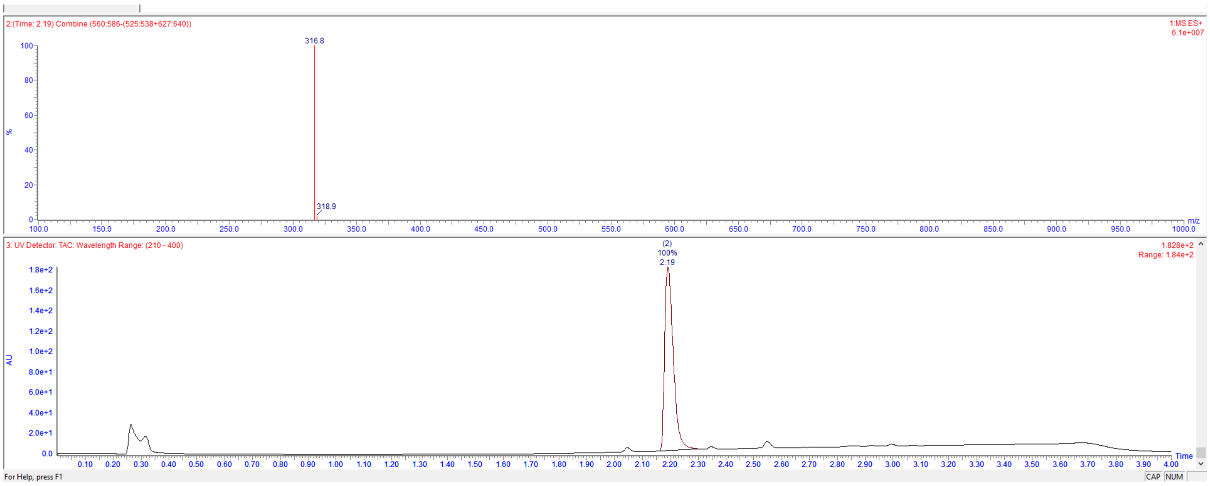

Figure S107. LCMS of 48

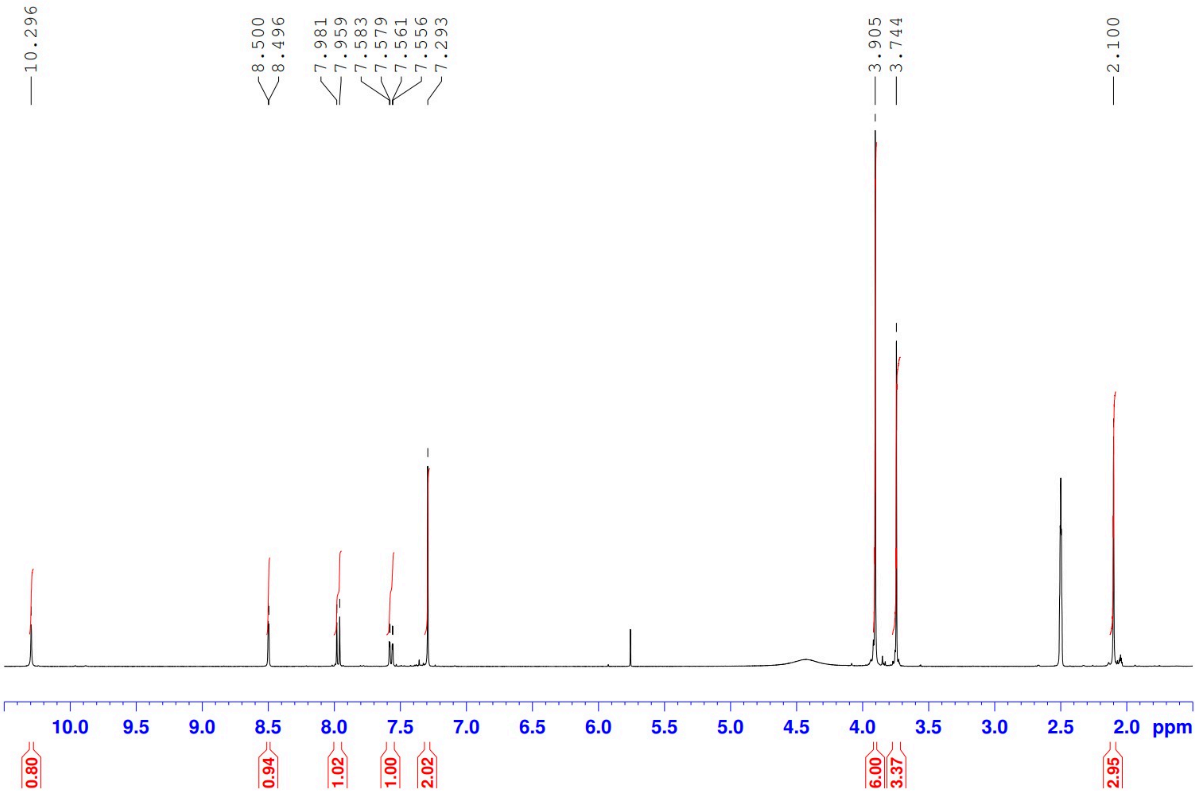

Figure S108. ^{1}H-NMR (400 MHz, DMSO-d$_6$) spectrum of 49

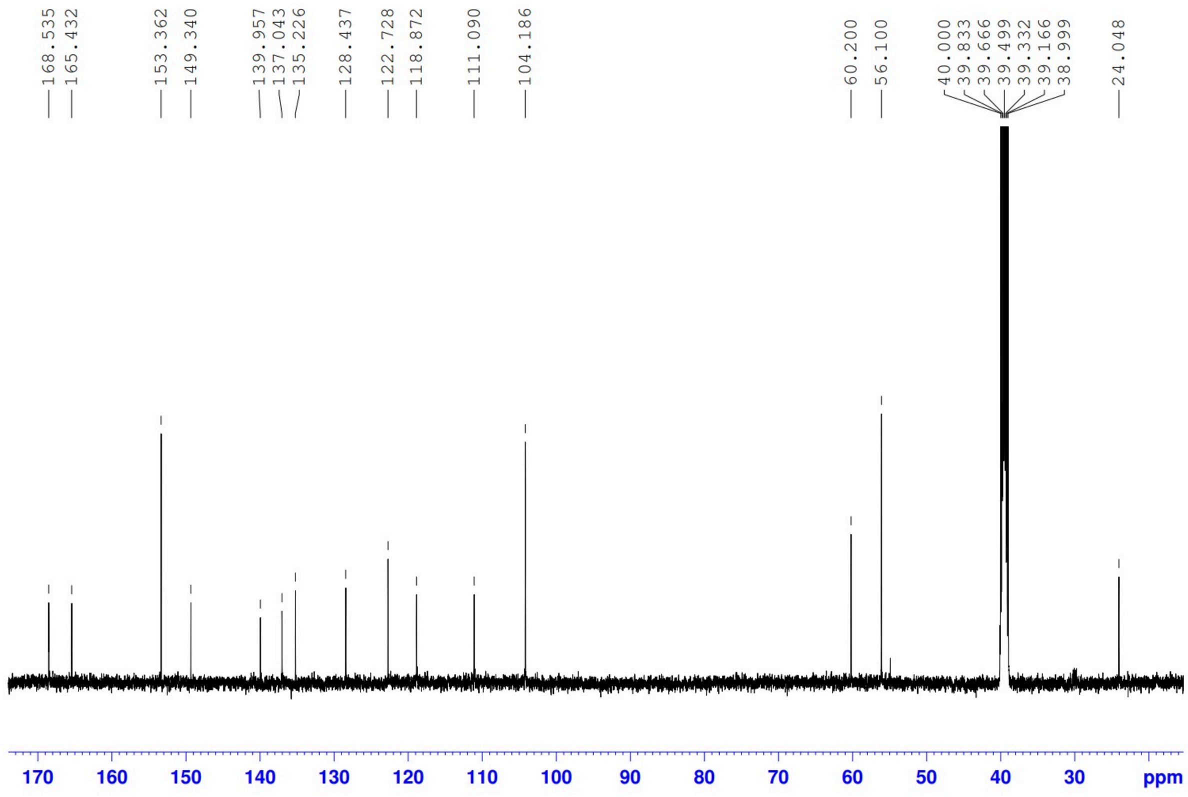

Figure S109. ^{13}**C-NMR (125 MHz, DMSO-d$_6$) spectrum of 49**

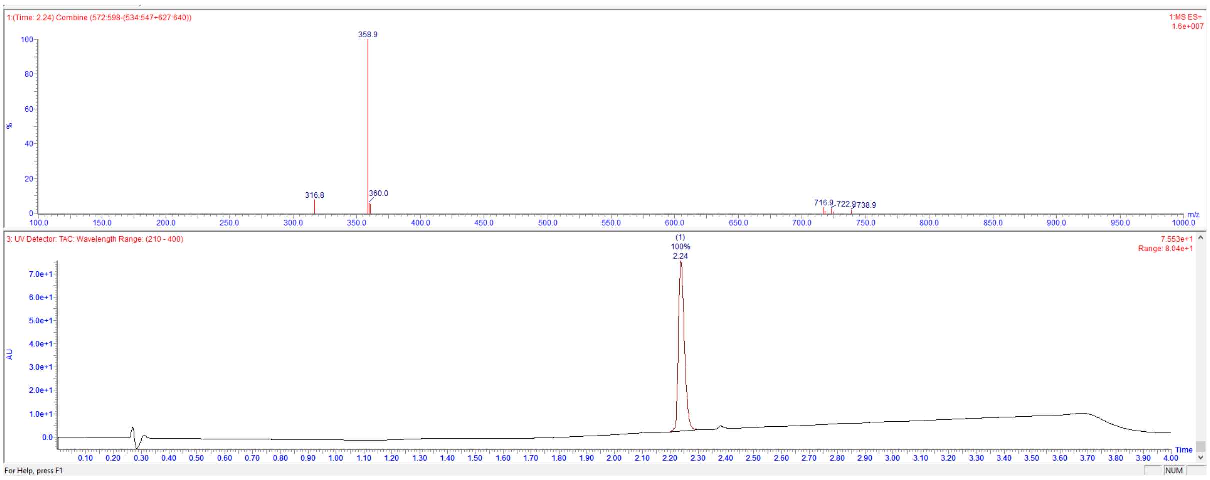

Figure S110. LCMS of 49

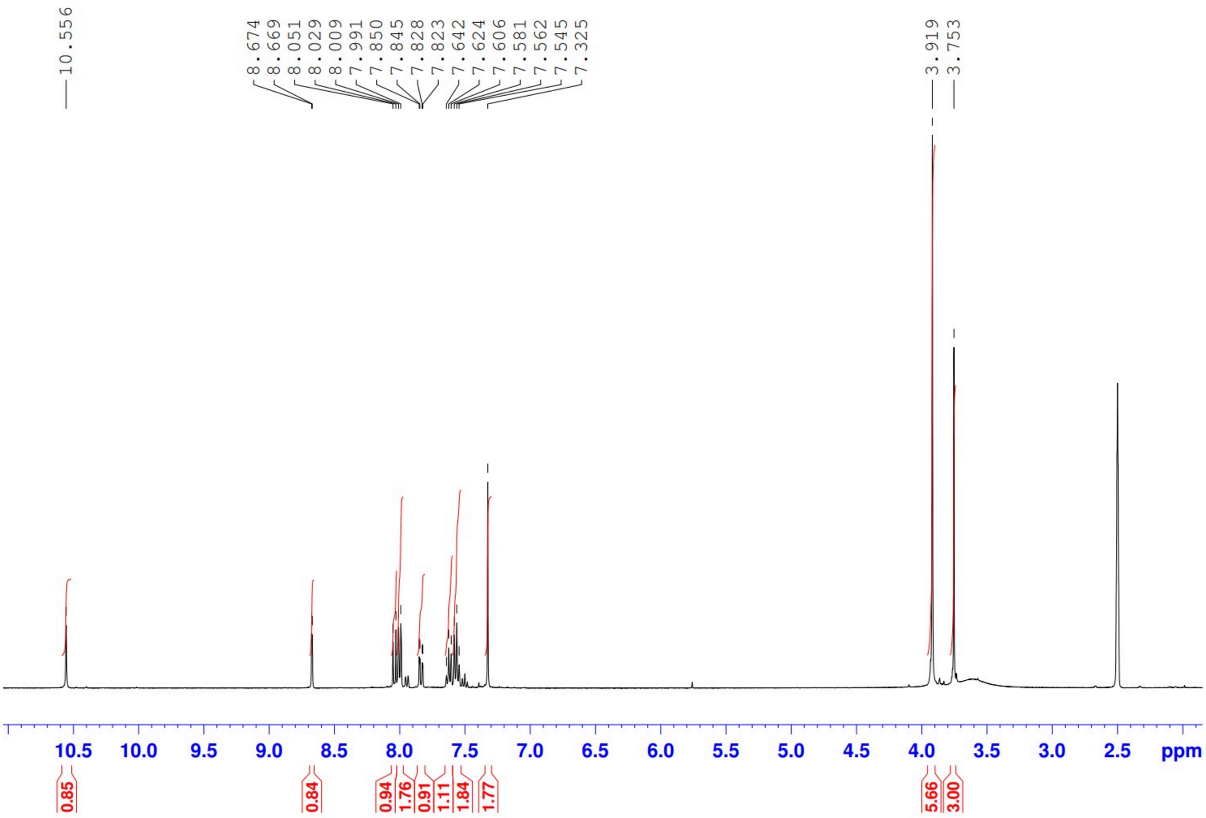

Figure S111. ^{1}**H-NMR (400 MHz, DMSO-d$_6$) spectrum of 50**

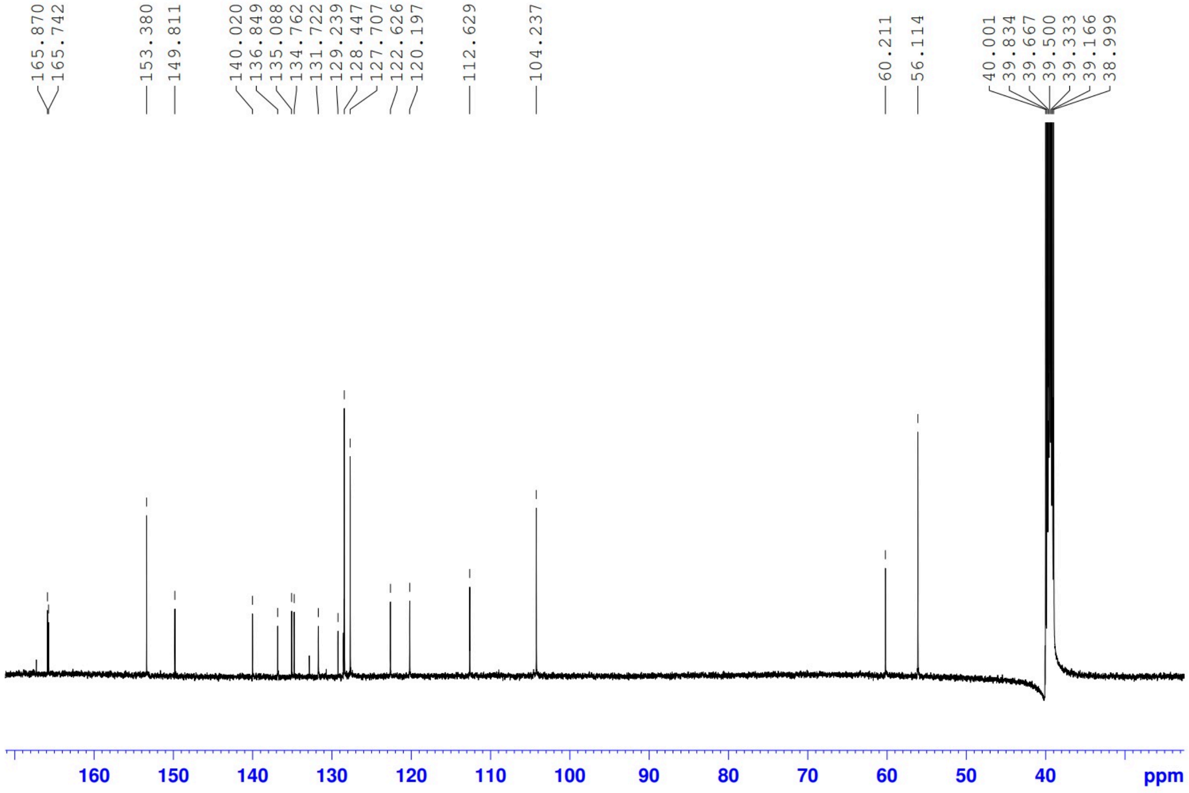

Figure S112. ^{13}**C-NMR (125 MHz, DMSO-d$_6$) spectrum of 50**

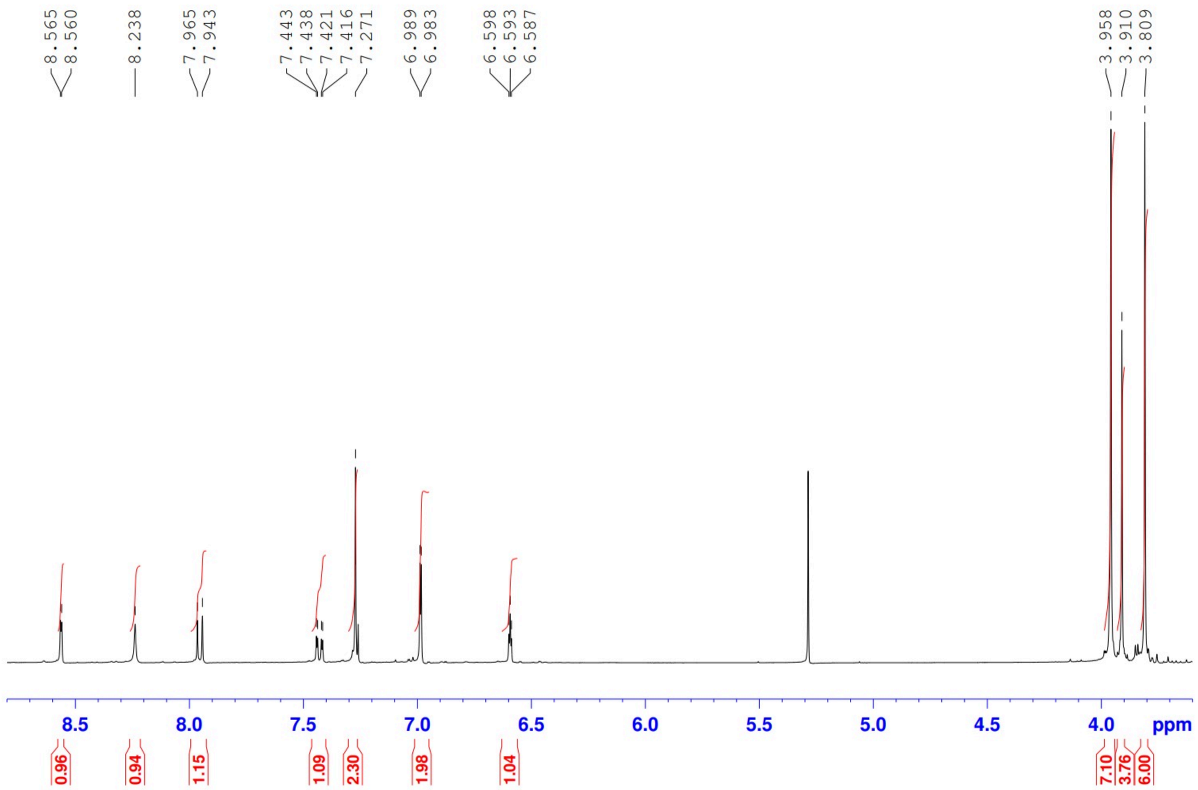

Figure S113. ^{1}H-NMR (400 MHz, $CDCl_3$) spectrum of 51

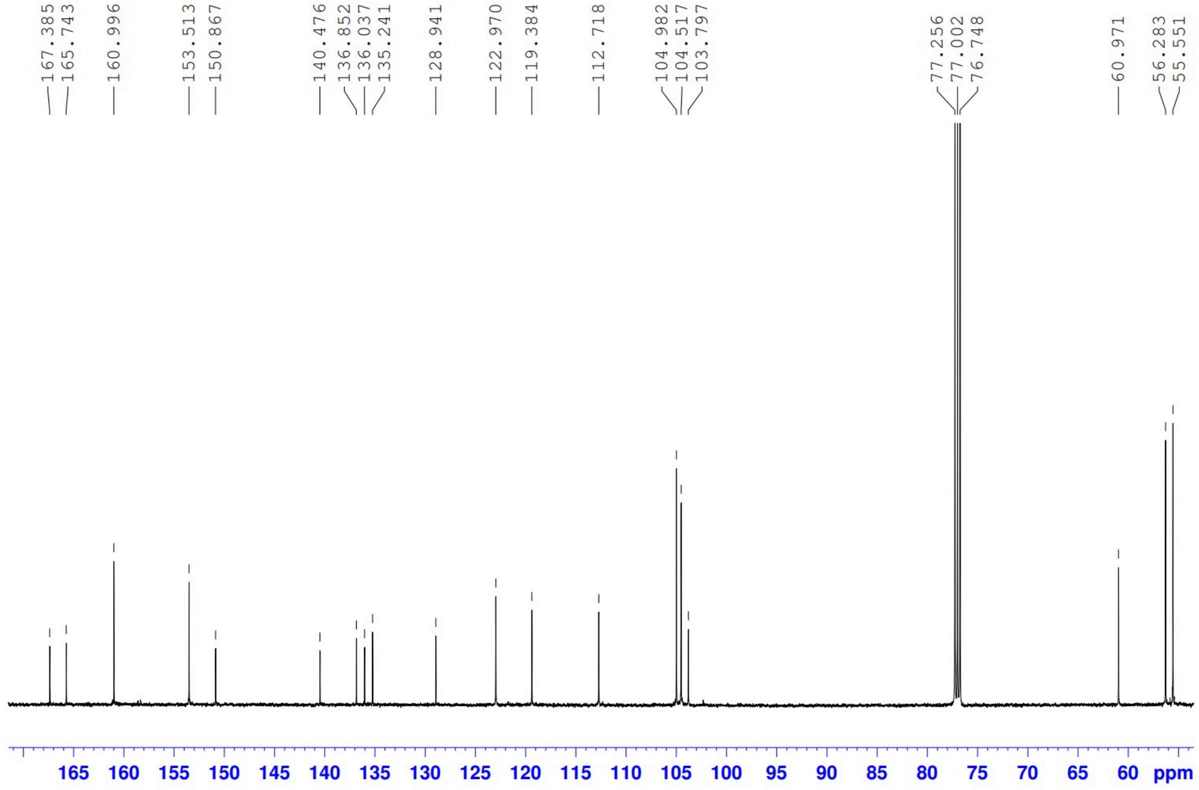

Figure S114. ^{13}C-NMR (125 MHz, $CDCl_3$) spectrum of 51

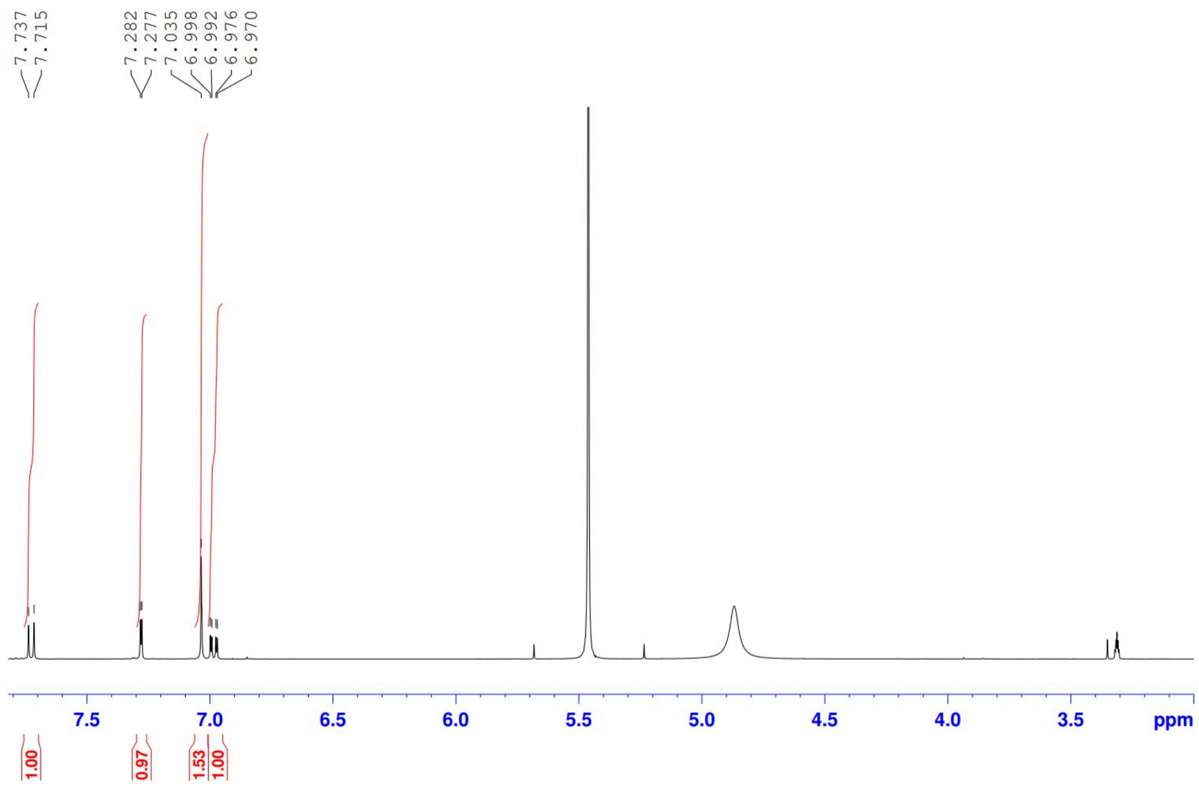

Figure S115. ^{1}H-NMR (400 MHz, methanol-d4) spectrum of 52

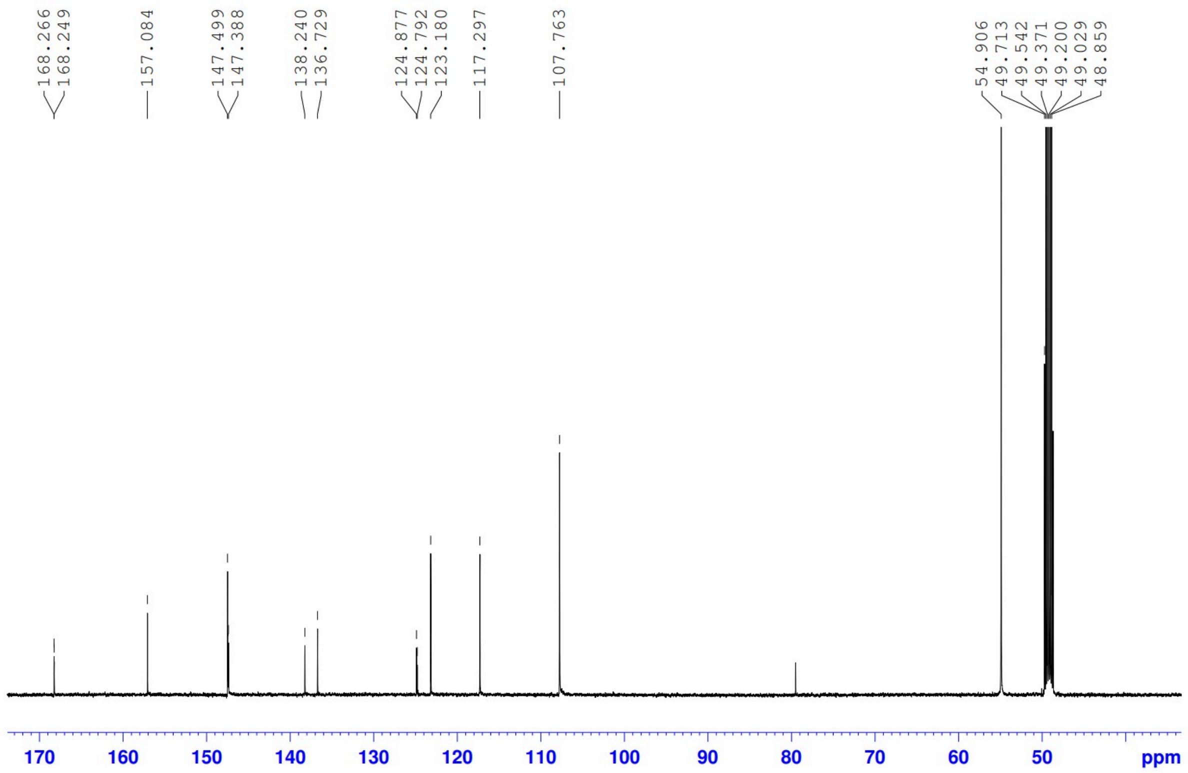

Figure S116. ^{13}C-NMR (125 MHz, methanol-d4) spectrum of 52

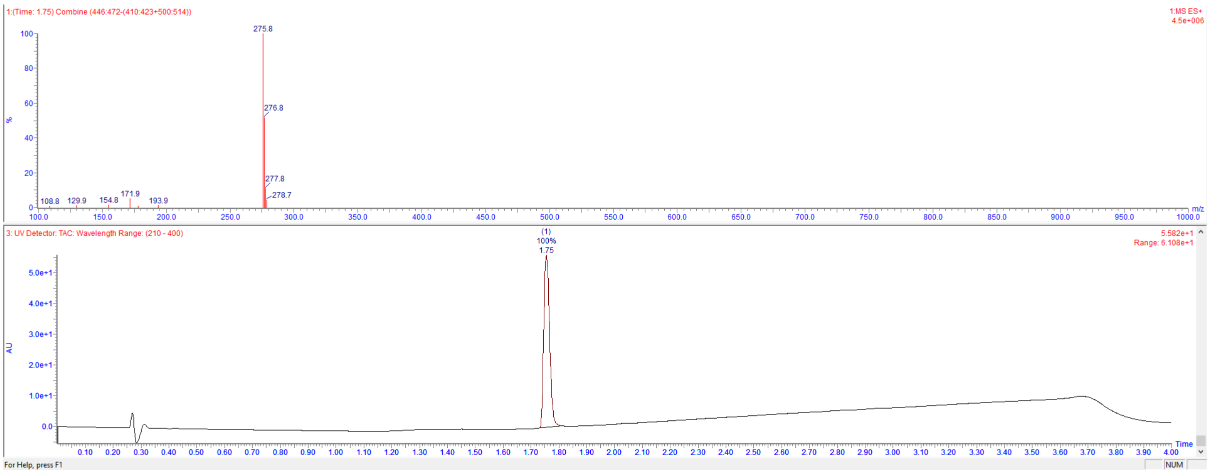

Figure S117. LCMS of 52

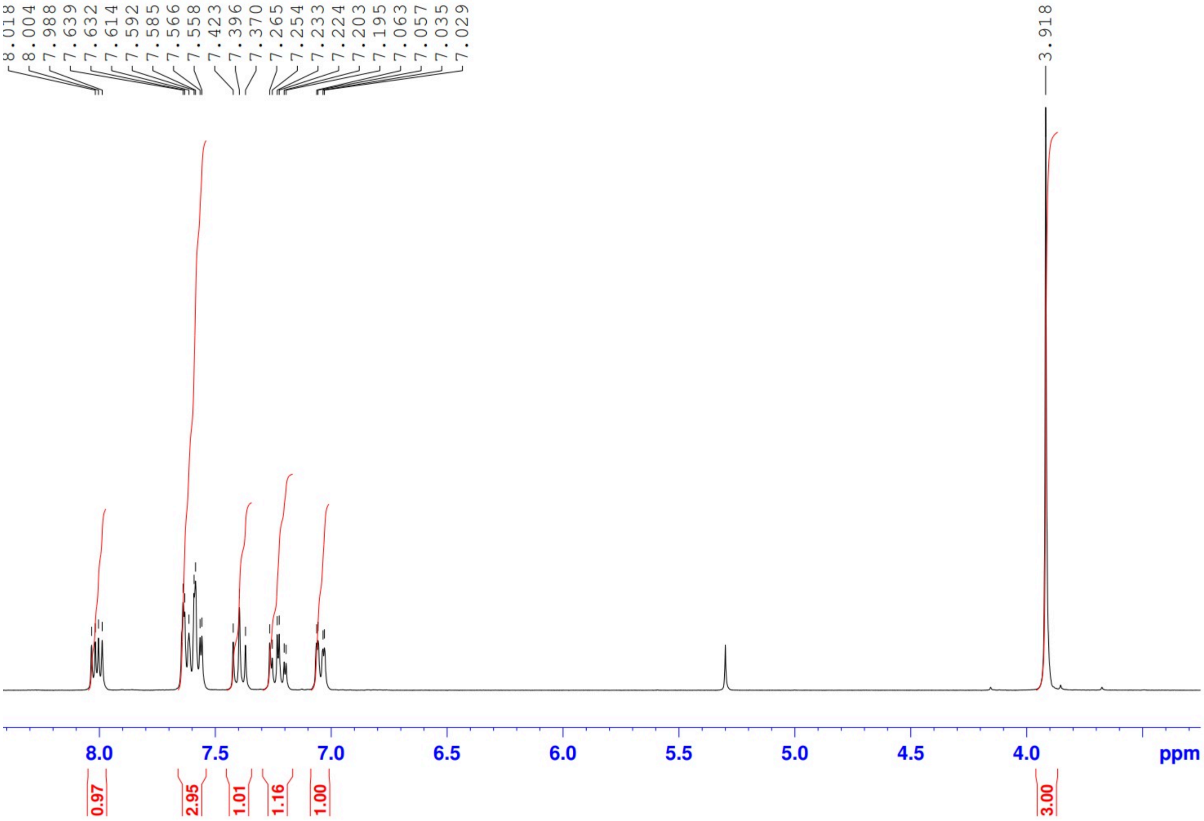

Figure S118. ¹H-NMR (300 MHz, $CDCl_3$) spectrum of 53

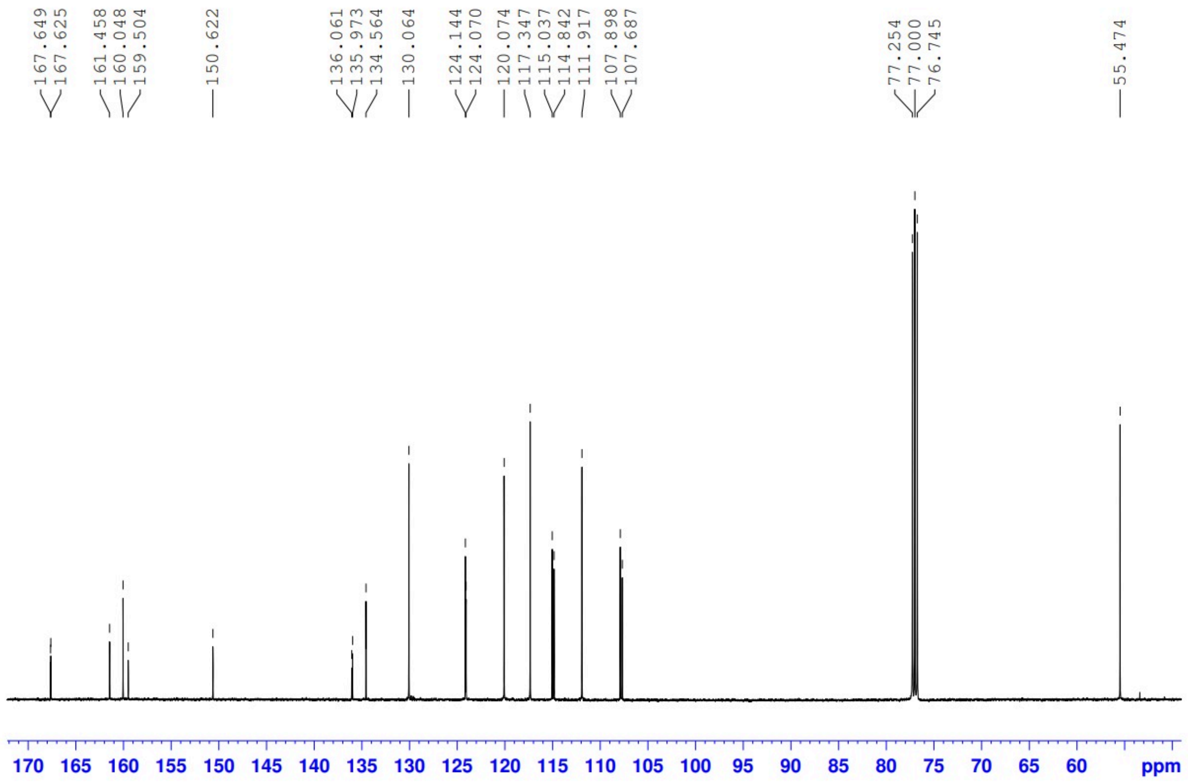

Figure S119. ^{13}**C-NMR (125 MHz, CDCl$_3$) spectrum of 53**

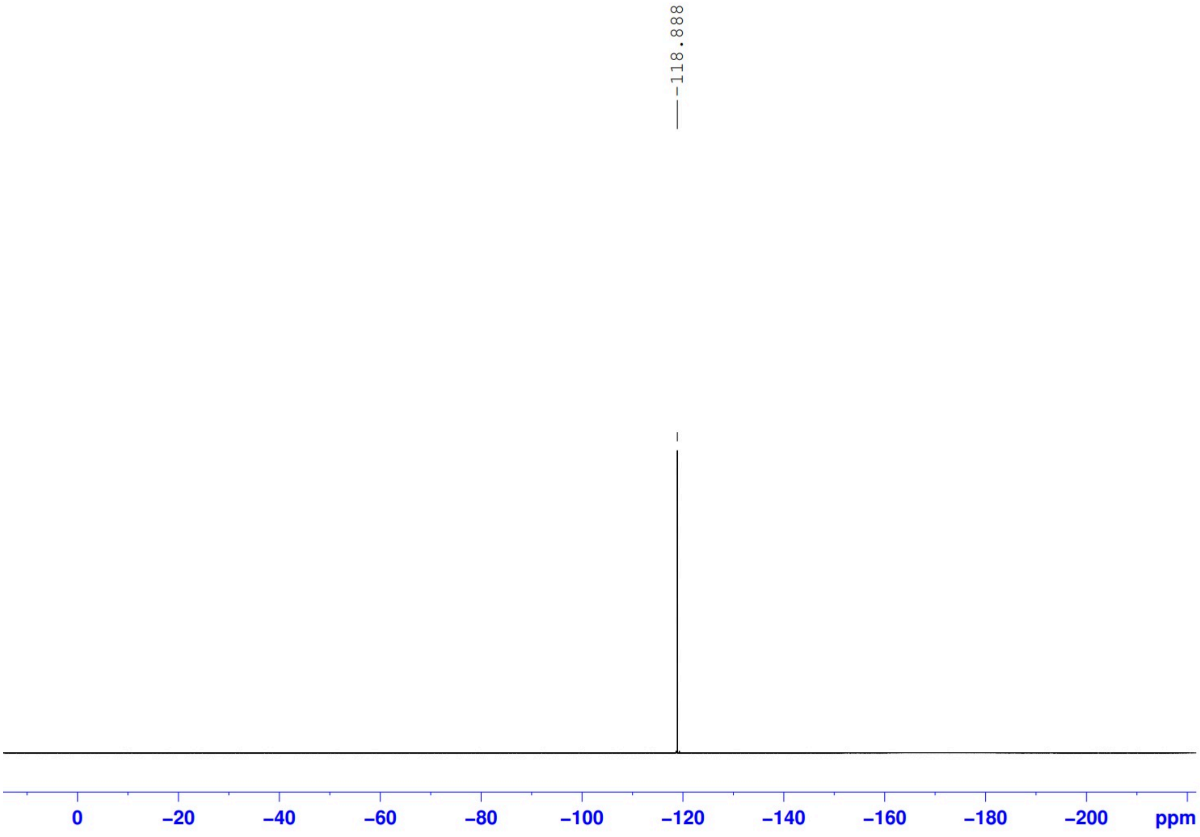

Figure S120. ^{19}**F-NMR (376 MHz, CDCl$_3$) spectrum of 53**

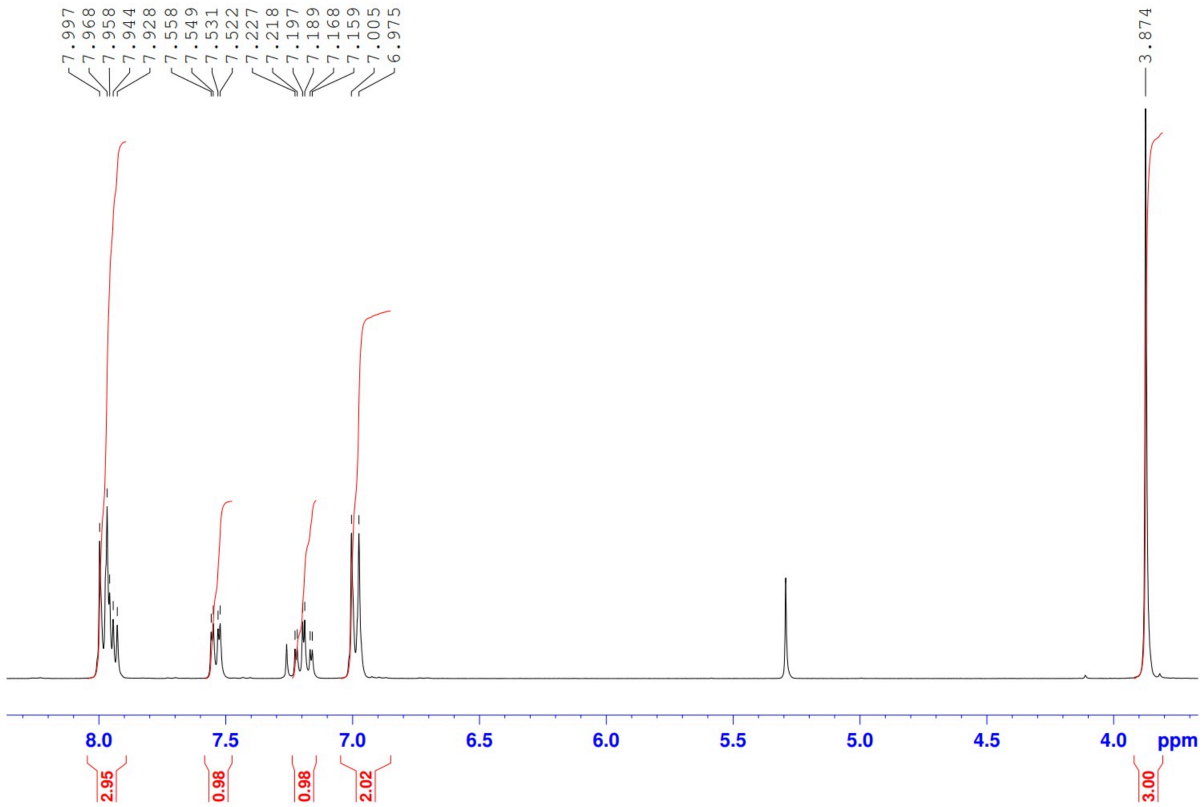

Figure S121. ^{1}**H-NMR (300 MHz, CDCl$_3$) spectrum of 54**

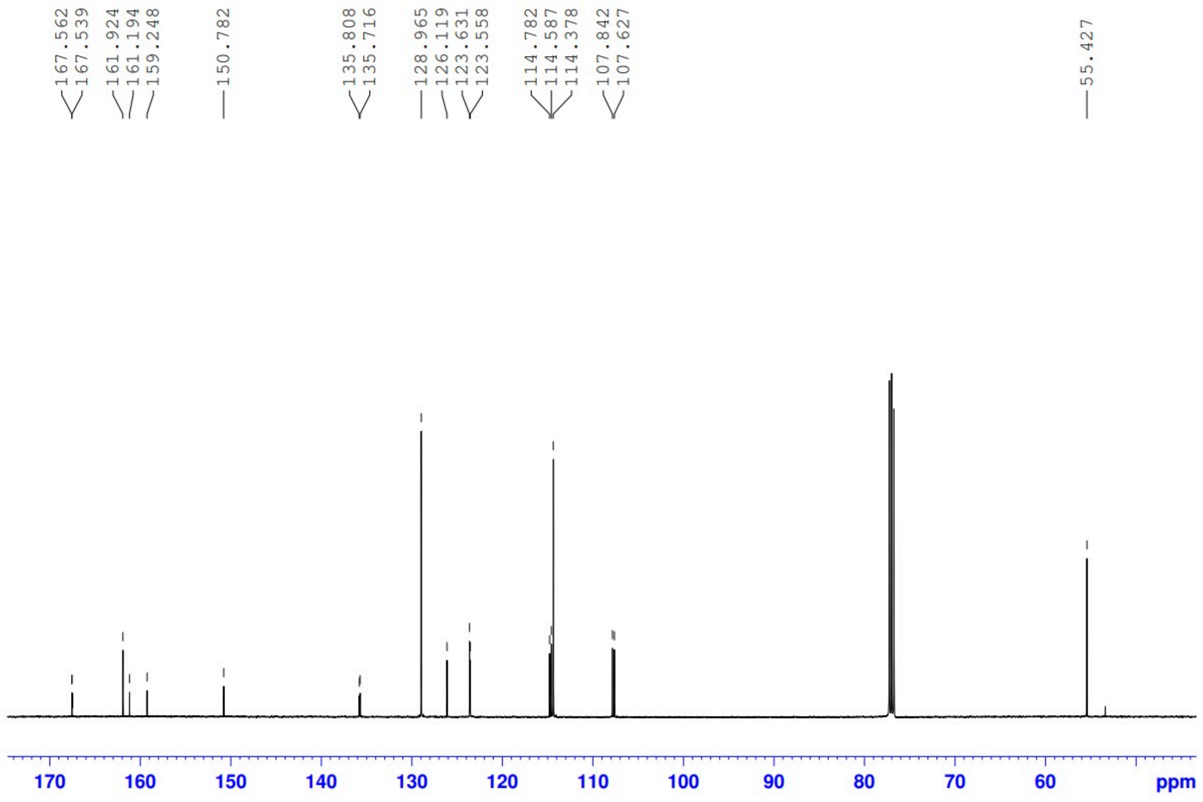

Figure S122. ^{13}**C-NMR (125 MHz, CDCl$_3$) spectrum of 54**

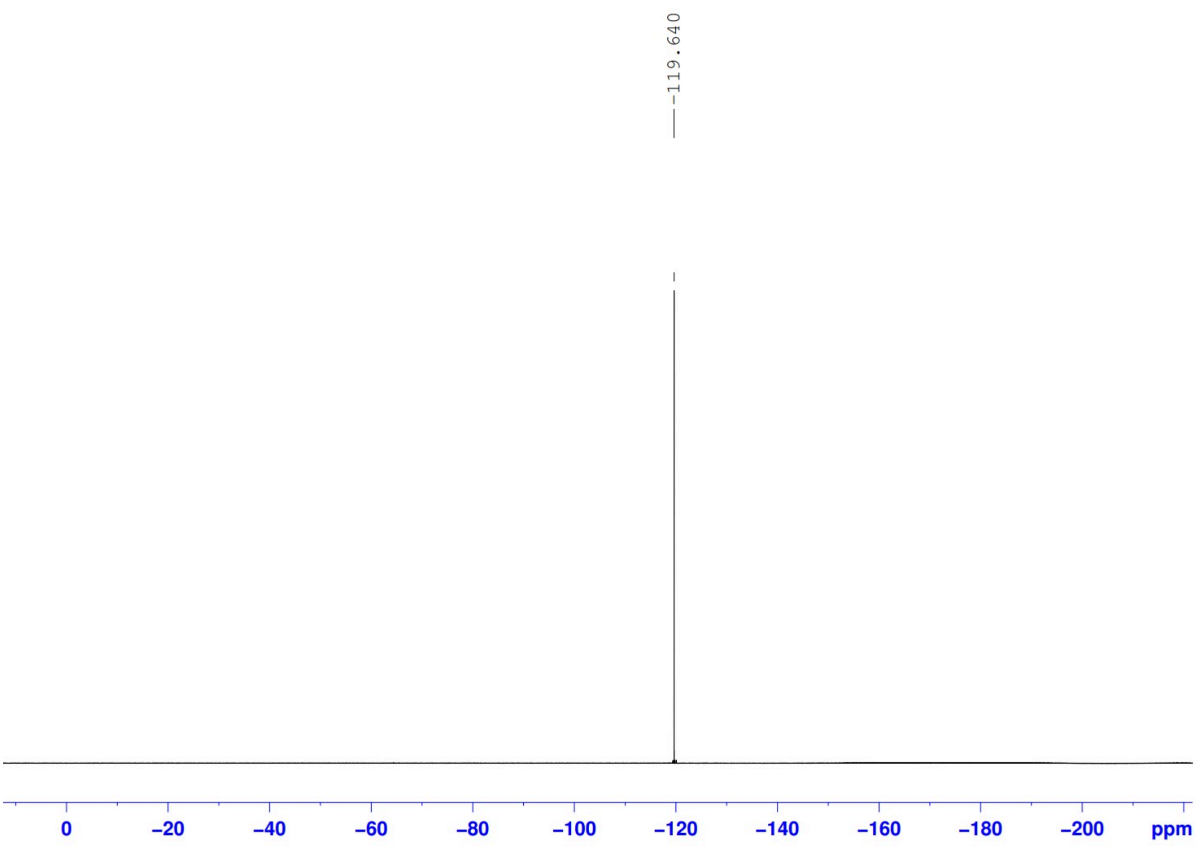

Figure S123. ^{19}F-NMR (376 MHz, $CDCl_3$) spectrum of 54

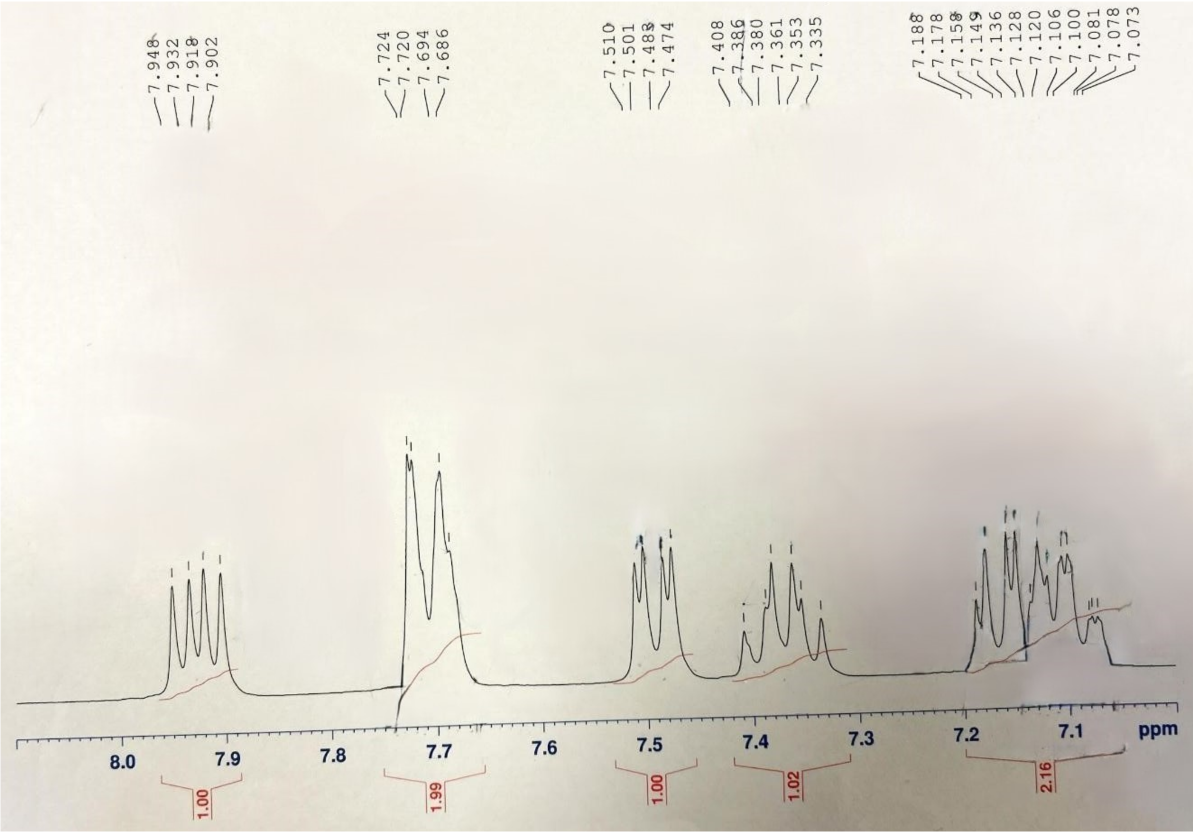

Figure S124. ^{1}H-NMR (300 MHz, $CDCl_3$) spectrum of 55

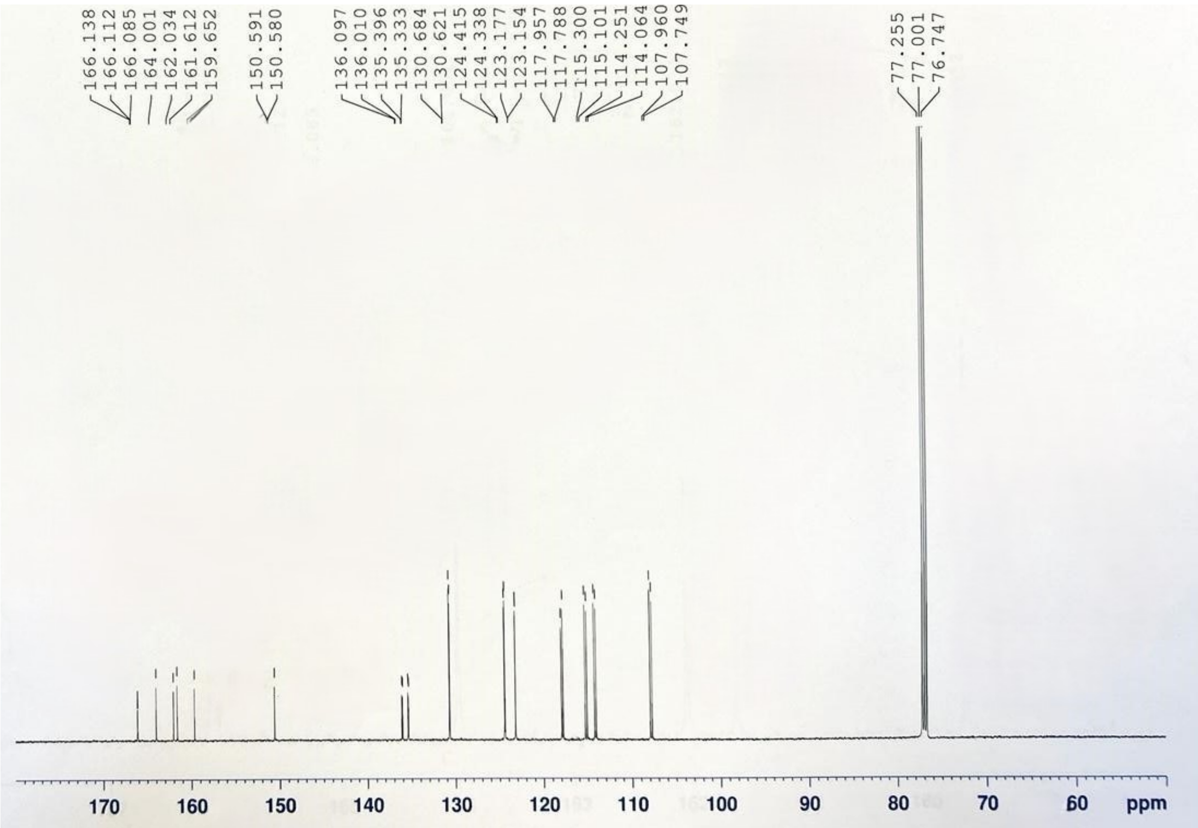

Figure S125. ^{13}**C-NMR (125 MHz, CDCl$_3$) spectrum of 55**

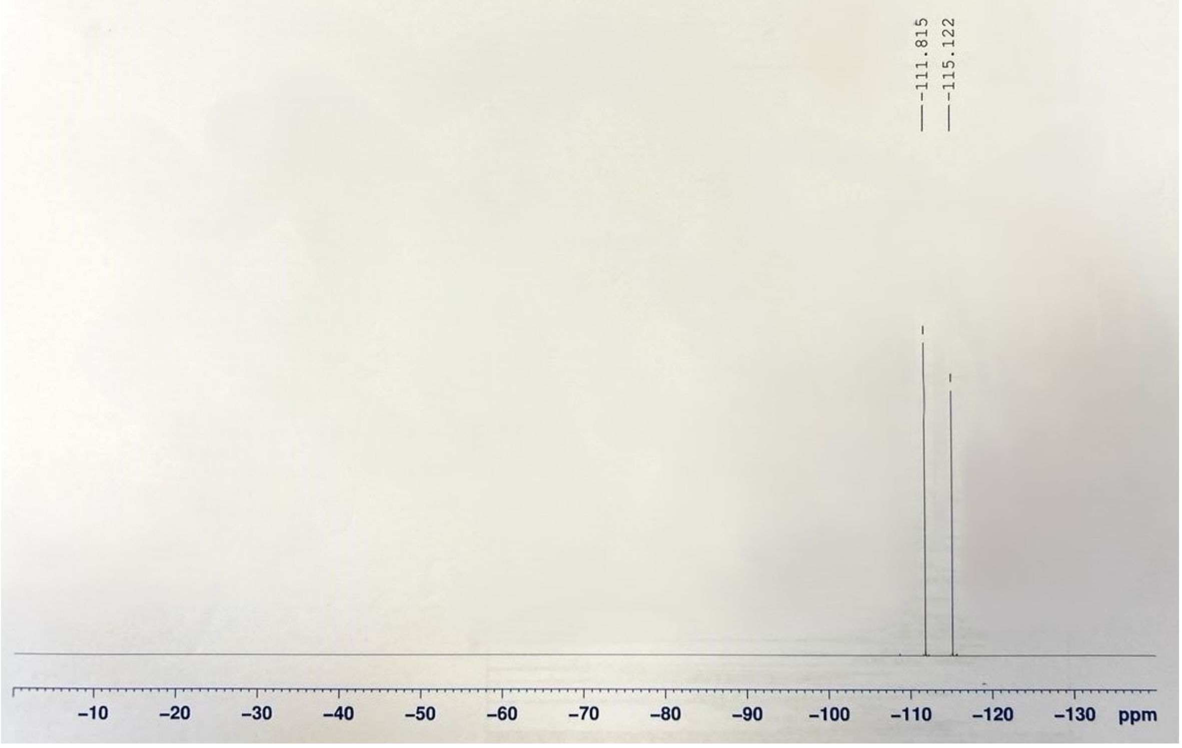

Figure S126. ^{19}**F-NMR (376 MHz, CDCl$_3$) spectrum of 55**

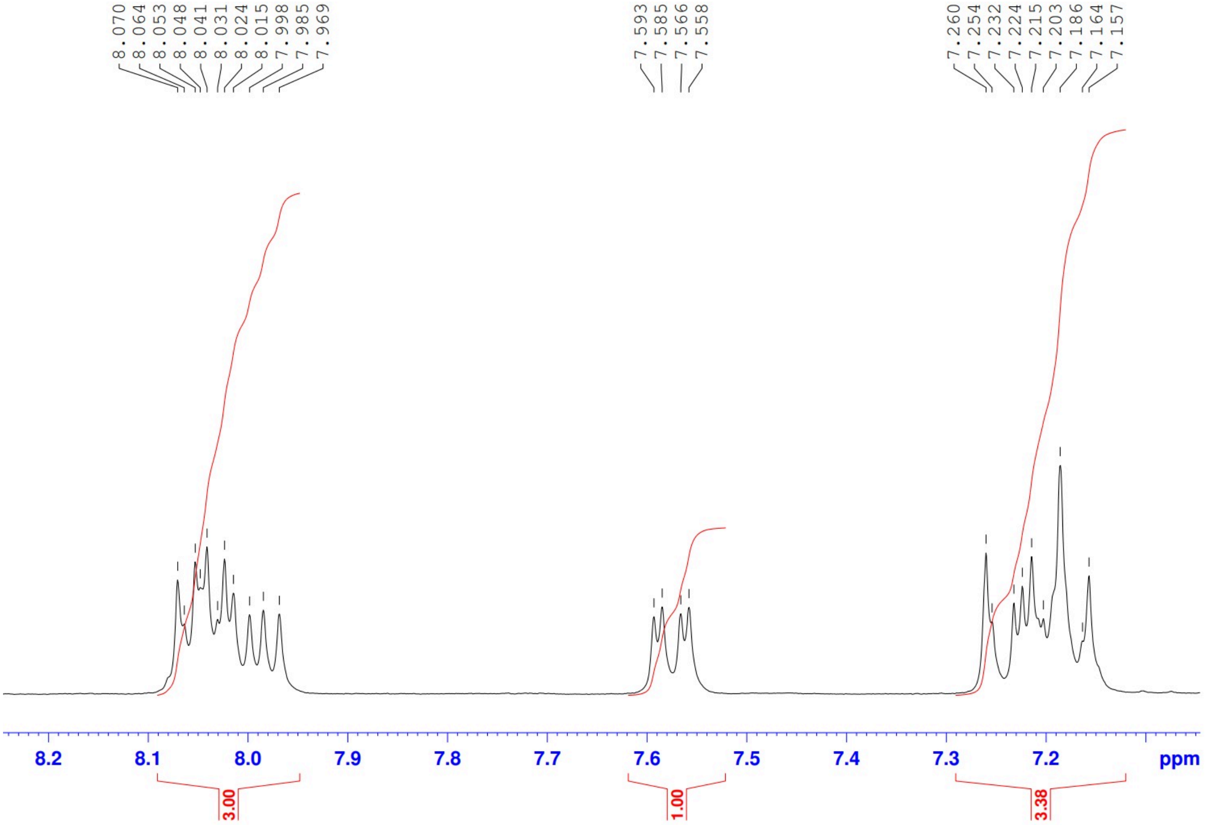

Figure S127. ^{1}**H-NMR (300 MHz,** $CDCl_3$**) spectrum of 56**

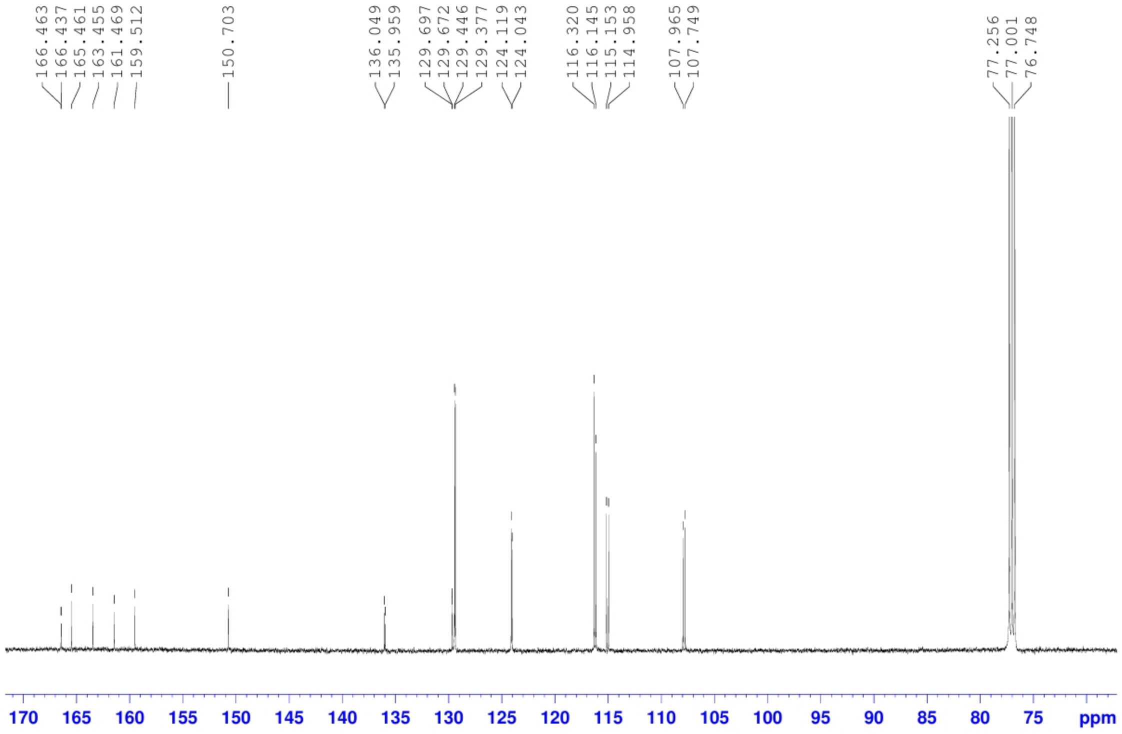

Figure S128. ^{13}**C-NMR (125 MHz,** $CDCl_3$**) spectrum of 56**

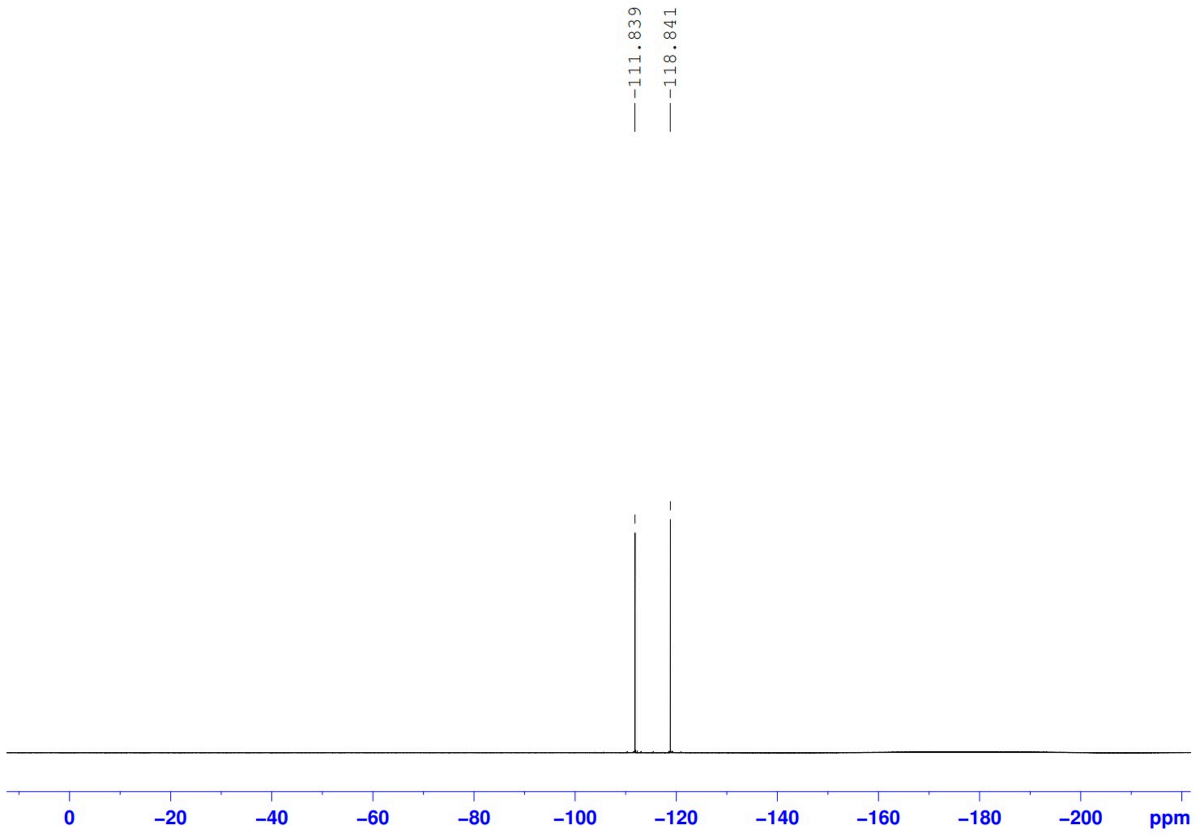

Figure S129. [19]**F-NMR (376 MHz, CDCl$_3$) spectrum of 56**

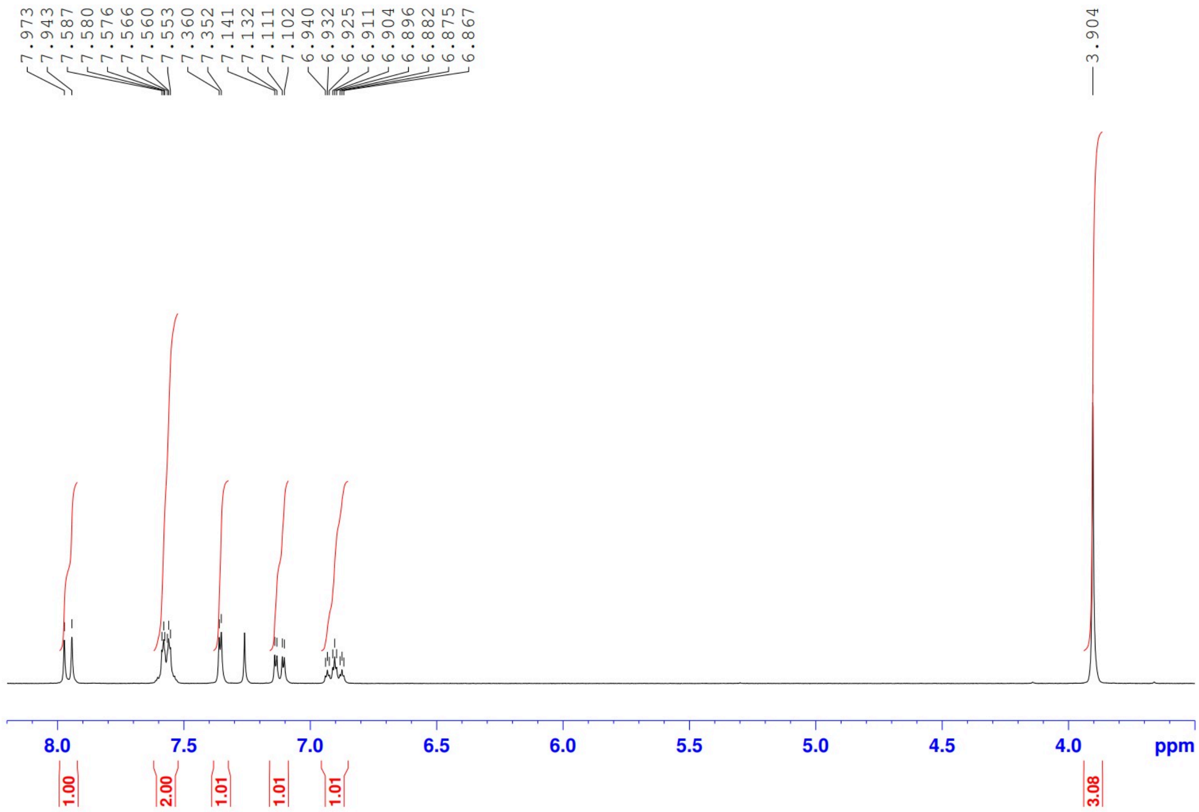

Figure S130. [1]**H-NMR (300 MHz, CDCl$_3$) spectrum of 57**

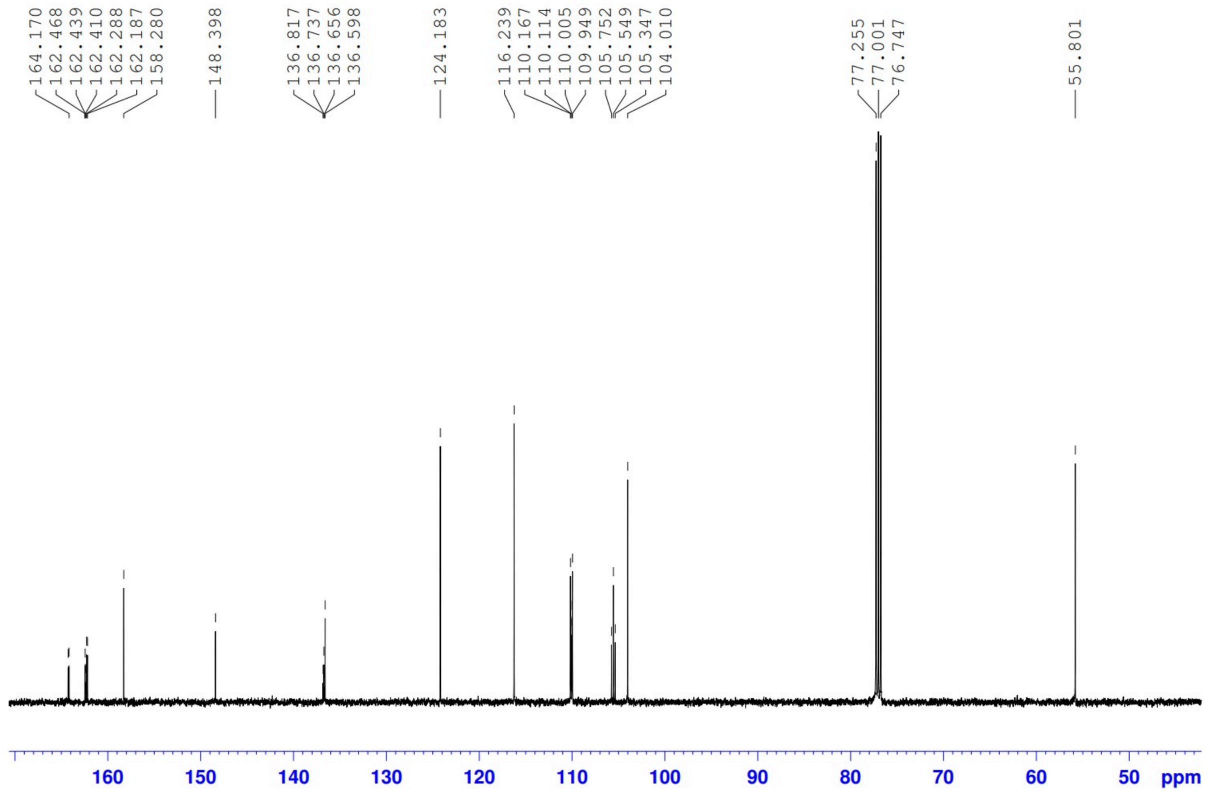

Figure S131. ^{13}**C-NMR (125 MHz, CDCl**$_3$**) spectrum of 57**

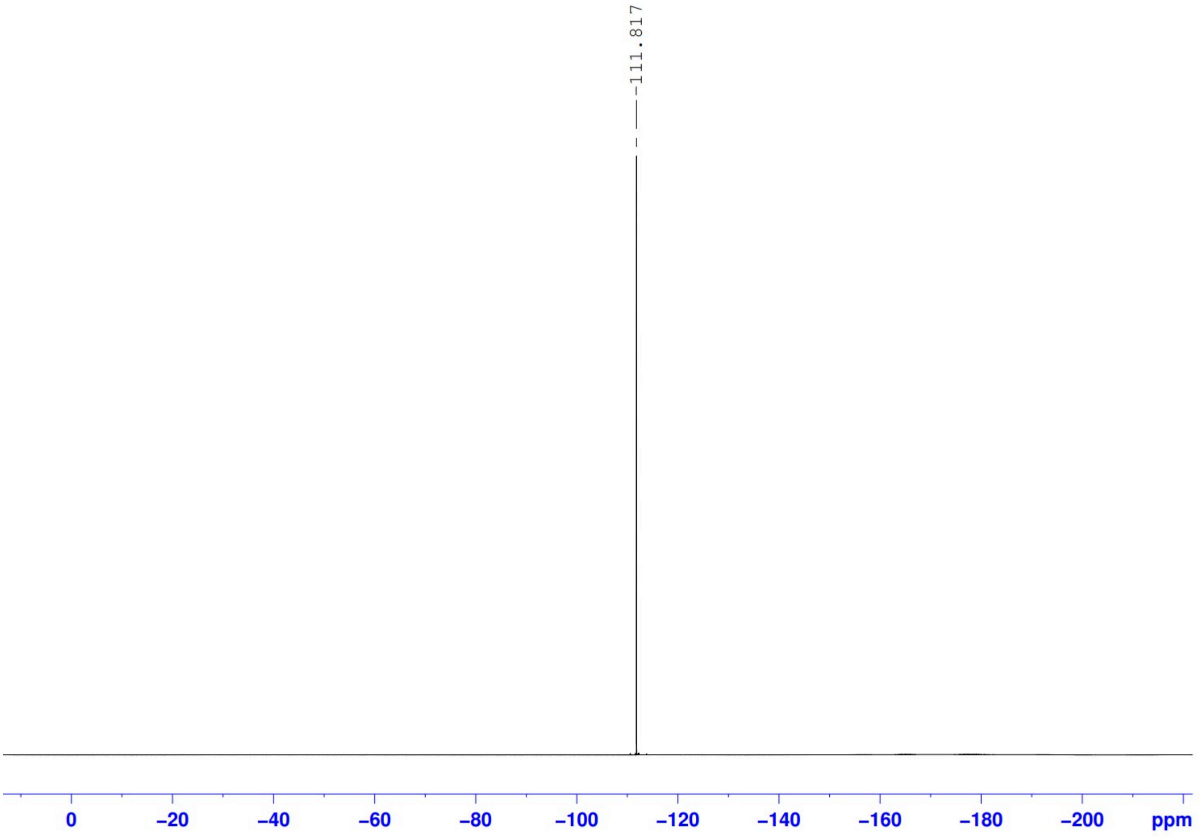

Figure S132. ^{19}**F-NMR (376 MHz, CDCl**$_3$**) spectrum of 57**

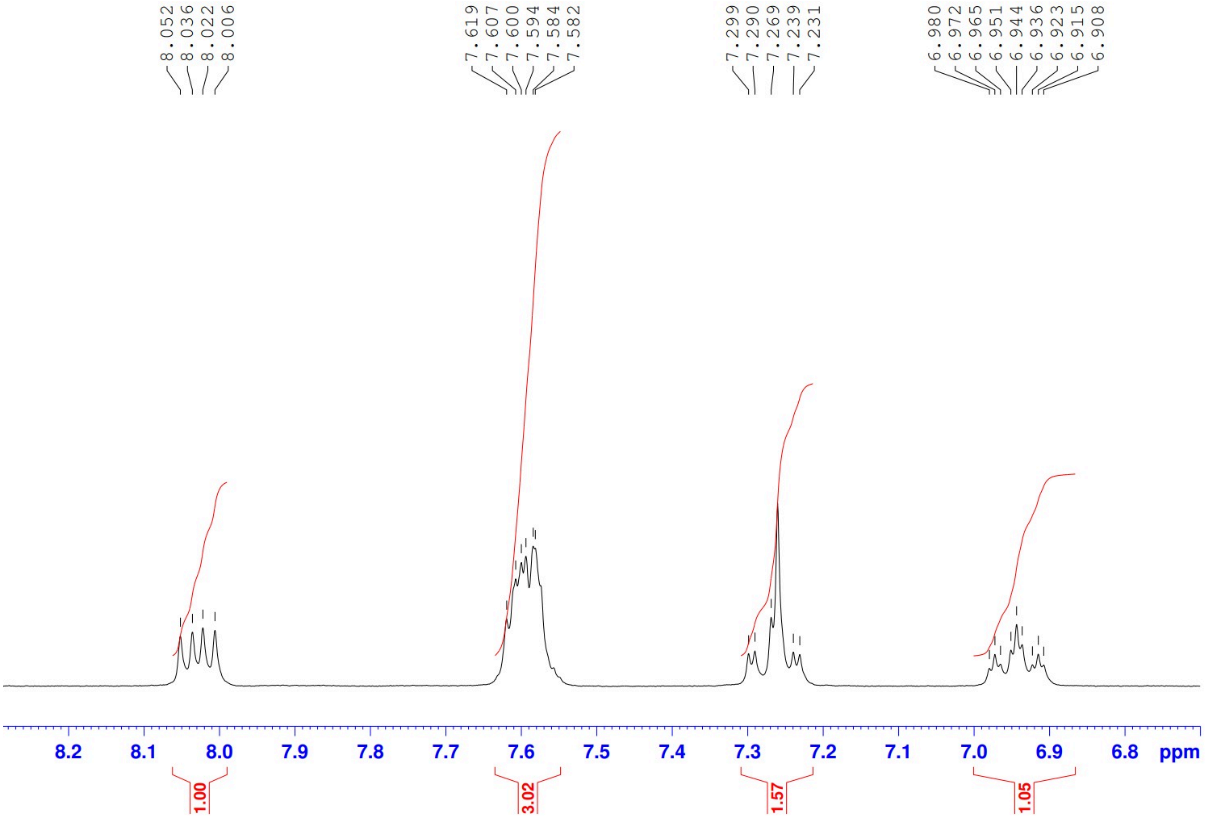

Figure S133. ^{1}H-NMR (300 MHz, $CDCl_3$) spectrum of 58

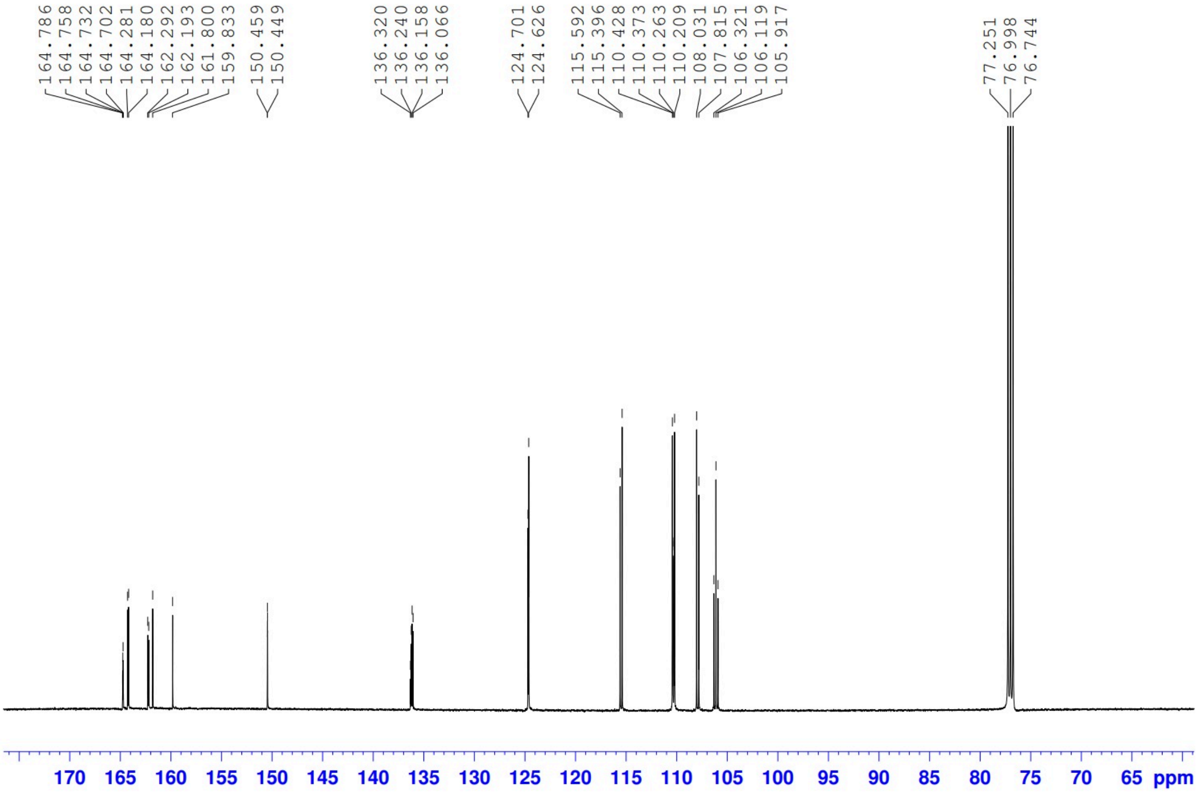

Figure S134. ^{13}C-NMR (125 MHz, $CDCl_3$) spectrum of 58

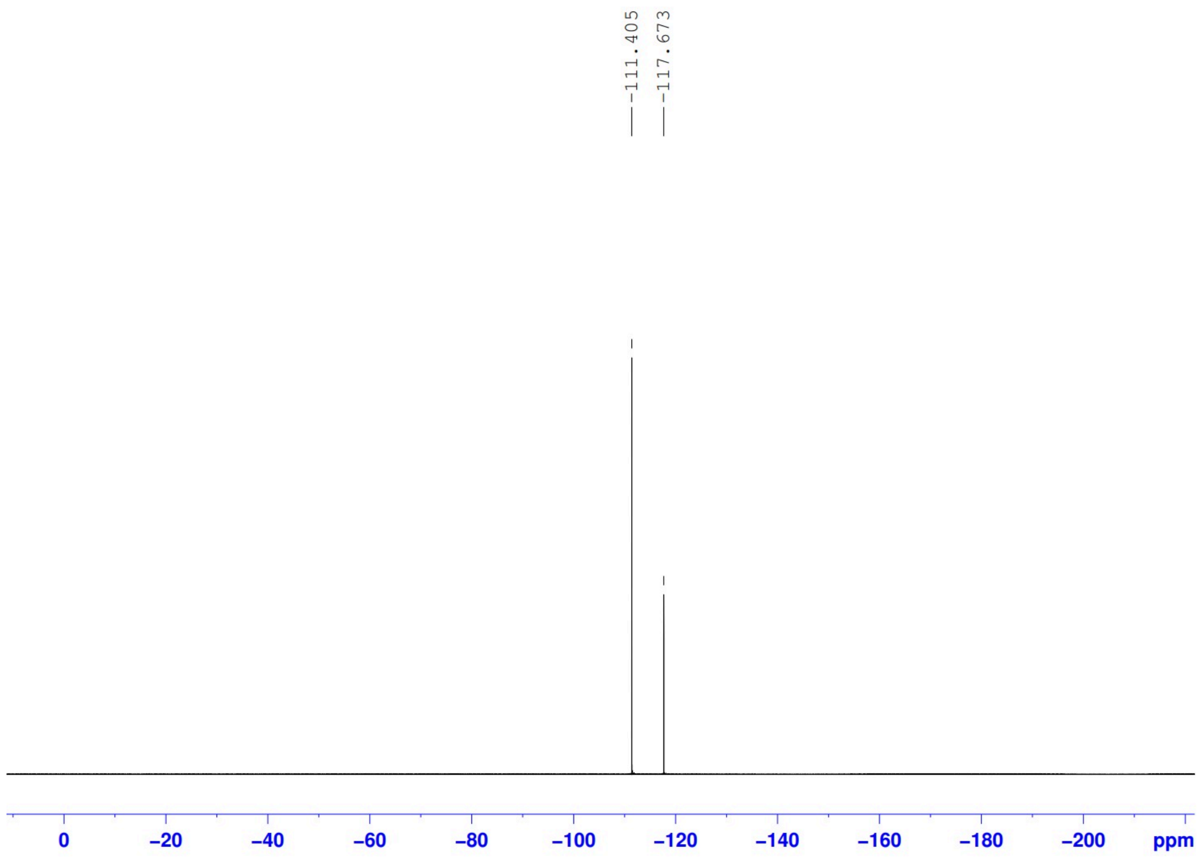

Figure S135. **^{19}F-NMR (376 MHz, $CDCl_3$) spectrum of 58**

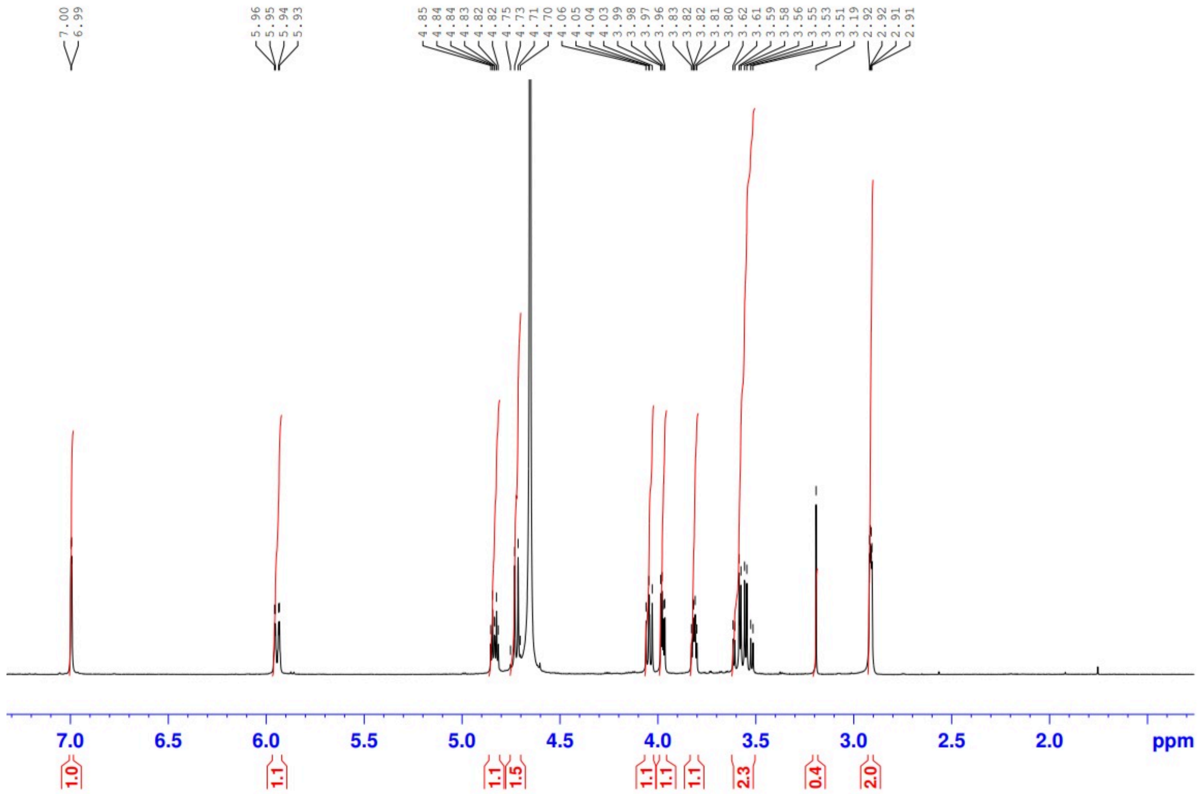

Figure S136.[1]**H-NMR (400 MHz, D_2O) spectrum of *N*-Ribosyldihydronicotinamide (NRH)**

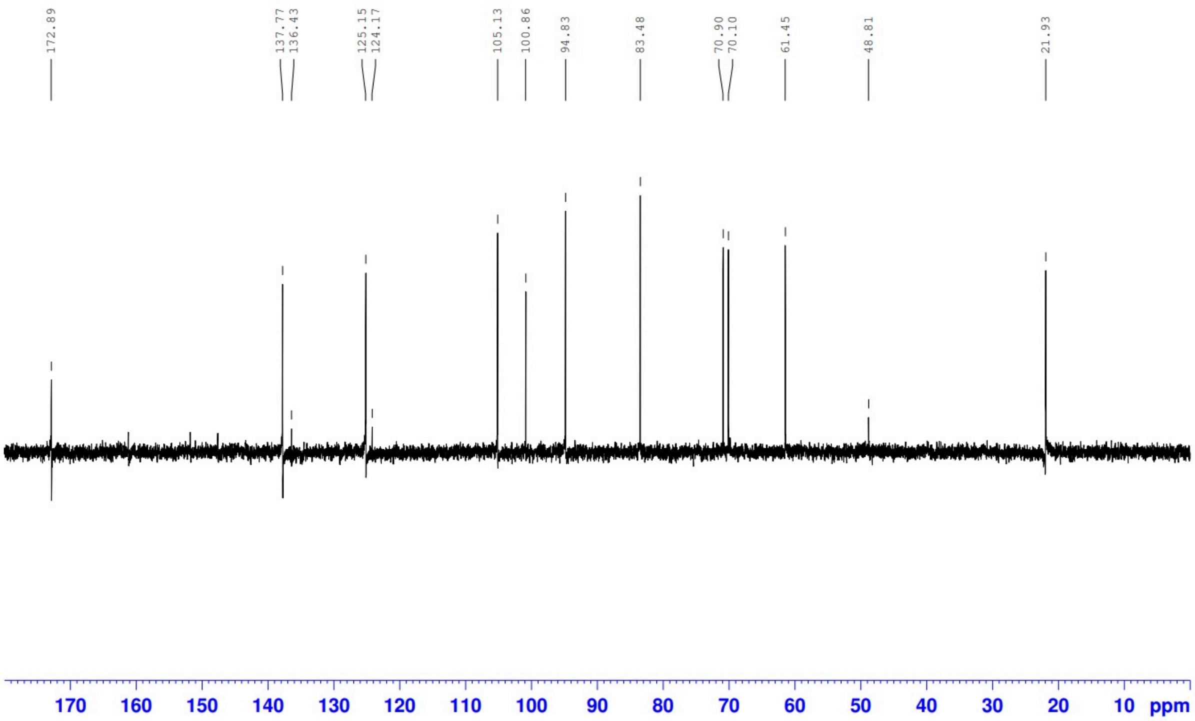

Figure S137. ^{13}**C-NMR (100 MHz,** D_2O**) spectrum of *N*-Ribosyldihydronicotinamide (NRH)**

10.Docking of Benzothiazoles in NQO2 active site

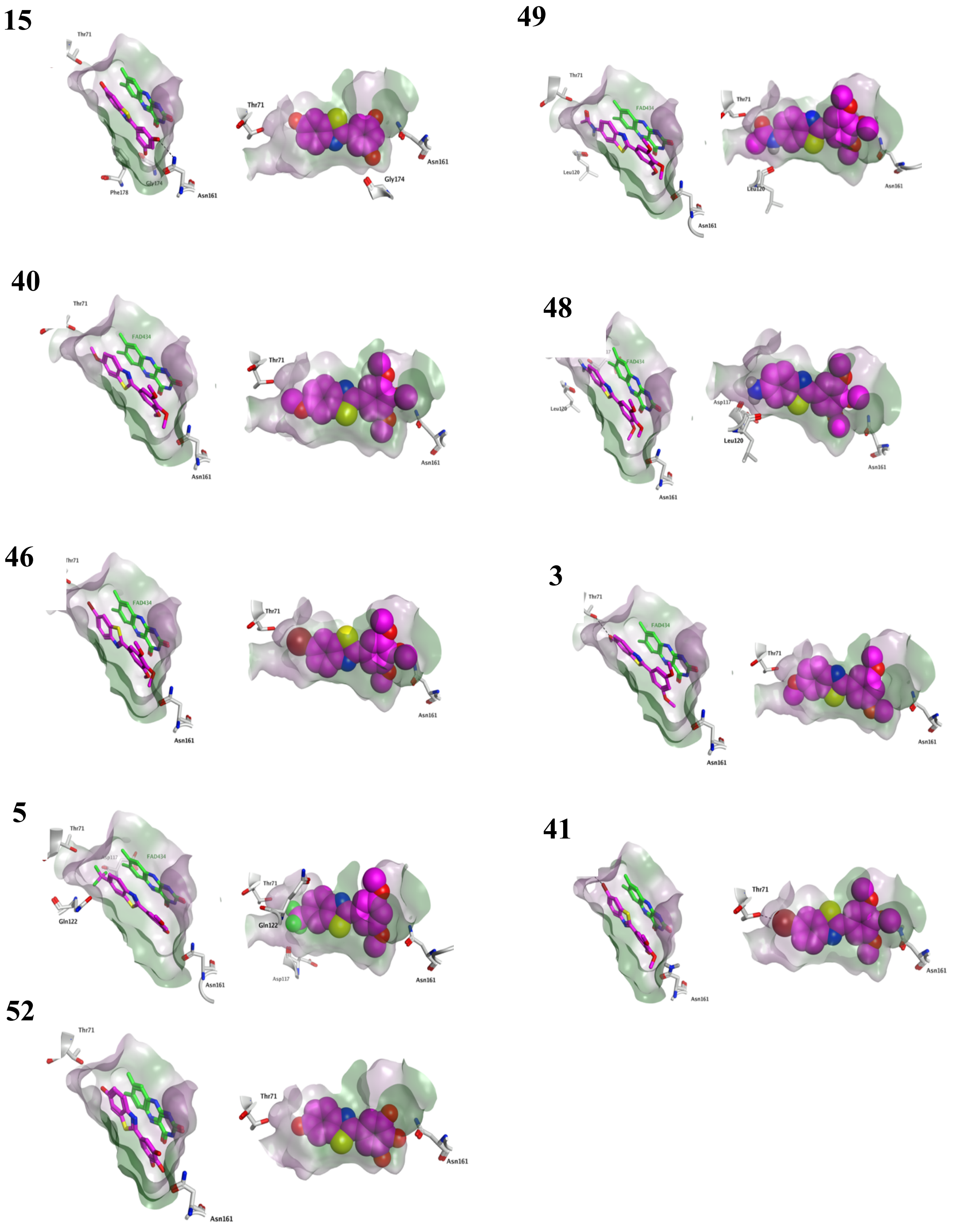

Figure S138. Docked poses of the 9 most potent benzothiazole compounds **15**, **49**, **40**, **48**, **46**, **3**, **5**, **41**, and **52** (listed in order of decreasing potency) in the NQO2 binding site (PDB ID: 1SG0). Stick representation shows π-π stacking of the docked compounds (magenta) with the isoalloxazine ring of FAD (green). Space-filling representation shows the orientation of the docked small molecules in NQO2 binding pocket, where the substituted phenyl ring is located on the right.

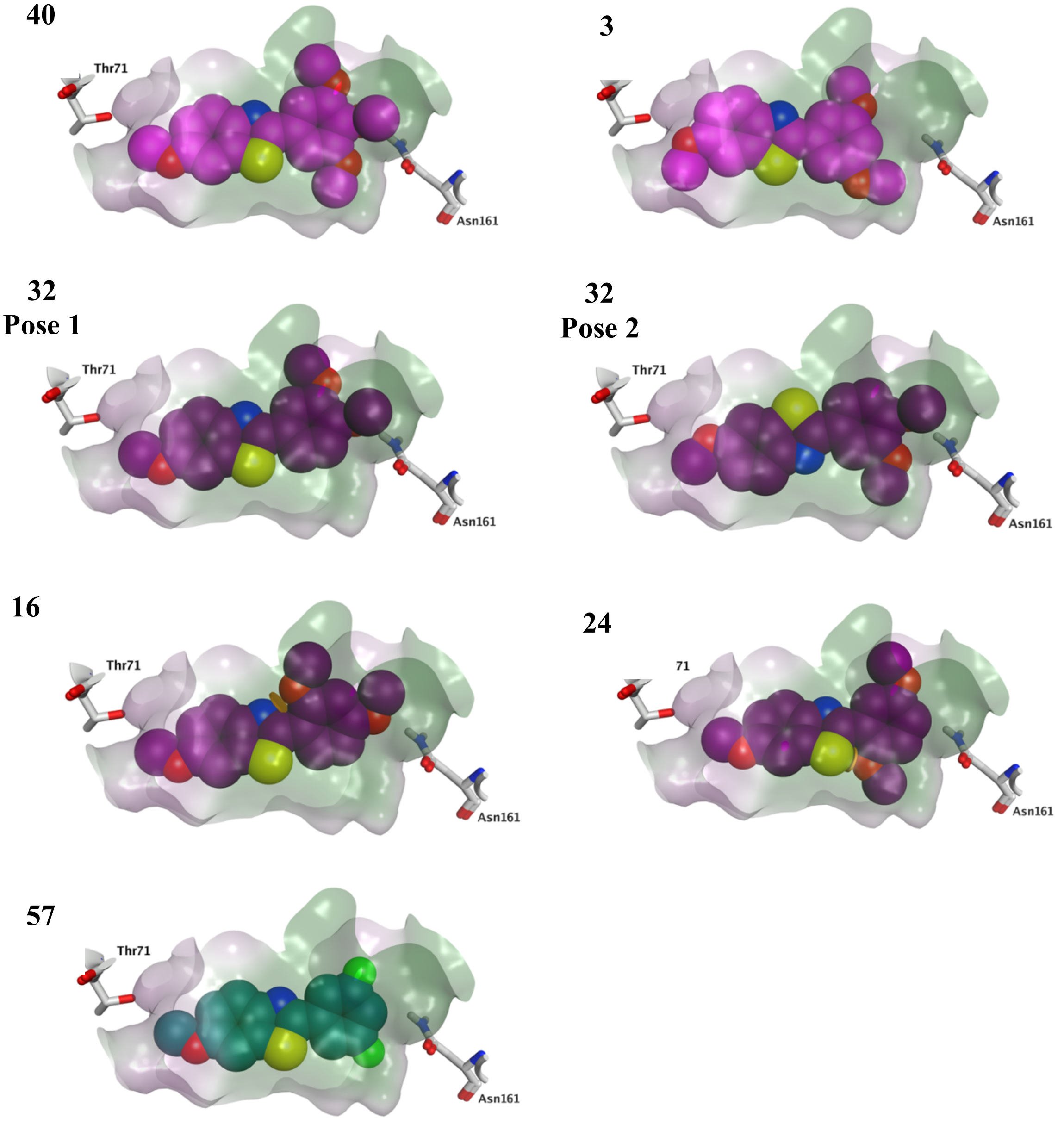

Figure S139. Space- filling representation of 6-methoxy benzothiazoles (compounds **40**, **3**, **24**, **32**, **16**, and **57** listed in order of decreasing activity) docked into the NQO2 binding site (PDB ID: 1SG0). Compounds **40** (3,4,5-trimethoxy phenyl) and **3** (3,5-dimethoxy phenyl) are coloured in magenta, compounds **32** (3,4-dimethoxy phenyl), **16** (2,4-dimethoxy phenyl), and **24** (2,5-dimethoxy phenyl) are coloured in dark magenta, compound **57** (difluoro phenyl) is coloured in dark cyan.
